# Brain areas lipidomics in female transgenic mouse model of Alzheimer's disease

**DOI:** 10.1038/s41598-024-51463-3

**Published:** 2024-01-09

**Authors:** Laura Ferré-González, Ángel Balaguer, Marta Roca, Artemis Ftara, Ana Lloret, Consuelo Cháfer-Pericás

**Affiliations:** 1 Alzheimer’s Disease Research Group, Health Research Institute La Fe, Avda de Fernando Abril Martorell, 106, 46026 Valencia, Spain; 2 Faculty of Mathematics, University of Valencia, Valencia, Spain; 3 Analytical Unit, Health Research Institute La Fe, Valencia, Spain; 4 University of Valencia, Valencia, Spain; 5 Department of Physiology, Faculty of Medicine, University of Valencia, Health Research Institute INCLIVA, Valencia, Spain

**Keywords:** Lipidomics, Lipids, Neurochemistry, Biochemistry, Biomarkers, Neurological disorders, Dementia, Alzheimer's disease

## Abstract

Lipids are the major component of the brain with important structural and functional properties. Lipid disruption could play a relevant role in Alzheimer’s disease (AD). Some brain lipidomic studies showed significant differences compared to controls, but few studies have focused on different brain areas related to AD. Furthermore, AD is more prevalent in females, but there is a lack of studies focusing on this sex. This work aims to perform a lipidomic study in selected brain areas (cerebellum, amygdala, hippocampus, entire cortex) from wild-type (WT, n = 10) and APPswe/PS1dE9 transgenic (TG, n = 10) female mice of 5 months of age, as a model of early AD, to identify alterations in lipid composition. A lipidomic mass spectrometry-based method was optimized and applied to brain tissue. As result, some lipids showed statistically significant differences between mice groups in cerebellum (n = 68), amygdala (n = 49), hippocampus (n = 48), and the cortex (n = 22). In addition, some lipids (n = 15) from the glycerolipid, phospholipid, and sphingolipid families were statistically significant in several brain areas simultaneously between WT and TG. A selection of lipid variables was made to develop a multivariate approach to assess their discriminant potential, showing high diagnostic indexes, especially in cerebellum and amygdala (sensitivity 70–100%, sensibility 80–100%).

## Introduction

Alzheimer's disease (AD) is currently the leading cause of dementia in elderly population^1^. The World Health Organisation estimates that more than 55 million people are affected worldwide. This number is expected to increase in the following years^2^, especially in women, as the incidence rate of AD is higher in women than in men^3^. So, studies focusing on females are of particular interest. Specifically, research focused on the advance in the knowledge of the molecular mechanisms involved in early AD stages, as well as the identification of reliable biomarkers is required.

Nowadays, early AD diagnosis consists of methods based on invasive and costly techniques (cerebrospinal fluid biomarkers, neuroimaging)^4,5^. In general, neuroimaging provides structural and functional information in more advanced stages of the disease. However, there is a lack of minimally invasive, such as blood biomarkers, to obtain brain molecular information in early AD. This knowledge would represent an important advance in early AD diagnosis^6^, the design of clinical trials, and the development of therapeutic drugs.

The main pathophysiological mechanisms involved in AD are the deposition of amyloid-β (Aβ) peptide in plaques and neurofibrillary tangles mainly formed by hyperphosphorylated Tau protein. Neurofibrillary tangles appear first in the entorhinal cortex and then in the hippocampus, and correlate with neuronal death and thus neurodegeneration^7,8^. These pathological alterations are related to impairment in memory capacity, language, reasoning, social behaviour and spatial cognition^9,10^. The amygdala is affected very early, resulting in neuropsychiatric symptoms and functional deficits, which contribute greatly to the disability associated with AD^11,12^. Moreover, cerebellar affectation is associated with cognitive and neuropsychiatric deficits in the stage of mild cognitive impairment^13,14^. So, some post-mortem studies in the brain showed that different brain areas were affected in AD development.

Regarding brain composition, lipids represent 50–60% of its dry weight^15^. Therefore, an extensive analysis of the brain lipid composition in AD would allow us to study the pathogenesis of the disease and its pathophysiological changes^16–19^. Additionally, a previous study in peripheral blood described that some lipid biomarkers predict the phenoconversion from mild cognitive impairment to AD^20^. In fact, lipids act as signalling molecules, and energy sources, in synaptogenesis, neurogenesis and impulse conduction, among other processes^21^.

Recent research has focused on brain lipidomic studies in the AD mouse model. In this sense, some lipids showed statistically significant different levels between AD and non-AD groups. Specifically, an increase in gangliosides^22,23^, sphingomyelins (SM)^24,25^, lysophospholipids (LPL)^26,27^ and monounsaturated fatty acids^28,29^ and a decrease in sulfatides^23,24^ were observed in AD mice compared to WT. However, these studies showed a wide variety of experimental conditions. First, different mouse models were used in these studies (e.g. APPswe/PS1dE9^25,30,31^, Tg2576^22^, 5xFAD^32^, PLB4^33^, SAMP8^28^, 3xTg-AD^34^), being APPswe/PS1dE9 one of the most studied and largely described. Second, most of these studies were conducted on the whole brain^32,35^ with few having assessed specific brain areas (e.g. hippocampus^24,36^, cortex^34,37^), it would therefore be interesting to consider other areas simultaneously. Third, the mice age ranged from 2 to 24 months^28,38^. In addition, a wide variety of sample pre-treatments were performed, such as manual or mechanical brain tissue homogenisation and lipids extraction, mostly with solvents that do not follow the philosophy based on sustainable chemistry (e.g. chloroform)^22,39^. Finally, most of the studies were carried out on male mice^28,35^ or on both sexes, without focusing on finding differences between sexes^32,38^.

The aim of this work is to perform a lipidomic study in four brain areas (cerebellum, amygdala, hippocampus, entire cortex) of wild type (WT) and transgenic APPswe/PS1dE9 (TG) female mice, identifying the main brain lipids altered in females due to early AD, in order to elucidate the main brain lipids impaired in early disease development.

## Materials and methods

### Animals

APPswe/PS1dE9 (line 85) TG and WT female mice from the same colony and littermates were bred and maintained under standard housing conditions in a 12:12-h dark–light cycle at 23 ± 1 °C and 60% relative humidity at the Animal Facilities Service from the University of Valencia (Valencia, Spain). Mice were housed in groups (2–6 mice per cage), fed with standard diets and free access to water ad libitum.

**Figure 1:**
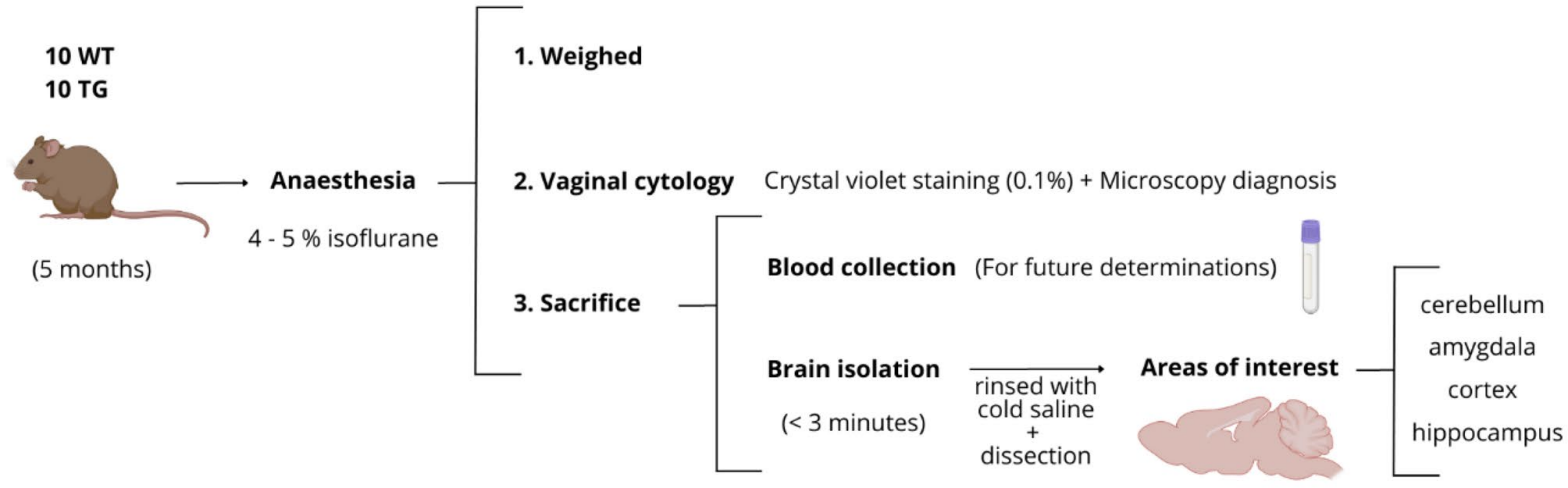
Experimental procedure with mice from anaesthesia to brain isolation. (Created with BioRender.com and Canva.com).

At the age of 5 months (± 4 days), 10 TG mice and 10 WT littermates were anaesthetised with isoflurane (4–5%) and sacrificed by cervical dislocation. This age corresponds to an early AD stage, in which amyloid deposits and cognitive impairment are not observed yet^40,41^. After weighing the animals, blood was collected in a heparinized tube for future determinations, and the brain was immediately removed from the skull to separate the cerebellum (CB), amygdala (AM), hippocampus (HPC) and the entire cortex (CX) from both hemispheres. Furthermore, vaginal cytology was performed by crystal violet staining (0.1%, w/v) and diagnosed by microscopy Leica DMD 108 (Wetzlar, Germany) to determine the stage of the estrous cycle, in which the female mice were found (Fig. 1).

All experimental methods were approved by the Ethics Committee for Experimentation and Animal Welfare at University of Valencia (reference: A202203291754, date: 22–08–2022), and performed in accordance with relevant animal experimentation guidelines and regulations (RD53/2013 on the Protection of Animals used for experimentation and other scientific purposes Ministry of the Presidency, Spain). Also, this study was carried out following the ARRIVE guidelines (https://arriveguidelines.org).

### Colony genotyping

After weaning, ear or tail tissue samples were taken from the mice to isolate genomic DNA for PCR genotyping with specific primers, using the QIAamp Fast DNA Tissue Kit (QIAGEN, Germany). APPswe/PS1dE9 mice (TG) were identified by the presence of a ≈ 500 bp band.

### Brain tissue sampling

The brain areas (CB, CX, AM, HPC) were quickly dissected from isolated brains with less than 3 min post-mortem delay. Each area was rinsed with cold saline, frozen in liquid nitrogen and stored at -80 °C in a clearly labelled tube until analysis.

### Brain sample treatment

**Figure 2:**
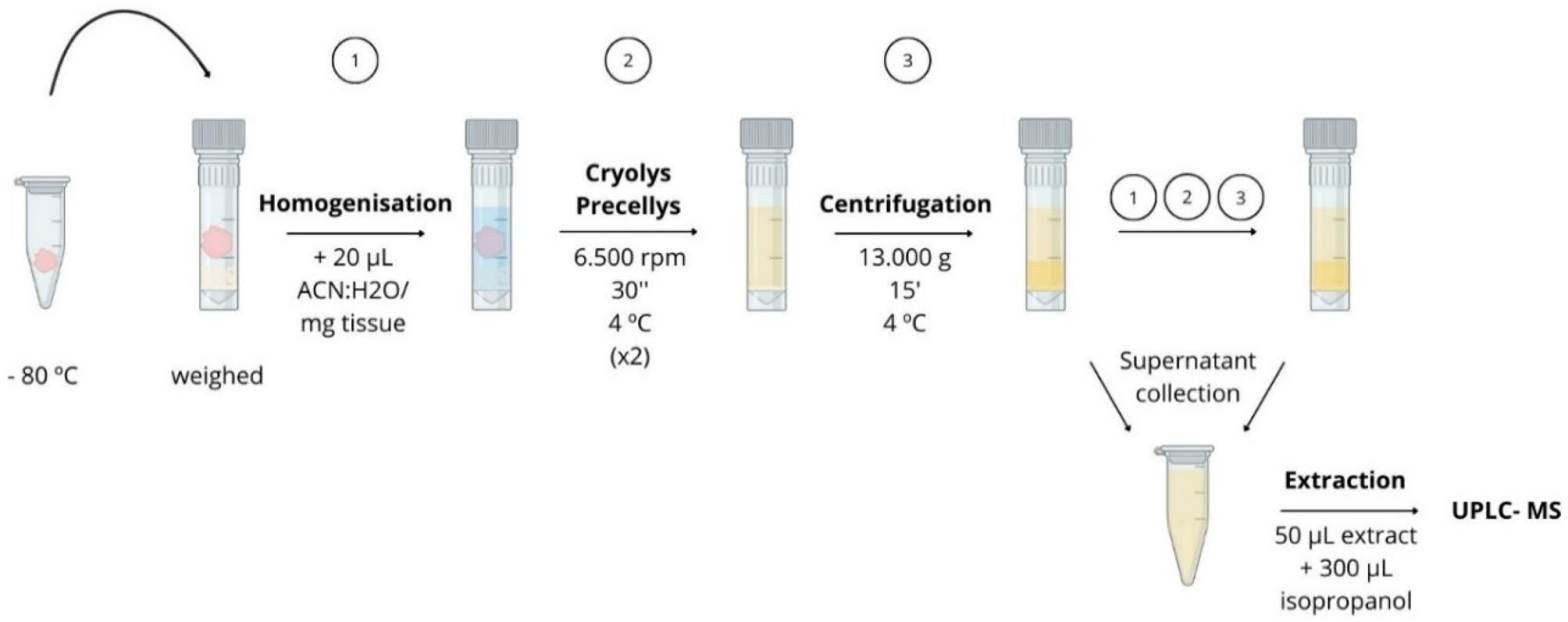
Brain tissue homogenisation and lipid extraction process. (Created with BioRender.com and Canva.com).

Frozen tissues were accurately weighed and homogenised with Cryolys Precellys Evolution Homogenizer in 20 μL of acetonitrile:water (50:50, v/v) per 1 mg of tissue in 2 mL Precellys tubes (Precellys Lising Kit, CK14), in order to obtain the same tissue concentration for all the samples. After that, they were centrifuged in duplicate (6500 rpm, 30 s, 4 °C) with 10 s of rest in between, and then they were centrifuged at 13.000 *g* for 15 min at 4 °C, and the supernatant was collected. The remaining pellet was subjected to the above procedures once again. Finally, the supernatants were put together, and these extracts were aliquoted (see Fig. 2).

Lipid extraction was performed by adding 300 μL of isopropanol to 50 μL of the extract, vortexed and left to stand for 30 min at − 20 °C. Later, it was centrifuged (13.000 *g*, 15 min, 4 °C) and the supernatant was collected; from which 90 μL were transferred to a 96-well injection plate. After that, 10 μL of the internal standard mix solution (MAG(17:0), LPC(17:0), Cer(d18:1/17:0), DAG(17:0/17:0), PE(17:0/17:0), PC(17:0/17:0), TAG(17:0/17:0/17:0), CE(17:0), for the positive ionization mode); LPC(17:0), Cer(d18:1/17:0), PE(17:0/17:0), PG(17:0/17:0), PC(17:0/17:0), PS(17:0/17:0), for the negative ionization mode) (3 µg mL^−1^, each compound) were added to each sample (Fig. 2).

### Liquid chromatography coupled to mass spectrometry analytical method

For the samples analysis, Ultra-Performance Liquid Chromatography equipment (UPLC) coupled to a high-resolution mass spectrometer (MS) with Orbitrap UPLC-QExactive Plus detector (UPLC-TOF/MS-Orbitrap QExactive Plus MS) available at the Analytical Unit of the Instituto de Investigación Sanitaria La Fe (IISLaFe, Valencia, Spain) was used.

The chromatographic and mass spectrometric conditions were those established in the standard procedures of the Analytical Unit. Briefly, the analytical column was an Acquity UPLC CSH C18 (100 × 2.1 mm, 1.7 μm) from Waters. The mobile phase in the positive ionization mode was acetonitrile/water (60:40, v/v) with ammonium formate (10 mM) (A), and isopropyl alcohol/acetonitrile (90:10, v/v) with ammonium formate (10 mM) (B); while in the negative ionization mode, it was acetonitrile/water (60:40, v/v) with ammonium acetate (10 mM) (A) and isopropyl alcohol/acetonitrile (90:10, v/v) with ammonium acetate (10 mM) (B). The flow rate was 400 μL min^-1^, the column temperature was 65 °C, and the injection volume was 5 µL.

To avoid intra-batch variability, as well as to ensure the quality and reproducibility of the analysis results, some aspects were considered such as the injection in random order, analysis of at least 5 quality control samples (QC) at the beginning of the sequence to condition the column and equipment, a QC analysis in MS mode (Full MS) every 5–7 samples, and at least two QC analysis in Data Independent Fragmentation mode (DIA) and Data Dependent Fragmentation mode (DDA) for annotation purpose. In general, the intra-batch coefficients of variation of the internal standards were between 10 and 21%. Finally, data pre-processing and lipid species were annotated with the LipidMSv3 package^42^.

### Lipidomic data processing

To analyse the quality of the lipidomic results, the variability of the internal standards in the samples was evaluated in each sequence to detect possible problems in the injection or sample preparation, as well as intra-batch variability.

Data processing, peak picking, retention time alignment and peak integration were performed by using the LipidMSv3 R package to obtain annotated lipids in the samples. Then, the data were filtered eliminating these molecular features with coefficients of variation in the QCs > 30% and number of zero > 60% in at least one of the two groups compared. Finally, the data were normalised by applying a Median Fold Change normalization method and grouped into lipid classes.

### Statistical analysis

Univariant analysis was carried out using IBM Statistical Package for the Social Sciences software version 23.0 (SPSS, Inc., Chicago, IL, USA). In descriptive analysis, categorical variables were expressed as frequencies and percentages (%), and numeric variables were expressed as medians and interquartile ranges (IQR). In all the cases, the statistical significance was set at a p-value ≤ 0.05. Furthermore, differences among medians were analysed by using the non-parametric test (Mann–Whitney U test) and described by the fold change.

Multivariate analysis was based on Lasso regression (least absolute shrinkage and selection operator)^43^, it was adjusted to identify the most influential variables in the differentiation between TG and WT groups. Genotype was dummified and used as the response variable. This method forces the shrinkage of the parameters to zero, potentially performing variable selection at the model-fitting step. Therefore, the lipids with the greatest influence are selected (inferential approach). The penalization factor (Fraction of the final L1 norm) for the Lasso regression was selected using Leave-One-Out Cross-Validation. The selection of variables with Lasso regression was carried out for the lipids detected in the different brain areas, individually and all areas simultaneously, and for each detection mode (positive ionization, negative ionization).

From the selected lipids, a Partial least squares Discriminant Analysis (PLS-DA) was built in the different brain areas (individually, simultaneously) to evaluate the potential of the selected variables, the quality indexes (by Leave-One-Out Cross-Validation) and to make inferences about the most important ones (VIP scores).

This multivariate analysis was performed using R (version 4.2.2), R packages lars (version 1.3) and mdatools (version 0.13.1), with IDE R-Studio (version 2022.12.0 Build 353).

## Results

### Animal model description

**Figure 3:**
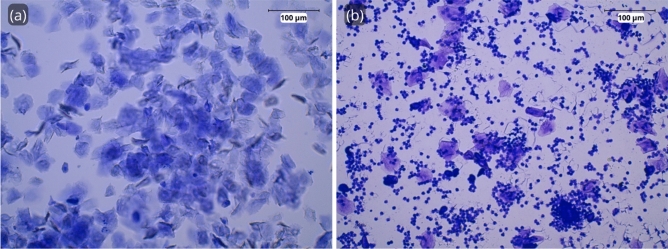
Microscopic photographs of vaginal cytology were performed on the mice. Estrus stage (**a**) where principally cornified epithelial cells were visible, and diestrus stage (**b**) where mainly leukocytes are present. Black scale bar size = 100 μm.

As shown in Table 1, there were no differences between WT and TG groups in terms of age, weight and the mouse estrous cycle. Specifically, all the female mice were between the estrus and diestrus stages (Fig. 3).

**Table 1:** Mice variables description.

Variable		WT (n = 10)	TG (n = 10)	p value (Mann Whitney *U* test)
Age (days, median (IQR))		164 (161–164)	162 (161–164)	0.58
Age (months, median (IQR))		5.39 (5.29–5.39)	5.32 (5.29–5.39)	0.58
Weight (grams, median (IQR))		42.25 (37.98–44.35)	37 (27.35–41.73)	0.08
Estrous cycle (n, (%))	Estrus	4 (40%)	6 (60%)	0.37
	Diestrus	6 (60%)	4 (40%)	

*IQR* inter-quartile range.

### Lipids univariate analysis

As can be seen in Table 2, the major lipids in WT and TG mice belong to the glycerophospholipid (GP) family in all the brain areas (WT: 70.8–79.5%, TG: 70.6–81.8%), followed by the fatty acid (FA) family (WT: 7.2–17.9%, TG: 6.8–17.8%). It should be noted that FA family in CB is particularly low concerning the other areas; while the other families (GL, GP, SP, ST) showed similar levels in all the brain areas. In general, not significant differences were observed between WT and TG mice.

**Table 2:** Percentage (mean) of lipid class in each brain area and mice group*.*

Lipid family	CB		AM		HPC		CX	
	WT	TG	WT	TG	WT	TG	WT	TG
FA (%)	7.2	6.8	15.0	14.2	13.3	15.9	17.9	17.8
GL (%)	10	8.3	7.8	7.8	6.8	7.6	8.0	8.2
GP (%)	79.5	81.8	73.0	73.5	75.6	72.0	70.8	70.6
SP (%)	3.3	3.1	4.1	4.5	4.2	4.5	3.3	3.4
ST (%)	< 0.01	< 0.01	< 0.01	< 0.01	< 0.01	< 0.01	< 0.01	< 0.01

*FA* fatty acid, *GL* glycerolipid, *GP* glycerophospholipid, *SP* sphingolipid, *ST* sterol lipid.

Regarding saturation degree, the different lipid groups showed similar levels in all the brain areas (approximately 15% of saturated lipids (SAT), 32% of monounsaturated lipids (MUs), 53% of polyunsaturated fatty acids (PUs)). In addition, not significant differences were observed between WT and TG mice (see Table 3).

**Table 3:** Percentage (mean) of lipids grouped by saturation degree in each brain area and mice group*.*

Saturation degree	CB		AM		HPC		CX	
	WT	TG	WT	TG	WT	TG	WT	TG
SATs (%)	12.7	12.7	16.1	16.6	15.6	15.7	15.1	15.5
MUs (%)	33.2	35.0	29.9	29.6	33.5	32.8	29.5	30.1
PUs (%)	54.0	52.4	54.0	53.8	50.9	51.5	55.4	54.3

*SAT* saturated lipid, *MU* monounsaturated lipid, *PU* polyunsaturated lipid.

**Figure 4:**
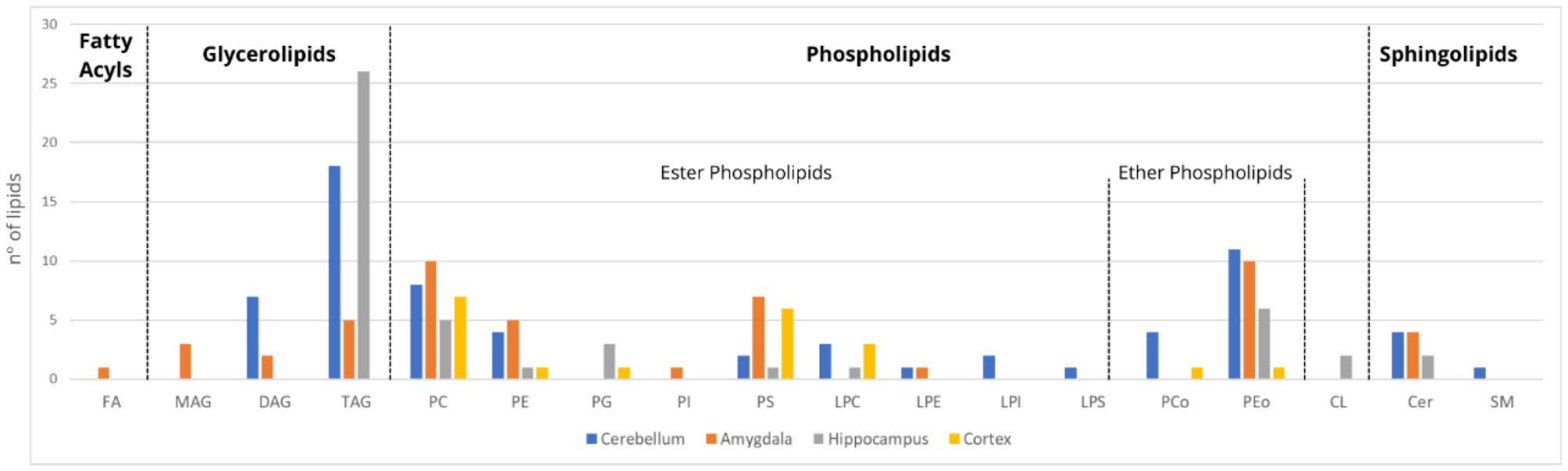
Number of lipids with statistically significant differences between mice groups in the cerebellum (blue), amygdala (orange), hippocampus (grey) and cortex (yellow). Lipids are classified into subfamilies. *Cer* ceramide, *CL* cardiolipin, *DAG* diacylglycerol, *FA* fatty acid, *LPC* lysophosphatidylcholine, *LPE* lysophosphatidylethanolamine, *LPI* lysophosphoinositol, *LPS* lysophosphatidylserine, *MAG* monoacylglycerol, *PC* phosphatidylcholine, *PCo* ether-linked phosphatidylcholine, *PE* phosphatidylethanolamine, *PEo* ether-linked phosphatidylethanolamine, *PG* phosphoglyceride, *PI* phosphatidylinositol, PS phosphatidylserine, *SM* sphingomyelin, *TAG* triacylglycerol.

A total of 250 lipid variables were detected in the positive ionisation mode and 196 in the negative ionisation mode (see Tables S1–S8 in Supplementary Material). From them, some lipids showed statistically significant differences between the two mice groups in CB (n = 66), AM (n = 49), HPC (n = 47) and CX (n = 20) (see Figs. S1, S2 in Supplementary Material). As can be seen in Fig. 4, FAs showed statistically significant differences between groups only in the AM, with decreased levels in AD. Regarding glycerolipids (GLs), a high number of statistically significant triacylglycerols (TAG) were detected in all brain areas except the cortex, especially in HPC and CB. In the AD group, TAG levels were increased only in HPC but decreased in AM and CB. Moreover, a low number of diacylglycerols (DAG) were found in CB and AM with reduced levels in AD; and monoacylglycerols (MAG) were only detected in the AM with reduced levels in AD. Moreover, the highest number of statistically significant lipids in all brain areas belonged to the phospholipid (PL) family, specifically phosphatidylcholines (PCs), phosphatidylethanolamines (PEs), phosphatidylserines (PSs) and ether-linked phosphatidylethanolamines (PEos). Interestingly, phosphoglycerides (PGs) only showed statistically significant differences in HPC and CX while lysophosphatidylcholines (LPCs) lipids, showed statistically significant differences in all brain areas except the AM. Also, a few lysophosphoinositols (LPIs) and lysophosphatidylserine (LPSs) were detected in the cerebellum; and only phosphoinositols (PIs) in the AM. Regarding PLs (PC, PE, PS), ether-linked PL (PCo, PEo) and LPLs (LPC, LPE, LPI, LPS), all levels were higher in the CB of AD mice. In the AM, the PLs (PC, PE, PEo) and LPE were higher and other LPs (PI, PS) were lower in the disease. In contrast, in HPC the levels of PLs (PC, PE, PG, PEo) and cardiolipins (CL), were found to be decreased, but LPC and PS levels were higher in AD. Moreover, in CX, LPC and PLs (PC, PS, PCo, PEo) were decreased and only PG were increased in AD females. Finally, in the sphingolipid family, ceramides (Cer) were detected in all brain areas except the cortex, with higher levels in AD, and statistically significant SM were only detected in CB with lower levels in AD mice.

Some lipids (n = 12) from glycerolipids (GLs), phospholipids (PLs) and sphingolipids (SPs) families were found to be statistically significant in several brain areas simultaneously (see Table 4). In fact, PEo(36:5) (M + Na) was found in CB, AM and HPC. Also, lipids from other families (TAG, PC, PE, LPC, LPE, Cer) were significant in two brain areas. In general, the fold change values were between 0.70 and 1.53.

**Table 4:** Fold change values for the statistically significant lipids between mice groups in some brain areas simultaneously (Fold change (p-value).

Lipid variable	m/z	∆m (ppm)	Adduct	Brain areas			
				CB FC (p-value)	AM FC (p-value)	HPC FC (p-value)	CX FC (p-value)
TAG(16:1/16:1/16:1)	823.6784	0.2428	[M + Na]^+^	–	0.90 (< 0.001)	1.11 (0.01)	–
PC(18:1/20:4)	830.5657	1.5651	[M + Na]^+^	–	1.30 (0.01)	–	0.88 (0.04)
PC(36:2)	770.5722	2.2061	[M-CH3]^-^	1.39 (0.01)	–	–	1.09 (0.04)
PC(33:2)	744.5540	0.2686	[M + H]^+^	1.18 (0.04)	–	0.85 (0.04)	–
LPC(16:0)	496.3396	0.4029	[M + H]^+^	1.17 (0.04)	–	–	0.92 (0.02)
LPE(18:0)	480.3105	2.0819	[M-H]^-^	1.36 (0.01)	1.44 (0.02)	–	–
PEo(16:1/20:4)	722.5148	2.4913	[M-H]^-^	1.24 (0.03)		0.75 (0.02)	–
PEo(18:1_18:2)	728.5592	0.5490	[M + H]^+^	1.23 (0.04)	1.22 (0.04)	–	–
PEo(16:1/22:5)	748.5300	1.8703	[M-H]^-^	1.37 (0.01)	1.27 (0.01)	–	–
PEo(36:4)	726.5435	0.4129	[M + H]^+^	1.53 (0.01)	–	0.84 (0.04)	–
PEo(36:5)	746.5113	2.4112	[M + Na]^+^	1.20 (0.04)	1.36 (< 0.001)	0.70 (0.03)	–
Cer(d18:1/16:0)	596.5274	2.5145	[M + CH3COO]^-^	–	0.88 (0.04)	1.18 (0.04)	–

**∆***m* absolute mass error, *FC* Fold change TG median/WT median, *m/z* mass-to-charge ratio.

In general, the lipids with the greatest fold change, PC(20:1/22:6) (M + H) and PS(18:1_20:1) (M-H) showed lower levels in the TG group than in WT mice; while PE(38:2) (M-H), LPE(18:0) (M-H), LPI(18:0) (M-H), PEo(36:4) (M + H), PEo(38:4) (M + H), Cer(d18:1/16:0) (M-H), Cer(d18:1/18:0) (M-H, M + CH3COO) showed higher levels in the TG group than in WT mice.

### Lipids multivariate analysis

A multivariate statistical analysis was performed to select lipid variables that could discriminate between TG and WT mice, using a method based on Lasso regression. For the positive ionization mode, the method selected a group of lipid variables (all brain areas simultaneous analysis (n = 25), CB (n = 11), AM (n = 4), HPC (n = 3), CX (n = 10)), which belonged to the subfamilies MAG, DAG, TAG, PC, PE, PG, PCo, Peo, LPC, Cer and SM. For the negative ionization mode, the method selected some lipid variables (all areas simultaneous analysis (n = 23), CB (n = 11), AM (n = 10), HPC (n = 5), CX (n = 2)), which belonged to the subfamilies FA, PC, PE, PG, PS, LPE, LPI, LPS, PEo, CI, and Cer (see Tables S9, S10 in Supplementary Material).

From the selected lipid variables, some PLS-DA models were built for each ionization mode and brain area to evaluate their discriminant potential (see Figs. S3–S7 in Supplementary Material). As can be seen in Table 5, the developed PLS-DA models showed high sensitivity (70–100%), specificity (80–100%), positive predictive value (PPV, 81.8–100%), negative predictive value (NPV, 75–100%) and accuracy (80–100%).

**Table 5:** Diagnosis indexes for the PLS-DA models developed for each ionization mode and brain area.

Ionization mode	Areas	Sensitivity (%, 95% CI)	Specificity (%, 95% CI)	PPV (%, 95% CI)	NPV (%, 95% CI)	Accuracy (%, 95% CI)
POSITIVE	All areas	89.7 (76.4–95.9)	87.5 (73.9–94.5)	87.5 (73.9–94.5)	89.7 (76.4–95.9)	88.6 (79.7–93.9)
	CB	100 (72.2–100)	100 (72.2–100)	100 (72.2–100)	100 (72.2–100)	100 (83.9–100)
	AM	100 (70.1–100)	80 (49–94.3)	81.8 (52.3–94.9)	100 (67.6–100)	89.5 (68.6–97.1)
	HPC	70 (39.7–89.2)	90 (59.6–98.2)	87.5 (52.9–97.8)	75 (46.8–91.1)	80 (58.4–91.9)
	CX	90 (59.6–98.2)	100 (72.2–100)	100 (70.1–100)	90.9 (62.3–98.4)	95 (76.4–99.1)
NEGATIVE	All areas	87.2 (73.3–94.4)	90 (76.9–96)	89.5 (75.9–95.8)	87.8 (74.5–94.7)	88.6 (79.7–93.9)
	CB	100 (72.2–100)	100 (72.2–100)	100 (72.2–100)	100 (72.2–100)	100 (83.9–100)
	AM	100 (70.1–100)	100 (72.2–100)	100 (70.1–100)	100 (72.2–100)	100 (83.2–100)
	HPC	90 (59.6–98.2)	90 (59.6–98.2)	90 (59.6–98.2)	90 (59.6–98.2)	90 (69.9–97.2)
	CX	80 (49–94.3)	90 (59.6–98.2)	88.9 (56.5–98)	81.8 (52.3–94.9)	85 (64–94.8)

*AM*: amygdala, *CB* cerebellum, *CI* confidence interval, *CX* cortex, *HPC* hippocampus, *NPV* negative predictive value, *PPV* positive predictive value.

## Discussion

A lipidomic study in different brain areas of WT and TG female mice has been carried out, taking into account their phase of the estrous cycle. Some differences in terms of brain lipid levels in the early stages of AD have been observed. Specifically, it has been evaluated from univariant and multivariant approaches. To our knowledge, this is the first lipidomic study performed in different brain areas to advance the knowledge of the main molecular pathways involved in early disease development, as well as to propose potential blood biomarkers.

First, the CB has been poorly studied, but there is some evidence that it modulates cognition and emotions^14^. Within the family of SPs, a decrease in SM levels and an increase in Cer levels were observed, similar to previous studies conducted in this area^31,44–46^. Regarding the GLs (DAG and TAG), a decrease was observed in the AD compared to the WT group, while other studies carried out in other areas showed increasing levels^47–49^, decreasing levels^50,51^ and no significant differences between groups using the same AD murine model, but in a more advanced AD stage^52^. As for the GPs family, we observed a generalised increase in PLs and LPS, as did another study in the same TG mouse model^53^ and a different AD model^45^.

Second, in the AM, as in CB, it was obtained that decreasing levels in SM and increasing levels in Cer in TG mice compared to WT. Also, lower levels were found for FA in AD than in WT mice. For the GPs family, some lipids were increased, and others were decreased. In general, the AM is difficult to isolate, so only one work was found, in which a detection of altered lipids in AD was performed. However, its results did not corroborate our findings, probably due to the use of a different mouse model, where only males were included, and at a later stage of the disease, where they already had cognitive impairment^54^.

Third, the HPC is the most studied brain area due to its high involvement in the disease^30^. As in CB and AM, an increase in Cer levels was observed, similar to previous studies^24,31,55^. So, Cers could be considered a key factor in the pathophysiology of AD, as an increase in these lipids has been associated with the activation of cell death and neuroinflammatory pathways^56,57^, with neuronal toxicity generated by Aβ aggregation^58,59^ and with tau hyperphosphorylation^56^. For GLs, we obtained an increase in TAG levels in TG mice compared to WT, while another study carried out in a different mouse male model showed a decrease^50^. On the other hand, and as corroborated by literature, levels of LPLs and PS increased^25,31,60,61^, and CL decreased^62^. Moreover, most of the PLs (PC, PE, PG) showed decreasing levels in AD in contrast to other studies^28,50,61^, possibly due to the use of different mouse models, sex or disease stages.

Finally, the CX is a brain area well studied as one of the structures most affected in the onset and progression of the disease with significant neurodegeneration in both cortical and subcortical regions^63^. In the present work and previous studies, it was observed a decrease in LPLs and some PLs (e.g. PS, PC)^22,27^, and an increase in PG levels^51^ in this area.

Regarding lipidomics studies in other animal models and human brain, some similarities were observed in comparison with the present study. In fact, an increase in Cer levels was observed in AD human brain^57,64,65^ and cerebral cortex of AD rat^66^. In addition, an increase in GLs (DAG and TAG) was observed in AD human brain^57^, as it was found in the HPC of the present work. Furthermore, a decrease in PL levels was observed in AD human frontal cortex and HPC^67^, the same as in HPC and CX in the present AD model.

Lipid alterations may correlate with cognitive impairment. For SPs, a recent study showed that plasma or serum Cer levels may be associated with the severity of AD and brain atrophy^58^; another work showed an association with Cer levels and the development of multiple psychiatric and neurological disorders with cognitive impairment^68^. Specifically, elevated serum Cer levels in patients predicted incident impairment^69^, increased risk of cognitive impairment in women^70^ and correlated with lower scores on the Mini-Mental State examination^71^. Also, elevated cerebrospinal fluid levels were associated with worse performances on certain neuropsychological tests^72^, although a previous work showed that ageing is associated with changes in the hippocampal sphingolipid profile regardless of sex^73^. For GLs, the present work showed decrease brain levels, specifically a previous study observed that plasma TAG levels progressively decreased from participants with normal cognition to MCI and AD, suggesting that lower TAG levels were associated with greater severity of cognitive impairment^74^. For GPs, lower levels showed association with deficits in the Morris Water Maze in mouse models, as well as with poorer memory performance and lower brain function during ageing^75^.

As expected, according to the multivariant analysis, many of the selected lipid variables coincide with the lipid variables that were significant in the univariate analysis. In addition, most of the lipids selected belonged to the GL and GP groups. To our knowledge, there are few previous studies of lipidomics in mouse brains, in which a selection of variables and discriminant analysis have been carried out^16,33,76,77^. In a study performed on whole brain tissue from AD and WT mice, they found that genotypes could be reliably discriminated using models with different lipid families. Specifically, with lipids belonging to the GP and SP families, the model showed R^2^ = 1.000 and Q^2^ = 0.840, and with the GL and ST families, the model showed R^2^ = 0.897 and Q2 = 0.619^16^. Also, in another study performed on the whole brain of AD and WT mice at different ages, they obtained a discriminant model with a fit of 69% and a predictive power of 51%, with the vast majority of lipids belonging to the GP family^33^. In another study on brain and plasma samples from AD and WT mice at different ages, using PCA and OPLS/PLS, the models provided reliable values for the quality parameters R2 and Q2, with an explained variance close to 100% and a predicted variance > 80% for all models. So, it was possible to clearly distinguish between genotypes at all ages from GL and GP lipids^76^. Finally, a recent work evaluated the genotype separation in mouse cortical lipidoma with 60 lipid species (40 lipid species in the negative mode dataset and 20 species in the positive mode dataset, most of them belonging to the GP family), using OPLS-DA and a satisfactory validation parameter was obtained (Q^2^ = 0.738)^77^.

In the present work, some discriminant models have been developed corresponding to the different analysed brain areas. Similarly to previous studies, lipids mainly belonged to the GL and GP families. The models developed for HPC and CX, showed lower diagnosis indexes (sensitivity, specificity, accuracy…), while the models developed for CB and AM showed better diagnostic indexes. It should be noted that the model considering all the studied areas simultaneously showed the best diagnosis indexes.

Regarding limitations, the lipidomic approach was not performed in some brain areas, such as bulb olfactory and brainstem. It was due to the difficulty of extracting these parts homogeneously from all mice, in order to assure the reproducibility of the study. However, the present study focused on cerebellum and amygdala, brain areas poorly studied by now, since its relationship with the disease is not well known and its extraction is difficult, respectively.

## Conclusions

The brain lipidomics study carried out in an AD female model has provided a thorough analysis of brain composition, observing significant differences in early AD. Specifically, an increase of Cer levels in CB, AM and HPC; a decrease of GLs levels in CB, AM and an increase in HPC; a decrease of FA only in AM; a clear increase of PLs and LPLs in CB, while some ambiguity was observed in both subfamilies in the rest of the areas. So, some lipid pathways could be identified in early AD development. The multivariate approach has revealed the satisfactory discrimination capacity of some lipid variables, especially in CB and AM. So, these variables could be considered potential blood biomarkers. Nevertheless, further work in plasma samples from these animal models should be carried out.

### Supplementary Information

Supplementary Figures.

Supplementary Figures.

Supplementary Tables.

Supplementary Tables.

## Data Availability

The datasets used and/or analysed during the current study are available from the corresponding author upon reasonable request.

## Abbreviations

Aβ: Amyloid-β
AD: Alzheimer’s disease
AMG: Amygdala
CB: Cerebellum
Cer: Ceramide
CL: Cardiolipin
CX: Cortex
DAG: Diacylglycerol
FA: Fatty acid
GL: Glycerolipid
GP: Glycerophospholipid
HPC: Hippocampus
IQR: Inter-quartile range
LPC: Lysophosphatidylcholine
LPE: Lysophosphatidylethanolamine
LPI: Lysophosphoinositol
LPL: Lysophospholipid
MAG: Monoacylglycerol
MU: Monounsaturated lipid
PC: Phosphatidylcholine
PCo: Ether-linked phosphatidylcholine
PE: Phosphatidylethanolamine
PEo: Ether-linked phosphatidylethanolamine
PG: Phosphoglyceride
PI: Phosphatidylinositol
PL: Phospholipid
PS: Phosphatidylserine
PU: Polyunsaturated lipid
SAT: Saturated lipid
SM: Sphingomyelin
SP: Sphingolipid
ST: Sterol lipid
TAG: Triacylglycerol
TG: Transgenic mice
WT: Wild type
