# Author Correction: Brain areas lipidomics in female transgenic mouse model of Alzheimer's disease

**DOI:** 10.1038/s41598-024-52528-z

**Published:** 2024-01-24

**Authors:** Laura Ferré-González, Ángel Balaguer, Marta Roca, Artemis Ftara, Ana Lloret, Consuelo Cháfer-Pericás

**Affiliations:** 1 Alzheimer’s Disease Research Group, Health Research Institute La Fe, Avda de Fernando Abril Martorell, 106, 46026 Valencia, Spain; 2 Faculty of Mathematics, University of Valencia, Valencia, Spain; 3 Analytical Unit, Health Research Institute La Fe, Valencia, Spain; 4 University of Valencia, Valencia, Spain; 5 Department of Physiology, Faculty of Medicine, University of Valencia, Health Research Institute INCLIVA, Valencia, Spain

Correction to: *Scientific Reports* 10.1038/s41598-024-51463-3, published online 09 January 2024

In the original version of this Article, the Acknowledgements and Funding section contained errors.

The Acknowledgements section

“This study has been funded by Instituto de Salud Carlos III through the projects "PI19/00570" and "PI22/00594" (Co-funded by European Regional Development Fund, "A way to make Europe") and by the Grant PID2021-127236OB-100 funded by MCIN/AEI/ 10.13039/501100011033 and by “ERDF A way of making Europe” (AL). CCP acknowledges CPII21/00006. The authors acknowledge the Analytical Unit in Health Research Institute, for their support in sample treatment and analysis.”

now reads,

"This study has been funded by Instituto de Salud Carlos III (ISCIII) through the project "PI22/00594" and co-funded by the European Union (CCP); and by the Grant PID2021-127236OB-100 funded by MCIN/AEI/ 10.13039/501100011033 and by “ERDF A way of making Europe” (AL). CCP acknowledges CPII21/00006. The authors acknowledge the Analytical Unit in Health Research Institute, for their support in sample treatment and analysis.”

The Funding section.

“This work was supported by the Instituto de Salud Carlos III through Projects PI19/00570 and PI22/00594 (Spanish Ministry of Economy and Competitiveness and co-funded by European Union, ERDF “A way to make Europe”) (CCP), and by the Grant PID2021-127236OB-100 funded by MCIN/AEI/ 10.13039/501100011033 and by “ERDF A way of making Europe” (AL). CCP acknowledges CPII21/00006. Part of the analytical equipment used in this work was co-funded by the Generalitat Valenciana and European Regional Development Fund (FEDER) funds (PO FEDER of Comunitat Valenciana 2014–2020).”

now reads,

"This work was supported by Instituto de Salud Carlos III (ISCIII) through the project "PI22/00594” and co-funded by the European Union (CCP); and by the Grant PID2021-127236OB-100 funded by MCIN/AEI/ 10.13039/501100011033 and by “ERDF A way of making Europe” (AL). CCP acknowledges CPII21/00006. Part of the analytical equipment used in this work was co-funded by the Generalitat Valenciana and European Regional Development Fund (FEDER) funds (PO FEDER of Comunitat Valenciana 2014‐2020)."

The original Article has been corrected.

