# Supplementary material for: Brain areas lipidomics in female transgenic mouse model of Alzheimer's disease
Source: Sci Rep. 2024 Jan 9;14:870. doi: 10.1038/s41598-024-51463-3 (PMC10776612; doi:10.1038/s41598-024-51463-3)
Supplement: Supplementary file 1 — Supplementary Figures. [file 41598_2024_51463_MOESM1_ESM.docx]

**Positive ionisation mode**


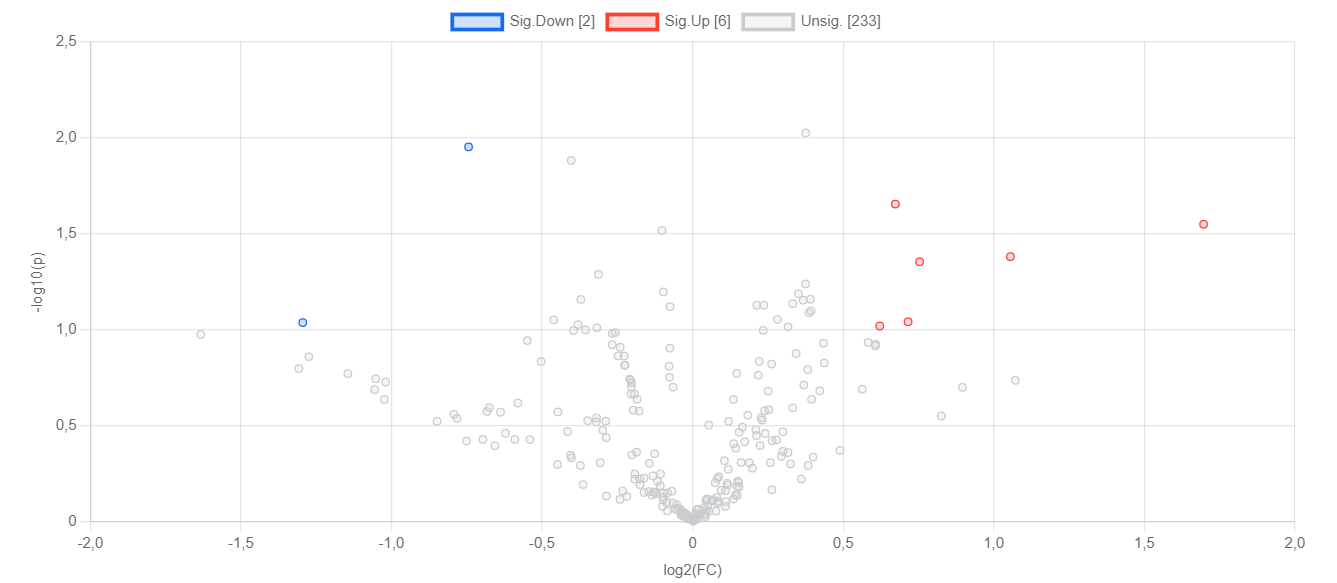

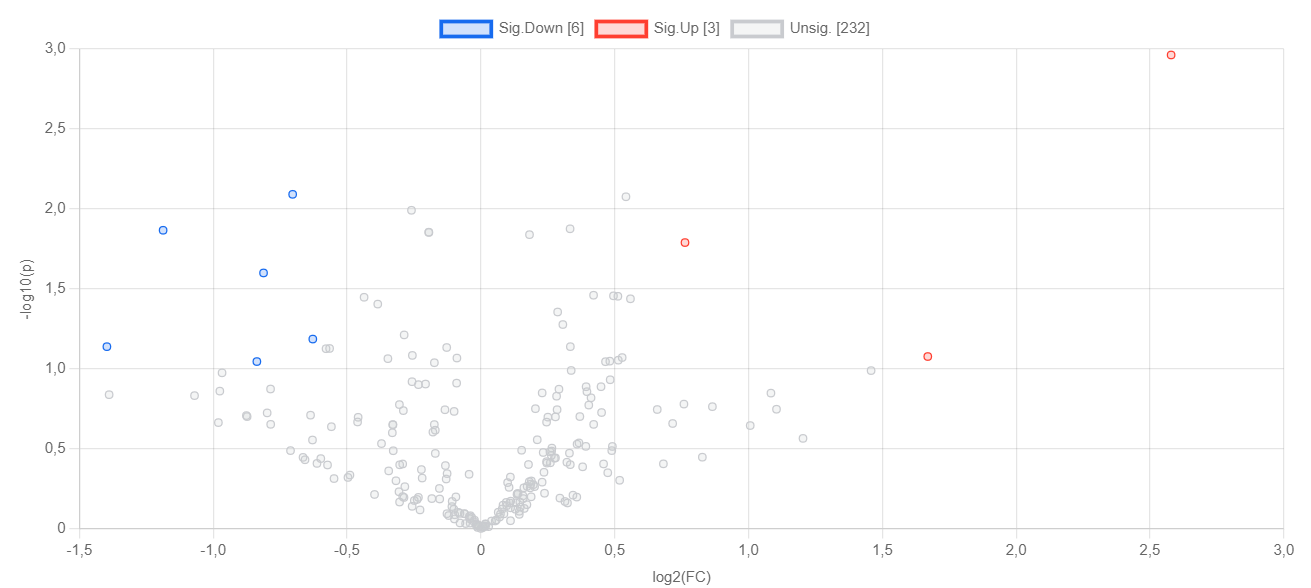
CB) AM)


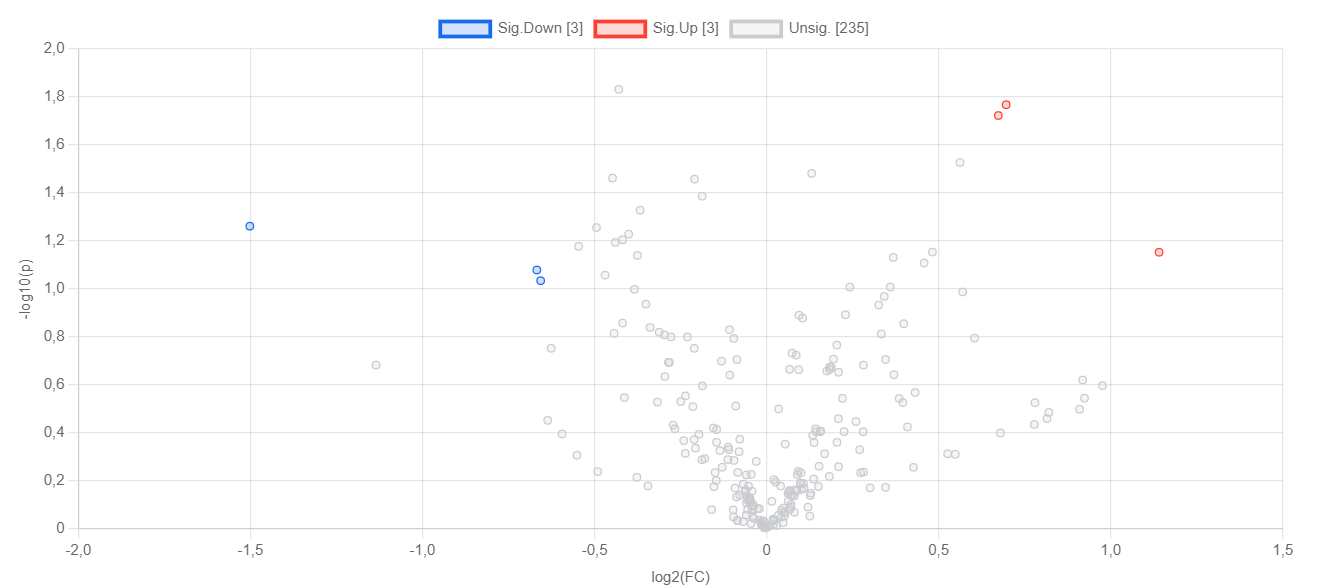

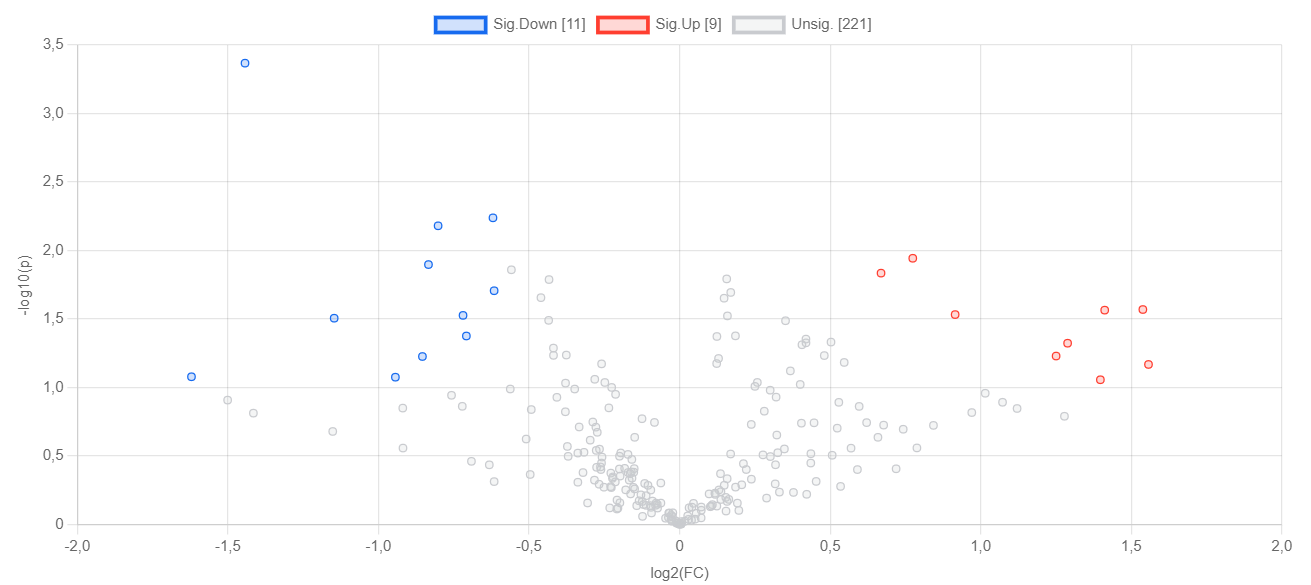
HPC) CX)

**Figure. S1**. Volcano plots of lipidomics showing differential substances between TG and WT in the positive ionisation mode in cerebellum (CB), amygdala (AM), hippocampus (HPC) and cortex (CX). Each dot represents one compound. The x-axis represents log2 (Fold change), and the y-axis represents −log10 (P-value). Red points represent lipid variables that show significantly higher levels in TG, and blue points represent lipid variables that are differed significantly lower levels in TG.

**Negative ionisation mode**


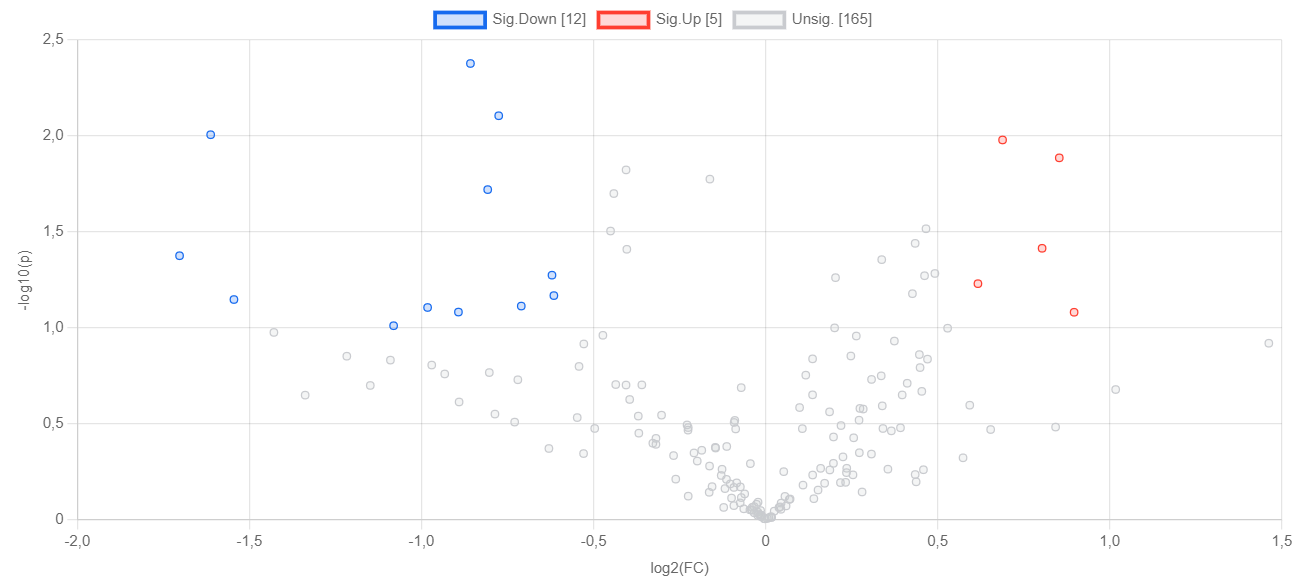
**
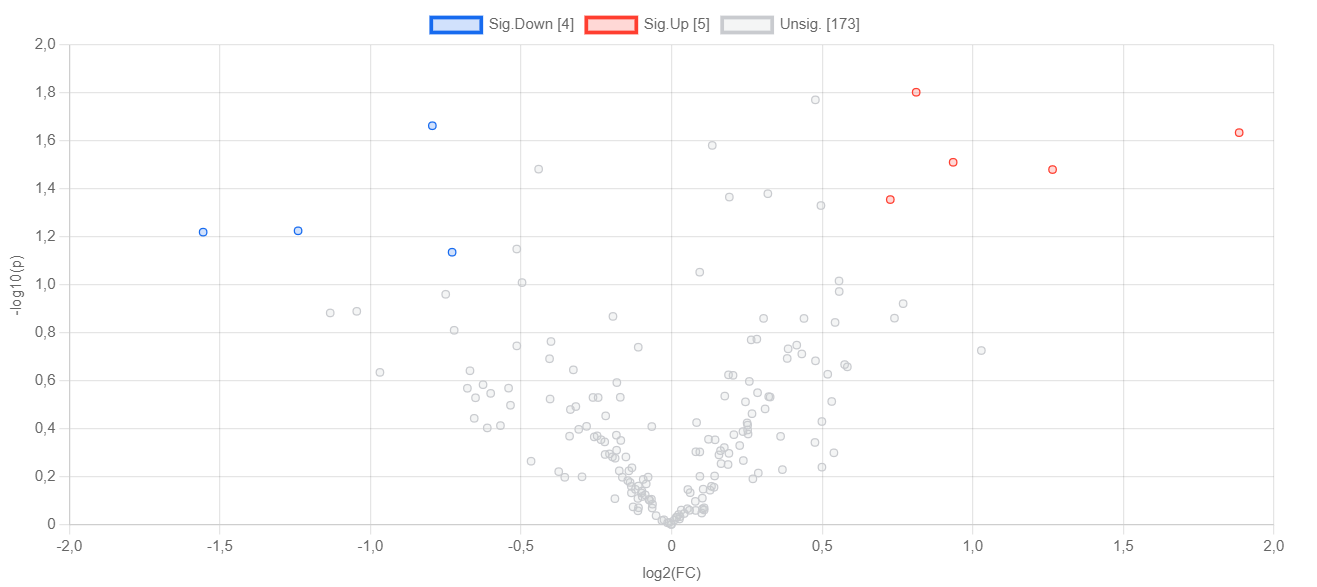
**CB) AM)


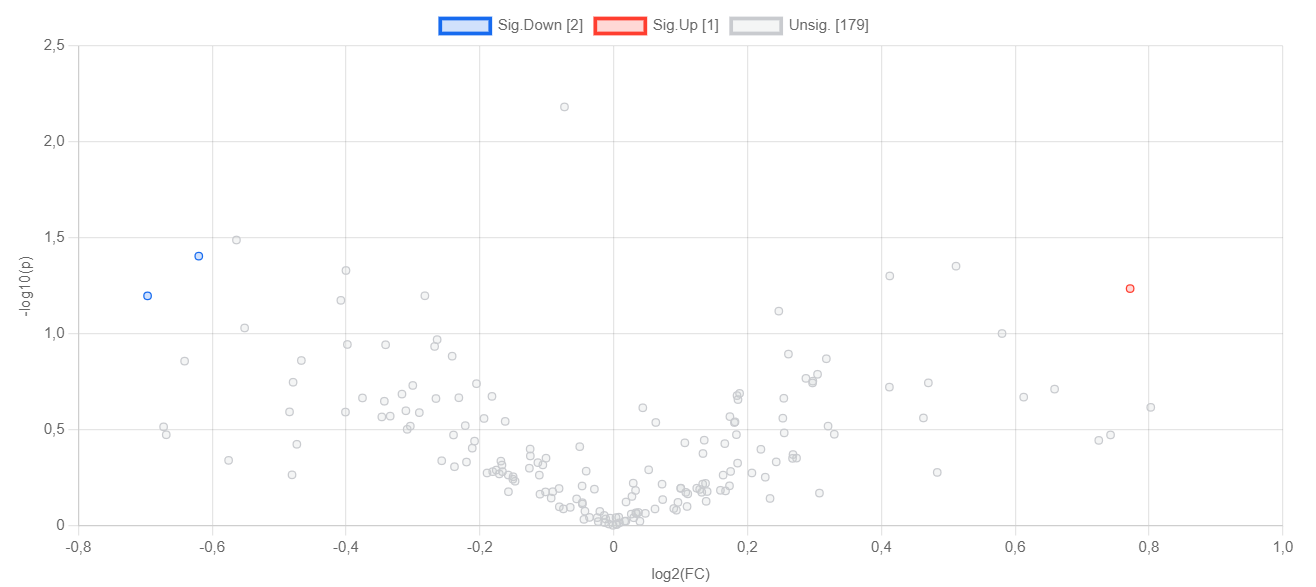

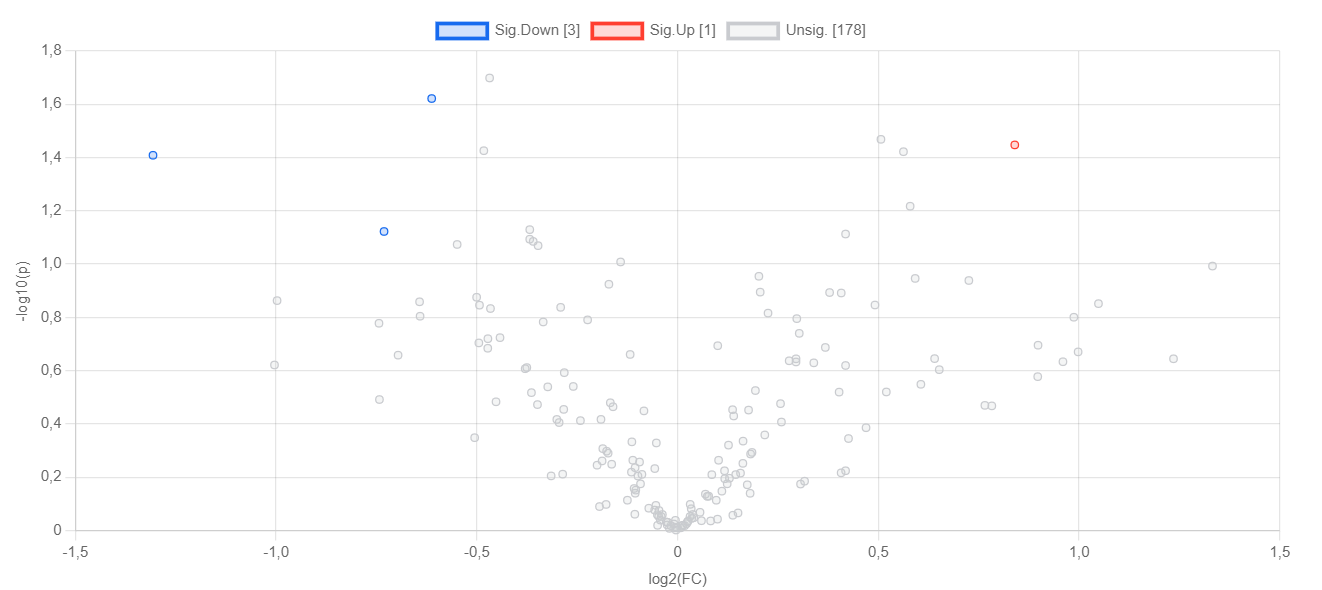
HPC) CX)

**Figure. S2**. Volcano plots of lipidomics showing differential substances between TG and WT in the negative ionisation mode in cerebellum (CB), amygdala (AM), hippocampus (HPC) and cortex (CX). Each dot represents one compound. The x-axis represents log2 (Fold change), and the y-axis represents −log10 (P-value). Red points represent lipid variables that show significantly higher levels in TG, and blue points represent lipid variables that are differed significantly lower levels in TG.
