# Supplementary material for: Brain areas lipidomics in female transgenic mouse model of Alzheimer's disease
Source: Sci Rep. 2024 Jan 9;14:870. doi: 10.1038/s41598-024-51463-3 (PMC10776612; doi:10.1038/s41598-024-51463-3)
Supplement: Supplementary file 2 — Supplementary Figures. [file 41598_2024_51463_MOESM2_ESM.docx]

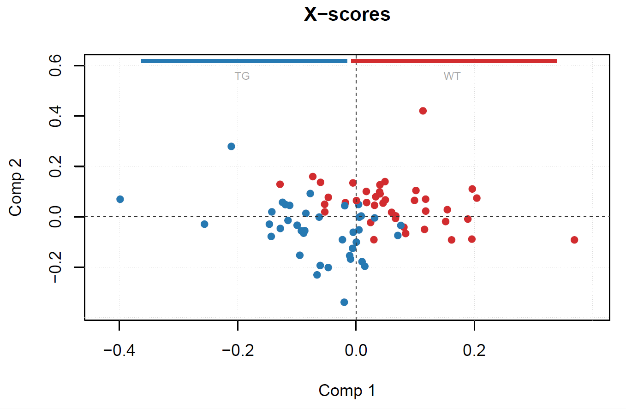

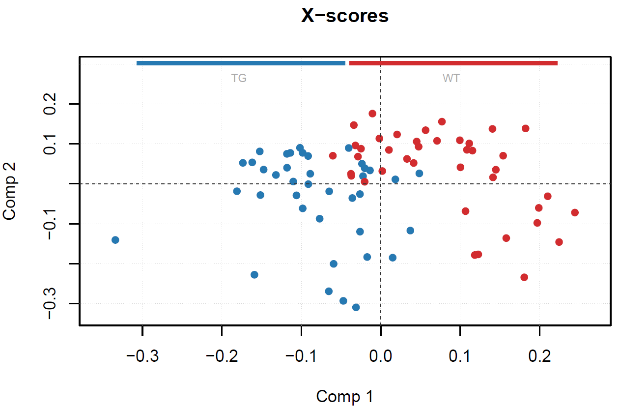
 **Positive ionisation mode Negative ionisation mode**

**Figure S3**. PLS-DA plots of lipidomics in the positive ionisation mode (left) and negative ionisation mode (right) in all brain areas. Each dot represents one mouse (blue: TG, red: WT).

**Positive ionisation mode Negative ionisation mode**


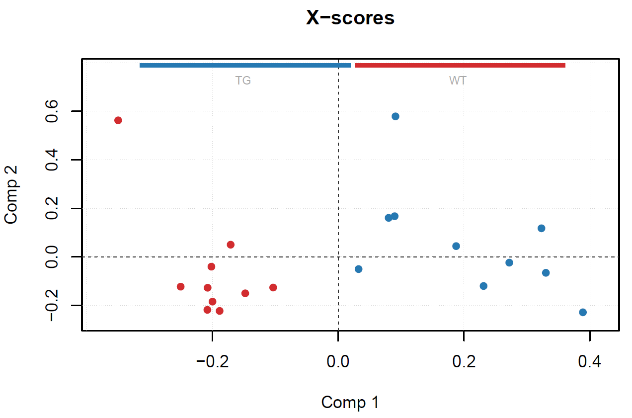

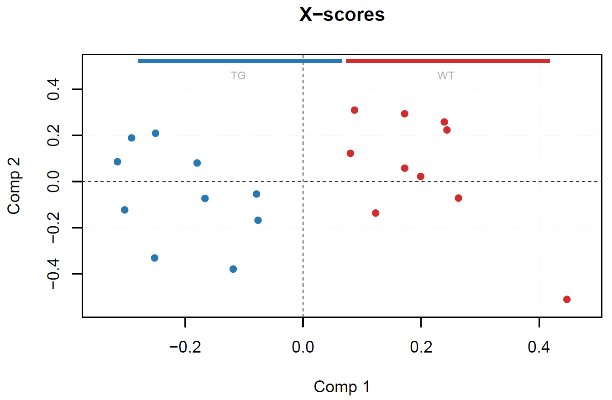


**Figure S4**. PLS-DA plots of lipidomics in the positive ionisation mode (left) and negative ionisation mode (right) in the cerebellum (CB). Each dot represents one mouse (blue: TG, red: WT).

**Positive ionisation mode Negative ionisation mode**


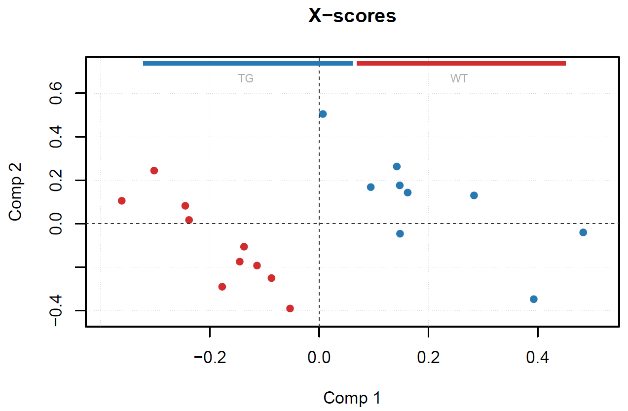

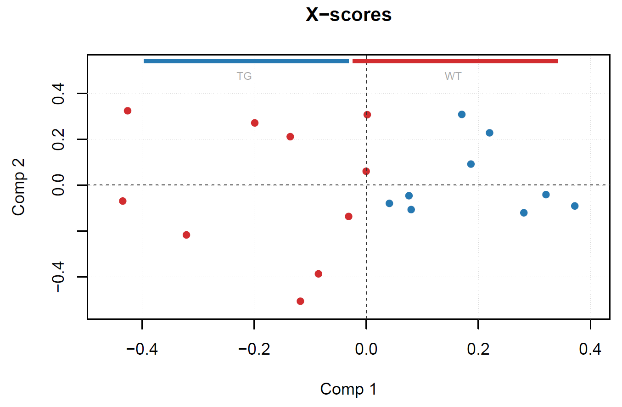


**Figure S5**. PLS-DA plots of lipidomics in the positive ionisation mode (left) and negative ionisation mode (right) in the amygdala (AM). Each dot represents one mouse (blue: TG, red: WT).

**Positive ionisation mode Negative ionisation mode**


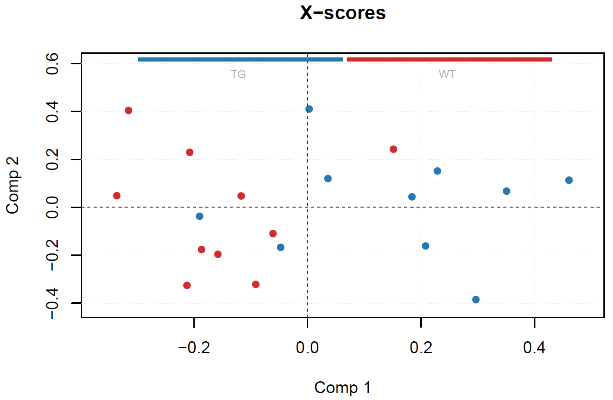

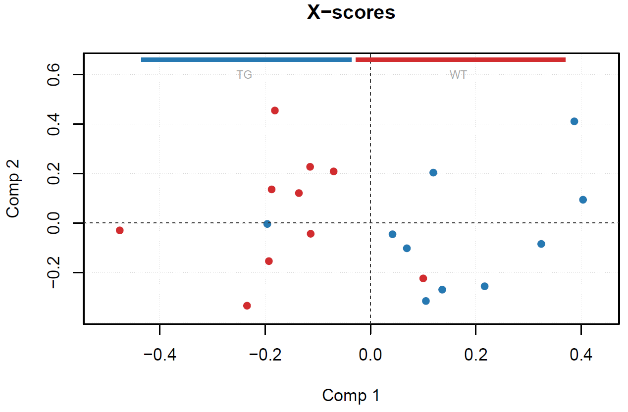


**Figure S6.** PLS-DA plots of lipidomics in the positive ionisation mode (left) and negative ionisation mode (right) the hippocampus (HPC). Each dot represents one mouse (blue: TG, red: WT).


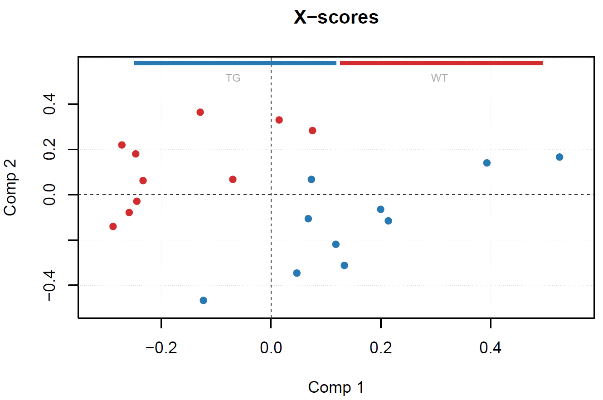
 **Positive ionisation mode Negative ionisation mode**


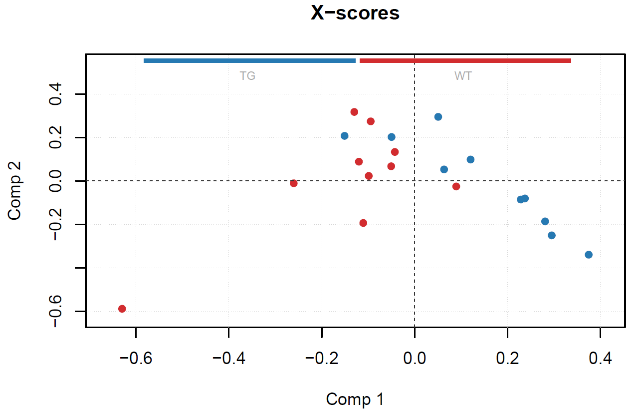


**Figure S7.** PLS-DA plots of lipidomics in the positive ionisation mode (left) and negative ionisation mode (right) in the cortex (CX). Each dot represents one mouse (blue: TG, red: WT).
