# Supplementary material for: Brain areas lipidomics in female transgenic mouse model of Alzheimer's disease
Source: Sci Rep. 2024 Jan 9;14:870. doi: 10.1038/s41598-024-51463-3 (PMC10776612; doi:10.1038/s41598-024-51463-3)
Supplement: Supplementary file 3 — Supplementary Tables. [file 41598_2024_51463_MOESM3_ESM.docx]

**Table S1.** Lipid variables by mouse genotype with median, interquartile and p-value in positive ionization mode in the cerebellum.

| **Lipid variables** | **WT** | | | **TG** | | | **p value (Mann-Whitney)** |
| --- | --- | --- | --- | --- | --- | --- | --- |
|  | **Median** | **Quartile 1** | **Quartile 3** | **Median** | **Quartile 1** | **Quartile 3** |  |
| CE(20:0)_M+NH4 | 5473562,2 | 4782644,4 | 6596183,0 | 5897918,2 | 5644667,3 | 6604255,1 | 0,247 |
| Cer(d16:1/17:0)_M+H-H2O | 8957187,8 | 7195385,8 | 9689819,6 | 10613716,6 | 9916720,8 | 12958517,5 | <0,001 |
| Cer(d16:1/17:0)_M+H | 7649732,1 | 6261567,1 | 8422044,3 | 9525155,3 | 8795311,1 | 11274472,4 | <0,001 |
| Cer(d18:1/17:0)_M+Na | 423658342,3 | 380299429,6 | 461746978,8 | 447118783,3 | 426656694,0 | 591305767,7 | 0,165 |
| Cer(d18:1/18:0)_M+H-H2O | 47819579,0 | 24628430,0 | 51441151,6 | 42571775,0 | 40676107,7 | 55578895,0 | 0,853 |
| Cer(d18:1/18:0)_M+H | 59172664,0 | 30890035,0 | 64577344,1 | 54050775,5 | 50942497,4 | 70947224,3 | 0,796 |
| Cer(d18:1/18:0)_M+Na | 41159445,6 | 17301669,9 | 44951402,4 | 33833129,7 | 30948799,4 | 41227771,1 | 0,971 |
| Cer(d18:1/18:1)_M+H-H2O | 1982735,7 | 1163406,0 | 2511931,8 | 2791467,6 | 2202201,5 | 3187719,2 | 0,063 |
| Cer(d18:1/18:1)_M+H | 2754130,9 | 1700258,7 | 3270380,0 | 3786355,5 | 2903331,8 | 4302947,9 | 0,105 |
| Cer(d18:1/18:1)_M+Na | 1114995,1 | 587700,0 | 1364789,1 | 1389937,1 | 1196799,4 | 1641920,1 | 0,105 |
| Cer(d18:1/22:0)_M+H | 3903596,7 | 3120781,6 | 4228473,5 | 4281470,5 | 3954050,5 | 4876487,5 | 0,123 |
| Cer(d18:1/24:1)_M+H-H2O | 7040571,0 | 5346642,2 | 7704274,6 | 8200254,8 | 7469052,3 | 9698717,4 | 0,019 |
| Cer(d18:1/24:1)_M+H | 25454876,6 | 20699863,5 | 26725166,9 | 30933262,6 | 26749132,1 | 35554681,0 | 0,011 |
| Total Cer | 590591939,9 | 519255173,2 | 655009860,7 | 635456449,0 | 598339832,3 | 756066460,7 | 0,063 |
| DAG(16:0/16:0)_M+H-H2O | 11192917,8 | 10596162,2 | 11452066,7 | 10309435,6 | 9946111,4 | 11018928,8 | 0,019 |
| DAG(16:0/18:1)_M+H-H2O | 38035757,9 | 36118402,5 | 39696918,2 | 33471928,6 | 25985458,0 | 35806881,0 | 0,019 |
| DAG(16:0/18:1)_M+NH4 | 56734726,5 | 43323554,1 | 68492393,2 | 57281061,0 | 42509815,8 | 65013740,9 | 1,000 |
| DAG(16:0/18:2)_M+H-H2O | 11301877,5 | 9955117,7 | 12602584,5 | 9516891,1 | 8381599,0 | 9904308,2 | 0,023 |
| DAG(16:0/20:4)_M+NH4 | 6997668,2 | 6326607,4 | 8930914,7 | 6448609,4 | 5301075,2 | 8078804,9 | 0,481 |
| DAG(16:0/20:4)_M+Na | 5783112,3 | 4973942,2 | 6758195,1 | 5709410,1 | 3994659,3 | 7186555,8 | 0,912 |
| DAG(16:0/22:1)_M+NH4 | 14759464,7 | 11912872,8 | 15970164,9 | 14684221,2 | 10846727,4 | 18552508,2 | 0,579 |
| DAG(16:0/22:6)_M+H-H2O | 30233409,2 | 23204452,4 | 35848612,0 | 32630395,4 | 27385965,2 | 40733912,3 | 0,315 |
| DAG(18:0/18:0)_M+H-H2O | 2796797,1 | 2496388,5 | 3010650,5 | 3126802,3 | 2835596,2 | 3368843,9 | 0,029 |
| DAG(18:0/18:0)_M+NH4 | 7009510,5 | 6321157,5 | 7193668,3 | 7433661,0 | 6953938,1 | 8105094,0 | 0,075 |
| DAG(18:0/18:0)_M+Na | 11057878,6 | 9994437,7 | 11576250,4 | 11654806,0 | 11309236,4 | 12706281,1 | 0,023 |
| DAG(18:0/20:4)_M+H-H2O | 44437256,4 | 33371012,6 | 50942008,4 | 53506694,0 | 41403475,8 | 62303452,8 | 0,063 |
| DAG(18:0/20:4)_M+NH4 | 333799805,7 | 292245562,9 | 378353306,6 | 316355300,0 | 256714196,8 | 417516210,1 | 1,000 |
| DAG(18:0/20:4)_M+Na | 157072726,8 | 141117537,1 | 167027965,7 | 146942115,6 | 126875867,4 | 180210941,1 | 0,912 |
| DAG(18:0/22:4)_M+NH4 | 24295882,3 | 20455778,2 | 27233200,6 | 24441287,8 | 19736454,3 | 31823240,2 | 0,853 |
| DAG(18:0/22:4)_M+Na | 13429539,8 | 11402056,4 | 15274675,6 | 14177655,8 | 10873937,5 | 18478005,4 | 0,739 |
| DAG(18:0/22:6)_M+H-H2O | 150669381,7 | 101164633,6 | 179202019,5 | 171236695,9 | 140306970,8 | 209211382,0 | 0,218 |
| DAG(18:1/16:0)_M+H-H2O | 44698028,1 | 43378737,4 | 47162009,2 | 39831358,1 | 31676936,4 | 43482388,2 | 0,007 |
| DAG(18:1/18:1)_M+H-H2O | 108214129,4 | 99318106,8 | 111468738,2 | 102550850,0 | 87471653,8 | 109605905,1 | 0,353 |
| DAG(18:1/18:1)_M+NH4 | 112025029,4 | 98131752,8 | 118940627,7 | 101315269,1 | 91632259,2 | 115552019,2 | 0,280 |
| DAG(18:1/18:1)_M+Na | 56299366,0 | 50033251,7 | 58938623,8 | 49370836,1 | 46437284,0 | 57123258,7 | 0,105 |
| DAG(18:1/18:2)_M+H-H2O | 15870131,5 | 14812050,9 | 16858293,2 | 14344240,3 | 12015328,8 | 15066471,8 | 0,015 |
| DAG(18:2/18:2)_M+H-H2O | 1686687,5 | 1570631,5 | 1936200,4 | 1562951,8 | 1493137,7 | 1736006,6 | 0,280 |
| Total DAG | 1254351771,5 | 1159912664,2 | 1332987882,0 | 1240199623,0 | 1097915370,0 | 1350349593,3 | 1,000 |
| LPC(16:0)_M+H | 145459357,2 | 125876325,4 | 163369393,2 | 169715829,0 | 159823349,4 | 180846860,1 | 0,043 |
| LPC(18:0)_M+H | 61857427,1 | 53124007,2 | 66508038,6 | 76866101,4 | 69816239,3 | 85951907,2 | <0,001 |
| LPC(18:1)_M+H | 69047195,5 | 54972384,1 | 133662556,9 | 77675920,4 | 66899791,6 | 83632191,3 | 0,393 |
| LPC(20:4)_M+H | 16981353,8 | 13392227,4 | 30234443,2 | 17961254,2 | 16747753,2 | 20473177,3 | 0,579 |
| LPC(22:6)_M+H | 38923290,3 | 31559500,4 | 75570076,8 | 39507112,5 | 34135563,3 | 45037728,5 | 1,000 |
| Total LPC | 336550572,2 | 285056545,2 | 455644345,4 | 381970941,6 | 351406763,7 | 399462556,1 | 0,218 |
| LPE(22:6)_M+H | 12695313,2 | 10576002,8 | 23070905,9 | 15206929,2 | 13267587,4 | 16680598,5 | 0,353 |
| MAG(18:2)_M+NH4 | 4574470,8 | 3678520,0 | 7629596,4 | 4453766,0 | 3299441,9 | 5839437,1 | 0,796 |
| MAG(20:1)_M+NH4 | 4983885,1 | 2801604,0 | 9676916,4 | 4667365,2 | 2448716,2 | 6781782,9 | 0,796 |
| MAG(20:2)_M+NH4 | 14207775,3 | 11402840,6 | 21139765,1 | 14580200,0 | 13181516,8 | 17852767,2 | 0,684 |
| MAG(20:4)_M+H-H2O | 4676542,7 | 3948917,3 | 5477633,2 | 4521893,4 | 3638269,1 | 6013119,7 | 1,000 |
| MAG(22:2)_M+NH4 | 15271392,2 | 13625434,9 | 16045195,4 | 16860053,5 | 13865036,5 | 19285380,2 | 0,190 |
| MAG(22:3)_ M+NH4 | 8169205,6 | 6586930,4 | 18175876,9 | 8467503,3 | 6461805,1 | 9695107,8 | 0,912 |
| MAG(22:4)_M+NH4 | 8202386,8 | 6597399,6 | 18247925,4 | 8447005,1 | 6508098,9 | 9707947,9 | 0,971 |
| Total MAG | 58906871,7 | 53300296,7 | 92104701,6 | 57548531,3 | 52979932,6 | 70609011,8 | 0,684 |
| PC(14:0_16:0)_M+H | 87438228,7 | 58732552,7 | 96345302,3 | 86044970,0 | 77334714,3 | 121735362,8 | 0,529 |
| PC(16:0/16:0)_M+H | 5541242447,0 | 3749157113,0 | 7751778566,5 | 5578043386,5 | 4546777203,3 | 7980702063,5 | 0,796 |
| PC(16:0/16:0)_M+Na | 158456659,9 | 99814734,8 | 238725641,0 | 170787745,2 | 128915279,0 | 220446635,9 | 0,912 |
| PC(16:0_18:1)_M+H | 17160414479,0 | 11906858673,8 | 19972569047,5 | 20046890038,0 | 15637975040,3 | 23976201264,0 | 0,105 |
| PC(16:0_18:1)_M+Na | 509055928,5 | 359695726,4 | 590154356,1 | 580859486,4 | 481333001,6 | 638203631,4 | 0,190 |
| PC(16:0_20:4)_M+H | 1123359322,0 | 860163530,9 | 1323049785,5 | 1215381458,5 | 1021289297,3 | 1607253754,8 | 0,393 |
| PC(16:0_20:4)_M+Na | 18051722,1 | 13272432,5 | 23955708,8 | 20013890,9 | 14291488,2 | 24462199,4 | 0,631 |
| PC(16:0_22:4)_M+H | 128032699,8 | 103819033,7 | 139333209,2 | 169672555,1 | 128750536,7 | 182871768,7 | 0,023 |
| PC(16:0_22:6)_M+H | 4270538523,0 | 3363652144,5 | 5201803015,0 | 4165106429,0 | 3675409478,0 | 5568451970,0 | 0,796 |
| PC(16:0_22:6)_M+Na | 43531691,0 | 34221728,8 | 59579031,2 | 37570739,1 | 34722727,8 | 49927494,4 | 0,912 |
| PC(16:1/22:6)_M+H | 9687139,5 | 7215875,9 | 18086522,3 | 8472848,0 | 7290735,8 | 13756221,9 | 0,684 |
| PC(17:0/18:1)_M+Na | 15672900,6 | 12559499,9 | 17590136,8 | 21132419,2 | 17027835,5 | 27310422,3 | 0,011 |
| PC(18:0/22:4)_M+H | 121215761,3 | 93632792,9 | 139783041,9 | 156403409,7 | 117606255,3 | 183717170,9 | 0,075 |
| PC(18:0/22:5)_M+H | 39840090,3 | 27687162,6 | 49978213,4 | 42312833,1 | 39669372,0 | 61164453,9 | 0,280 |
| PC(18:0_18:1)_M+H | 4879673697,5 | 3586976787,0 | 5428686636,8 | 5697652000,0 | 4588280063,8 | 6999325229,5 | 0,105 |
| PC(18:0_18:1)_M+Na | 87277834,6 | 58086545,8 | 108350087,2 | 103560713,2 | 80306674,3 | 129579761,8 | 0,315 |
| PC(18:0_20:3)_M+H | 79138410,4 | 55341813,5 | 98656135,3 | 85640021,5 | 70320097,6 | 113753816,7 | 0,436 |
| PC(18:0_20:4)_M+H | 1903399532,5 | 1377947922,0 | 2302496792,3 | 2207781486,5 | 1697313013,3 | 2664134903,3 | 0,190 |
| PC(18:0_20:4)_M+Na | 52188992,8 | 34318390,8 | 64970817,3 | 58543052,9 | 45774498,7 | 72902402,1 | 0,280 |
| PC(18:0_22:6)_M+H | 3182788984,0 | 2665492267,5 | 4140068511,5 | 3066157474,5 | 2831531117,5 | 4558762487,5 | 0,912 |
| PC(18:0_22:6)_M+Na | 84901676,7 | 67184494,0 | 117209183,7 | 87738445,1 | 69866261,8 | 115232471,7 | 0,853 |
| PC(18:1/16:1)_M+Na | 12706607,8 | 10466432,4 | 15912084,3 | 14414943,0 | 13171952,8 | 18738391,3 | 0,143 |
| PC(18:1/16:1)_M+H | 375471861,7 | 307006982,7 | 452805850,5 | 443986973,6 | 380035343,4 | 606991963,9 | 0,123 |
| PC(18:1/18:1)_M+H | 1591203605,0 | 1088215591,5 | 1700121185,0 | 1833078702,5 | 1374308026,0 | 2103204828,0 | 0,063 |
| PC(18:1/20:4)_M+H | 392369744,8 | 334380235,2 | 464740549,4 | 444163690,5 | 375991334,5 | 550462477,3 | 0,315 |
| PC(18:1/20:4)_M+Na | 12403081,9 | 9869620,8 | 15064250,3 | 13613197,7 | 11983317,7 | 15395927,8 | 0,436 |
| PC(18:2/20:4)_M+H | 8028217,5 | 5866620,1 | 11029244,9 | 8865503,8 | 6502911,7 | 10781731,8 | 0,579 |
| PC(20:1/20:4)_M+H | 62739846,7 | 48453843,8 | 72503663,5 | 79709578,6 | 59670048,6 | 84755241,7 | 0,063 |
| PC(20:1_22:6)_M+H | 97240123,7 | 70356048,8 | 126613791,6 | 110500076,4 | 86266371,2 | 135355887,9 | 0,436 |
| PC(20:4/20:4)_M+H | 12310153,7 | 9000865,7 | 22224481,3 | 12484257,8 | 9154368,7 | 17907738,5 | 0,912 |
| PC(20:4_22:6)_M+H | 16277735,5 | 13420086,0 | 45972192,6 | 16177127,8 | 12890464,7 | 25160212,4 | 0,912 |
| PC(22:6/22:6)_M+H | 46523329,7 | 41572707,3 | 184303446,8 | 48515428,0 | 40040471,0 | 67437124,6 | 0,971 |
| PC(31:0)_M+H | 19674417,3 | 15593436,0 | 25573397,1 | 20913041,8 | 18662845,6 | 26826568,4 | 0,631 |
| PC(33:2)_M+H | 367520178,3 | 281765879,4 | 408726315,1 | 435185423,2 | 354388710,1 | 492430690,2 | 0,043 |
| PC(33:3)_M+H | 12137287,0 | 9572935,3 | 14119373,9 | 15730449,6 | 12641120,1 | 18965108,4 | 0,052 |
| PC(35:4)_M+Na | 232928052,8 | 191428093,9 | 273315449,7 | 252513247,2 | 218625714,7 | 311283868,4 | 0,280 |
| PC(36:4)_M+H | 3732513,0 | 2560337,7 | 4341888,6 | 4543977,7 | 3710966,0 | 5168905,6 | 0,105 |
| PC(36:5)_M+H | 4189403,2 | 3200899,6 | 6852805,4 | 4557681,3 | 3702761,1 | 5891492,8 | 0,684 |
| PC(38:5)_M+H | 18281621,2 | 15662198,0 | 25009796,5 | 20407988,5 | 19804820,3 | 32348860,8 | 0,143 |
| PC(40:7)_M+H | 437105847,2 | 341443047,3 | 542166634,7 | 399217488,4 | 357479472,2 | 541629945,1 | 0,912 |
| PC(40:7)_M+Na | 6087448,6 | 4280443,0 | 7850886,7 | 4892096,6 | 4671143,4 | 6158461,5 | 0,853 |
| Total PC | 43620729567,0 | 32943674970,8 | 51444001786,5 | 47521907691,0 | 38125509912,0 | 60107732632,8 | 0,218 |
| PCo(16:0/16:0)_M+H | 16940670,2 | 13430807,6 | 21976515,2 | 19501322,9 | 15244727,0 | 23999180,2 | 0,579 |
| PCo(18:1/16:0)\|PCp(16:0_18:0)_M+H | 116745421,4 | 86624713,8 | 125913266,2 | 136924734,3 | 107916440,5 | 157812645,3 | 0,089 |
| PCo(35:5)\|PCp(35:4)_M+Na | 217172498,3 | 181062600,0 | 230183780,6 | 263315543,0 | 217040822,6 | 278311037,8 | 0,043 |
| PCo(36:7)\|PCp(36:6)_M+H | 55378440,5 | 46708979,0 | 65011439,5 | 75856326,2 | 62383115,9 | 83399819,4 | 0,019 |
| PCo(37:6)\|PCp(37:5)_M+Na | 88196642,9 | 84271914,6 | 92515946,1 | 98933744,2 | 93400791,6 | 109300245,2 | 0,002 |
| PCo(37:6)\|PCp(37:5)_M+H | 12709350,1 | 10744782,1 | 14094013,0 | 15193673,3 | 13808828,3 | 16351805,7 | 0,063 |
| PCo(38:5)\|PCp(38:4)_M+H | 9799985,3 | 7822238,2 | 11445564,9 | 11461265,8 | 8143488,1 | 13329750,7 | 0,190 |
| PCo(38:7)\|PCp(38:6)_M+H | 3639549,6 | 889047,8 | 5606130,2 | 4544578,4 | 1966370,4 | 7615711,1 | 0,280 |
| PCp(16:0/16:0)\|PCo(16:0_16:1)_M+H | 5654361,9 | 3484319,7 | 6778226,2 | 6970217,2 | 4475053,6 | 8449928,4 | 0,165 |
| PCp(34:6)_M+H | 26615330,5 | 10115647,7 | 39337663,2 | 27425006,6 | 16059149,4 | 46278338,4 | 0,684 |
| PCp(35:5)_M+Na | 3773017,8 | 2662167,3 | 4469672,6 | 4058901,2 | 3518479,1 | 5629446,3 | 0,353 |
| PCp(35:5)\|PEo(38:6)_M+Na | 41387556,3 | 26793926,0 | 58125947,9 | 45184541,2 | 33492544,9 | 53516971,6 | 0,796 |
| PCp(35:5)\|PCo(33:3)_M+H | 299920268,8 | 247100964,6 | 362340482,2 | 379609851,3 | 344425071,4 | 463074930,4 | 0,011 |
| PCp(37:6)_M+Na | 103172632,7 | 65644755,3 | 124222639,0 | 121330331,6 | 94086620,8 | 135762528,2 | 0,315 |
| PCp(38:5)\|\|PCo(38:6)_M+H | 13634681,8 | 11927830,7 | 15482223,5 | 13229245,1 | 12325393,0 | 18110816,5 | 0,579 |
| PCp(39:6)_M+Na | 11295901,0 | 6518917,6 | 14475704,7 | 13271384,3 | 9952228,9 | 19260044,3 | 0,481 |
| Total PCo | 1090635368,0 | 778841611,4 | 1126688755,8 | 1265368264,0 | 1014701863,4 | 1400522781,5 | 0,023 |
| PE(18:0_22:4)_M+H | 113400122,3 | 90276073,1 | 131805875,5 | 147573396,5 | 116870667,2 | 170623750,7 | 0,015 |
| PE(22:6/16:0)_M+H | 567254061,4 | 442486720,5 | 666129439,0 | 602395461,9 | 518921323,7 | 759610817,4 | 0,353 |
| PE(22:6/16:0)_M+Na | 17401233,7 | 14911748,0 | 20055665,7 | 18738706,9 | 16279800,3 | 19689623,4 | 0,579 |
| PE(22:6/18:0)_M+Na | 53477203,0 | 47503423,3 | 56637761,2 | 54250037,9 | 52509902,4 | 55103366,2 | 0,796 |
| PE(22:6/22:6)_M+H | 42796712,3 | 33271308,7 | 71991729,0 | 41150655,1 | 35756762,3 | 52822289,2 | 1,000 |
| PE(34:1)_M+H | 178961068,9 | 139922096,8 | 204167965,5 | 220563819,8 | 170179158,8 | 257511135,0 | 0,052 |
| PE(36:1)_M+H | 240011559,1 | 201236260,6 | 271704695,0 | 298471208,6 | 250887472,3 | 318017528,3 | 0,019 |
| PE(36:4)_M+H | 108189306,0 | 84450508,0 | 116042205,3 | 132177480,3 | 102636381,1 | 148545270,3 | 0,019 |
| PE(37:4)_M+H | 5499842,2 | 3879067,9 | 6332843,9 | 6399998,5 | 4924166,0 | 7368705,0 | 0,123 |
| PE(38:3)_M+H | 13425102,2 | 10134085,4 | 15138993,2 | 16676679,1 | 13552154,8 | 19800579,6 | 0,089 |
| PE(38:4)_M+H | 39322075,9 | 29161205,5 | 45848652,6 | 46936814,4 | 38543328,8 | 59945958,8 | 0,063 |
| PE(20:4/18:0)_M+H | 787196946,4 | 591748818,2 | 901225672,5 | 946199235,8 | 741896892,7 | 1093957997,5 | 0,063 |
| PE(38:4)_M+Na | 25741062,4 | 21402463,9 | 29254748,2 | 28571763,0 | 24665640,3 | 31620211,9 | 0,280 |
| PE(39:6)_M+H | 11505263,9 | 8567598,5 | 13295137,1 | 13071410,6 | 10944434,5 | 15051748,6 | 0,190 |
| PE(40:5)_M+H | 28537845,8 | 20689584,8 | 32505609,0 | 33897014,8 | 31443597,6 | 39880052,1 | 0,052 |
| PE(40:6)_M+Na | 54399491,6 | 48264369,7 | 57776896,8 | 55510684,0 | 53764679,3 | 55883875,8 | 0,796 |
| PE(40:6)_M+H | 3041922046,5 | 2078848468,3 | 3623969620,5 | 3477481797,5 | 2838400114,0 | 4245560099,8 | 0,165 |
| PE(42:10)_M+H | 7578461,6 | 5623743,3 | 9671085,0 | 7309960,0 | 5831050,3 | 9126596,5 | 1,000 |
| PE(44:10)_M+H | 41684684,6 | 36232517,0 | 57037404,2 | 53227267,2 | 45746553,5 | 60840369,0 | 0,123 |
| Total PE | 5451310519,5 | 3937157175,8 | 6216949849,0 | 6237264841,0 | 5051378958,5 | 7430926833,5 | 0,105 |
| PEo(16:1_20:4)\|PEp(36:4)_M+H | 79748330,2 | 63311615,9 | 92538324,1 | 99010042,8 | 74948522,7 | 110744634,2 | 0,035 |
| PEo(16:1_22:4)PEp(38:4)_M+H | 78434160,3 | 64819863,8 | 100574612,3 | 108139956,9 | 83029522,3 | 121039205,2 | 0,023 |
| PEo(16:1_22:6)\|PEp(16:0/22:6)_M+Na | 7733185,8 | 6545035,3 | 9286313,4 | 7703929,2 | 6851826,1 | 8805198,1 | 0,853 |
| PEo(16:1_22:6)\|PEp(16:0/22:6)_M+H | 381577078,0 | 276293121,8 | 451503509,3 | 396684050,5 | 355144032,2 | 515638007,1 | 0,353 |
| PEo(18:1_18:2)\|PEp(18:1/18:1)_M+H | 664412374,5 | 518683915,7 | 778990675,6 | 814158209,9 | 663249015,2 | 927805491,4 | 0,043 |
| PEo(18:1_20:4)\|PEp(18:0/20:4)_M+H | 303565749,0 | 237332882,0 | 339622058,3 | 365036515,5 | 291968711,5 | 437788947,1 | 0,052 |
| PEo(18:1_20:4)_M+Na | 11483445,2 | 9559843,3 | 12894777,2 | 13243927,9 | 11503102,9 | 14603404,2 | 0,105 |
| PEo(18:1_22:6)_M+H | 1184394859,0 | 857046457,7 | 1472765458,3 | 1213983753,0 | 1065309432,5 | 1595365868,5 | 0,579 |
| PEo(18:1_22:6)_M+Na | 38838915,4 | 32800424,7 | 44863112,9 | 39936946,0 | 36199077,8 | 42700832,0 | 0,796 |
| PEo(18:2_22:4)\|PEp(18:1/22:4)_M+H | 496942961,4 | 433194476,2 | 616056213,6 | 623331878,7 | 594720879,4 | 802124709,1 | 0,011 |
| PEo(18:2_22:6)_M+Na | 4622896,9 | 3501403,8 | 5382150,1 | 4515904,2 | 4071403,5 | 4844484,2 | 0,684 |
| PEo(34:2)\|PEp(34:1)_M+H | 269950187,0 | 216091967,8 | 304539194,7 | 327555094,0 | 280345554,3 | 360594720,7 | 0,035 |
| PEo(36:4)\|PEp(36:3)_M+H | 7746913,8 | 5749460,0 | 9972892,3 | 11822025,4 | 7949375,2 | 12470590,9 | 0,011 |
| PEo(36:5)\|PEp(36:4)_M+Na | 2682200,1 | 2289102,9 | 3098159,1 | 3228066,9 | 2742926,5 | 3415283,5 | 0,043 |
| PEo(37:5)\|PEp(37:4)_M+H | 6037730,6 | 4819970,2 | 6412980,7 | 7369181,6 | 5734357,6 | 8317443,9 | 0,043 |
| PEo(38:4)\|PEp(38:3)_M+H | 6938349,9 | 5564321,0 | 8432186,0 | 9842474,7 | 7284040,5 | 10598515,1 | 0,015 |
| PEo(38:6)\|PEp(38:5)_M+H | 42588656,1 | 33734065,1 | 54506604,8 | 48675833,2 | 42788126,8 | 55408614,2 | 0,315 |
| PEo(40:6)\|PEp(40:5)_M+H | 54376071,0 | 41594676,6 | 71073601,5 | 58867887,2 | 49807320,0 | 67326262,4 | 0,529 |
| PEo(40:7)\|PEp(40:6)_M+H | 11930807,6 | 8349136,1 | 16242177,2 | 12578629,9 | 11044477,4 | 18414841,3 | 0,481 |
| Total PEo | 3789177899,0 | 2837547438,8 | 4200000012,0 | 4252201493,0 | 3528537495,0 | 5053359711,5 | 0,123 |
| PG(34:1)_M+NH4 | 99279244,1 | 84320230,9 | 106376957,8 | 102209890,0 | 96526990,3 | 110814253,6 | 0,165 |
| PG(34:1)_M+Na | 10841874,0 | 8142828,5 | 12829827,2 | 11431995,0 | 9837915,4 | 12692656,3 | 0,796 |
| T.Pg | 1679918837,0 | 1459078423,3 | 1757060957,8 | 1995846441,5 | 1787281152,3 | 2463983726,5 | 0,002 |
| SM(d18:1/17:0)_M+Na | 272914385,0 | 242781883,0 | 286650366,2 | 314693569,8 | 263585091,1 | 352864796,4 | 0,023 |
| SM(d18:1/18:0)_M+H | 803863829,1 | 616593296,3 | 1049690701,5 | 798178516,6 | 649542121,9 | 1132617614,3 | 1,000 |
| SM(d18:1/18:0)_M+Na | 18587444,7 | 13958858,7 | 28222582,4 | 18722860,0 | 13629428,3 | 25659561,4 | 0,853 |
| SM(d38:1)_M+H | 100219266,6 | 78897377,2 | 131134997,6 | 88812364,6 | 77035989,9 | 138404277,5 | 0,684 |
| SM(d42:2)_M+H | 52772349,2 | 43979487,8 | 58754499,5 | 60662904,3 | 41575794,3 | 72734188,6 | 0,353 |
| Total SM | 1238774298,5 | 957796675,2 | 1513361948,3 | 1280176518,0 | 1092300524,0 | 1649517523,3 | 0,739 |
| TAG(10:0/14:1/18:0)_M+NH4 | 15273157,8 | 14344266,9 | 15417370,7 | 14819575,9 | 14322047,9 | 16221732,0 | 0,739 |
| TAG(10:0/15:0/18:0)_M+NH4 | 12581045,1 | 11618644,7 | 13158001,8 | 11988209,2 | 11151255,5 | 13973514,3 | 0,579 |
| TAG(10:0/16:1/18:0)_M+Na | 13733219,5 | 13297493,1 | 14602170,5 | 13100362,5 | 12310546,4 | 15147540,6 | 0,218 |
| TAG(10:0/16:1/18:0)_M+NH4 | 61095976,3 | 57348511,2 | 62804327,2 | 57574677,8 | 55508146,0 | 65757527,0 | 0,393 |
| TAG(10:0/18:2/18:0)_M+Na | 26336491,6 | 24740361,3 | 26993077,5 | 25165995,9 | 24227532,9 | 27121517,3 | 0,436 |
| TAG(12:0/14:0/18:0)_M+NH4 | 51466047,5 | 48761814,8 | 55079024,5 | 50377914,5 | 48888826,1 | 53396115,9 | 0,739 |
| TAG(12:0/14:0/18:0)_M+Na | 9913086,3 | 9378353,8 | 10306275,2 | 9533504,8 | 9371481,2 | 9905073,7 | 0,436 |
| TAG(12:0/14:0/18:2)_M+NH4 | 20331196,4 | 19556390,8 | 21062495,1 | 19868909,6 | 18818476,5 | 22393721,3 | 0,631 |
| TAG(12:0/15:0/18:0)_M+NH4 | 40889745,8 | 39486964,5 | 42847119,2 | 41297907,0 | 39807756,6 | 43889709,3 | 0,684 |
| TAG(12:0/15:0/18:0)_M+Na | 11161492,3 | 10726130,1 | 11703243,9 | 11240113,4 | 10681162,6 | 12199273,2 | 0,912 |
| TAG(12:0/16:0/20:1)_M+NH4 | 217272585,1 | 207768775,9 | 227647865,8 | 208134543,7 | 193585757,7 | 218600636,0 | 0,165 |
| TAG(12:0/16:0/20:1)_M+Na | 45899349,3 | 43597420,6 | 49415286,0 | 44237314,1 | 39798005,9 | 46309155,3 | 0,143 |
| TAG(12:0/16:1/17:0)_M+Na | 13412896,7 | 12621870,8 | 13854571,6 | 12637676,5 | 12293584,9 | 14133414,1 | 0,190 |
| TAG(14:0/16:1/15:0)_M+Na | 10708519,0 | 10264125,5 | 11152760,6 | 10363652,8 | 9476904,8 | 11871434,7 | 0,353 |
| TAG(12:0/16:1/17:0)_M+NH4 | 61227001,3 | 58805011,1 | 63446287,4 | 59795314,0 | 57422743,3 | 66162153,8 | 0,529 |
| TAG(12:0/16:1/18:0)_M+Na | 50829270,1 | 47310909,0 | 51557590,6 | 47873027,7 | 46574689,9 | 52146812,6 | 0,353 |
| TAG(12:0/18:2/12:0)_M+NH4 | 2358254,4 | 2301092,4 | 2558642,4 | 2441052,7 | 2309362,6 | 2667121,1 | 0,579 |
| TAG(12:0/20:0/22:1)_M+NH4 | 29746150,7 | 25868307,1 | 32500537,9 | 26581355,9 | 24958378,2 | 29374165,0 | 0,143 |
| TAG(12:0/20:0/22:1)_M+Na | 7391274,7 | 6411065,7 | 8184012,4 | 6614568,7 | 6283683,7 | 7059656,2 | 0,105 |
| TAG(14:0/14:0/14:0)_M+Na | 4976840,8 | 4639440,8 | 5252195,9 | 4704171,5 | 4535721,7 | 4939533,1 | 0,28 |
| TAG(14:0/14:0/14:0)_M+NH4 | 16211590,8 | 14912137,3 | 17712745,9 | 15946318,6 | 15428397,1 | 17040670,0 | 0,912 |
| TAG(14:0/14:0/18:0)_M+NH4 | 104283501,3 | 98117577,9 | 108629905,5 | 99472421,2 | 95837050,0 | 105848530,2 | 0,353 |
| TAG(14:0/14:0/18:2)_M+NH4 | 114015082,0 | 106156060,0 | 117845294,5 | 111121245,6 | 107105969,0 | 120533276,2 | 0,971 |
| TAG(14:0/14:0/24:0)_M+NH4 | 22340928,9 | 17942371,5 | 26693409,4 | 22505934,4 | 17398024,3 | 28777202,2 | 0,739 |
| TAG(14:0/15:0/18:1)_M+Na | 37312729,6 | 36025622,3 | 38414136,8 | 35539123,1 | 34752335,2 | 39576985,5 | 0,393 |
| TAG(14:0/15:0/18:1)_M+NH4 | 145186598,2 | 138376575,1 | 148632291,8 | 138598221,3 | 133904207,2 | 154337621,2 | 0,353 |
| TAG(14:0/15:0/18:2)_M+NH4 | 107967415,4 | 101271881,5 | 111073168,5 | 103761389,3 | 100420816,0 | 114446889,2 | 0,631 |
| TAG(14:0/15:0/20:0)_M+NH4 | 49552778,4 | 45368035,7 | 58114450,1 | 51444779,2 | 45270711,0 | 57006814,0 | 1,000 |
| TAG(14:0/15:0/20:0)_ | 12266953,1 | 11318413,2 | 14621121,1 | 12565784,3 | 11036164,9 | 14610072,0 | 0,853 |
| TAG(14:0/15:0/20:1)_M+Na | 29114133,9 | 28292453,3 | 30062868,5 | 27586058,9 | 26858805,3 | 30114641,8 | 0,143 |
| TAG(14:0/15:0/20:1)_M+NH4 | 107210793,7 | 103858044,4 | 112398112,0 | 102147133,9 | 99173567,1 | 111020797,7 | 0,089 |
| TAG(14:0/15:0/20:2)_M+Na | 35122977,4 | 32728348,6 | 35810491,8 | 33256283,7 | 32491911,8 | 36205891,8 | 0,393 |
| TAG(14:0/15:0/20:2)_M+NH4 | 123221703,0 | 115941041,9 | 126813263,4 | 117278325,9 | 114747834,7 | 128273662,9 | 0,436 |
| TAG(14:0/15:0/20:3)_M+NH4 | 49370815,6 | 45496549,9 | 51547819,0 | 47268838,7 | 46091888,8 | 51800728,7 | 0,684 |
| TAG(14:0/16:0/20:0)_M+NH4 | 50226829,8 | 44578743,3 | 56525870,7 | 50579409,7 | 44065854,2 | 58450088,5 | 0,796 |
| TAG(14:0/16:0/20:1)_M+Na | 68814650,5 | 66507986,9 | 71455766,7 | 64592057,2 | 56906667,2 | 66276228,4 | 0,005 |
| TAG(14:0/18:1/18:3)_M+NH4 | 13511379,0 | 10692284,1 | 16073790,3 | 10894859,0 | 10102211,3 | 13097716,3 | 0,165 |
| TAG(14:0/24:0/18:0)_M+NH4 | 6405106,2 | 6120090,1 | 6812632,0 | 6088950,5 | 5802770,7 | 7487756,0 | 0,436 |
| TAG(14:0/24:0/20:1)_M+NH4 | 7599503,1 | 7375410,0 | 7851070,1 | 7292326,5 | 7042333,1 | 8457506,7 | 0,353 |
| TAG(14:1/15:0/22:0)_M+NH4 | 37790243,2 | 36947783,2 | 39616071,8 | 36492204,2 | 35913210,6 | 37433240,6 | 0,063 |
| TAG(14:1/16:1/15:0)_M+NH4 | 20262754,8 | 19527181,9 | 21250467,7 | 19987518,4 | 18298174,8 | 22356438,8 | 0,631 |
| TAG(14:1/18:2/15:0)_M+NH4 | 13365697,3 | 12707132,8 | 13957170,5 | 12852927,5 | 12195568,6 | 14454756,4 | 0,579 |
| TAG(15:0/16:0/22:3)_M+NH4 | 25396335,0 | 24626459,0 | 26835196,0 | 23912248,0 | 22928712,5 | 25463033,7 | 0,019 |
| TAG(15:0/16:0/24:0)_M+NH4 | 7317951,0 | 6899568,2 | 7901991,6 | 7475216,6 | 6759961,7 | 8891204,4 | 1,000 |
| TAG(15:0/16:0/26:0)_M+NH4 | 4142892,7 | 3826329,7 | 4476605,6 | 4015495,6 | 3911710,1 | 4802642,2 | 0,853 |
| TAG(15:0/16:1/26:0)_M+NH4 | 8851322,2 | 8467361,5 | 9316811,5 | 8525937,4 | 8323532,1 | 9872366,6 | 0,579 |
| TAG(15:0/18:0/20:2)_M+NH4 | 26269486,4 | 25738483,6 | 27281342,1 | 25212566,5 | 23739194,1 | 25760818,8 | 0,007 |
| TAG(15:0/18:2/20:1)_M+NH4 | 25053231,4 | 24238592,8 | 26020545,5 | 23369036,2 | 22308825,6 | 24753124,1 | 0,011 |
| TAG(16:0/16:0/16:0)_M+NH4 | 100707853,2 | 97614209,2 | 108599478,7 | 98013567,5 | 94234246,4 | 101807192,7 | 0,165 |
| TAG(16:0/16:0/18:1)_M+Na | 68782173,8 | 66446172,3 | 71385145,1 | 64502066,4 | 57017406,0 | 66208656,8 | 0,005 |
| TAG(16:0/16:0/18:1)_M+NH4 | 320631555,3 | 315470382,8 | 337782253,8 | 293343106,0 | 246803991,2 | 312598536,5 | 0,001 |
| TAG(16:0/16:0/18:2)_M+NH4 | 318831199,7 | 288406448,2 | 352892602,7 | 275616802,9 | 266881185,6 | 292115528,5 | 0,009 |
| TAG(16:0/16:0/18:2)_M+Na | 84826215,2 | 76642545,0 | 94411479,7 | 72856841,3 | 71021119,1 | 77637927,4 | 0,007 |
| TAG(16:0/16:0/20:1)_M+NH4 | 130247508,0 | 116887099,2 | 140567772,2 | 116347131,6 | 103498563,4 | 129349324,2 | 0,063 |
| TAG(16:0/16:0/20:1)_M+Na | 9874610,5 | 8497262,5 | 11073894,3 | 8556749,2 | 7387579,5 | 9253743,2 | 0,075 |
| TAG(16:0/16:0/20:2)_M+NH4 | 1154427277,0 | 1099863893,0 | 1218838742,5 | 1030088253,0 | 802794664,0 | 1115256552,3 | 0,023 |
| TAG(16:0/16:0/20:2)_M+Na | 235846600,3 | 227941641,3 | 242764854,5 | 223193705,4 | 177627725,4 | 228819160,8 | 0,035 |
| TAG(16:0/16:0/20:3)_M+NH4 | 537134415,1 | 484413015,3 | 606415820,6 | 470618685,5 | 403653150,0 | 483637176,4 | 0,029 |
| TAG(16:0/16:0/20:3)_M+Na | 136645810,8 | 122400532,3 | 152938576,4 | 120520505,6 | 104343666,4 | 122820772,9 | 0,019 |
| TAG(16:0/16:1/18:2)_M+NH4 | 152500635,6 | 135566111,2 | 171901961,8 | 136639804,4 | 127378210,2 | 151014978,5 | 0,190 |
| TAG(16:0/16:1/18:2)_M+Na | 32572099,3 | 26989973,9 | 36728641,6 | 28005497,0 | 26250968,9 | 30754185,1 | 0,165 |
| TAG(16:0/18:1/22:1)_M+Na | 6251367,0 | 5123725,8 | 7087145,7 | 5201826,4 | 4673144,7 | 6012015,6 | 0,123 |
| TAG(16:0/18:1/22:1)_M+NH4 | 28122460,7 | 22597024,0 | 30937436,5 | 22981893,4 | 21396397,5 | 26945227,2 | 0,143 |
| TAG(16:0/18:1/24:1)_M+NH4 | 11730740,7 | 10902470,5 | 12325410,9 | 10981542,2 | 10268034,4 | 12500814,4 | 0,481 |
| TAG(16:1/15:0/20:1)_M+NH4 | 73196916,4 | 69705210,9 | 75766890,6 | 70546831,4 | 67777953,0 | 71888454,4 | 0,105 |
| TAG(16:1/15:0/20:1)_M+Na | 18612456,7 | 18332059,1 | 19347096,5 | 17834191,1 | 17583944,1 | 18354889,7 | 0,035 |
| TAG(16:1/16:0/16:1)_M+NH4 | 214023138,8 | 201190407,9 | 227228786,9 | 203729263,2 | 196528372,5 | 229950112,9 | 0,529 |
| TAG(16:1/16:1/16:1)_M+Na | 14814919,5 | 14106002,5 | 16275421,5 | 14169637,4 | 13604358,0 | 16544120,5 | 0,280 |
| TAG(16:1/16:1/20:2)_M+NH4 | 170628490,6 | 150065166,3 | 193675640,2 | 150252070,2 | 144763593,7 | 160116729,6 | 0,143 |
| TAG(16:1/16:1/20:2)_M+Na | 44325004,3 | 39016110,3 | 51698901,0 | 39046557,9 | 36768150,9 | 41477898,3 | 0,105 |
| TAG(17:0/17:0/17:0)_M+Na | 678684026,4 | 506441725,4 | 805992030,4 | 723855237,8 | 507091482,3 | 866340406,5 | 0,579 |
| TAG(17:0/18:1/18:3)_M+NH4 | 5754074,5 | 5505004,5 | 6655954,0 | 5435780,8 | 5292797,7 | 5767275,9 | 0,063 |
| TAG(18:1/18:0/18:1)_M+NH4 | 294306718,6 | 247808573,9 | 314575349,7 | 242004043,7 | 199542090,7 | 290627044,6 | 0,063 |
| TAG(18:1/18:0/18:1)_M+Na | 28481969,5 | 23055219,9 | 32537727,9 | 23476177,3 | 18540594,3 | 27045276,5 | 0,089 |
| TAG(18:1/18:1/18:1)_M+NH4 | 1826884518,5 | 1706607312,8 | 1912452178,0 | 1626211661,0 | 1282538601,2 | 1798088595,5 | 0,063 |
| TAG(18:1/18:1/18:1)_M+Na | 359006182,3 | 351623024,2 | 371645244,6 | 342627205,7 | 278995899,3 | 360046365,1 | 0,089 |
| TAG(18:1/18:1/18:2)_M+NH4 | 604923237,2 | 586191721,7 | 636621865,8 | 567677700,0 | 475664213,2 | 587788123,3 | 0,015 |
| TAG(18:1/18:1/18:2)_M+Na | 148444990,1 | 141958319,0 | 154328718,6 | 139815446,2 | 117589248,3 | 143813454,9 | 0,023 |
| TAG(18:1/18:1/18:3)_M+Na | 59261919,4 | 55988441,7 | 65106301,9 | 57127902,3 | 53176681,5 | 61894376,3 | 0,353 |
| TAG(18:1/18:1/18:3)_M+NH4 | 222861563,6 | 212866928,9 | 247199238,2 | 215647593,3 | 204320470,6 | 232754818,2 | 0,218 |
| TAG(18:1/20:1/18:1)_M+NH4 | 34130018,7 | 30354545,8 | 36028555,0 | 28762767,6 | 24004288,5 | 33898037,4 | 0,035 |
| TAG(18:1/20:1/18:1)_M+Na | 2036005,4 | 1755910,7 | 2380020,1 | 1510661,9 | 1013371,3 | 1869735,1 | 0,019 |
| TAG(18:1/20:1/18:1)\|TAG(18:2/20:1/18:0)_M+NH4 | 35647823,5 | 31968718,6 | 37625459,3 | 30077622,9 | 25481805,4 | 35452949,2 | 0,029 |
| TAG(18:1/20:1/18:2)_M+NH4 | 17635112,1 | 15794885,7 | 18977313,0 | 15153750,0 | 12557597,5 | 16465497,4 | 0,089 |
| TAG(18:2/16:1/18:2)_M+Na | 3868239,3 | 2781581,2 | 4727368,7 | 3213615,3 | 2829617,7 | 3566370,3 | 0,529 |
| TAG(18:2/18:1/15:0)_M+NH4 | 22659943,6 | 20248196,4 | 23959087,3 | 21310725,0 | 19838599,3 | 23475040,1 | 0,280 |
| TAG(18:2/18:1/15:0)_M+Na | 7197384,4 | 6648079,1 | 7928270,7 | 6813138,1 | 6351021,3 | 7748937,5 | 0,436 |
| TAG(24:0/18:2/17:0)_M+NH4 | 3839884,8 | 3618289,5 | 4014022,5 | 3724876,4 | 3560260,3 | 3986883,1 | 0,481 |
| Total TAG | 10042717989,0 | 9749908003,0 | 10496099373,3 | 9524482727,0 | 8725935202,0 | 9938814793,0 | 0,023 |

**Table S2.** Lipid variables by mouse genotype with median, interquartile and p-value in negative ionization mode in the cerebellum.

| **Lipid variables** | **WT** | | | **TG** | | | **p value (Mann-Whitney)** |
| --- | --- | --- | --- | --- | --- | --- | --- |
|  | **Median** | **IQ1** | **IQ3** | **Median** | **IQ1** | **IQ3** |  |
| Cer(d18:1/16:0)_M-H | 26234061,0 | 19174613,5 | 29182627,4 | 26483804,6 | 13388527,9 | 34269657,2 | 1,000 |
| Cer(d18:1/16:0)_M+CH3COO | 1293192538,1 | 1229108760,2 | 1708513763,1 | 1273336163,6 | 576793430,0 | 1853471378,3 | 0,684 |
| Cer(d18:0_18:1)_M-H | 141539335,0 | 85984497,1 | 164048808,2 | 124065815,3 | 22241637,3 | 181390296,1 | 0,739 |
| Cer(d18:0_18:1)_M+CH3COO | 211562121,9 | 126697019,4 | 248376406,2 | 187987733,8 | 33464147,6 | 270653633,6 | 0,739 |
| Cer(d18:1/20:0)_M-H | 53253077,6 | 38449457,4 | 62713100,6 | 39414887,6 | 2886683,9 | 53095585,2 | 0,143 |
| Cer(d18:1/20:0)_M+CH3COO | 94077458,9 | 66766285,2 | 109754516,4 | 68491628,5 | 5569040,3 | 93248208,2 | 0,143 |
| Cer(d18:1/24:0)_M-H | 5835664,1 | 4368403,1 | 6414458,9 | 4919332,5 | 730303,3 | 6277166,3 | 0,529 |
| Cer(d18:1/24:0)_M+CH3COO | 15113606,0 | 11053074,3 | 16054376,7 | 12332194,0 | 3391825,9 | 16680640,1 | 0,529 |
| Cer(d18:1/24:1)_M-H | 48966648,8 | 39757773,3 | 54936256,3 | 52210856,6 | 18346254,6 | 59649786,1 | 0,739 |
| Cer(d18:1/24:1)_M+CH3COO | 111277566,1 | 87652976,5 | 118920868,5 | 117466953,7 | 40622357,7 | 134153974,5 | 0,739 |
| Total Cer | 2135563509,5 | 1872128248,9 | 2325211500,9 | 2103683395,9 | 732418880,7 | 2481909695,1 | 1,000 |
| CL(16:1_18:1_20:4_20:4)_M-H | 9508976,1 | 6867830,9 | 9802566,6 | 9456488,6 | 8036700,7 | 10451757,7 | 0,684 |
| CL(16:1_18:1_20:4_22:6)_M-H | 9467482,3 | 7151471,5 | 10323416,6 | 8891788,5 | 7550921,7 | 10024280,2 | 0,853 |
| CL(18:1/18:1/18:1/18:1)_M-H | 38889366,9 | 30020424,1 | 43305058,5 | 36664926,4 | 24518038,4 | 38729638,1 | 0,315 |
| CL(18:1_18:1_18:1_18:2)_M-H | 23225747,1 | 15809491,3 | 25090191,9 | 21600770,7 | 16035118,8 | 25210792,5 | 0,853 |
| CL(18:1_18:1_18:1_20:4)_M-H | 26085511,9 | 19386590,3 | 27542370,9 | 25735440,6 | 17667483,3 | 26372440,2 | 0,739 |
| CL(18:1_18:1_18:1_22:6)_M-H | 38023561,6 | 27143766,7 | 43012376,6 | 34882176,0 | 25097192,0 | 40989138,0 | 0,481 |
| CL(18:1_18:1_20:4_20:4)_M-H | 30078488,9 | 21928735,5 | 32973712,1 | 29396577,4 | 22945001,5 | 33452247,2 | 0,971 |
| CL(18:1_18:1_20:4_22:6)_M-H | 32289698,3 | 23792558,0 | 35660550,7 | 30063726,0 | 22251331,0 | 34709754,4 | 0,481 |
| CL(18:1_18:1_22:6_22:6)_M-H | 37068695,8 | 25912810,0 | 38699664,9 | 31970717,0 | 23090129,6 | 37040052,0 | 0,315 |
| CL(18:1_18:2_22:6_22:6)_M-H | 18085022,2 | 14059541,5 | 19607286,5 | 16600983,3 | 12287231,8 | 19310575,5 | 0,481 |
| CL(18:1_20:4_22:6_22:6)_M-H | 13524342,7 | 11723584,3 | 15199894,3 | 11639089,2 | 9929640,3 | 13986563,5 | 0,19 |
| CL(18:2/18:2/18:2/18:2)_M-H | 5684210,1 | 4276942,3 | 6284035,5 | 5898379,3 | 4697837,9 | 7053745,5 | 0,684 |
| Total CL | 283195478,1 | 206418434,3 | 304512915,6 | 262454387,1 | 197499894,5 | 296971448,7 | 0,393 |
| FA(16:0)_M-H | 1203743477,0 | 1015090765,5 | 1496550495,6 | 1276486185,4 | 1093326187,2 | 1430227986,0 | 0,529 |
| FA(17:0)_M-H | 222591749,0 | 212232426,7 | 273590013,6 | 220390742,2 | 203613351,8 | 243380397,2 | 0,529 |
| FA(18:0)_M-H | 2353846763,2 | 2290723143,7 | 2687069971,9 | 2487125758,3 | 2191517513,5 | 2680433519,6 | 0,739 |
| FA(18:1)_M-H | 721661746,9 | 613023641,2 | 1474447091,7 | 988353383,3 | 698492037,0 | 1276530262,6 | 0,579 |
| FA(18:2)_M-H | 51261404,5 | 42169415,7 | 78346820,4 | 62328883,9 | 54590546,4 | 83498043,6 | 0,481 |
| FA(20:0)_M-H | 58061371,4 | 52882232,2 | 63826717,3 | 57602870,7 | 47358670,7 | 66457181,2 | 0,971 |
| FA(20:1)_M-H | 58117643,0 | 49435925,9 | 161429044,3 | 95303106,1 | 63955132,6 | 124586178,2 | 0,247 |
| FA(20:4)_M-H | 1079174811,1 | 856275394,2 | 2624479542,6 | 1509286910,0 | 983325976,0 | 2003775343,0 | 0,631 |
| FA(22:0)_M-H | 44662024,5 | 42015278,3 | 49885606,6 | 49831390,0 | 35127042,7 | 56489527,4 | 0,684 |
| FA(22:1)_M-H | 9980558,6 | 8875211,9 | 24096682,4 | 14707385,9 | 11059228,5 | 18134427,4 | 0,165 |
| FA(24:0)_M-H | 72433683,9 | 68441790,4 | 84362317,3 | 83699192,6 | 56017090,6 | 93989277,1 | 0,684 |
| FA(24:1)_M-H | 15588653,3 | 13947611,2 | 22111315,1 | 20355436,9 | 16505934,4 | 24287984,6 | 0,075 |
| FA(26:0)_M-H | 47990429,1 | 46174016,6 | 50903317,5 | 52175616,4 | 33109134,9 | 57459880,6 | 0,912 |
| Total FA | 5901417484,6 | 5281358607,1 | 8939689304,3 | 6650892499,5 | 5398596711,3 | 7849805926,0 | 0,529 |
| LPC(16:0)_M-CH3 | 22616009,1 | 19050580,5 | 26243139,9 | 26880188,6 | 23692419,0 | 35694448,2 | 0,052 |
| LPC(16:0)_M+CH3COO | 91278035,5 | 77196079,5 | 108462343,5 | 108399659,6 | 92257011,5 | 145756878,2 | 0,063 |
| LPC(16:1)_M-CH3 | 18262918,8 | 14428785,5 | 22885451,2 | 23007597,3 | 19451706,9 | 44218473,7 | 0,035 |
| LPC(18:0)_M-CH3 | 11914679,1 | 10322690,8 | 12989958,7 | 14116268,7 | 11626283,6 | 15778877,4 | 0,075 |
| LPC(18:0)_M+CH3COO | 55870307,7 | 48274446,4 | 61439496,4 | 68865555,7 | 59199177,0 | 72997221,9 | 0,035 |
| LPC(18:1)_M-CH3 | 6721254,2 | 5354233,0 | 10942684,2 | 7540480,9 | 6417289,6 | 12961794,7 | 0,315 |
| LPC(18:1)_M+CH3COO | 32321830,3 | 24915865,2 | 51857233,2 | 35198395,3 | 30450117,6 | 61121096,8 | 0,353 |
| LPC(20:1)_M+CH3COO | 3108282,8 | 2623344,1 | 5281594,6 | 3692388,5 | 3080621,5 | 6073054,2 | 0,393 |
| LPC(20:4)_M+CH3COO | 13009830,8 | 10726382,7 | 23222857,5 | 14061608,9 | 11535964,0 | 23141456,5 | 0,684 |
| LPC(22:6)_M+CH3COO | 28593168,4 | 25320828,2 | 56669032,9 | 28862075,9 | 23944145,0 | 41741064,1 | 0,796 |
| Total LPC | 287450833,7 | 242209635,9 | 353497439,3 | 330134925,9 | 276573820,9 | 478678182,2 | 0,19 |
| LPE(18:0)_M-H | 29653535,8 | 22401387,4 | 32266898,0 | 40231632,5 | 33385524,3 | 46031685,7 | 0,005 |
| LPE(20:4)_M-H | 18112032,7 | 15205272,4 | 31196089,7 | 22412835,7 | 18448972,2 | 54556502,7 | 0,315 |
| LPE(22:6)_M-H | 60942387,2 | 51576354,3 | 107083419,6 | 72585518,2 | 56764066,0 | 170143826,9 | 0,315 |
| Total LPE | 110289038,2 | 93717955,7 | 159827293,2 | 129013503,6 | 110951357,7 | 274757857,0 | 0,123 |
| LPI(18:0)_M-H | 55254675,9 | 42274410,0 | 58271158,3 | 88195079,8 | 70757363,0 | 103855874,0 | <0,001 |
| LPI(20:4)_M-H | 32760710,7 | 31045249,5 | 41381665,0 | 43497230,2 | 37323185,9 | 59116745,5 | 0,035 |
| Total LPI | 89532845,8 | 79166089,7 | 102562489,6 | 124502722,0 | 108519146,4 | 174768901,6 | <0,001 |
| LPS(18:0)_M-H | 11600477,0 | 9252011,0 | 12731391,8 | 14995201,5 | 13620539,0 | 20287483,8 | <0,001 |
| LPS(22:6)_M-H | 48573587,7 | 40620720,2 | 62306887,8 | 57923415,4 | 54295509,7 | 89960382,2 | 0,143 |
| Total LPS | 58957912,3 | 51632796,4 | 75758803,1 | 72949559,5 | 67911968,9 | 106386183,9 | 0,075 |
| PC(16:0/16:0)_M-CH3 | 126686161,1 | 83345662,3 | 134471127,8 | 93997490,9 | 40112416,5 | 103499618,7 | 0,063 |
| PC(16:0/16:0)_M+CH3COO-CH3 | 44058850,3 | 33549510,6 | 52531194,5 | 41061210,2 | 25086643,3 | 45419242,0 | 0,631 |
| PC(16:0_20:4)_M-CH3 | 35217378,7 | 34452420,7 | 38257429,8 | 37072423,7 | 34221581,5 | 42122673,9 | 0,436 |
| PC(16:0_20:4)_M+CH3COO | 388266930,6 | 381661206,6 | 435040781,7 | 460667502,0 | 380632253,5 | 490877378,0 | 0,28 |
| PC(16:0/22:4)_M+CH3COO | 39384564,9 | 38688796,8 | 41095763,3 | 43587597,6 | 34889310,7 | 55058010,9 | 1,000 |
| PC(18:0/16:1)_M+CH3COO | 60134449,0 | 56193298,9 | 62831264,8 | 63941001,3 | 59690917,3 | 68418524,2 | 0,075 |
| PC(16:0_18:1)_M+CH3COO // M+CH3COO | 59999425,2 | 55920606,9 | 62514616,6 | 63568069,6 | 59829141,1 | 67933213,8 | 0,089 |
| PC(16:1/22:6)_M+CH3COO | 3474881,8 | 3029384,9 | 6486896,7 | 3877756,1 | 2897593,7 | 4880662,6 | 0,796 |
| PC(17:0/16:0)_M+CH3COO | 19091940,8 | 13611540,2 | 22386350,3 | 15383016,8 | 5736909,5 | 17729849,9 | 0,247 |
| PC(17:0/18:1)_M+CH3COO | 29276196,2 | 26620301,5 | 30302127,3 | 28623322,9 | 25188646,8 | 33583900,7 | 0,853 |
| PC(18:0/18:1)_M-CH3 | 137781084,7 | 110334366,9 | 142874658,3 | 126511729,5 | 115612798,3 | 143634306,7 | 0,739 |
| PC(18:0/18:1)_M+CH3COO | 1644519127,5 | 1304782982,2 | 1692915182,2 | 1480694126,3 | 1358177628,7 | 1683529521,5 | 0,739 |
| PC(18:0/20:4)_M-CH3 | 42719697,2 | 41884105,9 | 44341099,0 | 46255053,1 | 43272269,8 | 51403208,9 | 0,029 |
| PC(18:0/20:4)_M+CH3COO | 490740294,7 | 481785753,0 | 516203636,7 | 525337499,8 | 486845488,0 | 551526128,4 | 0,143 |
| PC(18:0/22:4)_M+CH3COO | 36791142,9 | 33255818,3 | 39002445,4 | 34644203,6 | 31720849,0 | 50398489,2 | 1,000 |
| PC(18:0/22:5)_M+CH3COO | 31701176,2 | 29686146,7 | 33969048,6 | 35512636,8 | 31665737,0 | 39052207,8 | 0,19 |
| PC(18:0/22:6)_M+CH3COO | 595745407,2 | 536637409,5 | 660314215,3 | 530602316,5 | 467780782,4 | 613237961,5 | 0,105 |
| PC(18:1/18:1)_M-CH3 | 24554276,5 | 23712622,5 | 25833072,2 | 27960592,0 | 25189943,6 | 30384669,2 | 0,005 |
| PC(18:1/18:1)_M+CH3COO | 395125554,5 | 384403706,8 | 416710013,8 | 431084999,8 | 413792596,8 | 461306745,4 | 0,009 |
| PC(18:1/20:4)_M+CH3COO | 67093478,8 | 58300364,2 | 77778258,8 | 61285322,6 | 53087048,0 | 88624116,4 | 0,631 |
| PC(18:1_22:0)_M+CH3COO | 61572095,8 | 57223797,9 | 70085782,7 | 66344851,1 | 51982076,7 | 75134473,3 | 0,529 |
| PC(22:6/22:6)_M+CH3COO | 19995129,2 | 15952292,4 | 74622482,1 | 18412743,9 | 16149083,7 | 24290081,0 | 0,739 |
| PC(30:0)_M-CH3 | 2058334,7 | 1800532,4 | 2150681,7 | 1778312,7 | 1501573,2 | 1944697,5 | 0,123 |
| PC(30:0)_M+CH3COO | 22869549,1 | 19870711,0 | 25259627,0 | 20356484,5 | 17504714,8 | 23820745,6 | 0,315 |
| PC(32:0)_M+CH3COO | 1380093598,9 | 908664109,2 | 1444227299,0 | 1012863451,9 | 437450532,1 | 1118198101,7 | 0,063 |
| PC(32:1)_M+CH3COO | 93383084,6 | 86603968,6 | 104404570,2 | 98311780,5 | 86838772,3 | 110227701,9 | 0,529 |
| PC(33:1)_M+CH3COO | 19915524,0 | 18789939,5 | 21331577,4 | 20790978,6 | 18841786,7 | 24378677,8 | 0,579 |
| PC(34:1)_M-CH3 | 294928111,9 | 256961437,9 | 317939233,7 | 266193441,8 | 248231525,2 | 299977082,8 | 0,315 |
| PC(34:1)_M+CH3COO | 3254511554,7 | 2838819720,7 | 3525803882,7 | 2932264571,8 | 2721937902,9 | 3265756591,5 | 0,247 |
| PC(34:2)_M+CH3COO | 58173116,3 | 51718707,5 | 69454999,9 | 56111159,4 | 43432762,0 | 83358938,4 | 0,971 |
| PC(34:5)_M-CH3 | 9062303,1 | 7863458,4 | 10207242,3 | 9478301,1 | 8446731,6 | 11007851,6 | 0,393 |
| PC(36:2)_M+CH3COO | 61229973,9 | 54438259,9 | 66070437,2 | 64114887,6 | 56228291,4 | 79854148,0 | 0,353 |
| PC(36:2)_M-CH3 | 62176519,8 | 55835513,4 | 69343426,4 | 86267998,4 | 67184548,8 | 109952309,3 | 0,011 |
| PC(36:3)_M+CH3COO | 14995721,0 | 14103919,2 | 17630553,6 | 16433457,6 | 12092898,4 | 18690830,4 | 1,000 |
| PC(16:0_20:3)_M+CH3COO | 32850074,7 | 30535883,6 | 35458543,1 | 33087143,2 | 29405867,0 | 40332693,2 | 1,000 |
| PC(37:4)_M+CH3COO | 6267237,0 | 5786631,3 | 7055186,2 | 7077526,5 | 5951103,7 | 7957268,5 | 0,247 |
| PC(38:2)_M+CH3COO | 75619318,1 | 69593878,0 | 79452890,3 | 73379717,2 | 67246441,0 | 100623718,0 | 0,853 |
| PC(38:3)_M+CH3COO | 25350357,6 | 20301453,9 | 27517477,3 | 23110885,7 | 20504689,8 | 27479248,1 | 0,684 |
| PC(38:6)_M+CH3COO | 601161067,8 | 568399048,2 | 676785554,0 | 531572914,4 | 482815142,8 | 575195031,6 | 0,011 |
| PC(40:5)_M+CH3COO | 11089127,8 | 10590805,9 | 14598300,7 | 11759750,4 | 10142338,0 | 15276063,3 | 0,912 |
| PC(40:6)_M-CH3 | 41473031,2 | 36964162,0 | 45366739,1 | 36212731,4 | 31406135,7 | 41962036,4 | 0,123 |
| PC(40:7)_M+CH3COO | 175850332,0 | 154737040,6 | 234155616,8 | 171468247,9 | 146396526,2 | 179960664,2 | 0,436 |
| PC(42:10)_M+CH3COO | 8090139,3 | 6448118,9 | 23051667,5 | 8520800,6 | 7297237,1 | 11161947,1 | 1,000 |
| PC(42:7)_M+CH3COO | 19324056,3 | 18207883,8 | 23144677,5 | 18325231,5 | 15867481,4 | 22645264,5 | 0,353 |
| PC(42:8)_M+CH3COO | 356541,1 | 255962,6 | 1181483,1 | 370888,8 | 351377,3 | 684315,3 | 0,579 |
| PC(44:10)_M+CH3COO | 971323,2 | 632917,5 | 2032423,4 | 1147014,5 | 941873,2 | 1448932,7 | 0,529 |
| Total PC | 10808306072,9 | 9820561240,7 | 11079053405,5 | 9781042060,9 | 9395698609,3 | 10244398708,1 | 0,052 |
| PCo(34:2)\|PCp(34:1)_M+CH3COO | 12942431,4 | 11422438,0 | 14716292,2 | 14561200,9 | 12213478,9 | 16210071,2 | 0,218 |
| PCo(34:1)\|PCp(34:0)_M+CH3COO | 25677348,6 | 22048121,9 | 27153201,5 | 26210279,8 | 22853201,8 | 31590365,9 | 0,579 |
| Total Pco | 38940824,7 | 34104315,0 | 40829891,2 | 41213447,3 | 35066680,7 | 46325091,6 | 0,28 |
| PE(16:0/20:3)_M-H | 11862320,7 | 10367936,3 | 13065825,5 | 13019096,8 | 10832767,0 | 15372934,8 | 0,393 |
| PE(16:0/20:4)_M-H | 121219151,3 | 108970001,6 | 137620430,2 | 111959060,6 | 90042179,2 | 175480980,0 | 0,912 |
| PE(16:0/22:4)_M-H | 70717750,3 | 59956597,6 | 74658531,3 | 94143489,9 | 57939225,5 | 102423612,9 | 0,075 |
| PE(16:0/22:5)_M-H | 19908513,0 | 17490410,5 | 23259140,1 | 22241601,0 | 19895055,6 | 27165253,0 | 0,218 |
| PE(16:0/22:6)_M-H | 1056571965,6 | 808820821,7 | 1100025889,5 | 970847478,2 | 865052405,6 | 1065007946,5 | 0,529 |
| PE(18:0_18:1)_M-H | 709166716,5 | 677846029,4 | 752620974,1 | 948685516,0 | 580933285,2 | 1065880964,7 | 0,165 |
| PE(18:0/18:2)_M-H | 25938918,2 | 22438264,7 | 28883141,7 | 29477698,4 | 24917918,1 | 33856667,2 | 0,143 |
| PE(18:0/20:4)_M-H | 1441176556,0 | 1152176037,2 | 1527615658,5 | 1388356082,6 | 1295631919,4 | 1531787233,0 | 1,000 |
| PE(18:0/22:6)_M-H | 3613020625,4 | 2767641079,6 | 3856768592,7 | 3619593839,8 | 3131793866,3 | 3854064246,3 | 0,796 |
| PE(18:1/18:1)_M-H | 719965932,3 | 625554549,1 | 735521680,7 | 695374030,2 | 629996440,9 | 805807621,0 | 0,912 |
| PE(18:1/20:4)_M-H | 602839052,0 | 579691381,6 | 618769449,5 | 611775281,7 | 526773871,5 | 712423459,5 | 0,796 |
| PE(18:1_18:2)_M-H | 38127879,2 | 34419512,3 | 41869707,2 | 44312924,5 | 36344957,2 | 54454015,9 | 0,165 |
| PE(34:2)_M-H | 33534672,1 | 28676291,6 | 36533079,9 | 27110913,2 | 21213525,1 | 35494233,5 | 0,106 |
| PE(38:2)_M-H | 54107793,7 | 48465146,3 | 59334348,7 | 77431678,8 | 57071047,9 | 100889685,1 | 0,019 |
| Total PE | 8643331291,6 | 6941848002,3 | 8842060447,5 | 8477851537,9 | 7999296601,3 | 9306769836,7 | 0,912 |
| PEo(36:4)\|PEp(36:3)_M-H | 18944843,8 | 16292693,3 | 20676323,3 | 22724385,1 | 17612117,3 | 26874291,8 | 0,089 |
| PEo(38:6)\|PEp(38:5)_M-H | 101173582,9 | 72657242,5 | 114588560,3 | 104773096,7 | 87944885,7 | 110375548,2 | 0,796 |
| PEo(32:2)\|PEp(32:1)_M-H | 9371323,9 | 7164742,8 | 10096103,8 | 9779449,5 | 8229430,9 | 11897282,1 | 0,315 |
| PEo(16:1/20:3)\|PEp(16:0/20:3)_M-H | 25987196,1 | 22610117,9 | 28878457,2 | 36156843,1 | 29049037,0 | 41200211,7 | 0,002 |
| PEo(16:1/20:4)\|PEp(16:0/20:4)_M-H | 229591748,9 | 170229258,2 | 240934481,3 | 236810287,8 | 205865942,7 | 272818394,2 | 0,353 |
| PEo(16:1/20:4)\|PEp(16:0/20:4)_M+NaCH3COO | 11666458,0 | 9543720,8 | 12593437,9 | 12596086,3 | 9712379,9 | 14457913,0 | 0,481 |
| PEo(16:1/22:5)PEp(16:0/22:5)_M-H | 615037687,0 | 559672156,4 | 669672794,1 | 716314478,0 | 597921722,9 | 806574727,1 | 0,052 |
| PEo(16:1/22:5)\|PEp(16:0/22:5)_M-H | 16583836,6 | 16074015,3 | 18410970,6 | 22701687,1 | 17736942,5 | 30096756,3 | 0,009 |
| PEo(16:1/22:6)\|PEp(16:0_22:6)_M+NaCH3COO | 21476703,1 | 16892928,5 | 23391950,5 | 18098355,6 | 15954848,6 | 20049099,1 | 0,123 |
| PEo(16:1_22:6)\|PEp(16:0_22:6)_M-H | 823175649,0 | 606342614,6 | 870848798,7 | 748732219,8 | 677108127,5 | 824792560,6 | 0,529 |
| PEo(16:1_22:6)\|PEp(16:0_22:6)_M+NaCH3COO | 21476703,1 | 16878101,6 | 23359805,1 | 18070188,6 | 15950068,8 | 20018814,1 | 0,143 |
| PEo(18:1/22:6)\|PEp(18:0/22:6)_M-H // M-H | 42905031,0 | 38578296,5 | 48311582,4 | 41319829,6 | 37343049,3 | 47132179,0 | 0,912 |
| PEo(18:1/22:6)\|PEp(18:0/22:6)_M+NaCH3COO | 61054019,0 | 45995377,7 | 64814994,1 | 49353020,4 | 43265469,4 | 52064146,9 | 0,063 |
| PEo(18:1_22:6)\|PEp(18:0_22:6)_M-H | 2301717141,4 | 1600477973,1 | 2522704794,6 | 1909524613,8 | 1821329538,3 | 2361191949,5 | 0,579 |
| PEo(18:1_22:6)\|PEp(18:0_22:6)_M+NaCH3COO | 61136613,1 | 46004464,9 | 64814994,1 | 49421793,4 | 43265469,4 | 52202472,2 | 0,063 |
| PEo(18:2/16:1)\|PEp(18:1/16:1)_M-H | 60364726,7 | 49116371,4 | 63619357,1 | 65450080,9 | 52103097,9 | 72380255,4 | 0,19 |
| PEo(18:2/18:1)\|PEp(18:1/18:1)_M-H | 1581981793,6 | 1346647763,5 | 1723617643,5 | 1642734722,4 | 1434585197,5 | 1764060335,3 | 0,579 |
| PEo(18:2/18:2)\|PEp(18:1_18:2)\|PEp(18:2/18:1)_M-H | 44889604,7 | 36748365,7 | 60019425,1 | 59411561,3 | 47144702,7 | 77075594,3 | 0,165 |
| PEo(18:2/20:4)\|PEp(18:1/20:4)_M+NaCH3COO | 14018320,6 | 13494257,2 | 14899618,7 | 14407008,1 | 12025733,4 | 18233932,5 | 0,912 |
| PEo(18:2/22:6)\|PEp(18:1/22:6)_M-H | 478518446,5 | 443953713,0 | 508718564,6 | 517915469,2 | 422092088,4 | 542370832,8 | 0,481 |
| PEo(18:2/22:6)\|PEp(18:1/22:6)_M+NaCH3COO | 15196957,5 | 14502892,4 | 16909360,0 | 15307437,0 | 13241959,6 | 16845478,1 | 0,529 |
| PEo(35:2)\|PEp(17:0/18:1)_M-H | 39746489,6 | 36703385,9 | 46092923,0 | 56192130,4 | 30329008,2 | 63045766,7 | 0,247 |
| PEo(37:3)\|PEp(37:2)_M-H | 7972903,2 | 6884811,8 | 8419832,3 | 11622850,6 | 6647043,2 | 17386047,1 | 0,143 |
| PEo(38:5)\|PEp(38:4)_M-H | 159980914,0 | 144486709,0 | 187466311,6 | 189969105,4 | 144521693,2 | 208008211,9 | 0,19 |
| PEo(38:5)\|PEp(38:4)_M+NaCH3COO | 2556636,4 | 2173272,5 | 2894155,1 | 2662930,9 | 2233285,2 | 3364804,1 | 0,631 |
| PEo(39:5)\|PEp(17:0/22:4)_M-H | 9953687,5 | 9303455,4 | 10378674,6 | 11047933,2 | 8403667,4 | 12870815,7 | 0,105 |
| PEp(17:0/22:6)_M-H | 44247076,1 | 37802073,2 | 61861764,7 | 53786446,6 | 48304944,9 | 78178887,0 | 0,28 |
| Total PEo/Pep | 6977344524,5 | 5403394006,5 | 7240594644,3 | 6719489608,7 | 5863740349,5 | 7352016624,6 | 1,000 |
| PG(18:1/18:1)_M-H | 19161299,6 | 17051276,7 | 20188055,5 | 21691465,4 | 17888811,9 | 27044144,0 | 0,218 |
| PG(22:6/22:6)_M-H | 13666705,9 | 8498203,5 | 96570356,8 | 12734222,7 | 10599756,3 | 24919849,3 | 1,000 |
| PG(34:1)_M-H | 237298777,2 | 206208570,7 | 259955092,8 | 261944409,4 | 213997532,0 | 307452240,2 | 0,436 |
| PG(36:4)_M-H | 7341028,4 | 6154349,6 | 13325561,6 | 7899454,4 | 6977727,8 | 9592674,1 | 0,481 |
| PG(38:4)_M-H | 20525223,1 | 17454573,4 | 23549489,5 | 21396056,2 | 19013101,6 | 26536624,2 | 0,436 |
| PG(38:5)_M-H | 2966590,3 | 2487959,5 | 7310110,2 | 3351744,2 | 3226735,0 | 4224377,9 | 0,481 |
| PG(38:6)_M-H | 3716277,0 | 3026877,5 | 10559364,8 | 4202755,9 | 3754122,2 | 5290435,0 | 0,436 |
| Total PG | 319751155,3 | 272732353,1 | 395512270,8 | 366103620,8 | 286342384,5 | 440852734,6 | 0,579 |
| PI(16:0/20:4)_M-H | 403315183,0 | 392752789,7 | 421100760,5 | 470293567,2 | 394672686,6 | 520372116,1 | 0,123 |
| PI(18:0/20:4)_M-H | 2570164535,1 | 2385024577,8 | 2744056207,1 | 2650423477,7 | 2278933839,6 | 3119656812,8 | 0,529 |
| PI(18:0/22:6)_M-H | 231756382,3 | 226759143,1 | 240314784,3 | 251781412,6 | 208983389,4 | 298396746,7 | 0,579 |
| PI(18:1/18:1)_M-H | 42891324,9 | 40076548,1 | 44108890,7 | 48612652,3 | 39337579,4 | 56984387,3 | 0,165 |
| PI(18:1/20:4)_M-H | 383944583,8 | 359447036,3 | 431980947,0 | 458646914,9 | 388377029,8 | 517532749,3 | 0,19 |
| Total PI | 3617863490,9 | 3405011946,7 | 3927739277,2 | 3887329658,0 | 3284084748,1 | 4494451644,7 | 0,481 |
| PS(34:1)_M-H | 124687398,3 | 93641902,7 | 133236894,3 | 115711948,4 | 106983557,5 | 130822139,5 | 0,971 |
| PS(38:6)_M-H | 26321186,2 | 24708824,8 | 30523073,8 | 27820362,6 | 24527003,9 | 32354335,5 | 0,739 |
| PS(39:6)_M-H | 3738209,4 | 3458829,9 | 4360030,8 | 3970676,3 | 3802162,7 | 4661301,0 | 0,19 |
| PS(18:0/18:1)_M-H | 1331478695,0 | 1229482154,1 | 1450222838,7 | 1458961200,4 | 1109042933,2 | 1553672651,1 | 0,28 |
| PS(38:3)_M-H | 35353898,1 | 33378919,9 | 43281173,2 | 38700058,0 | 35064238,6 | 52511153,4 | 0,19 |
| PS(18:0/20:4)_M+Na-2H | 17080726,8 | 13114152,0 | 17833856,1 | 13894118,9 | 11755424,8 | 16170293,2 | 0,315 |
| PS(18:0/20:4)_M-H | 499701931,6 | 474410841,4 | 525155478,9 | 503165237,5 | 452972649,7 | 663697904,0 | 0,631 |
| PS(18:0/22:4)_M-H | 585357653,1 | 522938721,7 | 637837881,4 | 744562330,4 | 555957477,2 | 901829239,9 | 0,043 |
| PS(18:0/22:5)_M-H | 50204529,3 | 44828935,3 | 58523944,9 | 58681759,8 | 52314412,5 | 70323728,7 | 0,143 |
| PS(18:0/22:6)_M-H | 3205360277,4 | 2942759854,9 | 3459510080,8 | 2953418592,2 | 2783023984,4 | 3484088469,4 | 0,481 |
| PS(18:0/22:6)_M+Na-2H | 65635691,1 | 55836005,1 | 67370818,0 | 48489084,2 | 40576210,8 | 57251815,9 | 0,005 |
| PS(18:1/18:1)_M-H | 1147903398,3 | 1061533585,0 | 1184963268,9 | 1126028786,3 | 1005211501,9 | 1369534233,0 | 0,853 |
| PS(18:1/20:4)_M-H | 69366212,3 | 65511894,2 | 97381862,2 | 83461553,8 | 69933695,6 | 104213449,9 | 0,218 |
| PS(18:1/22:6)_M-H | 7262831,2 | 6435535,8 | 7832033,6 | 7233528,2 | 6683620,8 | 8077954,1 | 0,853 |
| PS(18:1/22:6))_M-H | 106042304,6 | 97112029,7 | 152589147,0 | 113739502,9 | 97919069,4 | 130529321,0 | 0,853 |
| PS(18:1_20:1)_M-H | 20427891,6 | 18954163,9 | 25099672,5 | 20796164,1 | 16194671,4 | 33879132,1 | 1,000 |
| PS(22:4/22:6)_M-H | 95055625,1 | 81400925,4 | 227669314,1 | 128225795,0 | 97628374,0 | 145890564,8 | 0,436 |
| PS(22:6/22:6)_M-H | 86169421,3 | 75163880,3 | 316014296,0 | 86616587,2 | 78865124,7 | 116857898,4 | 1,000 |
| PS(22:6/22:6)_M+Na-2H | 3118023,3 | 2815866,6 | 11643181,8 | 2987895,8 | 2772166,9 | 4426166,6 | 0,631 |
| PS(36:1)_M-H | 1339020513,6 | 1236075168,8 | 1465940377,5 | 1480456087,7 | 1114362393,8 | 1565301870,1 | 0,247 |
| PS(36:4)_M-H | 12810169,7 | 12167785,5 | 13672148,1 | 14562818,6 | 12370697,2 | 18251052,5 | 0,393 |
| PS(40:2)_M-H | 24720746,6 | 21420529,7 | 25923611,4 | 26993345,4 | 16716044,7 | 31626376,6 | 0,393 |
| PS(40:7)_M+Na-2H | 2146224,9 | 2000902,5 | 3017288,3 | 2126261,0 | 1845030,3 | 2485532,8 | 0,28 |
| PS(42:10)_M-H | 6656231,4 | 5985810,1 | 18826916,6 | 7792247,0 | 7255323,8 | 10621779,1 | 0,315 |
| PS(42:5)_M-H | 2977787,6 | 2512564,9 | 4444068,3 | 2885399,1 | 2858168,6 | 3314427,9 | 0,853 |
| PS(42:7)_M-H | 13348210,9 | 12073294,4 | 15917055,4 | 12505186,3 | 9170576,8 | 17057369,8 | 0,353 |
| PS(44:10)_M+Na-2H | 2722951,4 | 2296492,3 | 7743117,3 | 3736953,1 | 2793861,6 | 4091137,3 | 529 |
| PS(44:11)_M-H | 6340848,0 | 5713383,7 | 19475522,3 | 7252357,2 | 6601975,2 | 10282734,8 | 0,631 |
| PS(44:7)_M+Na-2H | 2160158,6 | 2001318,5 | 4462609,3 | 2982812,8 | 2228266,7 | 3733686,7 | 0,353 |
| Total PS | 9047690230,0 | 8726367004,3 | 9234919849,5 | 8979740962,4 | 8363977558,2 | 10434031195,7 | 0,971 |

**Table S3.** Lipid variables by mouse genotype with median, interquartile and p-value in positive ionization mode in the amygdala.

| **Lipid variables** | **WT** | | | **TG** | | | **p value (Mann-Whitney)** |
| --- | --- | --- | --- | --- | --- | --- | --- |
|  | **Median** | **IQ1** | **IQ3** | **Median** | **IQ1** | **IQ3** |  |
| CE(20:0)_M+NH4 | 6104522,6 | 5743153,5 | 6503905,3 | 5608827,9 | 5190144,6 | 6095202,8 | 0,065 |
| Cer(d16:1/17:0)_M+H-H2O | 10210306,5 | 9285752,2 | 10911917,2 | 9855650,7 | 9375215,0 | 10017517,2 | 0,400 |
| Cer(d16:1/17:0)_M+H | 9103601,8 | 8158926,6 | 9929427,2 | 8627953,4 | 8107251,0 | 8818987,9 | 0,243 |
| Cer(d18:1/17:0)_M+Na | 472510192,8 | 452627010,8 | 533869729,5 | 428251669,0 | 413520812,6 | 462970451,2 | 0,028 |
| Cer(d18:1/18:0)_M+H-H2O | 155546829,5 | 108269108,9 | 229308584,0 | 234827717,8 | 167800236,1 | 343200505,7 | 0,053 |
| Cer(d18:1/18:0)_M+H | 204525688,0 | 139755461,6 | 287762148,0 | 293203389,0 | 211751964,3 | 429861640,2 | 0,065 |
| Cer(d18:1/18:0)_M+Na | 105929028,5 | 71361431,7 | 170683066,3 | 169434345,7 | 113011113,8 | 242579379,3 | 0,530 |
| Cer(d18:1/18:1)_M+H-H2O | 31515227,5 | 22276168,3 | 41454374,6 | 40323014,9 | 33192833,0 | 55622281,8 | 0,790 |
| Cer(d18:1/18:1)_M+H | 42259995,0 | 29228900,2 | 54018199,0 | 51474550,3 | 42873106,0 | 70083823,1 | 0,950 |
| Cer(d18:1/18:1)_M+Na | 13727297,4 | 10397524,1 | 19652700,0 | 20381019,5 | 16212140,2 | 27980453,2 | 0,530 |
| Cer(d18:1/22:0)_M+H | 5683527,1 | 4552780,2 | 6768593,5 | 6435154,7 | 5268847,9 | 7537335,3 | 0,315 |
| Cer(d18:1/24:1)_M+H-H2O | 11693468,2 | 10716413,7 | 12222016,1 | 11340895,3 | 10160310,3 | 14594384,1 | 0,842 |
| Cer(d18:1/24:1)_M+H | 36357324,6 | 33178428,5 | 41372149,7 | 35538814,9 | 30425873,5 | 45651634,7 | 0,905 |
| Total Cer | 1085158032,0 | 909530172,3 | 1268720559,5 | 1295316682,0 | 1058137766,1 | 1646729490,0 | 0,156 |
| DAG(16:0/16:0)_M+H-H2O | 10736321,6 | 9573522,8 | 10983740,7 | 11010645,9 | 10732478,3 | 11761902,5 | 0,133 |
| DAG(16:0/18:1)_M+H-H2O | 34369437,5 | 25179331,9 | 37426884,1 | 36416160,1 | 35404337,3 | 39036513,2 | 0,278 |
| DAG(16:0/18:1)_M+NH4 | 24418835,3 | 22687185,5 | 38088610,0 | 24877887,9 | 23050853,8 | 29022237,1 | 0,968 |
| DAG(16:0/18:2)_M+H-H2O | 9527837,4 | 7639089,0 | 9936245,6 | 9067370,9 | 8695697,7 | 9283687,7 | 0,278 |
| DAG(16:0/20:4)_M+NH4 | 9540873,9 | 8982422,0 | 10195303,7 | 8966810,9 | 7775836,0 | 10584909,9 | 0,400 |
| DAG(16:0/20:4)_M+Na | 8510257,1 | 7500815,5 | 9042639,3 | 7259677,5 | 6731306,1 | 9791665,4 | 0,497 |
| DAG(16:0/22:1)_M+NH4 | 7100480,6 | 5907068,5 | 9387656,6 | 5816268,7 | 5233433,3 | 6250316,4 | 0,053 |
| DAG(16:0/22:6)_M+H-H2O | 35206266,8 | 28504792,4 | 41630032,9 | 40864230,4 | 33888262,0 | 42703175,4 | 0,400 |
| DAG(18:0/18:0)_M+H-H2O | 3066299,5 | 2885714,2 | 3196787,8 | 2967843,8 | 2891885,3 | 3050895,6 | 0,243 |
| DAG(18:0/18:0)_M+NH4 | 7329774,6 | 7038659,2 | 7683267,8 | 7153019,1 | 6788870,2 | 7483542,3 | 0,315 |
| DAG(18:0/18:0)_M+Na | 11432019,4 | 11063729,4 | 12229952,5 | 11479066,2 | 10969403,2 | 11942857,5 | 0,661 |
| DAG(18:0/20:4)_M+H-H2O | 85773424,6 | 57421569,4 | 103555160,6 | 107577297,0 | 82399785,9 | 123683362,3 | 0,243 |
| DAG(18:0/20:4)_M+NH4 | 262024529,4 | 239340707,8 | 270554483,8 | 277220022,1 | 220721575,6 | 317523281,5 | 0,243 |
| DAG(18:0/20:4)_M+Na | 131581947,9 | 116320822,0 | 135780844,5 | 138419351,2 | 113522545,9 | 159208829,3 | 0,243 |
| DAG(18:0/22:4)_M+NH4 | 11165998,4 | 9671163,2 | 14214328,2 | 7859675,2 | 6925820,9 | 9772344,4 | 0,008 |
| DAG(18:0/22:4)_M+Na | 6375192,1 | 5634315,6 | 8000492,4 | 4568349,4 | 3952048,0 | 5597843,3 | 0,008 |
| DAG(18:0/22:6)_M+H-H2O | 117886593,3 | 103794927,7 | 146813934,7 | 141590781,9 | 109429125,8 | 153417476,6 | 0,400 |
| DAG(18:1/16:0)_M+H-H2O | 41611706,8 | 30411615,2 | 43775477,9 | 42595753,4 | 41265371,2 | 44819325,3 | 0,447 |
| DAG(18:1/18:1)_M+H-H2O | 86946227,8 | 68618814,2 | 94590554,9 | 94159290,3 | 86784845,4 | 97594275,6 | 0,353 |
| DAG(18:1/18:1)_M+NH4 | 74489576,8 | 58729120,4 | 98472128,7 | 83129062,9 | 78937900,6 | 92392740,1 | 0,356 |
| DAG(18:1/18:1)_M+Na | 41257068,8 | 30902441,5 | 50135623,7 | 44953836,3 | 41530260,4 | 48558107,1 | 0,447 |
| DAG(18:1/18:2)_M+H-H2O | 14881510,9 | 11211684,0 | 15464197,7 | 14031660,2 | 13881510,6 | 14962440,4 | 0,968 |
| DAG(18:2/18:2)_M+H-H2O | 1517863,3 | 1473176,6 | 1665548,0 | 1449278,0 | 1357541,8 | 1536851,2 | 0,156 |
| Total DAG | 1042248363,0 | 995936440,4 | 1093893177,3 | 1123916652,0 | 1018815885,4 | 1188310432,5 | 0,113 |
| LPC(16:0)_M+H | 298889894,4 | 259043031,6 | 330702205,8 | 315017905,0 | 291577916,1 | 354148844,5 | 0,315 |
| LPC(18:0)_M+H | 79901787,2 | 71407511,5 | 88220218,5 | 85828114,6 | 75712534,6 | 98553938,5 | 0,356 |
| LPC(18:1)_M+H | 109706908,8 | 83294898,0 | 129384278,5 | 89184092,6 | 71920028,1 | 107806197,6 | 0,278 |
| LPC(20:4)_M+H | 49261598,1 | 40192810,1 | 65731503,6 | 53059470,4 | 45560424,6 | 61839423,6 | 0,661 |
| LPC(22:6)_M+H | 36906570,5 | 29238507,3 | 47777331,6 | 29586640,3 | 27322500,7 | 37642486,3 | 0,211 |
| Total LPC | 542633376,7 | 508238807,7 | 634398531,8 | 585837169,7 | 528648400,5 | 631997718,6 | 0,661 |
| LPE(22:6)_M+H | 30010745,1 | 27275700,2 | 37286709,6 | 31588926,1 | 26673917,5 | 32449730,7 | 0,780 |
| MAG(18:2)_M+NH4 | 6133765,0 | 3873892,9 | 7004804,1 | 4559102,3 | 3931991,6 | 5097986,9 | 0,211 |
| MAG(20:1)_M+NH4 | 2025164,7 | 1437152,0 | 2941556,2 | 1956203,1 | 1529961,9 | 5418619,2 | 0,661 |
| MAG(20:2)_M+NH4 | 17879143,6 | 16104152,3 | 21476646,0 | 13893015,6 | 12348549,5 | 16199487,2 | 0,013 |
| MAG(20:4)_M+H-H2O | 3656581,0 | 3239821,0 | 3823636,2 | 4035469,0 | 3100076,3 | 4579295,6 | 0,278 |
| MAG(22:2)_M+NH4 | 10886885,1 | 10040177,7 | 12939095,9 | 10047996,9 | 8594708,5 | 10802395,5 | 0,095 |
| MAG(22:3)_ M+NH4 | 12400072,1 | 10989610,2 | 14849094,4 | 10275215,8 | 6702238,4 | 11050335,7 | 0,017 |
| MAG(22:4)_M+NH4 | 12414995,2 | 11055028,0 | 14812755,7 | 10304465,5 | 6729794,9 | 11063111,5 | 0,013 |
| Total MAG | 64507409,7 | 60276398,9 | 76371115,8 | 54321198,2 | 47664371,7 | 62562555,4 | 0,035 |
| PC(14:0_16:0)_M+H | 125389432,1 | 94002413,4 | 151197754,4 | 128466883,6 | 78895343,5 | 181319508,2 | 0,604 |
| PC(16:0/16:0)_M+H | 8046747614,5 | 5485972054,5 | 12046913979,0 | 9189450451,0 | 7140663308,5 | 13617075020,5 | 0,400 |
| PC(16:0/16:0)_M+Na | 238212919,9 | 161420267,3 | 351626714,4 | 279724376,6 | 224663293,7 | 390052327,8 | 0,278 |
| PC(16:0_18:1)_M+H | 15657158493,0 | 12721379974,8 | 18446999730,3 | 17658755364,0 | 14621434125,5 | 20674736010,5 | 0,211 |
| PC(16:0_18:1)_M+Na | 426779086,8 | 396040757,2 | 535863221,0 | 520015732,3 | 453692852,8 | 599333497,0 | 0,079 |
| PC(16:0_20:4)_M+H | 3538556867,0 | 2906011099,5 | 4661917770,0 | 4419253104,0 | 3684294157,0 | 4630816258,5 | 0,156 |
| PC(16:0_20:4)_M+Na | 75220731,4 | 46104092,1 | 108121028,9 | 98613344,4 | 86240178,4 | 123205347,5 | 0,065 |
| PC(16:0_22:4)_M+H | 268096897,5 | 210152767,8 | 311327217,6 | 311990056,1 | 277186172,7 | 346380751,2 | 0,079 |
| PC(16:0_22:6)_M+H | 4302326469,5 | 3813457490,3 | 4780387172,0 | 4496566310,0 | 3819617459,0 | 4684331467,0 | 0,842 |
| PC(16:0_22:6)_M+Na | 42985823,0 | 32879839,8 | 51796630,2 | 49398256,9 | 42049782,5 | 50387918,2 | 0,356 |
| PC(16:1/22:6)_M+H | 27412700,3 | 25223445,0 | 33594109,7 | 30524186,7 | 28683455,1 | 33389575,9 | 0,156 |
| PC(17:0/18:1)_M+Na | 27181697,5 | 20574618,8 | 35773573,6 | 28635555,1 | 24639032,3 | 31753526,4 | 0,905 |
| PC(18:0/22:4)_M+H | 141529337,8 | 110596227,7 | 152770584,7 | 145466157,4 | 138208177,3 | 177134877,4 | 0,278 |
| PC(18:0/22:5)_M+H | 32904324,1 | 29248145,4 | 37442164,1 | 41726505,8 | 29715071,7 | 43194479,7 | 0,447 |
| PC(18:0_18:1)_M+H | 3523863787,0 | 2843758258,8 | 4106228518,3 | 3579183338,0 | 2782526766,0 | 4215305245,0 | 1,000 |
| PC(18:0_18:1)_M+Na | 57782589,2 | 45169880,9 | 70329085,0 | 69374475,9 | 47080385,9 | 85242881,0 | 0,278 |
| PC(18:0_20:3)_M+H | 51270993,0 | 36633346,1 | 56881485,4 | 56810530,8 | 46008544,4 | 66416599,1 | 0,278 |
| PC(18:0_20:4)_M+H | 3422263111,5 | 2474627763,3 | 4081256227,0 | 3935354380,0 | 3552661551,0 | 4745510558,0 | 0,113 |
| PC(18:0_20:4)_M+Na | 85187547,0 | 68318965,5 | 114394697,7 | 111774087,8 | 101701246,1 | 139488687,6 | 0,022 |
| PC(18:0_22:6)_M+H | 1267838673,0 | 1098893472,5 | 1471896233,3 | 1383569954,0 | 1089253211,5 | 1440738029,0 | 0,968 |
| PC(18:0_22:6)_M+Na | 31316907,8 | 26641685,4 | 38608127,8 | 35611451,5 | 29289202,9 | 38702348,5 | 0,315 |
| PC(18:1/16:1)_M+Na | 12058319,9 | 10336196,2 | 13642104,1 | 14145365,7 | 13127496,0 | 15827328,8 | 0,035 |
| PC(18:1/16:1)_M+H | 364111997,3 | 333161052,1 | 443440556,0 | 457696967,0 | 402796524,5 | 510769675,3 | 0,043 |
| PC(18:1/18:1)_M+H | 1161371684,5 | 1019141976,7 | 1276452897,5 | 1347445995,0 | 1140193359,5 | 1434107376,5 | 0,156 |
| PC(18:1/20:4)_M+H | 1361497231,5 | 1121663990,9 | 1762204532,3 | 1691468166,0 | 1527907017,5 | 1778808585,5 | 0,079 |
| PC(18:1/20:4)_M+Na | 43147977,6 | 34961750,1 | 52001010,3 | 53434480,1 | 50356716,2 | 57066490,9 | 0,010 |
| PC(18:2/20:4)_M+H | 29547823,1 | 22559200,0 | 34963761,9 | 37128615,4 | 29731943,8 | 39178106,4 | 0,133 |
| PC(20:1/20:4)_M+H | 67666473,6 | 57928250,9 | 77937549,1 | 81436502,8 | 73097303,5 | 83549867,8 | 0,280 |
| PC(20:1_22:6)_M+H | 28695564,3 | 26605500,4 | 35027614,7 | 27331555,6 | 25386776,5 | 31750346,8 | 0,400 |
| PC(20:4/20:4)_M+H | 40752702,1 | 36482526,6 | 44949374,0 | 44727195,5 | 41350109,4 | 48683692,0 | 0,113 |
| PC(20:4_22:6)_M+H | 97249719,1 | 90429651,9 | 101406942,2 | 97529857,9 | 92767731,9 | 105470594,7 | 0,661 |
| PC(22:6/22:6)_M+H | 83361188,9 | 75788325,4 | 100339944,5 | 70841360,9 | 63931468,5 | 82093071,9 | 0,065 |
| PC(31:0)_M+H | 26832309,2 | 20327130,3 | 35638748,5 | 28383967,2 | 21877318,6 | 39112808,7 | 0,661 |
| PC(33:2)_M+H | 175142062,9 | 151648817,2 | 199664880,7 | 166769702,5 | 136033287,5 | 185181488,2 | 0,497 |
| PC(33:3)_M+H | 6288470,5 | 4968577,7 | 6887172,0 | 6499141,3 | 6174752,1 | 7843662,1 | 0,447 |
| PC(35:4)_M+Na | 205144190,9 | 187690440,0 | 232045426,4 | 231092313,3 | 203207350,4 | 240987735,6 | 0,243 |
| PC(36:4)_M+H | 5529386,0 | 4509196,5 | 7465020,5 | 7578216,7 | 7369717,9 | 8679739,7 | 0,053 |
| PC(36:5)_M+H | 27914365,3 | 22301879,2 | 32252128,5 | 34491046,2 | 31398929,1 | 38157091,8 | 0,022 |
| PC(38:5)_M+H | 25472594,9 | 22202715,7 | 28723080,1 | 27289752,4 | 25907334,7 | 30178936,2 | 0,133 |
| PC(40:7)_M+H | 519631673,0 | 458814451,8 | 601070538,0 | 514740170,7 | 495696303,4 | 530876354,3 | 0,905 |
| PC(40:7)_M+Na | 6683594,6 | 5284774,1 | 7424525,4 | 7009671,1 | 6579050,7 | 7396569,0 | 0,315 |
| Total PC | 46080936179,5 | 36876965085,8 | 54800237013,0 | 50353947954,0 | 43017084823,5 | 61368393022,0 | 0,278 |
| PCo(16:0/16:0)_M+H | 54938408,8 | 34157710,0 | 78227838,9 | 62030679,6 | 46683426,1 | 102489385,0 | 0,447 |
| PCo(18:1/16:0)\|PCp(16:0_18:0)_M+H | 164925752,9 | 120026549,5 | 204838886,8 | 191089335,5 | 144198965,5 | 252617893,2 | 0,356 |
| PCo(35:5)\|PCp(35:4)_M+Na | 351433346,2 | 268673261,1 | 387895408,4 | 376586996,0 | 310418510,0 | 406501681,3 | 0,400 |
| PCo(36:7)\|PCp(36:6)_M+H | 59347286,4 | 47971201,1 | 72196161,6 | 57516303,9 | 43355185,2 | 69026953,1 | 0,497 |
| PCo(37:6)\|PCp(37:5)_M+Na | 92297850,7 | 89362532,7 | 97727831,0 | 95402101,2 | 92013741,8 | 102642738,5 | 0,211 |
| PCo(37:6)\|PCp(37:5)_M+H | 23521326,1 | 20663352,9 | 31648842,8 | 28812133,9 | 22275663,9 | 33086677,3 | 0,400 |
| PCo(38:5)\|PCp(38:4)_M+H | 15870923,1 | 12820411,2 | 17297966,9 | 17783185,5 | 14682094,0 | 20771583,6 | 0,243 |
| PCo(38:7)\|PCp(38:6)_M+H | 16542536,5 | 13664848,8 | 18272110,1 | 19740658,0 | 16046745,0 | 21686735,2 | 0,211 |
| PCp(16:0/16:0)\|PCo(16:0_16:1)_M+H | 34084050,1 | 19434138,9 | 49666668,4 | 43087464,9 | 30858580,4 | 69717193,9 | 0,278 |
| PCp(34:6)_M+H | 33248336,8 | 17338903,6 | 59334672,9 | 45516835,5 | 27065105,7 | 66669032,6 | 0,497 |
| PCp(35:5)_M+Na | 7425159,3 | 5719236,4 | 8557580,3 | 8649195,9 | 7896619,0 | 10407781,8 | 0,182 |
| PCp(35:5)\|PEo(38:6)_M+Na | 54891036,1 | 38884274,9 | 82106326,5 | 64491798,9 | 52928469,6 | 93879963,8 | 0,156 |
| PCp(35:5)\|PCo(33:3)_M+H | 341926632,2 | 300202130,6 | 359077781,4 | 330423656,2 | 276615169,5 | 368475662,4 | 0,968 |
| PCp(37:6)_M+Na | 79351510,0 | 75820614,3 | 110736772,0 | 106951621,3 | 88182958,2 | 124526997,5 | 0,053 |
| PCp(38:5)\|\|PCo(38:6)_M+H | 15689321,3 | 13284830,9 | 17428041,5 | 17140265,1 | 14294219,8 | 19205715,0 | 0,447 |
| PCp(39:6)_M+Na | 6619495,9 | 4864131,3 | 8243012,8 | 8078679,7 | 5213475,6 | 10703524,1 | 0,315 |
| Total PCo | 1325886770,5 | 1101001478,0 | 1565707241,8 | 1460282135,0 | 1184415330,5 | 1770687717,5 | 0,315 |
| PE(18:0_22:4)_M+H | 213505049,8 | 162394423,3 | 252580927,6 | 242717250,5 | 203429214,2 | 283533517,1 | 0,278 |
| PE(22:6/16:0)_M+H | 645749338,1 | 536893206,9 | 774132578,9 | 755248729,7 | 625488908,1 | 789082707,0 | 0,400 |
| PE(22:6/16:0)_M+Na | 16507290,2 | 14438196,5 | 17485941,5 | 17757299,3 | 16209241,2 | 18312484,4 | 0,278 |
| PE(22:6/18:0)_M+Na | 44011119,5 | 41279751,8 | 47852175,0 | 46401917,3 | 44542704,0 | 48273854,2 | 0,447 |
| PE(22:6/22:6)_M+H | 36127088,7 | 34503131,4 | 42554741,4 | 33261641,5 | 31941297,4 | 35393400,5 | 0,035 |
| PE(34:1)_M+H | 142820106,0 | 112864744,7 | 163454215,5 | 142717269,8 | 117711762,5 | 168879115,8 | 0,842 |
| PE(36:1)_M+H | 234353925,3 | 179689819,8 | 277196724,4 | 213458021,7 | 175697608,3 | 245886156,5 | 0,400 |
| PE(36:4)_M+H | 171330668,0 | 129995883,7 | 197952797,3 | 195316696,9 | 167137152,9 | 210400076,1 | 0,211 |
| PE(37:4)_M+H | 6079119,2 | 4545643,4 | 6832680,9 | 6355856,5 | 5401481,5 | 7290116,4 | 0,497 |
| PE(38:3)_M+H | 13845716,4 | 10542540,4 | 16078832,8 | 14441709,2 | 12685072,8 | 17551469,6 | 0,356 |
| PE(38:4)_M+H | 64521228,9 | 46221743,3 | 73499170,6 | 74630138,6 | 64180804,3 | 83019295,4 | 0,182 |
| PE(20:4/18:0)_M+H | 1500755473,0 | 1013875769,3 | 1796879388,5 | 1868134442,0 | 1452208048,0 | 2137194744,5 | 0,211 |
| PE(38:4)_M+Na | 43451158,5 | 35912555,4 | 48239986,3 | 50122086,1 | 47145353,0 | 56357714,0 | 0,013 |
| PE(39:6)_M+H | 8518631,1 | 7853196,8 | 10121284,8 | 8877976,1 | 7428890,7 | 9849793,6 | 0,968 |
| PE(40:5)_M+H | 61226393,5 | 53414799,0 | 75582970,9 | 77834844,2 | 63032196,9 | 85157452,1 | 0,211 |
| PE(40:6)_M+Na | 45018254,9 | 42425507,5 | 48930870,8 | 47289456,9 | 45757124,6 | 49200114,5 | 0,447 |
| PE(40:6)_M+H | 2417069968,5 | 2143615945,0 | 3031388169,5 | 2865394633,0 | 2252986309,5 | 3086124730,0 | 0,447 |
| PE(42:10)_M+H | 18434713,7 | 17595888,4 | 20024857,1 | 19491552,0 | 18955183,0 | 20097048,7 | 0,182 |
| PE(44:10)_M+H | 49811718,8 | 46464579,0 | 54479650,0 | 51687969,8 | 48614120,9 | 54732331,1 | 0,549 |
| Total PE | 5825992598,5 | 4611048594,0 | 6792856370,0 | 6550747132,0 | 5409934576,5 | 7394509681,5 | 0,211 |
| PEo(16:1_20:4)\|PEp(36:4)_M+H | 167797231,2 | 119659870,0 | 194667192,9 | 192463016,0 | 164430101,8 | 211774193,4 | 0,133 |
| PEo(16:1_22:4)PEp(38:4)_M+H | 190763870,7 | 126830138,9 | 218430406,6 | 212152239,0 | 178016429,0 | 268953068,5 | 0,182 |
| PEo(16:1_22:6)\|PEp(16:0/22:6)_M+Na | 11820748,2 | 10282875,1 | 13498837,4 | 14852065,2 | 13011264,9 | 16570994,1 | 0,010 |
| PEo(16:1_22:6)\|PEp(16:0/22:6)_M+H | 630099985,4 | 441816946,4 | 720303432,5 | 699983776,7 | 550789475,0 | 815487391,1 | 0,315 |
| PEo(18:1_18:2)\|PEp(18:1/18:1)_M+H | 312028394,5 | 191275925,5 | 430179573,0 | 182264334,9 | 177158983,7 | 233186094,7 | 0,043 |
| PEo(18:1_20:4)\|PEp(18:0/20:4)_M+H | 381092814,5 | 284686915,3 | 443759410,6 | 399611215,5 | 323540868,8 | 473422972,1 | 0,400 |
| PEo(18:1_20:4)_M+Na | 14994129,0 | 12698707,5 | 16926074,0 | 16995917,9 | 14345394,1 | 19498367,1 | 0,133 |
| PEo(18:1_22:6)_M+H | 968360372,2 | 835338582,6 | 1224942442,0 | 1114943958,0 | 804922293,9 | 1321432106,0 | 0,604 |
| PEo(18:1_22:6)_M+Na | 32670212,7 | 31280084,5 | 36982510,1 | 37638433,4 | 31261842,1 | 41075619,0 | 0,400 |
| PEo(18:2_22:4)\|PEp(18:1/22:4)_M+H | 454368908,2 | 314653648,3 | 492218519,6 | 337349113,4 | 304073802,4 | 413672657,8 | 0,156 |
| PEo(18:2_22:6)_M+Na | 6605916,4 | 5993378,5 | 7094091,1 | 8099941,3 | 7143760,3 | 8807300,9 | 0,001 |
| PEo(34:2)\|PEp(34:1)_M+H | 204344008,8 | 150619696,6 | 226788103,3 | 171088556,4 | 153760811,3 | 202385703,8 | 0,356 |
| PEo(36:4)\|PEp(36:3)_M+H | 20361408,9 | 13327308,2 | 22517923,1 | 20544005,7 | 16722955,5 | 27424310,1 | 0,400 |
| PEo(36:5)\|PEp(36:4)_M+Na | 5644853,2 | 4172409,3 | 6131439,1 | 6799734,1 | 6103908,8 | 7129428,5 | 0,002 |
| PEo(37:5)\|PEp(37:4)_M+H | 6096239,5 | 5182525,8 | 7113501,8 | 6435325,6 | 5175005,6 | 6861699,9 | 1,000 |
| PEo(38:4)\|PEp(38:3)_M+H | 23403106,0 | 16293042,2 | 26406669,2 | 24704302,7 | 21141126,0 | 34718299,9 | 0,182 |
| PEo(38:6)\|PEp(38:5)_M+H | 67171184,4 | 49179158,9 | 74932241,9 | 74855831,5 | 58779750,3 | 94285419,4 | 0,243 |
| PEo(40:6)\|PEp(40:5)_M+H | 40493893,2 | 34499623,3 | 48026233,5 | 47666688,8 | 35707272,8 | 57366316,1 | 0,278 |
| PEo(40:7)\|PEp(40:6)_M+H | 15003210,6 | 12344598,2 | 16864636,2 | 17595697,8 | 12283699,8 | 21488445,1 | 0,315 |
| Total PEo | 3432833571,0 | 2911678397,5 | 4025865304,3 | 3753208477,0 | 2850297982,0 | 4228875344,0 | 0,661 |
| PG(34:1)_M+NH4 | 57387568,1 | 51059714,7 | 60058974,8 | 55758137,0 | 51507701,8 | 62559540,5 | 1,000 |
| PG(34:1)_M+Na | 7031684,7 | 5727984,6 | 7826521,8 | 7534851,8 | 6464287,4 | 9454272,2 | 0,182 |
| T.Pg | 1757567115,0 | 1648179986,3 | 1839855416,0 | 1659644179,0 | 1587649840,0 | 1715503316,0 | 0,053 |
| SM(d18:1/17:0)_M+Na | 284235556,2 | 268559074,8 | 322090197,7 | 284275056,0 | 268797364,7 | 301806144,7 | 0,780 |
| SM(d18:1/18:0)_M+H | 629533469,6 | 509778330,6 | 1186172875,5 | 823504569,5 | 569999524,8 | 1381296929,0 | 0,497 |
| SM(d18:1/18:0)_M+Na | 13885644,2 | 11666317,5 | 28762419,9 | 18959432,1 | 13748305,0 | 41793026,7 | 0,182 |
| SM(d38:1)_M+H | 52114073,0 | 33515042,5 | 86491846,4 | 47436859,3 | 34159759,5 | 79258913,4 | 0,968 |
| SM(d42:2)_M+H | 28734942,7 | 16046255,8 | 38864476,4 | 19316349,7 | 12567981,4 | 23327427,6 | 0,065 |
| Total SM | 981646417,7 | 920653494,2 | 1617658491,5 | 1182102107,0 | 918794353,9 | 1799140543,0 | 0,661 |
| TAG(10:0/14:1/18:0)_M+NH4 | 14726362,6 | 14272799,7 | 16037920,6 | 14359670,0 | 13809765,6 | 14996279,0 | 0,182 |
| TAG(10:0/15:0/18:0)_M+NH4 | 12051661,6 | 11147295,0 | 12472374,0 | 11074170,9 | 10799371,9 | 11994995,1 | 0,095 |
| TAG(10:0/16:1/18:0)_M+Na | 13155813,5 | 12522178,0 | 13810053,0 | 12264179,8 | 12000056,5 | 12937788,7 | 0,113 |
| TAG(10:0/16:1/18:0)_M+NH4 | 58365676,8 | 56501051,6 | 61131223,4 | 56030788,0 | 54357476,0 | 58498923,0 | 0,156 |
| TAG(10:0/18:2/18:0)_M+Na | 24764159,3 | 23994832,4 | 26476763,6 | 23977205,1 | 22426540,6 | 25457309,0 | 0,278 |
| TAG(12:0/14:0/18:0)_M+NH4 | 50876542,6 | 49327408,0 | 54217881,6 | 51066339,3 | 48473505,6 | 53248171,6 | 0,968 |
| TAG(12:0/14:0/18:0)_M+Na | 9694900,1 | 8829082,0 | 10358253,0 | 9236104,4 | 8800454,2 | 9853299,0 | 0,356 |
| TAG(12:0/14:0/18:2)_M+NH4 | 19665147,1 | 18880433,8 | 20920793,8 | 18332145,7 | 18041906,3 | 19638582,1 | 0,095 |
| TAG(12:0/15:0/18:0)_M+NH4 | 41540637,9 | 39700352,0 | 42941379,6 | 40662163,1 | 39518000,4 | 41789528,3 | 0,400 |
| TAG(12:0/15:0/18:0)_M+Na | 11404057,7 | 10611822,8 | 11808216,0 | 11012745,1 | 10759027,6 | 11448443,1 | 0,780 |
| TAG(12:0/16:0/20:1)_M+NH4 | 203261658,4 | 186784975,0 | 215393029,0 | 195829905,3 | 189168673,3 | 202989970,2 | 0,661 |
| TAG(12:0/16:0/20:1)_M+Na | 41637830,3 | 39157954,8 | 44908299,9 | 40174983,1 | 38772343,9 | 41887129,7 | 0,243 |
| TAG(12:0/16:1/17:0)_M+Na | 12932432,3 | 12477392,5 | 13850444,6 | 12640989,8 | 12057611,9 | 13063019,4 | 0,243 |
| TAG(14:0/16:1/15:0)_M+Na | 10089338,1 | 9657346,1 | 10439565,8 | 9512009,8 | 9010893,0 | 10203276,4 | 0,156 |
| TAG(12:0/16:1/17:0)_M+NH4 | 60528869,0 | 58561244,7 | 64138582,6 | 59123737,3 | 56424047,2 | 60485326,5 | 0,156 |
| TAG(12:0/16:1/18:0)_M+Na | 47894721,4 | 46079354,8 | 50543938,9 | 45116050,4 | 43713373,2 | 48162828,2 | 0,182 |
| TAG(12:0/18:2/12:0)_M+NH4 | 2269403,0 | 2137792,4 | 2458201,4 | 2272890,5 | 2158601,9 | 2295054,3 | 0,661 |
| TAG(12:0/20:0/22:1)_M+NH4 | 26431243,8 | 22459869,4 | 28929736,2 | 28940949,4 | 26956288,1 | 31118163,5 | 0,095 |
| TAG(12:0/20:0/22:1)_M+Na | 6534528,4 | 5481537,4 | 7154325,0 | 7134425,6 | 6479279,4 | 7430350,3 | 0,133 |
| TAG(14:0/14:0/14:0)_M+Na | 4844540,1 | 4546550,1 | 5221318,0 | 4696373,4 | 4509085,1 | 4822822,2 | 0,182 |
| TAG(14:0/14:0/14:0)_M+NH4 | 16176822,1 | 15471191,1 | 17129488,5 | 15629253,3 | 15164652,6 | 16336098,1 | 0,278 |
| TAG(14:0/14:0/18:0)_M+NH4 | 102589941,6 | 93288960,2 | 107749463,5 | 101223983,8 | 95874592,3 | 103263281,5 | 0,720 |
| TAG(14:0/14:0/18:2)_M+NH4 | 110779187,7 | 107637999,9 | 117567624,4 | 105294782,7 | 101803306,9 | 111137277,8 | 0,156 |
| TAG(14:0/14:0/24:0)_M+NH4 | 21327576,2 | 20252852,7 | 22505553,7 | 22332097,0 | 16706108,1 | 24202795,8 | 0,780 |
| TAG(14:0/15:0/18:1)_M+Na | 36389949,8 | 33741473,6 | 37157633,4 | 34114320,3 | 32862322,6 | 36006048,1 | 0,113 |
| TAG(14:0/15:0/18:1)_M+NH4 | 139681749,7 | 133219109,7 | 145397014,1 | 136200175,9 | 130704162,6 | 140909761,9 | 0,497 |
| TAG(14:0/15:0/18:2)_M+NH4 | 104798933,6 | 101680204,3 | 109954610,7 | 99647478,2 | 96503881,6 | 104911274,5 | 0,156 |
| TAG(14:0/15:0/20:0)_M+NH4 | 49301306,4 | 47242588,5 | 54148098,9 | 50472050,5 | 46029386,6 | 55297868,8 | 0,780 |
| TAG(14:0/15:0/20:0)_ | 12319759,7 | 11781998,5 | 13082653,0 | 12771559,5 | 11070115,5 | 13912276,5 | 0,549 |
| TAG(14:0/15:0/20:1)_M+Na | 27883929,9 | 25090929,6 | 29496914,3 | 26326542,5 | 25885524,1 | 28120586,0 | 0,604 |
| TAG(14:0/15:0/20:1)_M+NH4 | 104592708,2 | 94387314,0 | 109819180,3 | 99368243,2 | 98252094,6 | 104213596,7 | 0,549 |
| TAG(14:0/15:0/20:2)_M+Na | 33802532,2 | 31992654,4 | 35530117,0 | 32600582,5 | 30744436,6 | 33928792,9 | 0,211 |
| TAG(14:0/15:0/20:2)_M+NH4 | 118939994,8 | 115167919,5 | 124878387,5 | 117277837,5 | 111990176,1 | 121283137,3 | 0,497 |
| TAG(14:0/15:0/20:3)_M+NH4 | 47973584,7 | 46669594,5 | 50594891,0 | 45543908,8 | 44307252,2 | 48015882,0 | 0,211 |
| TAG(14:0/16:0/20:0)_M+NH4 | 49936951,9 | 48324665,6 | 53288979,4 | 50649035,7 | 46193996,6 | 56758618,4 | 0,905 |
| TAG(14:0/16:0/20:1)_M+Na | 66159489,4 | 56088178,3 | 68356676,0 | 65485306,8 | 63912679,2 | 66681994,1 | 0,968 |
| TAG(14:0/18:1/18:3)_M+NH4 | 10145813,3 | 9496570,8 | 11744042,5 | 9470370,9 | 9286742,0 | 9612341,5 | 0,035 |
| TAG(14:0/24:0/18:0)_M+NH4 | 6547035,7 | 6130719,4 | 6918020,2 | 6338132,9 | 5987117,3 | 6401104,8 | 0,113 |
| TAG(14:0/24:0/20:1)_M+NH4 | 7456860,1 | 7256828,1 | 7978969,9 | 7304370,3 | 6844868,0 | 7419244,2 | 0,156 |
| TAG(14:1/15:0/22:0)_M+NH4 | 36502506,4 | 32933786,5 | 38766732,8 | 36948434,7 | 36231182,3 | 39178711,6 | 0,356 |
| TAG(14:1/16:1/15:0)_M+NH4 | 19390525,4 | 18737730,8 | 20519159,8 | 18350830,9 | 18157621,7 | 19546824,9 | 0,133 |
| TAG(14:1/18:2/15:0)_M+NH4 | 12888491,1 | 12477029,4 | 13756900,9 | 12042534,6 | 11739203,3 | 12751770,3 | 0,095 |
| TAG(15:0/16:0/22:3)_M+NH4 | 24560754,9 | 21530244,2 | 25886057,8 | 24076971,5 | 23937258,2 | 24730774,3 | 0,842 |
| TAG(15:0/16:0/24:0)_M+NH4 | 7571988,6 | 7258369,7 | 8314860,2 | 7346524,1 | 6932040,8 | 7501492,9 | 0,079 |
| TAG(15:0/16:0/26:0)_M+NH4 | 4252190,1 | 3965643,0 | 4522601,1 | 4079198,0 | 3807261,0 | 4160645,6 | 0,053 |
| TAG(15:0/16:1/26:0)_M+NH4 | 8920300,4 | 8544481,0 | 9296265,4 | 8488940,0 | 8233430,5 | 8635267,7 | 0,053 |
| TAG(15:0/18:0/20:2)_M+NH4 | 25337888,3 | 22189367,1 | 27316165,7 | 25689665,9 | 25110001,7 | 27317180,5 | 0,447 |
| TAG(15:0/18:2/20:1)_M+NH4 | 24071245,6 | 21132007,3 | 25507665,7 | 23619939,3 | 23484152,6 | 24355079,5 | 1,000 |
| TAG(16:0/16:0/16:0)_M+NH4 | 101235964,4 | 96504190,4 | 106466533,3 | 103361745,6 | 100891712,3 | 107264619,2 | 0,604 |
| TAG(16:0/16:0/18:1)_M+Na | 65976948,4 | 56068278,5 | 68315653,5 | 65392481,5 | 63865107,4 | 66570524,7 | 0,968 |
| TAG(16:0/16:0/18:1)_M+NH4 | 307066597,5 | 237870032,1 | 315548726,9 | 311531154,8 | 297364698,3 | 324496320,7 | 0,278 |
| TAG(16:0/16:0/18:2)_M+NH4 | 276768456,0 | 245121587,6 | 289122450,2 | 265484415,5 | 261045094,5 | 268936836,0 | 0,356 |
| TAG(16:0/16:0/18:2)_M+Na | 72949531,6 | 65661937,6 | 76671639,4 | 68653715,3 | 67461805,0 | 70896760,7 | 0,356 |
| TAG(16:0/16:0/20:1)_M+NH4 | 114810349,1 | 92060127,7 | 132024105,2 | 133348823,0 | 122638186,7 | 135677990,9 | 0,065 |
| TAG(16:0/16:0/20:1)_M+Na | 8685726,7 | 6573377,1 | 9773036,4 | 9589654,7 | 9070658,1 | 10456988,6 | 0,053 |
| TAG(16:0/16:0/20:2)_M+NH4 | 1045320946,5 | 769179249,8 | 1168917557,3 | 1158179082,0 | 1092756849,0 | 1203748763,5 | 0,156 |
| TAG(16:0/16:0/20:2)_M+Na | 224405753,8 | 173946514,5 | 236950691,6 | 229042032,7 | 221359974,2 | 240871526,9 | 0,400 |
| TAG(16:0/16:0/20:3)_M+NH4 | 483742888,7 | 374199684,5 | 498382525,7 | 455917549,4 | 448736710,7 | 467235242,3 | 0,447 |
| TAG(16:0/16:0/20:3)_M+Na | 123391143,7 | 97532448,7 | 127582289,1 | 113557899,8 | 111959806,4 | 117881783,0 | 0,400 |
| TAG(16:0/16:1/18:2)_M+NH4 | 129713944,8 | 125881527,8 | 138992623,2 | 125289537,8 | 123096654,5 | 127366475,8 | 0,028 |
| TAG(16:0/16:1/18:2)_M+Na | 26008132,4 | 24564717,1 | 28097867,3 | 24662274,9 | 24277366,0 | 25066603,7 | 0,035 |
| TAG(16:0/18:1/22:1)_M+Na | 5472799,2 | 4182156,2 | 6707595,3 | 6385262,7 | 5842834,9 | 6993868,9 | 0,156 |
| TAG(16:0/18:1/22:1)_M+NH4 | 24134769,1 | 18491284,4 | 28334980,7 | 28624564,2 | 25927184,6 | 30549761,4 | 0,035 |
| TAG(16:0/18:1/24:1)_M+NH4 | 11164735,3 | 10655144,8 | 12093062,4 | 11343359,2 | 11124411,6 | 12048822,0 | 0,400 |
| TAG(16:1/15:0/20:1)_M+NH4 | 70253966,4 | 64477388,5 | 73928651,4 | 67785523,0 | 66599711,3 | 71208245,2 | 0,720 |
| TAG(16:1/15:0/20:1)_M+Na | 18103436,9 | 16523543,2 | 18980933,8 | 17376183,9 | 16744153,9 | 18409344,6 | 0,549 |
| TAG(16:1/16:0/16:1)_M+NH4 | 200641469,6 | 193188151,8 | 213752992,4 | 193541441,1 | 187118713,5 | 201277034,9 | 0,211 |
| TAG(16:1/16:1/16:1)_M+Na | 14178601,8 | 13706182,6 | 15005716,6 | 13112317,7 | 12365066,1 | 13906697,1 | 0,004 |
| TAG(16:1/16:1/20:2)_M+NH4 | 146318813,5 | 136351839,0 | 157336166,8 | 143316249,8 | 140062427,0 | 145477992,3 | 0,400 |
| TAG(16:1/16:1/20:2)_M+Na | 37654957,9 | 34513786,8 | 40317265,2 | 36917754,2 | 35752512,0 | 37263929,5 | 0,182 |
| TAG(17:0/17:0/17:0)_M+Na | 677712871,4 | 559836659,0 | 693956134,2 | 660528267,3 | 378103267,0 | 808440169,5 | 0,842 |
| TAG(17:0/18:1/18:3)_M+NH4 | 5234579,6 | 5079162,6 | 5822063,9 | 5279159,7 | 5203351,5 | 5564437,9 | 0,661 |
| TAG(18:1/18:0/18:1)_M+NH4 | 244838036,7 | 172270258,2 | 298563936,6 | 301283301,4 | 272102821,6 | 307187102,5 | 0,053 |
| TAG(18:1/18:0/18:1)_M+Na | 24245855,2 | 16042982,9 | 28825698,3 | 27604367,5 | 25524020,4 | 30099032,2 | 0,113 |
| TAG(18:1/18:1/18:1)_M+NH4 | 1718273843,5 | 1236877505,3 | 1919424630,5 | 1887422662,0 | 1799099727,0 | 1983625061,5 | 0,133 |
| TAG(18:1/18:1/18:1)_M+Na | 350904641,7 | 275206263,1 | 383498107,2 | 364791294,5 | 352828746,3 | 386090910,9 | 0,315 |
| TAG(18:1/18:1/18:2)_M+NH4 | 583673583,0 | 447305110,6 | 617179569,3 | 568144281,8 | 562719322,7 | 586437707,4 | 0,905 |
| TAG(18:1/18:1/18:2)_M+Na | 143316930,2 | 111000365,4 | 152945653,6 | 137209923,0 | 134582650,5 | 143533147,5 | 0,720 |
| TAG(18:1/18:1/18:3)_M+Na | 57514565,9 | 51441759,9 | 61395408,1 | 55375795,7 | 54737943,9 | 57318384,8 | 0,400 |
| TAG(18:1/18:1/18:3)_M+NH4 | 216085842,7 | 196150170,4 | 228217413,7 | 212739687,4 | 208679909,4 | 218072682,1 | 0,842 |
| TAG(18:1/20:1/18:1)_M+NH4 | 29603540,3 | 21356910,2 | 34519644,7 | 34234111,6 | 31296430,5 | 35336120,4 | 0,095 |
| TAG(18:1/20:1/18:1)_M+Na | 1704745,7 | 1092577,0 | 2004007,2 | 1898121,4 | 1742142,0 | 2085971,7 | 0,156 |
| TAG(18:1/20:1/18:1)\|TAG(18:2/20:1/18:0)_M+NH4 | 31253187,1 | 22627937,5 | 36126586,1 | 35407748,5 | 32601791,7 | 36756876,4 | 0,133 |
| TAG(18:1/20:1/18:2)_M+NH4 | 14495360,9 | 11020739,4 | 15916213,5 | 15267113,2 | 14105204,4 | 16607713,2 | 0,400 |
| TAG(18:2/16:1/18:2)_M+Na | 2707508,8 | 2593157,3 | 3293592,3 | 2622604,8 | 2574491,0 | 2767188,1 | 0,278 |
| TAG(18:2/18:1/15:0)_M+NH4 | 21030776,7 | 19166890,4 | 21962399,5 | 20521234,2 | 19783848,5 | 21316809,0 | 1,000 |
| TAG(18:2/18:1/15:0)_M+Na | 6764306,0 | 6466344,1 | 7052345,4 | 6612862,6 | 6342384,0 | 6752210,4 | 0,182 |
| TAG(24:0/18:2/17:0)_M+NH4 | 3783706,0 | 3619342,3 | 3941354,9 | 3714018,4 | 3573056,8 | 3825477,2 | 0,315 |
| Total TAG | 9728278814,0 | 7936683686,0 | 10041020401,8 | 9642835814,0 | 9391165594,0 | 10124871613,5 | 0,661 |

**Table S4.** Lipid variables by mouse genotype with median, interquartile and p-value in negative ionization mode in the amygdala.

| **Lipid variables** | **WT** | | | **TG** | | | **p value (Mann-Whitney)** |
| --- | --- | --- | --- | --- | --- | --- | --- |
|  | **Median** | **IQ1** | **IQ3** | **Median** | **IQ1** | **IQ3** |  |
| Cer(d18:1/16:0)_M-H | 46699467,0 | 31031136,6 | 58310759,4 | 67141062,9 | 54123281,0 | 74621358,2 | 0,017 |
| Cer(d18:1/16:0)_M+CH3COO | 1364417787,0 | 1194837382,0 | 1504149874,3 | 1205530332,0 | 985123481,8 | 1274222924,0 | 0,043 |
| Cer(d18:0_18:1)_M-H | 489715835,2 | 220906950,2 | 642028024,8 | 813921650,2 | 545889016,0 | 1040505448,7 | 0,028 |
| Cer(d18:0_18:1)_M+CH3COO | 742057297,3 | 333392053,5 | 967474450,4 | 1229526491,0 | 815995437,0 | 1566118425,5 | 0,022 |
| Cer(d18:1/20:0)_M-H | 50108798,6 | 33852621,4 | 64709948,0 | 63501117,5 | 48470923,2 | 80101527,6 | 0,356 |
| Cer(d18:1/20:0)_M+CH3COO | 88299623,2 | 59325148,8 | 114286465,8 | 111903456,5 | 84573667,9 | 139124399,9 | 0,356 |
| Cer(d18:1/24:0)_M-H | 9766408,4 | 7053480,2 | 12533502,4 | 10083806,0 | 8919289,6 | 13787345,2 | 0,400 |
| Cer(d18:1/24:0)_M+CH3COO | 23795309,5 | 16960405,2 | 29817890,1 | 24057783,7 | 20820974,1 | 32034418,9 | 0,497 |
| Cer(d18:1/24:1)_M-H | 75681166,5 | 67263147,2 | 83106893,0 | 75409664,5 | 68829497,3 | 82695312,6 | 0,720 |
| Cer(d18:1/24:1)_M+CH3COO | 168785677,9 | 149243424,5 | 186684483,8 | 166794379,1 | 153111290,7 | 180890784,2 | 0,905 |
| Total Cer | 3171508143,0 | 2279609194,5 | 3703661159,0 | 3853756642,0 | 3033448147,0 | 4284621193,5 | 0,182 |
| CL(16:1_18:1_20:4_20:4)_M-H | 17853431,3 | 14066993,5 | 24917026,3 | 23006959,7 | 16376947,8 | 28923555,7 | 0,604 |
| CL(16:1_18:1_20:4_22:6)_M-H | 11761620,8 | 9730026,1 | 16418236,8 | 13910850,5 | 10030685,4 | 17443306,7 | 0,905 |
| CL(18:1/18:1/18:1/18:1)_M-H | 32396968,8 | 20256221,3 | 39329321,6 | 26590377,0 | 18383388,4 | 36262817,8 | 0,400 |
| CL(18:1_18:1_18:1_18:2)_M-H | 17372919,7 | 12629137,5 | 27080175,5 | 21746198,5 | 14405973,5 | 27236997,0 | 0,905 |
| CL(18:1_18:1_18:1_20:4)_M-H | 38955751,8 | 28340597,0 | 58764888,3 | 47832903,9 | 32863777,0 | 60499574,4 | 0,905 |
| CL(18:1_18:1_18:1_22:6)_M-H | 29127838,2 | 21191017,2 | 39065319,4 | 32879186,3 | 22682663,9 | 39537830,6 | 0,905 |
| CL(18:1_18:1_20:4_20:4)_M-H | 37117469,9 | 26925515,1 | 53071220,6 | 48097620,6 | 33233726,3 | 54845978,3 | 0,549 |
| CL(18:1_18:1_20:4_22:6)_M-H | 31021322,8 | 25948513,9 | 44081975,3 | 36589845,4 | 26696602,1 | 42941479,7 | 1,000 |
| CL(18:1_18:1_22:6_22:6)_M-H | 16328992,4 | 12470763,8 | 19616181,7 | 15774068,9 | 11961961,6 | 18135158,2 | 0,604 |
| CL(18:1_18:2_22:6_22:6)_M-H | 12954231,2 | 10902882,8 | 18853433,8 | 14756966,0 | 11663186,0 | 17116940,0 | 1,000 |
| CL(18:1_20:4_22:6_22:6)_M-H | 7927056,8 | 7032371,4 | 10724448,5 | 7644988,8 | 6582154,8 | 8627830,0 | 0,356 |
| CL(18:2/18:2/18:2/18:2)_M-H | 6999200,6 | 5134999,2 | 9798343,2 | 8984366,0 | 6382815,0 | 12440441,3 | 0,315 |
| Total CL | 246290561,2 | 195935447,0 | 358464970,9 | 304844638,4 | 212544311,7 | 367153140,8 | 0,905 |
| FA(16:0)_M-H | 2274857248,0 | 2181878913,8 | 2444199292,5 | 2390583923,0 | 2206214457,5 | 2758346658,5 | 0,211 |
| FA(17:0)_M-H | 220363176,3 | 206143572,1 | 261328439,5 | 213069280,7 | 206758123,3 | 224864789,5 | 0,356 |
| FA(18:0)_M-H | 3562816219,5 | 3312767188,0 | 3676663971,0 | 3846228194,0 | 3537600335,0 | 4289900297,5 | 0,079 |
| FA(18:1)_M-H | 3686779167,5 | 3079711473,3 | 4677801000,3 | 3326079766,0 | 3110113807,0 | 4335786316,5 | 0,604 |
| FA(18:2)_M-H | 186959631,1 | 157487905,0 | 197833030,3 | 164696587,0 | 154710440,0 | 192196756,2 | 0,661 |
| FA(20:0)_M-H | 60659105,8 | 48455597,2 | 64358840,6 | 60399370,0 | 56440560,9 | 68868954,5 | 0,400 |
| FA(20:1)_M-H | 277507783,9 | 208098508,9 | 409478323,0 | 229281575,1 | 201789923,3 | 337058861,4 | 0,549 |
| FA(20:4)_M-H | 8867819873,5 | 8266275612,3 | 9788611092,3 | 8826048355,0 | 8527191785,0 | 10268374293,0 | 1,000 |
| FA(22:0)_M-H | 47914611,4 | 39721462,2 | 51288629,9 | 47686884,8 | 44116062,9 | 50389805,5 | 1,000 |
| FA(22:1)_M-H | 37741306,4 | 26426220,9 | 52621398,1 | 25971569,5 | 21627236,8 | 34990970,5 | 0,035 |
| FA(24:0)_M-H | 74118543,5 | 58376385,8 | 76518605,1 | 67272985,9 | 62514627,3 | 73900700,5 | 0,549 |
| FA(24:1)_M-H | 20514953,0 | 14988434,5 | 30546854,8 | 17787054,5 | 12734209,8 | 21540475,3 | 0,156 |
| FA(26:0)_M-H | 46308747,8 | 39021024,5 | 51685971,1 | 46484022,7 | 40002487,1 | 50775607,8 | 1,000 |
| Total FA | 19467071698,0 | 17674293347,8 | 21781700689,5 | 19874938843,0 | 18193317000,5 | 21597516473,5 | 0,780 |
| LPC(16:0)_M-CH3 | 46342791,4 | 44094490,5 | 53928619,0 | 52500035,1 | 49748874,0 | 55977676,4 | 0,113 |
| LPC(16:0)_M+CH3COO | 186182602,0 | 176057042,1 | 214911639,2 | 207546564,4 | 197083522,0 | 219824475,6 | 0,113 |
| LPC(16:1)_M-CH3 | 24726441,2 | 21081743,0 | 29664091,6 | 27074015,7 | 23359501,5 | 29017688,5 | 0,780 |
| LPC(18:0)_M-CH3 | 13549865,3 | 12067039,6 | 15152654,3 | 14745119,1 | 14125943,9 | 15301188,9 | 0,156 |
| LPC(18:0)_M+CH3COO | 63655505,7 | 59785073,3 | 70869935,1 | 69766142,6 | 65113740,9 | 71802111,9 | 0,278 |
| LPC(18:1)_M-CH3 | 12096794,5 | 11051168,1 | 16850090,0 | 13729858,9 | 11732852,0 | 14569763,2 | 0,780 |
| LPC(18:1)_M+CH3COO | 59204490,5 | 51824916,0 | 78664698,6 | 64478115,9 | 54529419,7 | 66950866,2 | 0,780 |
| LPC(20:1)_M+CH3COO | 2376780,2 | 2152113,9 | 3502695,8 | 2586221,0 | 2044937,7 | 3063863,9 | 0,604 |
| LPC(20:4)_M+CH3COO | 34094299,7 | 28213300,0 | 51716524,6 | 33443599,0 | 29446329,7 | 46551874,2 | 0,968 |
| LPC(22:6)_M+CH3COO | 25563537,3 | 17341556,7 | 32852874,7 | 17765046,7 | 15168658,9 | 25375043,2 | 0,156 |
| Total LPC | 465170645,4 | 426043475,0 | 546599994,5 | 500402713,1 | 468300496,6 | 541129634,9 | 0,356 |
| LPE(18:0)_M-H | 52354045,5 | 42589342,6 | 66107697,7 | 75340767,9 | 61110559,9 | 79678452,2 | 0,022 |
| LPE(20:4)_M-H | 54047226,4 | 47572268,6 | 81999869,0 | 59323253,3 | 52568250,5 | 73175748,2 | 0,604 |
| LPE(22:6)_M-H | 133728991,3 | 111064590,2 | 189134147,7 | 115993549,5 | 103429423,5 | 146322634,3 | 0,497 |
| Total LPE | 233133427,4 | 218324074,0 | 336664107,9 | 254401974,0 | 235113630,6 | 284473023,3 | 0,497 |
| LPI(18:0)_M-H | 62531906,2 | 59798636,9 | 84079295,8 | 72964565,2 | 61861629,3 | 80712761,7 | 0,549 |
| LPI(20:4)_M-H | 35141496,5 | 29726013,7 | 57307309,9 | 41004374,7 | 31153380,8 | 50831221,4 | 0,842 |
| Total LPI | 102207775,9 | 89487057,8 | 130065486,6 | 113715525,9 | 93406011,7 | 130991252,1 | 0,720 |
| LPS(18:0)_M-H | 10719791,4 | 10270972,0 | 12689390,0 | 11135528,9 | 9225987,0 | 11827467,2 | 0,842 |
| LPS(22:6)_M-H | 74527518,7 | 67697020,5 | 119326486,4 | 90584124,5 | 67161568,3 | 101409376,0 | 1,000 |
| Total LPS | 84782034,7 | 79556277,5 | 131172738,9 | 102517903,5 | 76636004,0 | 112792270,2 | 1,000 |
| PC(16:0/16:0)_M-CH3 | 141377906,8 | 94592434,2 | 181034755,9 | 142355497,0 | 132093311,1 | 181164328,6 | 0,780 |
| PC(16:0/16:0)_M+CH3COO-CH3 | 54574551,2 | 43417498,5 | 72372517,3 | 47479233,6 | 40430771,8 | 55883848,3 | 0,447 |
| PC(16:0_20:4)_M-CH3 | 83637085,1 | 66018030,9 | 85153153,5 | 81636613,0 | 78171224,0 | 87337997,8 | 0,604 |
| PC(16:0_20:4)_M+CH3COO | 981047557,6 | 767161942,3 | 1012925815,5 | 970944006,6 | 909073022,8 | 1007293211,5 | 0,720 |
| PC(16:0/22:4)_M+CH3COO | 69462534,7 | 60648940,0 | 81544319,5 | 79006632,1 | 75790199,6 | 81312905,8 | 0,182 |
| PC(18:0/16:1)_M+CH3COO | 63387353,6 | 62116951,6 | 66937934,3 | 68805117,5 | 65990052,0 | 74012517,5 | 0,035 |
| PC(16:0_18:1)_M+CH3COO // M+CH3COO | 62936526,4 | 61394562,9 | 66664552,9 | 68445567,4 | 65763789,8 | 73566020,9 | 0,043 |
| PC(16:1/22:6)_M+CH3COO | 8895789,6 | 7503321,7 | 10242490,3 | 9312464,9 | 8667878,7 | 10453215,8 | 0,549 |
| PC(17:0/16:0)_M+CH3COO | 13345024,6 | 11267257,9 | 17827147,8 | 14547157,6 | 11732382,2 | 18834083,2 | 0,497 |
| PC(17:0/18:1)_M+CH3COO | 17926472,3 | 15236855,6 | 19156017,4 | 15568293,6 | 14303509,9 | 16722471,5 | 0,065 |
| PC(18:0/18:1)_M-CH3 | 86465930,3 | 78337570,0 | 94615009,9 | 74545051,8 | 71319957,6 | 86546309,0 | 0,113 |
| PC(18:0/18:1)_M+CH3COO | 1021169054,6 | 931353564,1 | 1110471273,8 | 879932436,0 | 845300544,5 | 1025819422,5 | 0,095 |
| PC(18:0/20:4)_M-CH3 | 68617236,1 | 65196264,6 | 71770920,8 | 71119143,6 | 67977543,4 | 83362374,8 | 0,243 |
| PC(18:0/20:4)_M+CH3COO | 810456663,0 | 696437343,5 | 848022827,6 | 835691276,4 | 798246154,7 | 966788633,9 | 0,211 |
| PC(18:0/22:4)_M+CH3COO | 36319138,8 | 32273069,1 | 39749422,7 | 40013088,6 | 37258474,8 | 43227662,2 | 0,133 |
| PC(18:0/22:5)_M+CH3COO | 32653613,7 | 29780396,4 | 39194727,2 | 34505157,9 | 30768491,1 | 42770784,6 | 0,549 |
| PC(18:0/22:6)_M+CH3COO | 194968793,5 | 187283204,6 | 244325954,2 | 191432273,0 | 169727714,7 | 197772495,2 | 0,133 |
| PC(18:1/18:1)_M-CH3 | 20092489,0 | 18715304,6 | 21394581,6 | 19998119,1 | 18969106,7 | 25373357,3 | 1,000 |
| PC(18:1/18:1)_M+CH3COO | 310102477,9 | 275759388,3 | 324522486,7 | 292115648,2 | 275291669,2 | 352177529,7 | 0,842 |
| PC(18:1/20:4)_M+CH3COO | 188305891,1 | 124682305,8 | 202491413,5 | 178552072,7 | 168534890,6 | 191958411,0 | 1,000 |
| PC(18:1_22:0)_M+CH3COO | 35125787,2 | 17849989,7 | 42564148,0 | 20572290,4 | 18059503,1 | 24253824,0 | 0,133 |
| PC(22:6/22:6)_M+CH3COO | 28443721,1 | 26048058,7 | 43176725,3 | 22318613,1 | 18639710,1 | 30888123,7 | 0,350 |
| PC(30:0)_M-CH3 | 2343473,1 | 1565596,1 | 2739128,1 | 2273350,4 | 1758229,8 | 2835339,9 | 0,842 |
| PC(30:0)_M+CH3COO | 26835583,6 | 16658572,3 | 31576752,0 | 24779207,2 | 18608408,1 | 32036982,5 | 0,842 |
| PC(32:0)_M+CH3COO | 1519263632,0 | 1029228143,6 | 1924520866,8 | 1503393550,0 | 1403651925,0 | 1929788902,5 | 0,780 |
| PC(32:1)_M+CH3COO | 126797387,2 | 108496360,0 | 144690046,4 | 124639899,8 | 120918702,9 | 142956445,3 | 0,549 |
| PC(33:1)_M+CH3COO | 15177375,6 | 14123493,6 | 16127631,5 | 15050896,1 | 13682283,2 | 15684192,0 | 0,497 |
| PC(34:1)_M-CH3 | 241478583,9 | 220403303,3 | 269384458,9 | 223602156,4 | 211641600,7 | 250714283,3 | 0,447 |
| PC(34:1)_M+CH3COO | 2675229394,5 | 2452160401,5 | 2970502491,3 | 2458712459,0 | 2325552362,5 | 2776933123,0 | 0,315 |
| PC(34:2)_M+CH3COO | 45945380,8 | 34475528,1 | 55874271,4 | 42762302,6 | 41605634,0 | 51493562,6 | 0,968 |
| PC(34:5)_M-CH3 | 10834617,8 | 10122343,4 | 11773606,5 | 11823607,4 | 10981505,1 | 12922532,6 | 0,156 |
| PC(36:2)_M+CH3COO | 41845592,2 | 39579828,8 | 45190068,1 | 40122922,9 | 38065711,6 | 43135854,1 | 0,315 |
| PC(36:2)_M-CH3 | 29460099,5 | 16494964,4 | 50814396,2 | 20895300,9 | 17223639,5 | 28799709,2 | 0,447 |
| PC(36:3)_M+CH3COO | 14554756,8 | 12803887,3 | 17293138,8 | 16630854,1 | 16058128,8 | 18499688,3 | 0,211 |
| PC(16:0_20:3)_M+CH3COO | 43381697,3 | 37900087,1 | 48469974,5 | 49650382,1 | 44312670,4 | 52817472,7 | 0,133 |
| PC(37:4)_M+CH3COO | 9167754,6 | 7885023,4 | 10195230,8 | 9263736,1 | 9106084,2 | 10046993,8 | 0,400 |
| PC(38:2)_M+CH3COO | 21291521,9 | 19566362,6 | 31458733,4 | 17977820,4 | 17037890,3 | 20727303,7 | 0,043 |
| PC(38:3)_M+CH3COO | 14156413,2 | 12946995,0 | 14656084,3 | 14466799,9 | 13429473,3 | 15498641,7 | 0,447 |
| PC(38:6)_M+CH3COO | 522848103,5 | 466414772,5 | 583198289,9 | 488459739,0 | 473761905,3 | 506655014,5 | 0,133 |
| PC(40:5)_M+CH3COO | 8945174,1 | 8278212,8 | 9471860,8 | 9662821,4 | 8649051,7 | 10235898,7 | 0,243 |
| PC(40:6)_M-CH3 | 13928268,7 | 12910707,5 | 16689973,2 | 13063871,2 | 11629847,6 | 14336203,8 | 0,211 |
| PC(40:7)_M+CH3COO | 187703536,3 | 172017757,0 | 221550854,4 | 181249226,7 | 164619032,6 | 189961697,9 | 0,356 |
| PC(42:10)_M+CH3COO | 42651949,8 | 39360529,4 | 48050695,0 | 45376019,4 | 39483289,7 | 52380068,6 | 1,000 |
| PC(42:7)_M+CH3COO | 4808899,3 | 4497299,3 | 7335677,2 | 4148374,5 | 3407785,3 | 4996280,2 | 0,043 |
| PC(42:8)_M+CH3COO | 4514189,8 | 3684072,7 | 5856767,4 | 5214623,1 | 4964935,1 | 6307690,0 | 0,156 |
| PC(44:10)_M+CH3COO | 1934521,1 | 1710657,5 | 2247690,1 | 2179979,9 | 1817609,9 | 2372748,1 | 0,400 |
| Total PC | 9962162907,5 | 9098955780,5 | 10920118119,3 | 9735054461,0 | 9269105790,0 | 10336228974,0 | 0,661 |
| PCo(34:2)\|PCp(34:1)_M+CH3COO | 7911775,6 | 6920413,3 | 9310021,9 | 7154786,1 | 6297127,4 | 8331157,0 | 0,156 |
| PCo(34:1)\|PCp(34:0)_M+CH3COO | 43105946,0 | 34559950,1 | 49298180,6 | 45811704,6 | 40133854,4 | 54146564,7 | 0,400 |
| Total Pco | 50760524,2 | 43313585,5 | 58078451,7 | 54097881,6 | 46349996,4 | 62185766,3 | 0,549 |
| PE(16:0/20:3)_M-H | 10111563,5 | 9214665,6 | 10527439,4 | 10688893,4 | 9962745,7 | 11679207,4 | 0,182 |
| PE(16:0/20:4)_M-H | 158321345,5 | 127246840,2 | 207713112,8 | 169601840,2 | 145086904,7 | 193528399,7 | 0,820 |
| PE(16:0/22:4)_M-H | 104549164,6 | 92617929,5 | 121773675,4 | 122351581,3 | 119097622,3 | 139194795,5 | 0,017 |
| PE(16:0/22:5)_M-H | 40901524,6 | 33918961,7 | 44984715,1 | 47395988,2 | 43079100,6 | 49425237,1 | 0,043 |
| PE(16:0/22:6)_M-H | 962223709,2 | 827107057,5 | 1034103161,5 | 942334743,7 | 901709401,3 | 980283528,5 | 0,780 |
| PE(18:0_18:1)_M-H | 878726052,1 | 483345977,6 | 1045932090,0 | 680455924,8 | 622035807,8 | 821806197,8 | 0,549 |
| PE(18:0/18:2)_M-H | 17135149,4 | 14270826,1 | 18473342,2 | 15048674,0 | 14091371,4 | 16724659,5 | 0,211 |
| PE(18:0/20:4)_M-H | 2065113913,0 | 1757979252,0 | 2286117040,5 | 2250901193,0 | 2031775433,5 | 2458519326,0 | 0,211 |
| PE(18:0/22:6)_M-H | 2574303292,0 | 2437808996,5 | 2878628771,0 | 2858597980,0 | 2583338656,0 | 3028395834,5 | 0,211 |
| PE(18:1/18:1)_M-H | 262333687,6 | 232099711,6 | 311863124,3 | 222286110,2 | 208049532,5 | 240608680,3 | 0,022 |
| PE(18:1/20:4)_M-H | 440706913,0 | 426119522,3 | 459495141,2 | 439393765,6 | 423453742,9 | 457282810,0 | 0,842 |
| PE(18:1_18:2)_M-H | 16709651,8 | 15457430,7 | 19480683,2 | 18697879,6 | 16053449,3 | 19280361,2 | 0,549 |
| PE(34:2)_M-H | 29712995,3 | 18760337,2 | 42679775,7 | 37162483,3 | 32112387,4 | 41630758,1 | 0,156 |
| PE(38:2)_M-H | 26343803,5 | 14817821,2 | 46702485,8 | 18992882,5 | 14804466,3 | 26741192,0 | 0,400 |
| Total PE | 7425464929,0 | 6877887475,3 | 8160755851,8 | 7851207525,0 | 7430783846,0 | 8219033886,0 | 0,243 |
| PEo(36:4)\|PEp(36:3)_M-H | 39690770,7 | 32424526,7 | 47078725,0 | 47858570,5 | 40937652,4 | 55476114,2 | 0,035 |
| PEo(38:6)\|PEp(38:5)_M-H | 137647790,8 | 107080742,8 | 146973589,2 | 144682113,0 | 121908479,6 | 156042767,9 | 0,356 |
| PEo(32:2)\|PEp(32:1)_M-H | 5342055,9 | 4724777,5 | 6286027,5 | 4497249,9 | 3201801,2 | 4993504,6 | 0,028 |
| PEo(16:1/20:3)\|PEp(16:0/20:3)_M-H | 19756200,9 | 17173444,9 | 30286173,8 | 22011179,2 | 18368286,6 | 25832386,3 | 1,000 |
| PEo(16:1/20:4)\|PEp(16:0/20:4)_M-H | 378585897,8 | 318381456,2 | 437755734,0 | 461169800,6 | 390372963,8 | 482550382,3 | 0,043 |
| PEo(16:1/20:4)\|PEp(16:0/20:4)_M+NaCH3COO | 18945180,4 | 15987062,1 | 22192757,9 | 23221568,0 | 20090899,1 | 24517905,7 | 0,043 |
| PEo(16:1/22:5)PEp(16:0/22:5)_M-H | 648190615,9 | 591545631,6 | 674801495,3 | 673547231,8 | 584855423,6 | 705868611,4 | 0,604 |
| PEo(16:1/22:5)\|PEp(16:0/22:5)_M-H | 55541728,5 | 46145996,8 | 63931164,3 | 70637873,3 | 60360481,4 | 108471157,9 | 0,013 |
| PEo(16:1/22:6)\|PEp(16:0_22:6)_M+NaCH3COO | 26339409,3 | 22493660,7 | 29121881,9 | 25202307,9 | 23575457,5 | 30760560,2 | 0,842 |
| PEo(16:1_22:6)\|PEp(16:0_22:6)_M-H | 1126435590,0 | 911058438,5 | 1247435724,3 | 1181068766,0 | 983546925,9 | 1307270764,0 | 0,780 |
| PEo(16:1_22:6)\|PEp(16:0_22:6)_M+NaCH3COO | 26321192,0 | 22465665,0 | 29093116,6 | 25168316,4 | 23536611,4 | 30685055,6 | 0,842 |
| PEo(18:1/22:6)\|PEp(18:0/22:6)_M-H // M-H | 40415522,0 | 38230641,8 | 44815227,9 | 44517951,5 | 40202941,8 | 48859534,2 | 0,133 |
| PEo(18:1/22:6)\|PEp(18:0/22:6)_M+NaCH3COO | 42690505,5 | 40072873,0 | 47312332,2 | 36433830,2 | 36134130,2 | 47358079,7 | 0,447 |
| PEo(18:1_22:6)\|PEp(18:0_22:6)_M-H | 1699975669,5 | 1509107384,3 | 1926306088,5 | 1728334443,0 | 1395214458,0 | 1936457261,5 | 0,842 |
| PEo(18:1_22:6)\|PEp(18:0_22:6)_M+NaCH3COO | 42690505,5 | 40085896,8 | 47363806,3 | 36443194,7 | 36147520,0 | 47363054,9 | 0,447 |
| PEo(18:2/16:1)\|PEp(18:1/16:1)_M-H | 27445692,5 | 21062212,6 | 35980842,3 | 18968109,2 | 17283919,5 | 23035593,9 | 0,013 |
| PEo(18:2/18:1)\|PEp(18:1/18:1)_M-H | 593635318,9 | 448785216,9 | 877441513,5 | 398261411,4 | 361042281,6 | 465333244,6 | 0,004 |
| PEo(18:2/18:2)\|PEp(18:1_18:2)\|PEp(18:2/18:1)_M-H | 13344179,5 | 9343060,3 | 16700912,8 | 11768051,5 | 9953169,3 | 15639436,1 | 0,780 |
| PEo(18:2/20:4)\|PEp(18:1/20:4)_M+NaCH3COO | 13812692,0 | 12263881,1 | 14540139,0 | 11868740,5 | 11316551,0 | 13232992,8 | 0,013 |
| PEo(18:2/22:6)\|PEp(18:1/22:6)_M-H | 648450400,5 | 587542838,7 | 721707713,3 | 668481711,8 | 602754017,3 | 727884649,3 | 0,720 |
| PEo(18:2/22:6)\|PEp(18:1/22:6)_M+NaCH3COO | 19713314,0 | 18162137,3 | 21515670,8 | 20193049,0 | 19047335,7 | 23156091,1 | 0,447 |
| PEo(35:2)\|PEp(17:0/18:1)_M-H | 25768949,7 | 11822905,0 | 36356109,9 | 18284197,5 | 14595545,3 | 21464685,1 | 0,549 |
| PEo(37:3)\|PEp(37:2)_M-H | 4076659,2 | 2052770,9 | 7794444,7 | 2559622,9 | 1906665,8 | 3575228,0 | 0,315 |
| PEo(38:5)\|PEp(38:4)_M-H | 330960385,5 | 274647979,6 | 369764314,3 | 371188428,8 | 324119926,9 | 426005063,6 | 0,182 |
| PEo(38:5)\|PEp(38:4)_M+NaCH3COO | 5256232,1 | 4780747,0 | 5954405,3 | 5895593,1 | 5243066,2 | 6348631,9 | 0,182 |
| PEo(39:5)\|PEp(17:0/22:4)_M-H | 9140935,1 | 8132526,4 | 10429560,5 | 8019123,7 | 7130475,3 | 9119174,5 | 0,156 |
| PEp(17:0/22:6)_M-H | 41499143,8 | 38453025,0 | 53160824,7 | 51846886,6 | 42064442,1 | 62247315,6 | 0,095 |
| Total PEo/Pep | 6039628658,0 | 5198102601,8 | 6598368536,5 | 6067018468,0 | 5224279871,5 | 6874167139,0 | 0,905 |
| PG(18:1/18:1)_M-H | 13576650,4 | 10995168,5 | 14458647,4 | 13143269,4 | 12241668,5 | 14788804,5 | 0,780 |
| PG(22:6/22:6)_M-H | 167853581,0 | 150222274,6 | 215968425,2 | 210647000,4 | 144769872,0 | 230289247,6 | 0,604 |
| PG(34:1)_M-H | 99843236,9 | 86403643,3 | 113947831,9 | 92874666,5 | 86615737,7 | 108945360,9 | 0,780 |
| PG(36:4)_M-H | 45096593,2 | 30029628,2 | 55676694,0 | 47953335,2 | 43020074,5 | 53000542,0 | 0,549 |
| PG(38:4)_M-H | 37640998,6 | 30164757,1 | 44190287,1 | 38565569,4 | 37161609,6 | 43157467,9 | 0,447 |
| PG(38:5)_M-H | 18695487,3 | 16105899,5 | 22867526,8 | 21625585,7 | 19898075,1 | 26888771,7 | 0,133 |
| PG(38:6)_M-H | 9358376,1 | 8346390,5 | 12396600,8 | 8458820,7 | 7981824,1 | 10467561,5 | 0,243 |
| Total PG | 390069668,2 | 355448094,7 | 472149479,2 | 444898127,0 | 353186861,1 | 467177904,0 | 0,549 |
| PI(16:0/20:4)_M-H | 398885488,9 | 338816869,2 | 455245977,8 | 413985511,0 | 381114296,0 | 460903079,0 | 0,497 |
| PI(18:0/20:4)_M-H | 2093948760,0 | 1686759610,8 | 2381762193,3 | 2273504571,0 | 2119848622,5 | 2384150332,0 | 0,211 |
| PI(18:0/22:6)_M-H | 52937738,4 | 44041150,7 | 80022472,5 | 37674101,6 | 35043788,2 | 42113501,7 | 0,008 |
| PI(18:1/18:1)_M-H | 14138569,2 | 12019464,3 | 19612727,7 | 12052970,5 | 10306052,1 | 13457065,5 | 0,053 |
| PI(18:1/20:4)_M-H | 341934456,6 | 287174012,7 | 436986981,5 | 411770596,3 | 366685774,7 | 426827061,8 | 0,278 |
| Total PI | 2889855861,0 | 2458452497,0 | 3283190195,3 | 3076903381,0 | 2951174595,5 | 3332065759,5 | 0,243 |
| PS(34:1)_M-H | 82789174,8 | 79941515,4 | 85842556,0 | 81701392,1 | 79681939,3 | 89578140,5 | 0,905 |
| PS(38:6)_M-H | 53250272,5 | 40852929,8 | 64460480,0 | 53105435,7 | 51763674,4 | 60080378,4 | 0,720 |
| PS(39:6)_M-H | 4609770,0 | 3438589,7 | 5479418,0 | 4756069,5 | 4490816,9 | 5147004,8 | 0,720 |
| PS(18:0/18:1)_M-H | 629350213,4 | 471795330,4 | 826560050,9 | 455936206,1 | 423463120,2 | 544837676,2 | 0,013 |
| PS(38:3)_M-H | 23070939,3 | 21429515,0 | 26800073,9 | 20223042,3 | 18002382,7 | 22167661,1 | 0,017 |
| PS(18:0/20:4)_M+Na-2H | 12524693,2 | 11472841,2 | 14269892,9 | 11423074,9 | 8109504,1 | 11726370,2 | 0,043 |
| PS(18:0/20:4)_M-H | 352135090,3 | 331770719,6 | 379464742,6 | 299874963,6 | 290453964,1 | 310465217,6 | <0,001 |
| PS(18:0/22:4)_M-H | 632793382,0 | 535057831,4 | 726869384,2 | 708641238,9 | 618786867,7 | 754055736,7 | 0,278 |
| PS(18:0/22:5)_M-H | 130871354,1 | 104063567,9 | 144960010,1 | 136273911,4 | 126208674,1 | 139318487,2 | 0,549 |
| PS(18:0/22:6)_M-H | 3899421170,5 | 3078942039,0 | 4451785983,8 | 3921412249,0 | 3771195883,0 | 4036899887,0 | 1,000 |
| PS(18:0/22:6)_M+Na-2H | 57172952,6 | 49709900,3 | 67229501,6 | 54225147,2 | 41508102,1 | 59095418,4 | 0,315 |
| PS(18:1/18:1)_M-H | 302292606,9 | 275723518,1 | 357288841,7 | 270724020,1 | 265042414,8 | 284998543,5 | 0,065 |
| PS(18:1/20:4)_M-H | 57913004,0 | 50396873,3 | 72613804,7 | 59082113,9 | 49445665,8 | 62235567,0 | 0,497 |
| PS(18:1/22:6)_M-H | 12264267,0 | 9080083,4 | 14372381,6 | 12943412,8 | 12038718,7 | 13774297,7 | 0,447 |
| PS(18:1/22:6))_M-H | 162555988,7 | 141426025,5 | 205371562,6 | 178137119,7 | 149346743,0 | 187525090,2 | 1,000 |
| PS(18:1_20:1)_M-H | 5425589,9 | 4451238,1 | 8375629,4 | 3158885,2 | 2230321,8 | 4840402,3 | 0,004 |
| PS(22:4/22:6)_M-H | 245075264,1 | 198536829,3 | 266881500,6 | 236430152,1 | 201903074,2 | 273789369,3 | 1,000 |
| PS(22:6/22:6)_M-H | 139729248,8 | 126058625,7 | 191487491,5 | 131874642,0 | 102920589,6 | 141548295,2 | 0,053 |
| PS(22:6/22:6)_M+Na-2H | 5061016,2 | 4503828,3 | 7116748,3 | 4519034,8 | 3878703,9 | 5135679,8 | 0,095 |
| PS(36:1)_M-H | 627579330,7 | 470402804,9 | 826875088,9 | 456630604,7 | 422846705,0 | 545316927,3 | 0,013 |
| PS(36:4)_M-H | 17306833,0 | 14234148,6 | 18961975,0 | 17941835,0 | 17290849,6 | 19012059,0 | 0,315 |
| PS(40:2)_M-H | 9114590,7 | 6623893,0 | 12904069,6 | 6977387,1 | 5484524,0 | 7363419,4 | 0,113 |
| PS(40:7)_M+Na-2H | 2933373,4 | 2610915,3 | 3916962,4 | 3207857,7 | 2989643,7 | 3410022,5 | 0,905 |
| PS(42:10)_M-H | 40020625,0 | 33386965,8 | 49396613,7 | 39860400,4 | 36063336,5 | 47266946,6 | 0,780 |
| PS(42:5)_M-H | 11192551,3 | 9189225,6 | 13885311,5 | 12249018,2 | 11028135,0 | 14606489,9 | 0,278 |
| PS(42:7)_M-H | 4587273,0 | 3968416,1 | 6124132,7 | 4009584,2 | 2174102,7 | 4347463,6 | 0,028 |
| PS(44:10)_M+Na-2H | 8235366,7 | 6633156,4 | 9337142,6 | 8266468,7 | 7112231,6 | 9674766,8 | 0,661 |
| PS(44:11)_M-H | 11947021,0 | 11169444,2 | 16023569,8 | 11067052,3 | 10005325,5 | 13741797,8 | 0,182 |
| PS(44:7)_M+Na-2H | 6000027,8 | 5473622,5 | 8102098,4 | 6912061,1 | 6427181,9 | 8539140,0 | 0,315 |
| Total PS | 7545360262,5 | 7306894648,0 | 7801758382,5 | 7224767595,0 | 7136475542,0 | 7361316615,5 | 0,035 |

**Table S5.** Lipid variables by mouse genotype with median, interquartile and p-value in positive ionization mode in the hippocampus.

| **Lipid variables** | **WT** | | | **TG** | | | **p value (Mann-Whitney)** |
| --- | --- | --- | --- | --- | --- | --- | --- |
|  | **Median** | **IQ1** | **IQ3** | **Median** | **IQ1** | **IQ3** |  |
| CE(20:0)_M+NH4 | 4781334,5 | 4359571,8 | 5078801,3 | 5402046,5 | 4649358,1 | 5945314,2 | 0,075 |
| Cer(d16:1/17:0)_M+H-H2O | 9207721,9 | 9035086,2 | 9801063,0 | 9727540,0 | 9206340,3 | 10261058,4 | 0,280 |
| Cer(d16:1/17:0)_M+H | 8134270,8 | 7829551,7 | 8616233,6 | 8529681,0 | 8077941,5 | 9137369,1 | 0,165 |
| Cer(d18:1/17:0)_M+Na | 383628151,9 | 356480272,5 | 401098158,5 | 441550918,4 | 383731814,3 | 490156626,6 | 0,019 |
| Cer(d18:1/18:0)_M+H-H2O | 193359655,4 | 144695057,6 | 261586761,6 | 176813950,2 | 127398763,3 | 226544706,7 | 0,631 |
| Cer(d18:1/18:0)_M+H | 245524486,5 | 183510305,1 | 330799867,5 | 227162508,8 | 161121545,4 | 296122673,0 | 0,684 |
| Cer(d18:1/18:0)_M+Na | 125203384,3 | 106811546,6 | 172140878,6 | 112190539,3 | 94628448,6 | 130594989,9 | 0,247 |
| Cer(d18:1/18:1)_M+H-H2O | 36566065,0 | 26987951,2 | 47815194,6 | 31828438,2 | 19323530,9 | 41245828,4 | 0,190 |
| Cer(d18:1/18:1)_M+H | 45048840,2 | 33128578,7 | 57869121,2 | 39607493,6 | 24726459,2 | 51083747,6 | 0,218 |
| Cer(d18:1/18:1)_M+Na | 17085285,5 | 12640829,6 | 21002421,3 | 14552278,8 | 9238258,9 | 18397617,2 | 0,143 |
| Cer(d18:1/22:0)_M+H | 7192164,9 | 6301085,3 | 9029609,3 | 7690091,2 | 5604225,0 | 8407863,1 | 0,631 |
| Cer(d18:1/24:1)_M+H-H2O | 19474988,3 | 17788845,7 | 22369197,5 | 18393181,8 | 15025493,4 | 21246866,4 | 0,353 |
| Cer(d18:1/24:1)_M+H | 60689157,2 | 59329120,7 | 68749231,7 | 57699104,2 | 49853852,0 | 69050285,3 | 0,247 |
| Total Cer | 1075336794,7 | 939679178,2 | 1301662099,8 | 1088079470,5 | 944531480,9 | 1262542148,8 | 0,912 |
| DAG(16:0/16:0)_M+H-H2O | 9997781,9 | 8624074,0 | 10817339,3 | 10043688,8 | 9290454,2 | 11057545,1 | 0,579 |
| DAG(16:0/18:1)_M+H-H2O | 33068252,9 | 29653359,2 | 36753657,8 | 33891114,7 | 30286019,6 | 36694900,0 | 0,796 |
| DAG(16:0/18:1)_M+NH4 | 57134351,2 | 52354999,6 | 69549265,8 | 52487622,4 | 40719150,9 | 60313886,2 | 0,190 |
| DAG(16:0/18:2)_M+H-H2O | 7959757,8 | 7406404,3 | 8476631,0 | 8652950,0 | 7915563,8 | 9473640,4 | 0,143 |
| DAG(16:0/20:4)_M+NH4 | 15643112,1 | 13660767,9 | 18856578,2 | 14097341,1 | 10799827,5 | 17153613,1 | 0,165 |
| DAG(16:0/20:4)_M+Na | 14073654,8 | 12124851,8 | 17320992,2 | 12245975,3 | 9398899,6 | 14659810,5 | 0,190 |
| DAG(16:0/22:1)_M+NH4 | 13607586,8 | 10687748,9 | 15536665,5 | 14977216,7 | 9701121,4 | 16742504,9 | 0,971 |
| DAG(16:0/22:6)_M+H-H2O | 35545989,0 | 34259007,5 | 39194069,0 | 37746801,9 | 24698593,6 | 39907810,9 | 1,000 |
| DAG(18:0/18:0)_M+H-H2O | 2767293,2 | 2501534,5 | 2883045,6 | 2864596,9 | 2682789,6 | 3015718,7 | 0,218 |
| DAG(18:0/18:0)_M+NH4 | 6958273,1 | 6050857,8 | 7061980,5 | 7203342,9 | 6504587,8 | 7451217,5 | 0,165 |
| DAG(18:0/18:0)_M+Na | 10854998,6 | 9567856,0 | 11401015,2 | 11165649,3 | 10264634,5 | 12146185,7 | 0,280 |
| DAG(18:0/20:4)_M+H-H2O | 96826676,2 | 77975551,0 | 110989535,6 | 73440480,5 | 54620847,9 | 123596625,2 | 0,579 |
| DAG(18:0/20:4)_M+NH4 | 388126017,3 | 350746545,4 | 427349480,5 | 362719116,9 | 261353113,6 | 411717720,2 | 0,353 |
| DAG(18:0/20:4)_M+Na | 163015638,6 | 147792351,2 | 174869617,7 | 164935610,2 | 129098137,1 | 178604369,0 | 0,853 |
| DAG(18:0/22:4)_M+NH4 | 27916775,7 | 21161726,8 | 32945339,1 | 25325493,7 | 19224243,7 | 36175643,9 | 0,796 |
| DAG(18:0/22:4)_M+Na | 15647525,3 | 12005387,1 | 18594292,9 | 13943357,9 | 10620306,3 | 20446345,7 | 0,912 |
| DAG(18:0/22:6)_M+H-H2O | 141468028,1 | 135832870,2 | 153576930,0 | 138626669,2 | 90684108,9 | 147087114,1 | 0,315 |
| DAG(18:1/16:0)_M+H-H2O | 38466814,4 | 34730877,0 | 42225675,4 | 39847457,6 | 35429770,3 | 43336064,1 | 0,436 |
| DAG(18:1/18:1)_M+H-H2O | 88197758,3 | 83627843,9 | 94931906,8 | 90345168,4 | 77813774,5 | 92193042,8 | 0,739 |
| DAG(18:1/18:1)_M+NH4 | 108563208,0 | 103759751,0 | 111718781,2 | 103443907,0 | 85257000,3 | 112620268,8 | 0,247 |
| DAG(18:1/18:1)_M+Na | 45581160,5 | 42189596,0 | 50598971,2 | 47557980,0 | 39489024,9 | 49967304,9 | 0,853 |
| DAG(18:1/18:2)_M+H-H2O | 12916432,2 | 11702512,2 | 13498091,6 | 13458952,9 | 12327846,1 | 15139903,2 | 0,280 |
| DAG(18:2/18:2)_M+H-H2O | 1277865,3 | 1161812,2 | 1406477,7 | 1437037,5 | 1295296,9 | 1565558,5 | 0,052 |
| Total DAG | 1331259920,5 | 1278111912,3 | 1448347640,0 | 1257008741,0 | 1069454626,7 | 1384344783,5 | 0,280 |
| LPC(16:0)_M+H | 281301252,6 | 269771438,4 | 321458477,1 | 298596636,6 | 270980201,9 | 372884076,7 | 0,315 |
| LPC(18:0)_M+H | 91574888,6 | 86937784,8 | 98279911,6 | 107854888,7 | 79822007,5 | 122950062,8 | 0,579 |
| LPC(18:1)_M+H | 109162387,1 | 97112720,3 | 127760289,5 | 103034206,2 | 97431158,4 | 122047892,7 | 0,739 |
| LPC(20:4)_M+H | 48377649,4 | 44201524,3 | 57540520,0 | 48747831,4 | 43629768,9 | 63030609,1 | 0,631 |
| LPC(22:6)_M+H | 39869532,5 | 31397973,5 | 44319757,8 | 36882923,0 | 33625021,7 | 41216590,6 | 0,579 |
| Total LPC | 587017555,1 | 550235216,5 | 661930342,0 | 579624597,9 | 542752303,3 | 710035735,5 | 0,796 |
| LPE(22:6)_M+H | 31677401,7 | 28244548,0 | 35558631,5 | 31642340,6 | 29259361,1 | 36701512,4 | 0,739 |
| MAG(18:2)_M+NH4 | 9355447,6 | 8494259,7 | 10565247,7 | 9393171,1 | 7243867,3 | 13383104,4 | 0,912 |
| MAG(20:1)_M+NH4 | 3682338,2 | 2415180,8 | 7696996,4 | 3825880,2 | 2392028,7 | 4368153,5 | 0,631 |
| MAG(20:2)_M+NH4 | 28661316,1 | 26664789,2 | 33540615,5 | 28168134,7 | 23177733,0 | 39594319,5 | 1,000 |
| MAG(20:4)_M+H-H2O | 5722551,6 | 4983816,3 | 6349717,6 | 5237486,1 | 3801412,3 | 5986544,6 | 0,315 |
| MAG(22:2)_M+NH4 | 15989753,1 | 14896848,5 | 19159654,9 | 15765742,6 | 13481811,3 | 16755391,5 | 0,393 |
| MAG(22:3)_ M+NH4 | 20833199,3 | 16191167,5 | 25014726,3 | 20208201,5 | 16193194,0 | 30748131,0 | 0,912 |
| MAG(22:4)_M+NH4 | 20958316,3 | 16248474,0 | 24998519,2 | 20226756,7 | 16098547,0 | 30866434,0 | 0,912 |
| Total MAG | 108066258,1 | 95897047,3 | 121140111,2 | 103780156,7 | 84810540,6 | 139294279,0 | 0,739 |
| PC(14:0_16:0)_M+H | 172285938,8 | 128627286,4 | 235035281,5 | 144055797,2 | 107817952,7 | 171130715,6 | 0,089 |
| PC(16:0/16:0)_M+H | 10196873900,5 | 7433858450,5 | 12521410270,3 | 8220994443,0 | 5124878130,0 | 11648731332,0 | 0,315 |
| PC(16:0/16:0)_M+Na | 298483109,6 | 236578757,4 | 331251272,1 | 255672013,4 | 151532307,0 | 326981480,4 | 0,280 |
| PC(16:0_18:1)_M+H | 20604750680,0 | 18652890978,0 | 21889759500,5 | 19273895350,5 | 12831193236,5 | 21395262361,3 | 0,218 |
| PC(16:0_18:1)_M+Na | 546535961,7 | 536362726,0 | 564032470,5 | 529852285,2 | 390652874,1 | 565564564,0 | 0,247 |
| PC(16:0_20:4)_M+H | 3810326344,5 | 3003426963,8 | 3888650256,5 | 3498129625,0 | 2350974360,8 | 4169278313,8 | 0,971 |
| PC(16:0_20:4)_M+Na | 95727768,2 | 62587869,3 | 97526628,4 | 80189616,6 | 42670571,7 | 106669674,1 | 0,436 |
| PC(16:0_22:4)_M+H | 307139780,8 | 265324159,8 | 340193501,9 | 285265249,5 | 201698641,9 | 315635817,3 | 0,143 |
| PC(16:0_22:6)_M+H | 5488892034,5 | 4543642609,8 | 5871828974,3 | 4525464623,5 | 3810586095,3 | 5431971048,8 | 0,105 |
| PC(16:0_22:6)_M+Na | 55415740,5 | 44049669,7 | 60116926,9 | 40980907,1 | 34826439,0 | 51779579,6 | 0,165 |
| PC(16:1/22:6)_M+H | 25650559,6 | 23708699,6 | 27871915,6 | 23206334,5 | 18440333,7 | 28810291,1 | 0,684 |
| PC(17:0/18:1)_M+Na | 31544134,6 | 28481715,3 | 36237700,4 | 30954292,5 | 21477941,0 | 36402716,4 | 0,739 |
| PC(18:0/22:4)_M+H | 209088443,4 | 177334405,3 | 236841812,3 | 178195182,9 | 125669549,2 | 188274527,2 | 0,023 |
| PC(18:0/22:5)_M+H | 43622370,9 | 36729718,2 | 51178605,0 | 38307991,4 | 28060023,4 | 48068975,5 | 0,247 |
| PC(18:0_18:1)_M+H | 5425130658,5 | 5161671664,8 | 6583484554,8 | 4483126156,5 | 3685352326,8 | 6506758371,5 | 0,190 |
| PC(18:0_18:1)_M+Na | 124509994,1 | 110262817,7 | 162328719,4 | 94425878,2 | 70393624,7 | 129491903,5 | 0,165 |
| PC(18:0_20:3)_M+H | 64571035,3 | 56982264,8 | 70719270,8 | 60202098,3 | 41833775,8 | 69975949,6 | 0,190 |
| PC(18:0_20:4)_M+H | 4096231492,0 | 3643790073,3 | 4418650853,0 | 3717065747,5 | 2323638231,0 | 4831700377,3 | 0,529 |
| PC(18:0_20:4)_M+Na | 107610245,2 | 98642153,1 | 114710707,2 | 100517140,1 | 62111421,5 | 132881389,7 | 0,796 |
| PC(18:0_22:6)_M+H | 1729453562,5 | 1417063632,3 | 1966494317,3 | 1276750321,5 | 1023588501,1 | 1533476145,5 | 0,029 |
| PC(18:0_22:6)_M+Na | 47084368,0 | 37368787,2 | 51930070,8 | 32556722,0 | 25663595,6 | 38368046,0 | 0,043 |
| PC(18:1/16:1)_M+Na | 13370296,6 | 12614434,8 | 14700872,4 | 12707552,9 | 10641183,2 | 15772681,0 | 0,393 |
| PC(18:1/16:1)_M+H | 411852564,4 | 373542283,9 | 462126799,1 | 408738221,3 | 338106889,7 | 480937608,5 | 0,579 |
| PC(18:1/18:1)_M+H | 1710334470,0 | 1473859259,5 | 1855959997,8 | 1629188891,5 | 1089679180,7 | 1742923467,0 | 0,165 |
| PC(18:1/20:4)_M+H | 1385571943,0 | 1172001613,0 | 1475977351,5 | 1326027697,0 | 1133604904,6 | 1705370645,8 | 0,796 |
| PC(18:1/20:4)_M+Na | 42717851,2 | 38133941,3 | 47667790,5 | 41547078,3 | 36148413,1 | 52713888,2 | 0,853 |
| PC(18:2/20:4)_M+H | 28557917,5 | 24561644,5 | 30900569,1 | 30338556,4 | 26436344,7 | 36564759,5 | 0,315 |
| PC(20:1/20:4)_M+H | 90537101,6 | 84201906,6 | 100572706,0 | 83299400,6 | 64320698,5 | 106192796,1 | 0,481 |
| PC(20:1_22:6)_M+H | 53805328,7 | 39364390,9 | 59056903,7 | 30995959,4 | 23756749,1 | 42119855,3 | 0,011 |
| PC(20:4/20:4)_M+H | 40979900,4 | 38944360,0 | 42008477,7 | 40393006,5 | 38367017,1 | 42202210,3 | 0,912 |
| PC(20:4_22:6)_M+H | 100865573,6 | 90340046,2 | 104909037,6 | 94019439,3 | 90196956,3 | 98626957,1 | 0,165 |
| PC(22:6/22:6)_M+H | 100941900,1 | 71215131,8 | 152977969,4 | 82362393,6 | 62042714,8 | 113634637,6 | 0,280 |
| PC(31:0)_M+H | 34483573,6 | 26080551,5 | 42077257,7 | 28653910,8 | 22002060,3 | 33433587,5 | 0,063 |
| PC(33:2)_M+H | 249951475,8 | 201978236,8 | 281032774,0 | 212949492,4 | 157505407,2 | 225927273,9 | 0,035 |
| PC(33:3)_M+H | 7041498,8 | 6618707,5 | 8051750,8 | 6812803,1 | 5825568,2 | 8077753,8 | 0,529 |
| PC(35:4)_M+Na | 233488038,3 | 223554532,6 | 243985408,5 | 230581725,1 | 180241543,9 | 246551029,9 | 0,315 |
| PC(36:4)_M+H | 6568142,5 | 5503841,5 | 7536933,1 | 6959344,1 | 3890708,4 | 7719779,8 | 1,000 |
| PC(36:5)_M+H | 24941955,7 | 22407961,8 | 26622085,6 | 26276810,1 | 22180341,0 | 28507061,7 | 0,353 |
| PC(38:5)_M+H | 27522814,0 | 26146301,0 | 33131538,1 | 27087909,7 | 23472083,7 | 30947351,6 | 0,579 |
| PC(40:7)_M+H | 656370930,4 | 493642553,4 | 703484745,2 | 482365049,2 | 366585685,4 | 651408154,7 | 0,143 |
| PC(40:7)_M+Na | 8833095,2 | 7141085,3 | 9806361,2 | 5723536,0 | 4338147,7 | 8234790,2 | 0,075 |
| Total PC | 58483467179,5 | 53928262008,3 | 60814435534,3 | 54298051148,5 | 36064617481,3 | 61462901936,8 | 0,393 |
| PCo(16:0/16:0)_M+H | 73044800,3 | 45517326,5 | 95771221,2 | 56446359,8 | 36625337,4 | 87250081,0 | 0,218 |
| PCo(18:1/16:0)\|PCp(16:0_18:0)_M+H | 237781312,6 | 199448847,3 | 280999233,0 | 204395136,1 | 146971525,9 | 253852360,5 | 0,143 |
| PCo(35:5)\|PCp(35:4)_M+Na | 385491203,5 | 370649761,0 | 438215562,7 | 399456192,9 | 303150059,9 | 459433528,5 | 0,853 |
| PCo(36:7)\|PCp(36:6)_M+H | 63285575,4 | 62174909,9 | 68569709,8 | 60607681,6 | 54873353,9 | 64778482,8 | 0,247 |
| PCo(37:6)\|PCp(37:5)_M+Na | 91741444,1 | 85312199,6 | 93893259,2 | 86866038,0 | 82882738,6 | 95921457,2 | 0,631 |
| PCo(37:6)\|PCp(37:5)_M+H | 29478392,6 | 24190525,4 | 34325800,9 | 27195790,3 | 22073088,9 | 32550579,3 | 0,631 |
| PCo(38:5)\|PCp(38:4)_M+H | 20607442,5 | 18188372,2 | 23307718,3 | 17872018,7 | 13384400,4 | 20273003,4 | 0,063 |
| PCo(38:7)\|PCp(38:6)_M+H | 21474136,8 | 19127926,9 | 23871049,6 | 19895177,9 | 17301544,0 | 29273401,4 | 0,739 |
| PCp(16:0/16:0)\|PCo(16:0_16:1)_M+H | 52749950,7 | 31876340,7 | 69418929,1 | 40157439,8 | 23114158,7 | 70167692,0 | 0,481 |
| PCp(34:6)_M+H | 50458232,6 | 35669618,5 | 57729011,5 | 39586805,4 | 18287063,9 | 53748653,2 | 0,247 |
| PCp(35:5)_M+Na | 8664026,0 | 7624997,3 | 10646933,4 | 9174118,8 | 8038698,4 | 10846137,8 | 0,684 |
| PCp(35:5)\|PEo(38:6)_M+Na | 74970432,9 | 62676849,1 | 83917784,0 | 64491345,6 | 38259119,8 | 79371872,9 | 0,218 |
| PCp(35:5)\|PCo(33:3)_M+H | 519124312,2 | 442639545,1 | 559873070,4 | 458746804,1 | 342167687,6 | 548426015,3 | 0,165 |
| PCp(37:6)_M+Na | 118861121,9 | 112269343,6 | 124410410,1 | 107827835,9 | 72183336,0 | 120955326,5 | 0,063 |
| PCp(38:5)\|\|PCo(38:6)_M+H | 17423041,2 | 15160191,3 | 18172537,3 | 18185819,4 | 14323742,6 | 20391248,4 | 0,579 |
| PCp(39:6)_M+Na | 18371765,4 | 15945834,0 | 25741203,4 | 12948827,6 | 8654423,1 | 19209448,2 | 0,089 |
| Total PCo | 1766900905,5 | 1637279436,8 | 1927013041,8 | 1631277150,5 | 1208190037,0 | 1943659057,8 | 0,315 |
| PE(18:0_22:4)_M+H | 260701477,2 | 233893069,4 | 348882921,5 | 218817726,3 | 193199211,8 | 337054135,0 | 0,165 |
| PE(22:6/16:0)_M+H | 658607138,2 | 633270506,1 | 718991730,8 | 690560821,2 | 461565440,5 | 743065517,3 | 1,000 |
| PE(22:6/16:0)_M+Na | 16284446,1 | 15489676,1 | 17727143,3 | 15858819,1 | 13707116,7 | 17427758,1 | 0,393 |
| PE(22:6/18:0)_M+Na | 41811249,8 | 39942438,7 | 44003796,8 | 40622385,3 | 39718423,2 | 42504220,5 | 0,353 |
| PE(22:6/22:6)_M+H | 39712539,2 | 31001826,4 | 55945649,0 | 35307641,4 | 32224398,0 | 40545866,1 | 0,315 |
| PE(34:1)_M+H | 174811641,7 | 169305980,9 | 191463580,8 | 152919706,3 | 122240678,4 | 198241404,1 | 0,218 |
| PE(36:1)_M+H | 311831922,0 | 280044338,6 | 383191469,3 | 309351185,4 | 237328928,5 | 415401382,9 | 0,579 |
| PE(36:4)_M+H | 185789045,0 | 175626781,0 | 194157098,6 | 161168034,5 | 127275238,9 | 205555823,0 | 0,436 |
| PE(37:4)_M+H | 7573502,3 | 6641057,5 | 7952839,2 | 6351399,5 | 4673520,4 | 8281077,1 | 0,436 |
| PE(38:3)_M+H | 15991439,4 | 15292643,7 | 19223180,3 | 14513240,6 | 11526590,8 | 20513373,5 | 0,315 |
| PE(38:4)_M+H | 77911954,2 | 72179122,4 | 92294154,6 | 65993358,8 | 50115806,0 | 96071538,1 | 0,393 |
| PE(20:4/18:0)_M+H | 1684142686,5 | 1366156633,5 | 1919039919,3 | 1276882549,0 | 953409018,6 | 2125063101,3 | 0,529 |
| PE(38:4)_M+Na | 41772919,8 | 39245135,4 | 49454593,1 | 37605677,2 | 32641618,0 | 48277392,8 | 0,247 |
| PE(39:6)_M+H | 10231255,5 | 9097430,1 | 11401119,3 | 9070986,0 | 7353404,5 | 10005914,0 | 0,123 |
| PE(40:5)_M+H | 73737520,9 | 60346114,1 | 78305267,3 | 64960644,8 | 47921498,8 | 85164717,8 | 0,481 |
| PE(40:6)_M+Na | 42583724,8 | 40652844,9 | 44744156,6 | 41819976,2 | 40494762,6 | 43475173,9 | 0,393 |
| PE(40:6)_M+H | 2892492881,5 | 2742143236,0 | 3106528818,3 | 2801896008,0 | 1816656170,5 | 2991947208,5 | 0,247 |
| PE(42:10)_M+H | 18887725,6 | 16982550,0 | 19947551,3 | 18053089,8 | 17094812,5 | 19277720,3 | 0,393 |
| PE(44:10)_M+H | 56845575,6 | 51802036,5 | 66064594,9 | 50437914,0 | 45890795,3 | 55776812,5 | 0,089 |
| Total PE | 6626628015,5 | 6360597990,3 | 6928221583,5 | 6310075897,5 | 4264394593,5 | 7366474693,0 | 0,631 |
| PEo(16:1_20:4)\|PEp(36:4)_M+H | 222444202,5 | 159308593,6 | 238703726,9 | 150720901,1 | 124417835,7 | 184764578,0 | 0,035 |
| PEo(16:1_22:4)PEp(38:4)_M+H | 207016991,6 | 182809361,8 | 259730824,4 | 167341482,6 | 136773062,0 | 259664193,2 | 0,218 |
| PEo(16:1_22:6)\|PEp(16:0/22:6)_M+Na | 13151940,7 | 12601849,1 | 15082420,5 | 14431318,3 | 11083969,9 | 16584884,9 | 0,579 |
| PEo(16:1_22:6)\|PEp(16:0/22:6)_M+H | 721179975,0 | 608017610,7 | 760439770,5 | 728851048,1 | 428998781,9 | 814945236,7 | 0,853 |
| PEo(18:1_18:2)\|PEp(18:1/18:1)_M+H | 573809393,1 | 524103636,5 | 698267344,6 | 475866028,4 | 407959324,8 | 629236307,9 | 0,075 |
| PEo(18:1_20:4)\|PEp(18:0/20:4)_M+H | 501512996,8 | 414038596,9 | 605654647,5 | 426536396,3 | 291529361,2 | 467655213,0 | 0,035 |
| PEo(18:1_20:4)_M+Na | 17334465,9 | 15640383,4 | 19857664,9 | 14549933,1 | 11993351,5 | 16968788,5 | 0,023 |
| PEo(18:1_22:6)_M+H | 1232393521,0 | 1151594949,5 | 1372305349,5 | 1203127922,0 | 722405385,6 | 1371504084,5 | 0,436 |
| PEo(18:1_22:6)_M+Na | 37559753,3 | 36930245,1 | 40659294,5 | 36196177,9 | 27986725,5 | 39328152,0 | 0,190 |
| PEo(18:2_22:4)\|PEp(18:1/22:4)_M+H | 695173545,3 | 659590951,0 | 768611009,8 | 643411749,7 | 498114588,2 | 917415630,2 | 0,796 |
| PEo(18:2_22:6)_M+Na | 8583195,1 | 7490838,4 | 9122391,6 | 7799289,2 | 6018621,3 | 9124367,2 | 0,353 |
| PEo(34:2)\|PEp(34:1)_M+H | 285825800,9 | 258943765,5 | 326690376,4 | 260282107,0 | 224180496,7 | 333066735,7 | 0,315 |
| PEo(36:4)\|PEp(36:3)_M+H | 23818523,9 | 19047318,0 | 28632593,8 | 20108496,2 | 14222815,1 | 22574981,1 | 0,043 |
| PEo(36:5)\|PEp(36:4)_M+Na | 6934425,4 | 5287544,7 | 7532730,1 | 4864858,2 | 3729193,6 | 5794790,9 | 0,290 |
| PEo(37:5)\|PEp(37:4)_M+H | 8739056,7 | 7556106,5 | 10221013,4 | 7330724,9 | 5853180,8 | 8815154,5 | 0,063 |
| PEo(38:4)\|PEp(38:3)_M+H | 27114327,8 | 22860045,5 | 36829961,1 | 22641793,4 | 20713872,9 | 28670844,2 | 0,143 |
| PEo(38:6)\|PEp(38:5)_M+H | 80422101,1 | 66792758,5 | 88038520,5 | 71687528,1 | 47989512,3 | 86202255,4 | 0,535 |
| PEo(40:6)\|PEp(40:5)_M+H | 55492647,2 | 52906579,1 | 64119943,8 | 48892091,8 | 37076112,7 | 55260852,7 | 0,035 |
| PEo(40:7)\|PEp(40:6)_M+H | 18582123,7 | 16918778,8 | 21832332,5 | 17439036,9 | 11083096,9 | 19384652,7 | 0,190 |
| Total PEo | 4681541199,0 | 4392400334,3 | 5230341546,5 | 4271293832,5 | 3062008006,8 | 5238012907,0 | 0,353 |
| PG(34:1)_M+NH4 | 71270868,4 | 61778028,4 | 80920709,1 | 54047617,3 | 47714214,6 | 63949265,4 | 0,019 |
| PG(34:1)_M+Na | 11672663,5 | 10918773,2 | 13030727,4 | 7859102,1 | 6324001,1 | 10638971,2 | 0,004 |
| T.Pg | 1547741131,5 | 1504195815,8 | 1664221722,0 | 1727622170,0 | 1537926694,3 | 1835734059,5 | 0,052 |
| SM(d18:1/17:0)_M+Na | 259201021,0 | 237728264,3 | 272248114,6 | 263238565,5 | 250595382,5 | 299492540,6 | 0,529 |
| SM(d18:1/18:0)_M+H | 1021985421,0 | 632916909,2 | 1428834315,3 | 603287164,6 | 520726502,7 | 1316544852,0 | 0,247 |
| SM(d18:1/18:0)_M+Na | 27589764,0 | 14529595,8 | 44942960,8 | 14284354,5 | 11551821,8 | 31778565,0 | 0,165 |
| SM(d38:1)_M+H | 73727129,9 | 64542036,4 | 127764285,3 | 63445178,5 | 40477968,2 | 94265159,6 | 0,165 |
| SM(d42:2)_M+H | 50850594,4 | 38875282,9 | 73542628,2 | 48317006,6 | 26343925,5 | 65650629,4 | 0,436 |
| Total SM | 1429782800,5 | 1011444244,4 | 1877456622,3 | 1055984023,0 | 873607995,9 | 1730181185,3 | 0,353 |
| TAG(10:0/14:1/18:0)_M+NH4 | 12825917,6 | 11718790,1 | 13361756,3 | 14229628,5 | 12193852,1 | 14635939,7 | 0,029 |
| TAG(10:0/15:0/18:0)_M+NH4 | 10050130,1 | 9221157,5 | 10674607,6 | 11262945,8 | 9703804,7 | 11735217,5 | 0,089 |
| TAG(10:0/16:1/18:0)_M+Na | 10979470,6 | 10175485,1 | 11830601,4 | 12395399,0 | 10928815,4 | 12717812,7 | 0,015 |
| TAG(10:0/16:1/18:0)_M+NH4 | 49978873,8 | 45553278,4 | 52327250,7 | 55943725,7 | 48160145,5 | 57381838,7 | 0,029 |
| TAG(10:0/18:2/18:0)_M+Na | 21568343,6 | 19371717,9 | 22313570,3 | 23770663,2 | 20693904,9 | 24365952,9 | 0,029 |
| TAG(12:0/14:0/18:0)_M+NH4 | 46071675,7 | 42003606,7 | 48420091,1 | 49436769,0 | 43438953,0 | 51770263,0 | 0,043 |
| TAG(12:0/14:0/18:0)_M+Na | 8456724,2 | 7480267,1 | 8940635,4 | 8859973,0 | 8205784,4 | 9586507,5 | 0,105 |
| TAG(12:0/14:0/18:2)_M+NH4 | 16809833,7 | 15124158,6 | 17517798,2 | 18772393,2 | 16236086,0 | 19273692,1 | 0,019 |
| TAG(12:0/15:0/18:0)_M+NH4 | 37803008,0 | 33589686,5 | 38521086,1 | 38653195,2 | 35728787,9 | 41521464,8 | 0,165 |
| TAG(12:0/15:0/18:0)_M+Na | 10034332,5 | 9201327,3 | 10541171,1 | 10893123,3 | 9794330,3 | 11302857,3 | 0,052 |
| TAG(12:0/16:0/20:1)_M+NH4 | 174283833,9 | 161886036,2 | 181594165,0 | 189893477,9 | 170873545,3 | 202281095,2 | 0,089 |
| TAG(12:0/16:0/20:1)_M+Na | 35655087,0 | 33216896,1 | 36894275,2 | 38371583,6 | 34768280,1 | 41174043,2 | 0,105 |
| TAG(12:0/16:1/17:0)_M+Na | 11308431,5 | 9543668,8 | 11639270,3 | 11904148,4 | 11035417,3 | 12760953,5 | 0,035 |
| TAG(14:0/16:1/15:0)_M+Na | 8550591,4 | 7533534,8 | 8938663,1 | 9621031,1 | 8622176,4 | 9948382,6 | 0,023 |
| TAG(12:0/16:1/17:0)_M+NH4 | 53150870,7 | 46541393,3 | 54947056,2 | 57267613,2 | 50518545,6 | 60798698,8 | 0,023 |
| TAG(12:0/16:1/18:0)_M+Na | 41651739,3 | 37639159,6 | 43617698,7 | 44580700,7 | 40248983,4 | 48183697,6 | 0,035 |
| TAG(12:0/18:2/12:0)_M+NH4 | 2009011,7 | 1901364,2 | 2069274,3 | 2194946,9 | 1972704,9 | 2247930,7 | 0,089 |
| TAG(12:0/20:0/22:1)_M+NH4 | 27575524,7 | 22258117,8 | 30156351,1 | 27515250,8 | 23297935,0 | 29544345,0 | 0,912 |
| TAG(12:0/20:0/22:1)_M+Na | 6688065,5 | 5605973,9 | 7481255,8 | 6800229,1 | 5709537,3 | 7213372,2 | 1,000 |
| TAG(14:0/14:0/14:0)_M+Na | 4242557,6 | 3825273,1 | 4465058,0 | 4609479,4 | 4086227,1 | 4731294,1 | 0,029 |
| TAG(14:0/14:0/14:0)_M+NH4 | 14104140,0 | 12937727,1 | 14840981,9 | 15659828,9 | 13356646,7 | 15993005,5 | 0,089 |
| TAG(14:0/14:0/18:0)_M+NH4 | 90602938,0 | 82553919,9 | 92515488,5 | 96561646,0 | 85645819,8 | 102405404,2 | 0,089 |
| TAG(14:0/14:0/18:2)_M+NH4 | 95728942,4 | 84395901,9 | 98301740,3 | 106758204,4 | 91324195,9 | 108541760,2 | 0,023 |
| TAG(14:0/14:0/24:0)_M+NH4 | 16072145,6 | 14104075,1 | 18234027,2 | 17246629,8 | 14710601,1 | 25316380,7 | 0,481 |
| TAG(14:0/15:0/18:1)_M+Na | 31427702,6 | 28410163,2 | 32414076,0 | 33010587,0 | 30069337,4 | 35690504,9 | 0,035 |
| TAG(14:0/15:0/18:1)_M+NH4 | 123114943,7 | 111741310,1 | 126590970,6 | 131497169,7 | 115911560,8 | 139194666,8 | 0,029 |
| TAG(14:0/15:0/18:2)_M+NH4 | 90428830,5 | 80016869,9 | 95084491,2 | 101294559,1 | 86678663,1 | 104746492,2 | 0,019 |
| TAG(14:0/15:0/20:0)_M+NH4 | 41555068,2 | 38695340,3 | 45620785,9 | 44761060,5 | 41094225,1 | 50654097,1 | 0,280 |
| TAG(14:0/15:0/20:0)_ | 10219861,6 | 9456685,7 | 11004064,8 | 10786822,2 | 9949563,9 | 13143575,5 | 0,353 |
| TAG(14:0/15:0/20:1)_M+Na | 23991863,0 | 22511282,1 | 25275261,3 | 25879961,1 | 23501887,1 | 28332983,9 | 0,075 |
| TAG(14:0/15:0/20:1)_M+NH4 | 91806141,9 | 84118487,2 | 94220789,8 | 97392135,4 | 87349337,2 | 105127645,9 | 0,025 |
| TAG(14:0/15:0/20:2)_M+Na | 28890970,3 | 25964546,1 | 30337070,9 | 31536231,2 | 27988343,7 | 33346779,4 | 0,035 |
| TAG(14:0/15:0/20:2)_M+NH4 | 105199669,9 | 94607456,9 | 107259855,2 | 111852358,9 | 99105143,7 | 119385589,5 | 0,052 |
| TAG(14:0/15:0/20:3)_M+NH4 | 41483496,4 | 37639447,0 | 43647432,2 | 46206922,9 | 39552355,0 | 48057565,3 | 0,023 |
| TAG(14:0/16:0/20:0)_M+NH4 | 40627134,1 | 38118036,7 | 45683245,7 | 44978694,6 | 39940690,4 | 54746422,0 | 0,247 |
| TAG(14:0/16:0/20:1)_M+Na | 58161702,9 | 52239636,9 | 62497167,6 | 59736018,8 | 56437170,0 | 65428336,0 | 0,393 |
| TAG(14:0/18:1/18:3)_M+NH4 | 8299368,6 | 7984766,2 | 8823809,7 | 9381437,1 | 8683085,1 | 9794459,8 | 0,063 |
| TAG(14:0/24:0/18:0)_M+NH4 | 5575433,0 | 5110562,0 | 5817157,8 | 6204199,3 | 5435918,4 | 6559194,8 | 0,023 |
| TAG(14:0/24:0/20:1)_M+NH4 | 6575812,0 | 5957842,5 | 6881429,2 | 7129184,0 | 6184181,4 | 7443233,4 | 0,023 |
| TAG(14:1/15:0/22:0)_M+NH4 | 33589842,4 | 30162027,2 | 35774656,1 | 33517951,6 | 32220201,4 | 38033413,1 | 0,393 |
| TAG(14:1/16:1/15:0)_M+NH4 | 16680516,1 | 15420696,4 | 17540412,9 | 18853679,2 | 15912810,5 | 19036345,7 | 0,029 |
| TAG(14:1/18:2/15:0)_M+NH4 | 11098596,9 | 9974111,5 | 11670036,9 | 12392234,8 | 10335271,3 | 13058367,9 | 0,043 |
| TAG(15:0/16:0/22:3)_M+NH4 | 21604550,5 | 20173095,0 | 22578677,5 | 21820247,4 | 20246798,9 | 25114166,4 | 0,393 |
| TAG(15:0/16:0/24:0)_M+NH4 | 6618480,4 | 5850847,3 | 6868748,3 | 7229434,5 | 6221143,9 | 7866724,7 | 0,043 |
| TAG(15:0/16:0/26:0)_M+NH4 | 3661477,9 | 3358180,5 | 3767122,8 | 4081885,1 | 3498050,8 | 4202930,9 | 0,052 |
| TAG(15:0/16:1/26:0)_M+NH4 | 7810106,9 | 6894606,9 | 7994037,2 | 8064083,1 | 7572899,9 | 8959714,8 | 0,105 |
| TAG(15:0/18:0/20:2)_M+NH4 | 23068821,0 | 20911199,6 | 24646903,6 | 23258846,8 | 21982698,3 | 26982942,2 | 0,436 |
| TAG(15:0/18:2/20:1)_M+NH4 | 21041882,1 | 19699055,4 | 22055419,0 | 21471424,6 | 19826421,1 | 24648029,7 | 0,280 |
| TAG(16:0/16:0/16:0)_M+NH4 | 93635581,9 | 83960933,8 | 96982560,6 | 95652897,0 | 88671770,0 | 102803110,1 | 0,315 |
| TAG(16:0/16:0/18:1)_M+Na | 58042484,0 | 52160264,2 | 62275423,8 | 59678327,7 | 56445360,9 | 65175172,5 | 0,436 |
| TAG(16:0/16:0/18:1)_M+NH4 | 280839144,8 | 250525650,2 | 302547562,2 | 287249116,1 | 256410827,0 | 311323180,1 | 0,631 |
| TAG(16:0/16:0/18:2)_M+NH4 | 234442317,1 | 220558129,7 | 249336223,0 | 252210545,3 | 227687139,5 | 272189205,7 | 0,165 |
| TAG(16:0/16:0/18:2)_M+Na | 61323541,7 | 57851461,2 | 65599648,5 | 66779579,5 | 60802448,0 | 71922745,2 | 0,089 |
| TAG(16:0/16:0/20:1)_M+NH4 | 119574535,9 | 101718464,6 | 129798717,5 | 124491821,0 | 104244607,5 | 131997797,6 | 0,796 |
| TAG(16:0/16:0/20:1)_M+Na | 8805584,8 | 7600868,0 | 10057837,3 | 9680347,3 | 7819324,1 | 10218014,5 | 0,631 |
| TAG(16:0/16:0/20:2)_M+NH4 | 1044587428,5 | 920190088,7 | 1140905988,5 | 1031687350,0 | 927797397,9 | 1150844307,5 | 0,912 |
| TAG(16:0/16:0/20:2)_M+Na | 206489997,6 | 186355639,2 | 225626862,9 | 211010103,5 | 192456991,6 | 230147072,6 | 0,529 |
| TAG(16:0/16:0/20:3)_M+NH4 | 408490761,4 | 376310990,4 | 423910653,8 | 427029618,7 | 390195024,1 | 472307824,6 | 0,315 |
| TAG(16:0/16:0/20:3)_M+Na | 102585332,9 | 95411292,2 | 108656980,5 | 108115173,3 | 100217490,0 | 120109262,0 | 0,190 |
| TAG(16:0/16:1/18:2)_M+NH4 | 113481764,7 | 105320960,8 | 116215388,2 | 121678324,4 | 109677961,9 | 127031515,2 | 0,063 |
| TAG(16:0/16:1/18:2)_M+Na | 22430529,6 | 20950731,8 | 22827458,6 | 24051276,8 | 22235381,6 | 25067536,2 | 0,105 |
| TAG(16:0/18:1/22:1)_M+Na | 6280426,2 | 4888278,8 | 6931844,7 | 5881472,5 | 4765784,1 | 6286982,6 | 0,529 |
| TAG(16:0/18:1/22:1)_M+NH4 | 27300501,2 | 21071864,7 | 29550550,3 | 25572199,8 | 21919341,7 | 27363702,9 | 0,579 |
| TAG(16:0/18:1/24:1)_M+NH4 | 10556811,3 | 9045009,1 | 11385911,0 | 10789188,0 | 9761310,2 | 11769274,7 | 0,393 |
| TAG(16:1/15:0/20:1)_M+NH4 | 62194659,3 | 57441321,8 | 63798383,4 | 65945178,7 | 59223299,7 | 71015365,1 | 0,143 |
| TAG(16:1/15:0/20:1)_M+Na | 15550516,9 | 14438805,3 | 16310114,3 | 16748710,5 | 15154872,6 | 17991879,8 | 0,075 |
| TAG(16:1/16:0/16:1)_M+NH4 | 174971523,1 | 159822042,6 | 181053400,4 | 188681264,4 | 168234367,5 | 198539144,5 | 0,029 |
| TAG(16:1/16:1/16:1)_M+Na | 11821750,2 | 10600416,5 | 12357024,3 | 13165216,9 | 11627429,8 | 13534421,4 | 0,011 |
| TAG(16:1/16:1/20:2)_M+NH4 | 129164708,7 | 120607942,6 | 134103544,4 | 137033543,6 | 123689931,1 | 143890545,4 | 0,143 |
| TAG(16:1/16:1/20:2)_M+Na | 32716322,0 | 31011897,7 | 34072292,1 | 34756094,8 | 31601707,8 | 37762177,8 | 0,143 |
| TAG(17:0/17:0/17:0)_M+Na | 359839298,7 | 214533401,7 | 558053077,0 | 432137973,1 | 301650489,8 | 732168995,6 | 0,353 |
| TAG(17:0/18:1/18:3)_M+NH4 | 4617113,6 | 4339121,1 | 4963142,1 | 4913266,3 | 4450813,1 | 5442758,4 | 0,190 |
| TAG(18:1/18:0/18:1)_M+NH4 | 265926618,0 | 222730345,4 | 297543187,3 | 266775465,0 | 229996184,3 | 299780020,0 | 0,912 |
| TAG(18:1/18:0/18:1)_M+Na | 26518441,5 | 21077630,6 | 29152495,2 | 26943406,5 | 21669421,8 | 28523205,5 | 0,912 |
| TAG(18:1/18:1/18:1)_M+NH4 | 1699718739,5 | 1488548561,0 | 1856768379,5 | 1692552834,0 | 1420574040,3 | 1885247776,8 | 1,000 |
| TAG(18:1/18:1/18:1)_M+Na | 326014616,4 | 298372431,4 | 356029010,3 | 328309272,6 | 303704411,9 | 371170314,9 | 0,579 |
| TAG(18:1/18:1/18:2)_M+NH4 | 514987535,5 | 470049648,4 | 526824210,4 | 539327100,1 | 479975756,6 | 599957510,9 | 0,315 |
| TAG(18:1/18:1/18:2)_M+Na | 124103759,8 | 114372247,5 | 128359474,2 | 132356058,0 | 118838402,3 | 147427577,1 | 0,190 |
| TAG(18:1/18:1/18:3)_M+Na | 51310891,7 | 47230761,6 | 52224998,7 | 53546027,3 | 49537721,3 | 58431181,6 | 0,105 |
| TAG(18:1/18:1/18:3)_M+NH4 | 195741670,6 | 178342909,1 | 200491405,8 | 202827332,1 | 184584884,0 | 218439602,0 | 0,143 |
| TAG(18:1/20:1/18:1)_M+NH4 | 31114681,1 | 25963357,5 | 33115717,7 | 30535497,8 | 26828914,4 | 33895018,0 | 0,912 |
| TAG(18:1/20:1/18:1)_M+Na | 1731275,2 | 1352583,2 | 1890357,0 | 1796331,7 | 1663481,8 | 1949701,3 | 0,579 |
| TAG(18:1/20:1/18:1)\|TAG(18:2/20:1/18:0)_M+NH4 | 32365418,1 | 27066261,8 | 34772727,7 | 31965988,8 | 28036425,9 | 35551734,7 | 0,912 |
| TAG(18:1/20:1/18:2)_M+NH4 | 13872006,1 | 12181644,3 | 15476147,9 | 13887209,2 | 13238942,2 | 15619791,8 | 0,853 |
| TAG(18:2/16:1/18:2)_M+Na | 2266800,1 | 2154686,7 | 2416304,3 | 2691851,7 | 2469094,1 | 2827217,9 | 0,019 |
| TAG(18:2/18:1/15:0)_M+NH4 | 17806845,0 | 16815043,1 | 19603399,1 | 19585066,9 | 17160320,6 | 20785626,3 | 0,165 |
| TAG(18:2/18:1/15:0)_M+Na | 5683827,1 | 5256363,7 | 5928676,1 | 6158630,4 | 5628507,0 | 6827292,9 | 0,043 |
| TAG(24:0/18:2/17:0)_M+NH4 | 3380687,0 | 2883929,5 | 3543544,8 | 3403389,8 | 3345488,6 | 3885938,6 | 0,165 |
| Total TAG | 8695712479,5 | 7816517192,8 | 9035933001,3 | 8687195469,5 | 8083463252,5 | 10042196934,0 | 0,529 |

**Table S6.** Lipid variables by mouse genotype with median, interquartile and p-value in negative ionization mode in the hippocampus.

| **Lipid variables** | **WT** | | | **TG** | | | **p value (Mann-Whitney)** |
| --- | --- | --- | --- | --- | --- | --- | --- |
|  | **Median** | **IQ1** | **IQ3** | **Median** | **IQ1** | **IQ3** |  |
| Cer(d18:1/16:0)_M-H | 54767758,8 | 45235218,3 | 79143209,0 | 62581194,1 | 53767180,2 | 73156496,2 | 0,579 |
| Cer(d18:1/16:0)_M+CH3COO | 955306353,9 | 836997321,6 | 1011765253,8 | 1130859676,5 | 918851650,6 | 1367357177,0 | 0,043 |
| Cer(d18:0_18:1)_M-H | 637903522,9 | 416252821,0 | 997224938,4 | 723871315,4 | 609703045,2 | 850879894,6 | 0,853 |
| Cer(d18:0_18:1)_M+CH3COO | 963261402,2 | 626189192,5 | 1499289456,5 | 1098419849,4 | 932483300,3 | 1287403056,5 | 0,853 |
| Cer(d18:1/20:0)_M-H | 77654009,2 | 63119837,7 | 83082261,7 | 73396027,7 | 57761295,3 | 79249813,6 | 0,315 |
| Cer(d18:1/20:0)_M+CH3COO | 135371363,5 | 112191847,8 | 144712488,8 | 129488881,0 | 103223519,0 | 140886926,7 | 0,436 |
| Cer(d18:1/24:0)_M-H | 11803112,1 | 9463417,6 | 13759622,6 | 11912290,6 | 9715018,6 | 13718967,0 | 1,000 |
| Cer(d18:1/24:0)_M+CH3COO | 27179011,0 | 23011434,1 | 31988998,5 | 27373441,3 | 23392945,8 | 32129144,9 | 1,000 |
| Cer(d18:1/24:1)_M-H | 120188631,6 | 109465746,3 | 144692600,0 | 115710714,0 | 101545204,0 | 146404460,7 | 0,684 |
| Cer(d18:1/24:1)_M+CH3COO | 263944302,7 | 243345058,9 | 317919459,6 | 257951164,3 | 228625076,5 | 323879600,2 | 0,796 |
| Total Cer | 3187996973,0 | 2685533387,5 | 4252340093,3 | 3663239249,5 | 3282257801,5 | 4087225282,5 | 0,315 |
| CL(16:1_18:1_20:4_20:4)_M-H | 16934869,0 | 12761139,7 | 21614510,8 | 15109052,7 | 11713411,0 | 17042487,5 | 0,218 |
| CL(16:1_18:1_20:4_22:6)_M-H | 10895457,7 | 9654172,3 | 13995377,7 | 10188433,8 | 8198159,6 | 11228633,3 | 0,123 |
| CL(18:1/18:1/18:1/18:1)_M-H | 27320288,1 | 22479040,6 | 39103848,1 | 26607877,0 | 16680856,4 | 34985402,9 | 0,353 |
| CL(18:1_18:1_18:1_18:2)_M-H | 17030738,4 | 10683339,8 | 24990217,8 | 15710946,9 | 12080027,1 | 21741982,5 | 0,739 |
| CL(18:1_18:1_18:1_20:4)_M-H | 34064135,9 | 26727307,3 | 60575374,3 | 31848303,8 | 23103782,5 | 52572266,4 | 0,481 |
| CL(18:1_18:1_18:1_22:6)_M-H | 26697699,0 | 21198074,0 | 37601278,7 | 21444900,4 | 17890324,9 | 32773438,3 | 0,247 |
| CL(18:1_18:1_20:4_20:4)_M-H | 37478683,5 | 27114572,3 | 53027177,9 | 28996250,2 | 24895553,1 | 46420924,7 | 0,315 |
| CL(18:1_18:1_20:4_22:6)_M-H | 30691634,5 | 26262332,2 | 38855009,7 | 25583291,3 | 22435391,9 | 35057897,3 | 0,105 |
| CL(18:1_18:1_22:6_22:6)_M-H | 15499550,3 | 14455513,5 | 17804236,4 | 13886083,2 | 12211977,1 | 15461783,0 | 0,023 |
| CL(18:1_18:2_22:6_22:6)_M-H | 13531589,1 | 12642952,6 | 15696920,2 | 12399922,9 | 9362980,7 | 13095679,7 | 0,052 |
| CL(18:1_20:4_22:6_22:6)_M-H | 8474567,9 | 7747222,2 | 9497929,4 | 6460896,5 | 5966343,6 | 7820207,0 | 0,011 |
| CL(18:2/18:2/18:2/18:2)_M-H | 7589417,1 | 4578797,2 | 9974148,2 | 6250062,6 | 4899472,5 | 7503714,7 | 0,315 |
| Total CL | 251783784,9 | 197695086,5 | 334491634,6 | 202527462,2 | 181359831,9 | 294964892,8 | 0,218 |
| FA(16:0)_M-H | 2427542603,0 | 1981662828,8 | 3065718043,3 | 2545390464,0 | 2236401002,8 | 3118476960,3 | 0,579 |
| FA(17:0)_M-H | 199939214,7 | 168476792,1 | 213457041,7 | 197865248,7 | 186594669,1 | 274541352,9 | 0,315 |
| FA(18:0)_M-H | 3841196075,0 | 3438604050,3 | 4208893718,0 | 3584131142,5 | 3282614808,3 | 4245889473,3 | 0,631 |
| FA(18:1)_M-H | 3871087859,0 | 3442724384,8 | 4440912395,0 | 4381599854,0 | 3584412702,8 | 5751756225,0 | 0,190 |
| FA(18:2)_M-H | 181523448,8 | 166832476,4 | 192313038,2 | 206634270,4 | 181164424,6 | 257254921,3 | 0,089 |
| FA(20:0)_M-H | 64730044,0 | 58004101,3 | 69393171,9 | 62642658,5 | 58410570,7 | 77319214,9 | 0,853 |
| FA(20:1)_M-H | 385491582,5 | 320593833,7 | 481724557,1 | 422914749,6 | 310203904,7 | 608636452,1 | 0,529 |
| FA(20:4)_M-H | 7526703970,5 | 6935293760,0 | 8481239658,0 | 8032249859,5 | 7237854058,0 | 10416573672,8 | 0,247 |
| FA(22:0)_M-H | 45660499,9 | 43074810,9 | 52235628,0 | 47772780,5 | 43022123,7 | 55553716,4 | 0,796 |
| FA(22:1)_M-H | 50611667,9 | 47588598,5 | 59062884,1 | 55365898,4 | 45374483,8 | 74322360,3 | 0,796 |
| FA(24:0)_M-H | 68144429,7 | 58192221,3 | 72889047,1 | 65354543,0 | 57963438,2 | 76753536,0 | 0,853 |
| FA(24:1)_M-H | 34709182,6 | 24330229,9 | 42617469,2 | 29652753,9 | 26952755,5 | 32451527,0 | 0,353 |
| FA(26:0)_M-H | 43447269,8 | 37027387,6 | 45659104,1 | 40903758,0 | 35220510,2 | 47399067,7 | 0,912 |
| Total FA | 18655561688,5 | 17117191369,8 | 21323545645,5 | 19596579703,0 | 17456299054,8 | 24966258135,0 | 0,529 |
| LPC(16:0)_M-CH3 | 41960767,8 | 37439442,9 | 47362613,8 | 50222220,0 | 40803804,4 | 56679514,3 | 0,052 |
| LPC(16:0)_M+CH3COO | 168806444,9 | 148915992,8 | 189850716,4 | 199963591,7 | 164485105,3 | 222776699,9 | 0,063 |
| LPC(16:1)_M-CH3 | 22322090,9 | 19842475,9 | 27633170,6 | 26528014,9 | 20902189,0 | 29027035,3 | 0,530 |
| LPC(18:0)_M-CH3 | 14633199,7 | 14171658,4 | 15734980,5 | 17027320,3 | 13376416,6 | 20865988,2 | 0,280 |
| LPC(18:0)_M+CH3COO | 68233158,1 | 64968924,4 | 73009009,2 | 79574525,9 | 62372473,4 | 95578161,2 | 0,280 |
| LPC(18:1)_M-CH3 | 11415937,4 | 10150511,8 | 13708756,3 | 13866750,0 | 11930210,5 | 16168434,6 | 0,052 |
| LPC(18:1)_M+CH3COO | 54309236,9 | 47810900,3 | 64276973,5 | 64740769,8 | 56686237,0 | 74922492,4 | 0,043 |
| LPC(20:1)_M+CH3COO | 3363193,6 | 2936386,9 | 3935282,4 | 3762899,7 | 3185273,3 | 4325952,5 | 0,579 |
| LPC(20:4)_M+CH3COO | 31609852,6 | 28616744,9 | 36566482,8 | 36542991,6 | 32060018,7 | 40962875,1 | 0,075 |
| LPC(22:6)_M+CH3COO | 22087446,9 | 18692858,3 | 27686914,1 | 24500862,7 | 18422517,2 | 28254326,7 | 0,853 |
| Total LPC | 441109879,6 | 401934010,3 | 495975100,6 | 511844822,6 | 438404943,0 | 577406953,2 | 0,105 |
| LPE(18:0)_M-H | 50966304,2 | 45078307,5 | 67468619,0 | 62808730,9 | 48601908,9 | 88467820,6 | 0,353 |
| LPE(20:4)_M-H | 52629170,6 | 46634334,0 | 59953849,8 | 58253956,5 | 49687605,3 | 65175852,7 | 0,165 |
| LPE(22:6)_M-H | 122762485,2 | 106563718,0 | 135171908,8 | 127800031,3 | 118878150,9 | 144361175,6 | 0,247 |
| Total LPE | 222478916,7 | 206401291,3 | 252241297,9 | 247465666,8 | 229118578,7 | 282193525,0 | 0,143 |
| LPI(18:0)_M-H | 66537319,2 | 54537249,5 | 72818815,1 | 67286393,9 | 61505312,2 | 80555714,0 | 0,579 |
| LPI(20:4)_M-H | 37135411,5 | 32109660,8 | 42741350,6 | 43561944,1 | 35701931,9 | 50888992,6 | 0,393 |
| Total LPI | 105152369,5 | 92335844,9 | 113713003,9 | 111512807,8 | 98863819,3 | 128756487,0 | 0,393 |
| LPS(18:0)_M-H | 11090388,4 | 10292866,9 | 11972468,0 | 11595763,4 | 9250610,8 | 13964077,5 | 1,000 |
| LPS(22:6)_M-H | 73796931,4 | 68311077,7 | 77685637,4 | 79400630,2 | 71531826,6 | 84338853,5 | 0,218 |
| Total LPS | 84994523,8 | 78796545,2 | 91212360,0 | 90028730,3 | 83642258,7 | 95051017,5 | 0,280 |
| PC(16:0/16:0)_M-CH3 | 109747859,5 | 102311798,5 | 134915878,4 | 112242407,1 | 98788259,7 | 125976350,2 | 1,000 |
| PC(16:0/16:0)_M+CH3COO-CH3 | 48893061,9 | 45499190,6 | 59403368,3 | 67127895,2 | 47199889,5 | 79067823,9 | 0,165 |
| PC(16:0_20:4)_M-CH3 | 62819857,4 | 57291840,5 | 64031453,2 | 67615344,7 | 59322142,5 | 74531238,6 | 0,063 |
| PC(16:0_20:4)_M+CH3COO | 739664338,4 | 688821611,2 | 754913987,9 | 780860198,1 | 701063331,1 | 851991910,9 | 0,218 |
| PC(16:0/22:4)_M+CH3COO | 54847465,5 | 50969325,5 | 59841021,6 | 56876554,1 | 52817295,5 | 61295411,0 | 0,684 |
| PC(18:0/16:1)_M+CH3COO | 67070468,6 | 57665147,1 | 72302841,7 | 60301953,7 | 55494138,4 | 74197211,2 | 0,631 |
| PC(16:0_18:1)_M+CH3COO // M+CH3COO | 66630855,6 | 57488262,9 | 72012283,4 | 60085502,9 | 55351105,7 | 73566801,5 | 0,684 |
| PC(16:1/22:6)_M+CH3COO | 7211872,4 | 6436729,2 | 7886559,0 | 5942257,8 | 5203283,7 | 10291957,3 | 0,853 |
| PC(17:0/16:0)_M+CH3COO | 14977414,4 | 13001217,6 | 18118096,2 | 13492186,9 | 12769625,8 | 15014861,5 | 0,190 |
| PC(17:0/18:1)_M+CH3COO | 20789388,9 | 19262164,1 | 21676872,4 | 19511839,3 | 18206326,0 | 22288520,4 | 0,579 |
| PC(18:0/18:1)_M-CH3 | 90405596,2 | 86253853,7 | 95582248,8 | 91397941,9 | 82394117,9 | 99985031,7 | 0,912 |
| PC(18:0/18:1)_M+CH3COO | 1054703981,0 | 1023366813,8 | 1133826707,3 | 1085185039,0 | 970563105,6 | 1175476626,0 | 1,000 |
| PC(18:0/20:4)_M-CH3 | 62059640,8 | 56614414,7 | 68747975,0 | 64531489,0 | 59349781,5 | 73305343,6 | 0,436 |
| PC(18:0/20:4)_M+CH3COO | 722195474,1 | 644038855,0 | 763017564,5 | 750351632,8 | 684946480,1 | 817256347,0 | 0,529 |
| PC(18:0/22:4)_M+CH3COO | 38490680,4 | 33503260,2 | 39571992,1 | 34401224,0 | 32776573,2 | 37340899,5 | 0,315 |
| PC(18:0/22:5)_M+CH3COO | 39215862,6 | 34825398,2 | 47659864,1 | 41279305,1 | 36600849,9 | 45266641,4 | 0,436 |
| PC(18:0/22:6)_M+CH3COO | 173835190,7 | 126864869,0 | 204277541,8 | 132918884,7 | 108909398,5 | 204631125,9 | 0,529 |
| PC(18:1/18:1)_M-CH3 | 22458879,6 | 18428496,6 | 25069542,3 | 19692375,0 | 17280754,5 | 26659542,6 | 0,853 |
| PC(18:1/18:1)_M+CH3COO | 324909237,6 | 275781138,5 | 359029036,2 | 308008371,1 | 260319448,6 | 381198161,0 | 0,971 |
| PC(18:1/20:4)_M+CH3COO | 126427920,8 | 107359089,8 | 138717232,9 | 144436808,2 | 110500011,6 | 152047617,4 | 0,579 |
| PC(18:1_22:0)_M+CH3COO | 43523978,4 | 40639936,1 | 57318740,6 | 50737493,6 | 33012890,3 | 58330921,4 | 0,796 |
| PC(22:6/22:6)_M+CH3COO | 29370467,0 | 19752002,3 | 48849724,6 | 27382299,1 | 18029744,9 | 41006954,3 | 0,393 |
| PC(30:0)_M-CH3 | 2313045,0 | 2055364,2 | 2802162,5 | 1976884,9 | 1877146,9 | 2284619,7 | 0,052 |
| PC(30:0)_M+CH3COO | 25009203,7 | 21026039,7 | 30973053,2 | 23195171,7 | 20631087,4 | 25210899,6 | 0,436 |
| PC(32:0)_M+CH3COO | 1188969440,0 | 1122989462,8 | 1462974497,3 | 1208234780,5 | 1083960396,8 | 1357752255,3 | 1,000 |
| PC(32:1)_M+CH3COO | 110037101,6 | 101542574,4 | 120412767,0 | 108824013,5 | 94027797,7 | 121760583,8 | 0,912 |
| PC(33:1)_M+CH3COO | 15981667,4 | 13960898,4 | 17014736,3 | 14267639,7 | 13459787,0 | 15723308,7 | 0,105 |
| PC(34:1)_M-CH3 | 209882025,1 | 194731063,5 | 220693271,6 | 208300351,1 | 199471712,2 | 221815679,1 | 1,000 |
| PC(34:1)_M+CH3COO | 2310821393,5 | 2140802023,5 | 2438301154,3 | 2306563880,5 | 2213956522,8 | 2454360973,8 | 1,000 |
| PC(34:2)_M+CH3COO | 37279302,0 | 33238260,1 | 40892949,3 | 36944229,6 | 31744902,9 | 38564856,9 | 0,579 |
| PC(34:5)_M-CH3 | 9367150,3 | 8386573,7 | 9965684,4 | 9244730,0 | 8493640,9 | 10633144,9 | 0,853 |
| PC(36:2)_M+CH3COO | 45898434,1 | 44157789,3 | 48837146,6 | 46596008,9 | 42357015,4 | 49131101,7 | 0,853 |
| PC(36:2)_M-CH3 | 47242550,1 | 42122189,6 | 62775569,5 | 54494341,3 | 46102173,6 | 67678626,6 | 0,436 |
| PC(36:3)_M+CH3COO | 13988564,7 | 11431683,3 | 14642379,2 | 13097551,4 | 11178458,1 | 14721169,1 | 0,739 |
| PC(16:0_20:3)_M+CH3COO | 32532909,5 | 28627263,5 | 34785957,7 | 31972880,5 | 28067588,7 | 34814799,5 | 0,796 |
| PC(37:4)_M+CH3COO | 8496990,7 | 7956624,8 | 8966822,8 | 8566330,7 | 7909903,2 | 9041783,4 | 0,796 |
| PC(38:2)_M+CH3COO | 33606016,1 | 27401910,4 | 38731800,4 | 31137607,3 | 29628568,4 | 33609327,4 | 0,315 |
| PC(38:3)_M+CH3COO | 12291603,5 | 11454912,2 | 13150661,9 | 12210596,3 | 11341792,1 | 13221770,5 | 1,000 |
| PC(38:6)_M+CH3COO | 407263182,1 | 353312036,8 | 472357644,5 | 383799104,1 | 342317551,7 | 476749378,5 | 0,971 |
| PC(40:5)_M+CH3COO | 7479218,1 | 6854372,4 | 9088473,7 | 7976120,4 | 6523641,8 | 9398748,9 | 0,739 |
| PC(40:6)_M-CH3 | 11749315,9 | 8988149,0 | 14204002,0 | 9455070,6 | 7917686,3 | 13780027,1 | 0,529 |
| PC(40:7)_M+CH3COO | 167113456,2 | 141488656,8 | 202462943,0 | 133185998,7 | 109555294,5 | 214863045,7 | 0,631 |
| PC(42:10)_M+CH3COO | 41725179,0 | 34727650,0 | 43635384,8 | 41482959,4 | 37594205,8 | 47270525,5 | 0,481 |
| PC(42:7)_M+CH3COO | 5365129,9 | 3567087,4 | 7098960,1 | 3894940,5 | 2862488,2 | 6890277,6 | 0,280 |
| PC(42:8)_M+CH3COO | 3422624,9 | 3118990,8 | 3797588,9 | 3905794,2 | 3256331,1 | 4435206,0 | 0,190 |
| PC(44:10)_M+CH3COO | 2543199,4 | 2190348,0 | 2609545,5 | 2220371,4 | 2036631,4 | 2480596,2 | 0,315 |
| Total PC | 8591125958,0 | 8403597121,0 | 9262924033,8 | 8630325099,0 | 8250649074,3 | 9397882320,0 | 0,912 |
| PCo(34:2)\|PCp(34:1)_M+CH3COO | 11242662,7 | 10693233,5 | 12394601,4 | 11869034,1 | 10589650,4 | 13356568,3 | 0,739 |
| PCo(34:1)\|PCp(34:0)_M+CH3COO | 38379548,2 | 34779253,6 | 41962917,0 | 39106525,0 | 35393367,9 | 42550905,0 | 1,000 |
| Total Pco | 50376475,2 | 45502266,1 | 53083995,2 | 51071524,5 | 45830100,9 | 54667152,3 | 0,912 |
| PE(16:0/20:3)_M-H | 9618425,1 | 9374505,1 | 9967680,1 | 9464190,0 | 8259082,4 | 9965925,4 | 0,315 |
| PE(16:0/20:4)_M-H | 133877428,1 | 122824224,0 | 144279889,3 | 123964605,6 | 99120013,4 | 143158179,9 | 0,353 |
| PE(16:0/22:4)_M-H | 125951088,1 | 119082178,6 | 148589793,3 | 120819386,4 | 101494881,2 | 149823573,1 | 0,436 |
| PE(16:0/22:5)_M-H | 36217830,1 | 28034105,3 | 39501586,4 | 35416640,3 | 31863916,3 | 41940910,0 | 0,436 |
| PE(16:0/22:6)_M-H | 791020210,0 | 707686209,7 | 828083877,8 | 760912360,3 | 710943169,4 | 852658801,8 | 0,853 |
| PE(18:0_18:1)_M-H | 941481740,8 | 853790516,4 | 1205806951,0 | 1081950734,5 | 889977029,7 | 1162507062,3 | 0,739 |
| PE(18:0/18:2)_M-H | 19924418,7 | 19591970,2 | 20706378,7 | 19011211,5 | 17983228,0 | 21075860,1 | 0,280 |
| PE(18:0/20:4)_M-H | 1786428775,0 | 1586033781,0 | 1926051397,5 | 1620524671,0 | 1543055468,0 | 2027750618,5 | 0,971 |
| PE(18:0/22:6)_M-H | 2744620612,0 | 2623360682,3 | 2892614747,8 | 2505544560,5 | 2285944883,0 | 2632353105,5 | 0,019 |
| PE(18:1/18:1)_M-H | 309963853,9 | 234572981,7 | 340905139,2 | 255238445,4 | 238030871,0 | 291277294,4 | 0,280 |
| PE(18:1/20:4)_M-H | 422029946,8 | 360190327,6 | 446280231,4 | 398800142,0 | 368571956,3 | 420500104,8 | 0,315 |
| PE(18:1_18:2)_M-H | 19719977,6 | 17932097,3 | 20361975,7 | 19138528,5 | 15153030,4 | 21369450,9 | 0,436 |
| PE(34:2)_M-H | 29411314,8 | 27903894,6 | 36840944,3 | 32792162,3 | 27876712,5 | 41323423,2 | 0,481 |
| PE(38:2)_M-H | 42365478,2 | 37929072,8 | 57529370,2 | 50621487,7 | 41529480,7 | 61813386,6 | 0,393 |
| Total PE | 7406730686,5 | 7328540615,3 | 7595112137,0 | 7358107620,0 | 6692883925,3 | 7512127970,3 | 0,247 |
| PEo(36:4)\|PEp(36:3)_M-H | 42830440,2 | 32023411,6 | 51113991,4 | 32251346,0 | 29089924,7 | 36789303,7 | 0,023 |
| PEo(38:6)\|PEp(38:5)_M-H | 125556103,0 | 110233240,1 | 131787721,0 | 124988608,6 | 103336489,2 | 139779168,6 | 0,912 |
| PEo(32:2)\|PEp(32:1)_M-H | 8630706,2 | 6797545,9 | 9329629,0 | 7357799,4 | 7155498,0 | 7820146,4 | 0,218 |
| PEo(16:1/20:3)\|PEp(16:0/20:3)_M-H | 29821224,4 | 24354053,2 | 37482816,9 | 27072268,1 | 25264352,6 | 38751559,7 | 0,853 |
| PEo(16:1/20:4)\|PEp(16:0/20:4)_M-H | 441996117,4 | 312075860,1 | 506545968,8 | 322496440,1 | 251555527,3 | 393436205,9 | 0,063 |
| PEo(16:1/20:4)\|PEp(16:0/20:4)_M+NaCH3COO | 19471037,6 | 15560580,1 | 22771049,2 | 14912573,3 | 11811992,6 | 19648960,9 | 0,075 |
| PEo(16:1/22:5)PEp(16:0/22:5)_M-H | 748250097,2 | 704979322,8 | 839224215,2 | 732139268,0 | 681026160,5 | 797415980,7 | 0,393 |
| PEo(16:1/22:5)\|PEp(16:0/22:5)_M-H | 58148875,5 | 52933499,2 | 68674502,6 | 65083290,9 | 56767034,8 | 87268660,6 | 0,280 |
| PEo(16:1/22:6)\|PEp(16:0_22:6)_M+NaCH3COO | 21345016,9 | 18408677,0 | 24301054,9 | 21684263,7 | 19110690,2 | 24601874,1 | 0,684 |
| PEo(16:1_22:6)\|PEp(16:0_22:6)_M-H | 948934167,6 | 880147565,4 | 1079085143,5 | 1072393156,5 | 812239014,8 | 1092553845,8 | 0,481 |
| PEo(16:1_22:6)\|PEp(16:0_22:6)_M+NaCH3COO | 21308227,5 | 18378875,0 | 24269812,6 | 21652523,1 | 19065494,6 | 24554083,8 | 0,684 |
| PEo(18:1/22:6)\|PEp(18:0/22:6)_M-H // M-H | 39057186,3 | 37880678,0 | 41580508,4 | 38377420,7 | 34111565,4 | 40700870,3 | 0,353 |
| PEo(18:1/22:6)\|PEp(18:0/22:6)_M+NaCH3COO | 36627131,4 | 35142704,8 | 39670090,9 | 37810600,5 | 34521379,9 | 40273858,1 | 0,912 |
| PEo(18:1_22:6)\|PEp(18:0_22:6)_M-H | 1673541351,5 | 1544506363,5 | 1799108658,3 | 1699975276,5 | 1360386593,5 | 1755350015,8 | 0,796 |
| PEo(18:1_22:6)\|PEp(18:0_22:6)_M+NaCH3COO | 36635033,3 | 35255803,6 | 39676196,4 | 37820063,8 | 34521379,9 | 40281093,2 | 0,912 |
| PEo(18:2/16:1)\|PEp(18:1/16:1)_M-H | 46177557,9 | 41348360,8 | 53159706,3 | 42784390,9 | 39987120,0 | 44874970,6 | 0,089 |
| PEo(18:2/18:1)\|PEp(18:1/18:1)_M-H | 974226085,2 | 856619278,1 | 1091431923,3 | 919198673,8 | 852294660,9 | 1028904482,0 | 0,631 |
| PEo(18:2/18:2)\|PEp(18:1_18:2)\|PEp(18:2/18:1)_M-H | 26274077,4 | 22248442,3 | 28576836,5 | 22318034,5 | 20576260,2 | 26576007,3 | 0,143 |
| PEo(18:2/20:4)\|PEp(18:1/20:4)_M+NaCH3COO | 14654062,7 | 13628949,3 | 16095160,4 | 14336904,7 | 13827337,0 | 14925838,2 | 0,631 |
| PEo(18:2/22:6)\|PEp(18:1/22:6)_M-H | 622639848,9 | 581744347,5 | 693111466,5 | 667261010,1 | 592539507,9 | 714410569,6 | 0,436 |
| PEo(18:2/22:6)\|PEp(18:1/22:6)_M+NaCH3COO | 19460635,5 | 17647522,9 | 20691494,2 | 19863356,4 | 18192106,5 | 20668718,3 | 0,684 |
| PEo(35:2)\|PEp(17:0/18:1)_M-H | 44910108,6 | 37630095,0 | 50645129,8 | 40960877,6 | 34409736,4 | 49273679,3 | 0,481 |
| PEo(37:3)\|PEp(37:2)_M-H | 8390193,8 | 7146964,3 | 10946658,7 | 7645550,5 | 6768947,7 | 10730714,9 | 0,684 |
| PEo(38:5)\|PEp(38:4)_M-H | 295620234,3 | 265942910,1 | 353444576,7 | 298803683,7 | 252121345,0 | 341412046,3 | 0,684 |
| PEo(38:5)\|PEp(38:4)_M+NaCH3COO | 4133189,3 | 3730424,2 | 4630094,3 | 4429217,0 | 3869110,5 | 5119270,3 | 0,529 |
| PEo(39:5)\|PEp(17:0/22:4)_M-H | 11158286,4 | 9812841,6 | 13247267,1 | 11885181,5 | 8540301,3 | 13624059,5 | 1,000 |
| PEp(17:0/22:6)_M-H | 69917162,4 | 57037907,4 | 77006791,8 | 61272656,3 | 46169995,7 | 69976266,7 | 0,190 |
| Total PEo/Pep | 6415642475,0 | 6201075335,5 | 6709689084,8 | 6416091533,0 | 5510848220,0 | 6626069761,5 | 0,796 |
| PG(18:1/18:1)_M-H | 15394037,1 | 9979037,3 | 17176521,4 | 10273753,2 | 9126031,7 | 14141545,6 | 0,165 |
| PG(22:6/22:6)_M-H | 160803010,1 | 153636248,3 | 175111666,5 | 124238170,4 | 102778221,8 | 158722939,4 | 0,043 |
| PG(34:1)_M-H | 156586632,0 | 91945572,3 | 167422263,6 | 92647144,0 | 81425275,0 | 121556864,9 | 0,089 |
| PG(36:4)_M-H | 37718541,5 | 34790782,3 | 44189849,4 | 28329145,5 | 24232176,6 | 50362329,3 | 0,481 |
| PG(38:4)_M-H | 46021175,7 | 38730616,8 | 50793319,0 | 25979722,6 | 23419574,5 | 56098163,2 | 0,315 |
| PG(38:5)_M-H | 20100908,1 | 17566620,5 | 22290547,2 | 17318358,1 | 13354877,1 | 21878486,1 | 0,315 |
| PG(38:6)_M-H | 9292533,1 | 6558034,0 | 11568796,9 | 6171867,7 | 5377280,1 | 10861614,2 | 0,353 |
| Total PG | 449026966,0 | 353499870,3 | 482042234,0 | 311843910,2 | 272240332,1 | 413023823,6 | 0,035 |
| PI(16:0/20:4)_M-H | 395010162,7 | 333973023,6 | 468621415,4 | 310327086,3 | 293406354,3 | 398347035,7 | 0,218 |
| PI(18:0/20:4)_M-H | 2067333280,0 | 1972487628,0 | 2237474768,0 | 1956715902,0 | 1737585825,5 | 2026418056,5 | 0,075 |
| PI(18:0/22:6)_M-H | 88885855,7 | 38545088,8 | 114083906,1 | 58038311,1 | 37000031,9 | 80314048,7 | 0,315 |
| PI(18:1/18:1)_M-H | 25385707,7 | 18121090,5 | 29376261,0 | 19073461,6 | 16938528,4 | 23076968,4 | 0,190 |
| PI(18:1/20:4)_M-H | 318480606,7 | 307404967,7 | 343716198,3 | 305505804,6 | 277798018,3 | 321408785,9 | 0,280 |
| Total PI | 2892552294,5 | 2709095160,0 | 3178597010,5 | 2651800791,5 | 2429578990,8 | 2796427573,3 | 0,105 |
| PS(34:1)_M-H | 76626751,3 | 70536708,6 | 78223195,9 | 72702640,6 | 67615586,3 | 76571705,3 | 0,315 |
| PS(38:6)_M-H | 42259880,7 | 38319401,3 | 44773281,3 | 42252357,9 | 36252570,0 | 47833533,8 | 0,912 |
| PS(39:6)_M-H | 4028238,3 | 3396463,6 | 4588434,2 | 3934498,3 | 3570555,4 | 4133979,7 | 0,853 |
| PS(18:0/18:1)_M-H | 952948318,6 | 932172503,4 | 1061745180,3 | 976990533,2 | 794547857,4 | 1095640409,3 | 0,796 |
| PS(38:3)_M-H | 26753511,2 | 24403367,6 | 28967379,2 | 28327459,5 | 24576499,8 | 31130497,2 | 0,529 |
| PS(18:0/20:4)_M+Na-2H | 14457626,9 | 13296906,3 | 15570618,9 | 14229060,9 | 13016489,3 | 18951936,4 | 0,853 |
| PS(18:0/20:4)_M-H | 472161358,7 | 437576271,2 | 506116613,9 | 466956323,6 | 401318802,1 | 495566543,2 | 0,631 |
| PS(18:0/22:4)_M-H | 884895606,5 | 756490778,2 | 955326663,2 | 866357163,2 | 676578637,6 | 959570991,9 | 0,631 |
| PS(18:0/22:5)_M-H | 97378907,3 | 89608886,8 | 105499806,6 | 101631019,4 | 92300421,9 | 116771768,0 | 0,315 |
| PS(18:0/22:6)_M-H | 3282370542,0 | 2955638331,5 | 3324952798,0 | 3054347505,5 | 2763351541,5 | 3554675858,5 | 0,684 |
| PS(18:0/22:6)_M+Na-2H | 40567130,6 | 36807267,2 | 45500232,6 | 44738642,4 | 39190269,2 | 49739610,1 | 0,280 |
| PS(18:1/18:1)_M-H | 356663151,6 | 226584301,0 | 413640310,5 | 265288093,7 | 250574359,6 | 339450679,7 | 0,315 |
| PS(18:1/20:4)_M-H | 62255263,2 | 49693023,8 | 72769077,3 | 56475254,0 | 53721848,3 | 61775544,4 | 0,684 |
| PS(18:1/22:6)_M-H | 9917774,1 | 8690415,3 | 10611613,1 | 9757851,3 | 8253261,5 | 11221465,5 | 0,971 |
| PS(18:1/22:6))_M-H | 145927812,4 | 132206073,8 | 153593718,3 | 141838806,0 | 126271921,7 | 162068323,0 | 1,000 |
| PS(18:1_20:1)_M-H | 5983056,4 | 5399843,9 | 7478416,4 | 7368221,3 | 4824465,3 | 7839828,0 | 0,579 |
| PS(22:4/22:6)_M-H | 203879288,6 | 176339468,2 | 233537963,9 | 196375823,5 | 184733192,3 | 213172279,7 | 0,796 |
| PS(22:6/22:6)_M-H | 126342242,9 | 97400712,2 | 163650343,7 | 118458941,3 | 100425685,8 | 147231095,6 | 0,684 |
| PS(22:6/22:6)_M+Na-2H | 4423035,7 | 3467589,4 | 5968070,9 | 3850905,3 | 3588722,6 | 5357821,1 | 0,796 |
| PS(36:1)_M-H | 964678022,9 | 944959723,8 | 1069570846,2 | 984022030,7 | 795706910,4 | 1110261896,8 | 0,739 |
| PS(36:4)_M-H | 14378870,4 | 12964663,4 | 15246732,5 | 13185383,9 | 12335691,5 | 14824809,5 | 0,315 |
| PS(40:2)_M-H | 15068137,4 | 14302913,7 | 15954683,0 | 15095707,1 | 12287282,3 | 19673462,4 | 0,971 |
| PS(40:7)_M+Na-2H | 2507843,7 | 2017528,2 | 2698291,3 | 2498413,6 | 2264774,0 | 2764771,8 | 0,796 |
| PS(42:10)_M-H | 29550140,2 | 26940293,9 | 35339237,9 | 33255620,0 | 31194971,9 | 35732879,1 | 0,143 |
| PS(42:5)_M-H | 7941317,1 | 7564839,3 | 9989767,7 | 10404755,3 | 8760364,9 | 11737791,1 | 0,019 |
| PS(42:7)_M-H | 3730844,6 | 3224635,3 | 4427370,2 | 4010776,7 | 3458151,2 | 5509070,3 | 0,436 |
| PS(44:10)_M+Na-2H | 7209105,4 | 6022205,7 | 7917873,4 | 6632403,9 | 6225336,9 | 7355578,5 | 0,796 |
| PS(44:11)_M-H | 10349234,6 | 8731824,8 | 13191470,6 | 9828183,8 | 9310614,5 | 13361404,3 | 0,853 |
| PS(44:7)_M+Na-2H | 5038403,7 | 4783997,7 | 5689577,4 | 5378740,6 | 4778026,2 | 5567561,0 | 0,912 |
| Total PS | 7907943124,5 | 7272816018,5 | 8188335854,8 | 7492599041,0 | 7188813590,5 | 7628343726,5 | 0,089 |

**Table S7.** Lipid variables by mouse genotype with median, interquartile and p-value in positive ionization mode in the cortex.

| **Lipid variables** | **WT** | | | **TG** | | | **p value (Mann-Whitney)** |
| --- | --- | --- | --- | --- | --- | --- | --- |
|  | **Median** | **IQ1** | **IQ3** | **Median** | **IQ1** | **IQ3** |  |
| CE(20:0)_M+NH4 | 5605231,0 | 5217519,4 | 6385902,6 | 6037010,4 | 5590348,5 | 6520337,9 | 0,353 |
| Cer(d16:1/17:0)_M+H-H2O | 9793686,6 | 9238647,3 | 10730178,8 | 9760204,4 | 9268532,7 | 10469472,1 | 0,912 |
| Cer(d16:1/17:0)_M+H | 8557789,6 | 8023537,8 | 8987728,3 | 8724391,4 | 8062882,1 | 9297909,6 | 0,684 |
| Cer(d18:1/17:0)_M+Na | 450225597,2 | 433008735,4 | 492036183,5 | 500834530,7 | 442853496,0 | 533663340,7 | 0,353 |
| Cer(d18:1/18:0)_M+H-H2O | 128788701,7 | 92264338,6 | 150199498,3 | 120178116,3 | 98238964,6 | 137732346,0 | 0,529 |
| Cer(d18:1/18:0)_M+H | 163805559,4 | 117664482,7 | 189160569,3 | 152239700,2 | 128905039,9 | 176149336,1 | 0,529 |
| Cer(d18:1/18:0)_M+Na | 86416832,0 | 67209570,5 | 110767849,5 | 79759132,3 | 59944838,6 | 97328995,4 | 0,631 |
| Cer(d18:1/18:1)_M+H-H2O | 26463916,8 | 21479189,5 | 28818151,4 | 24297475,7 | 19292027,1 | 27176343,2 | 0,481 |
| Cer(d18:1/18:1)_M+H | 33495665,5 | 28045899,3 | 37265297,0 | 31682656,1 | 24655535,2 | 36013386,7 | 0,481 |
| Cer(d18:1/18:1)_M+Na | 12334349,9 | 9651206,0 | 13200905,5 | 11256647,1 | 9213851,8 | 11942411,2 | 0,247 |
| Cer(d18:1/22:0)_M+H | 4885464,3 | 4544452,3 | 5151733,8 | 4569676,3 | 4078238,5 | 5488749,3 | 0,684 |
| Cer(d18:1/24:1)_M+H-H2O | 13670346,5 | 12721538,9 | 15074797,7 | 13184884,3 | 11003741,0 | 15675875,7 | 0,529 |
| Cer(d18:1/24:1)_M+H | 41679576,8 | 38699593,9 | 46642946,8 | 38568956,1 | 33796578,7 | 46571347,2 | 0,684 |
| Total Cer | 943888954,9 | 832912815,4 | 1024645427,3 | 934311412,1 | 879370448,9 | 999308456,8 | 1,000 |
| DAG(16:0/16:0)_M+H-H2O | 11222663,3 | 10402252,5 | 11487091,9 | 10929111,3 | 9871802,3 | 11924132,8 | 0,579 |
| DAG(16:0/18:1)_M+H-H2O | 36310770,1 | 33247548,3 | 38177256,4 | 35194871,7 | 27933047,5 | 37750624,9 | 0,529 |
| DAG(16:0/18:1)_M+NH4 | 33112785,7 | 29299691,3 | 36949368,8 | 28269668,8 | 24707043,3 | 35702119,7 | 0,280 |
| DAG(16:0/18:2)_M+H-H2O | 9149323,8 | 8853367,4 | 9469913,9 | 8891625,4 | 8225088,0 | 10029572,5 | 0,739 |
| DAG(16:0/20:4)_M+NH4 | 11648512,5 | 9661129,5 | 12862147,4 | 9204644,3 | 8938777,2 | 12426238,5 | 0,165 |
| DAG(16:0/20:4)_M+Na | 9063179,4 | 7538235,9 | 10361178,1 | 7642840,3 | 7216334,4 | 9459600,6 | 0,247 |
| DAG(16:0/22:1)_M+NH4 | 7260402,9 | 6899972,4 | 7856074,8 | 7042403,8 | 6783782,0 | 8123676,5 | 0,796 |
| DAG(16:0/22:6)_M+H-H2O | 39317592,3 | 34772033,1 | 42052283,2 | 36409627,8 | 33152278,7 | 42566370,3 | 0,579 |
| DAG(18:0/18:0)_M+H-H2O | 2976992,6 | 2831817,1 | 3131675,2 | 3275227,9 | 2840242,5 | 3450132,7 | 0,280 |
| DAG(18:0/18:0)_M+NH4 | 7077611,3 | 6863931,4 | 7497187,9 | 7661609,0 | 7132103,9 | 8407502,3 | 0,052 |
| DAG(18:0/18:0)_M+Na | 11566726,1 | 10803345,1 | 12401556,4 | 11942625,3 | 11442510,6 | 13786546,3 | 0,143 |
| DAG(18:0/20:4)_M+H-H2O | 61846717,8 | 52844266,3 | 68527052,3 | 62319071,0 | 51797226,5 | 70883146,6 | 0,971 |
| DAG(18:0/20:4)_M+NH4 | 275045416,2 | 233291746,7 | 298563603,6 | 235989348,4 | 217791208,0 | 287738793,4 | 0,247 |
| DAG(18:0/20:4)_M+Na | 136665382,0 | 120895217,2 | 147156948,0 | 120628577,7 | 113292455,7 | 140160190,0 | 0,315 |
| DAG(18:0/22:4)_M+NH4 | 13271977,1 | 11884654,8 | 14577681,6 | 11871940,9 | 10596483,0 | 14197104,5 | 0,393 |
| DAG(18:0/22:4)_M+Na | 7278683,1 | 6436453,8 | 8045743,5 | 6678072,8 | 5824532,2 | 7689256,7 | 0,529 |
| DAG(18:0/22:6)_M+H-H2O | 132069673,1 | 112695571,0 | 144370837,4 | 130836177,1 | 117136964,7 | 145239844,2 | 0,971 |
| DAG(18:1/16:0)_M+H-H2O | 42088950,2 | 39359492,0 | 44280500,8 | 41110931,9 | 33808035,0 | 44046611,4 | 0,579 |
| DAG(18:1/18:1)_M+H-H2O | 92062680,6 | 85501387,9 | 96368150,1 | 90648216,9 | 74566942,9 | 98379282,7 | 0,912 |
| DAG(18:1/18:1)_M+NH4 | 89043923,9 | 84681711,0 | 99118466,7 | 91884044,5 | 71381483,5 | 101677367,5 | 1,000 |
| DAG(18:1/18:1)_M+Na | 48398615,7 | 45012316,2 | 52438722,7 | 49353858,6 | 38395849,5 | 53089933,7 | 0,971 |
| DAG(18:1/18:2)_M+H-H2O | 14621805,8 | 14161349,6 | 15555389,6 | 14470946,7 | 13111151,8 | 16031488,3 | 0,684 |
| DAG(18:2/18:2)_M+H-H2O | 1477779,1 | 1457834,4 | 1586665,4 | 1571023,8 | 1371550,0 | 1686853,9 | 0,631 |
| Total DAG | 1076158750,5 | 994528829,5 | 1167365290,8 | 1008555899,4 | 953341337,3 | 1131197233,8 | 0,353 |
| LPC(16:0)_M+H | 332661987,8 | 317080649,4 | 368317198,5 | 306608786,2 | 290470922,0 | 319656762,3 | 0,015 |
| LPC(18:0)_M+H | 85500022,0 | 81321149,3 | 90016972,8 | 81213414,0 | 70554704,6 | 86864995,8 | 0,280 |
| LPC(18:1)_M+H | 121115906,2 | 105437640,2 | 127309681,2 | 107605595,5 | 96552696,0 | 118286031,0 | 0,089 |
| LPC(20:4)_M+H | 56670426,4 | 48906619,6 | 59130210,9 | 47523140,1 | 43915421,8 | 52216714,1 | 0,011 |
| LPC(22:6)_M+H | 48244549,7 | 42299532,3 | 52272153,7 | 41792091,9 | 40486997,0 | 45442714,4 | 0,043 |
| Total LPC | 650379443,2 | 596886826,9 | 680227646,4 | 595807155,7 | 559698037,4 | 609428814,7 | 0,019 |
| LPE(22:6)_M+H | 40571183,9 | 38448425,5 | 43074595,2 | 37236186,6 | 34887639,1 | 40855575,7 | 0,052 |
| MAG(18:2)_M+NH4 | 7898084,2 | 7110643,0 | 9515671,4 | 7759412,8 | 6075680,6 | 9568125,6 | 0,796 |
| MAG(20:1)_M+NH4 | 2072402,6 | 1273737,0 | 2802826,4 | 3548265,1 | 2061522,6 | 3967975,3 | 0,052 |
| MAG(20:2)_M+NH4 | 24371243,2 | 22491318,6 | 26855385,0 | 24849171,9 | 20656806,5 | 29209844,1 | 1,000 |
| MAG(20:4)_M+H-H2O | 3804384,4 | 3299704,4 | 4261715,7 | 3292836,4 | 3096932,8 | 4145306,3 | 0,247 |
| MAG(22:2)_M+NH4 | 12796051,8 | 12545470,0 | 14244370,6 | 12987977,5 | 11559919,3 | 13665835,4 | 0,796 |
| MAG(22:3)_ M+NH4 | 16729934,1 | 15237720,4 | 18194211,6 | 14978131,0 | 13098241,9 | 21061059,6 | 0,579 |
| MAG(22:4)_M+NH4 | 16750415,7 | 15256035,0 | 18257117,7 | 14967192,5 | 13014940,0 | 21172147,9 | 0,631 |
| Total MAG | 86008555,4 | 79815082,7 | 94037076,3 | 82640073,4 | 70818848,1 | 99574515,9 | 0,631 |
| PC(14:0_16:0)_M+H | 137698704,9 | 117348535,8 | 151938716,6 | 122436769,5 | 105963983,9 | 141426411,5 | 0,393 |
| PC(16:0/16:0)_M+H | 7466094275,0 | 6211311897,3 | 8491895060,5 | 7093930019,5 | 5592275742,8 | 7589910140,3 | 0,684 |
| PC(16:0/16:0)_M+Na | 235792471,8 | 170932228,4 | 251887984,4 | 201649710,1 | 160908896,7 | 227875662,4 | 0,218 |
| PC(16:0_18:1)_M+H | 16537341030,0 | 14494479296,8 | 18042454453,3 | 16286819655,0 | 14583413172,5 | 17840632798,5 | 0,912 |
| PC(16:0_18:1)_M+Na | 488518663,2 | 428431098,6 | 537991778,5 | 483662141,8 | 440274082,2 | 500641282,5 | 0,579 |
| PC(16:0_20:4)_M+H | 3659254373,5 | 3280335203,3 | 3788104202,0 | 3338531867,0 | 2740242045,8 | 3642435239,3 | 0,143 |
| PC(16:0_20:4)_M+Na | 77689001,4 | 65821565,2 | 90724172,8 | 67306517,8 | 53781414,8 | 73898644,1 | 0,123 |
| PC(16:0_22:4)_M+H | 259196835,5 | 236270881,4 | 289191330,4 | 248334088,6 | 217732595,4 | 276153815,7 | 0,579 |
| PC(16:0_22:6)_M+H | 5279773796,5 | 4772689811,5 | 5615651768,5 | 4850125916,5 | 4576801406,0 | 5258391783,3 | 0,315 |
| PC(16:0_22:6)_M+Na | 63776815,2 | 52744006,3 | 69471652,5 | 54427538,0 | 45470158,5 | 56456024,6 | 0,105 |
| PC(16:1/22:6)_M+H | 37322966,3 | 36705272,1 | 41545513,5 | 34217557,1 | 33260750,1 | 37795942,1 | 0,023 |
| PC(17:0/18:1)_M+Na | 28519935,2 | 22987447,5 | 31321838,3 | 25983029,3 | 21164600,5 | 32043526,7 | 0,529 |
| PC(18:0/22:4)_M+H | 136404445,2 | 124377736,5 | 156111589,8 | 135609314,2 | 116865535,4 | 151566378,1 | 0,853 |
| PC(18:0/22:5)_M+H | 40718574,3 | 33349595,8 | 41588644,9 | 38636002,9 | 34955862,4 | 43987277,2 | 0,796 |
| PC(18:0_18:1)_M+H | 3382869321,0 | 2999447663,0 | 3671610942,8 | 3492092432,5 | 3114448748,5 | 3562481520,8 | 1,000 |
| PC(18:0_18:1)_M+Na | 58058607,3 | 48799947,5 | 70075571,1 | 59443114,5 | 49105542,0 | 65172727,0 | 0,796 |
| PC(18:0_20:3)_M+H | 49439744,2 | 43371233,0 | 53723461,0 | 47105766,0 | 42475934,8 | 53632850,1 | 0,684 |
| PC(18:0_20:4)_M+H | 3122497707,0 | 2765483233,5 | 3426226411,8 | 3095317796,5 | 2503411085,8 | 3463541211,5 | 0,684 |
| PC(18:0_20:4)_M+Na | 86003434,1 | 70555246,3 | 97032168,3 | 86627834,9 | 69027703,6 | 91485718,6 | 0,739 |
| PC(18:0_22:6)_M+H | 1511276421,5 | 1329370800,3 | 1669062477,8 | 1437966875,5 | 1331629314,5 | 1465900502,5 | 0,218 |
| PC(18:0_22:6)_M+Na | 41064432,2 | 31836221,1 | 45461795,7 | 36782089,5 | 31857178,1 | 38965491,0 | 0,143 |
| PC(18:1/16:1)_M+Na | 13859238,6 | 12844170,3 | 15393790,8 | 15442892,5 | 13630513,2 | 16250589,4 | 0,190 |
| PC(18:1/16:1)_M+H | 436651520,7 | 390052137,9 | 468939774,5 | 456212883,2 | 407376573,9 | 515955679,6 | 0,481 |
| PC(18:1/18:1)_M+H | 1311783605,5 | 1146512936,8 | 1444784290,0 | 1329033931,0 | 1143345882,5 | 1427174071,0 | 0,971 |
| PC(18:1/20:4)_M+H | 1437192544,5 | 1335520694,5 | 1468635748,5 | 1299841628,0 | 1126281244,8 | 1454415149,8 | 0,165 |
| PC(18:1/20:4)_M+Na | 45466553,7 | 43218072,8 | 46539356,7 | 39929611,6 | 37282858,5 | 44294328,3 | 0,035 |
| PC(18:2/20:4)_M+H | 35530485,6 | 32935368,1 | 38082509,3 | 34066589,8 | 29946245,2 | 38515124,2 | 0,631 |
| PC(20:1/20:4)_M+H | 73103750,7 | 66637569,2 | 75518775,0 | 68159332,5 | 58565341,8 | 76114290,2 | 0,579 |
| PC(20:1_22:6)_M+H | 38780509,8 | 35118712,3 | 41472889,1 | 36109044,6 | 30806022,5 | 39801408,6 | 0,247 |
| PC(20:4/20:4)_M+H | 53913558,5 | 49895496,4 | 59149635,3 | 47817599,6 | 44216416,5 | 53792853,1 | 0,075 |
| PC(20:4_22:6)_M+H | 115491829,5 | 106945700,6 | 124657538,6 | 103637354,8 | 98537503,2 | 107945757,8 | 0,009 |
| PC(22:6/22:6)_M+H | 113274049,2 | 109448404,0 | 125258595,9 | 103736449,6 | 98000818,7 | 117049916,4 | 0,247 |
| PC(31:0)_M+H | 29212845,7 | 24538482,6 | 31784891,2 | 26723557,4 | 25009343,6 | 29936047,5 | 0,739 |
| PC(33:2)_M+H | 153668717,5 | 139137259,8 | 169080136,3 | 157291169,9 | 143531386,9 | 171999920,9 | 0,739 |
| PC(33:3)_M+H | 6024728,1 | 5215855,0 | 6685264,3 | 6702522,0 | 5639122,8 | 7344595,5 | 0,247 |
| PC(35:4)_M+Na | 248674451,2 | 219045737,0 | 259033398,5 | 229161614,4 | 218504429,1 | 257551115,5 | 0,481 |
| PC(36:4)_M+H | 7242770,7 | 6362892,9 | 8011243,3 | 7616866,4 | 6001145,6 | 8607527,1 | 0,631 |
| PC(36:5)_M+H | 33571936,7 | 31136284,4 | 35636050,3 | 30420845,4 | 26635360,7 | 33225045,2 | 0,089 |
| PC(38:5)_M+H | 28308699,1 | 26938851,3 | 31795085,7 | 29556915,9 | 27792168,4 | 31778582,0 | 0,529 |
| PC(40:7)_M+H | 706084439,2 | 668956436,7 | 741904707,1 | 630612568,2 | 608307513,8 | 664031433,9 | 2,000 |
| PC(40:7)_M+Na | 9640193,6 | 8778516,7 | 10484556,6 | 8308083,2 | 7528524,9 | 8692927,5 | 0,007 |
| Total PC | 47407589388,0 | 42132627680,8 | 51337324757,8 | 45849175049,5 | 40408749804,3 | 49487538217,8 | 0,631 |
| PCo(16:0/16:0)_M+H | 45431974,5 | 36810758,0 | 51797116,1 | 42642009,0 | 34781822,2 | 48552387,2 | 0,684 |
| PCo(18:1/16:0)\|PCp(16:0_18:0)_M+H | 156993293,5 | 135678825,6 | 173247117,6 | 150496767,2 | 133277480,3 | 168632327,8 | 0,796 |
| PCo(35:5)\|PCp(35:4)_M+Na | 365347847,6 | 341869433,8 | 408634994,5 | 359986603,3 | 324822811,3 | 371483172,5 | 0,579 |
| PCo(36:7)\|PCp(36:6)_M+H | 50504187,0 | 38023505,2 | 57961320,0 | 43379890,0 | 31303210,1 | 53307285,8 | 0,481 |
| PCo(37:6)\|PCp(37:5)_M+Na | 91117894,0 | 85637763,2 | 94404168,4 | 92486980,8 | 84342807,8 | 98453567,1 | 0,796 |
| PCo(37:6)\|PCp(37:5)_M+H | 22561632,9 | 20402397,8 | 24740060,0 | 23147274,1 | 21037821,2 | 25771007,4 | 0,579 |
| PCo(38:5)\|PCp(38:4)_M+H | 16292205,8 | 14013997,6 | 17034930,9 | 14673432,9 | 13388820,1 | 15935865,8 | 0,218 |
| PCo(38:7)\|PCp(38:6)_M+H | 23511296,2 | 19644824,6 | 24676622,8 | 18344418,1 | 16283997,0 | 20654328,4 | 0,029 |
| PCp(16:0/16:0)\|PCo(16:0_16:1)_M+H | 27578467,2 | 22250098,2 | 32288412,4 | 24103485,7 | 18394610,7 | 31815153,4 | 0,436 |
| PCp(34:6)_M+H | 31204293,1 | 23085703,9 | 40540061,4 | 28137528,9 | 20893169,0 | 32666863,4 | 0,579 |
| PCp(35:5)_M+Na | 9313820,4 | 8202317,2 | 9601175,9 | 9072387,5 | 7842715,1 | 9394926,2 | 0,436 |
| PCp(35:5)\|PEo(38:6)_M+Na | 56579719,4 | 41017273,8 | 60849775,9 | 48070240,2 | 40217876,9 | 54403777,3 | 0,165 |
| PCp(35:5)\|PCo(33:3)_M+H | 339164557,3 | 304223079,9 | 366348364,0 | 336363524,6 | 309302894,1 | 349585619,1 | 0,853 |
| PCp(37:6)_M+Na | 95264685,4 | 83101380,4 | 108603310,7 | 92047645,7 | 84125491,4 | 97351085,0 | 0,393 |
| PCp(38:5)\|\|PCo(38:6)_M+H | 15987897,8 | 15731345,8 | 17311471,1 | 14617640,9 | 13997265,4 | 16079414,0 | 0,075 |
| PCp(39:6)_M+Na | 6798190,1 | 5338353,4 | 8077950,5 | 6915529,7 | 5329081,8 | 7698678,6 | 0,796 |
| Total PCo | 1369549163,5 | 1210596334,5 | 1468850004,8 | 1304555442,5 | 1206329494,8 | 1392113972,0 | 0,393 |
| PE(18:0_22:4)_M+H | 174521336,3 | 154825453,3 | 205528945,0 | 184399186,2 | 159458853,1 | 204272375,8 | 0,739 |
| PE(22:6/16:0)_M+H | 720985324,8 | 648108138,0 | 770357147,4 | 681047031,2 | 615771387,4 | 791902215,6 | 0,579 |
| PE(22:6/16:0)_M+Na | 18576954,7 | 16890913,6 | 19813368,4 | 18289222,0 | 16704227,8 | 18868582,4 | 0,579 |
| PE(22:6/18:0)_M+Na | 47819118,7 | 45220532,2 | 50070391,8 | 49570126,1 | 46688262,5 | 50903047,9 | 0,393 |
| PE(22:6/22:6)_M+H | 42967067,8 | 39288765,8 | 46376927,5 | 41015418,7 | 37412863,8 | 52740809,2 | 0,912 |
| PE(34:1)_M+H | 120562884,6 | 109548270,2 | 136037346,4 | 126154783,8 | 115122329,5 | 137330794,7 | 0,684 |
| PE(36:1)_M+H | 197718290,0 | 185885871,9 | 219863417,4 | 212153851,2 | 200401104,5 | 217007181,9 | 0,353 |
| PE(36:4)_M+H | 147361155,1 | 130443479,8 | 159535452,4 | 143122242,1 | 128290125,7 | 158463960,2 | 0,853 |
| PE(37:4)_M+H | 5149304,3 | 4635134,0 | 5706357,8 | 4984505,4 | 4451529,4 | 5645126,9 | 0,796 |
| PE(38:3)_M+H | 11809545,1 | 10736065,0 | 12666999,5 | 11962045,0 | 10857937,4 | 13748606,8 | 0,631 |
| PE(38:4)_M+H | 55707937,1 | 47994313,3 | 63184394,3 | 56890971,1 | 47211809,0 | 63536859,5 | 0,912 |
| PE(20:4/18:0)_M+H | 1092685827,5 | 938134743,6 | 1212346317,8 | 1101237429,5 | 928003745,2 | 1250005054,3 | 1,000 |
| PE(38:4)_M+Na | 35481444,6 | 33095789,8 | 39127347,3 | 35036182,3 | 33518321,6 | 36836684,9 | 0,631 |
| PE(39:6)_M+H | 9899041,7 | 8903860,2 | 11017530,6 | 9899840,2 | 9167588,6 | 10414125,3 | 1,000 |
| PE(40:5)_M+H | 66783091,2 | 58914292,9 | 71738117,3 | 69767774,2 | 60329977,2 | 77523820,6 | 0,481 |
| PE(40:6)_M+Na | 49186102,3 | 46373712,1 | 51026529,8 | 50794158,4 | 47881379,1 | 51758603,9 | 0,315 |
| PE(40:6)_M+H | 2640403877,5 | 2300143162,5 | 2931500500,0 | 2673817616,0 | 2407969175,8 | 2973791750,8 | 1,000 |
| PE(42:10)_M+H | 21424156,1 | 19760090,9 | 23470010,9 | 20435811,8 | 18951470,9 | 21767487,4 | 0,190 |
| PE(44:10)_M+H | 49051875,4 | 46596183,3 | 53186769,8 | 49823880,2 | 45600914,2 | 56473300,4 | 0,853 |
| Total PE | 5462491588,0 | 4930955591,3 | 6070844425,3 | 5607962167,5 | 4999803287,8 | 6114154677,5 | 0,971 |
| PEo(16:1_20:4)\|PEp(36:4)_M+H | 149724563,9 | 118787646,5 | 176192247,6 | 135324111,2 | 108686620,2 | 149368423,8 | 0,315 |
| PEo(16:1_22:4)PEp(38:4)_M+H | 134558725,3 | 112140966,3 | 155711492,8 | 129185460,8 | 111101442,7 | 150808691,2 | 0,684 |
| PEo(16:1_22:6)\|PEp(16:0/22:6)_M+Na | 12777973,3 | 11788379,1 | 13822679,1 | 13476869,0 | 11933923,5 | 14421205,1 | 0,631 |
| PEo(16:1_22:6)\|PEp(16:0/22:6)_M+H | 598597908,2 | 545292930,8 | 666486497,2 | 527698071,9 | 452971589,1 | 607905242,2 | 0,143 |
| PEo(18:1_18:2)\|PEp(18:1/18:1)_M+H | 302430596,9 | 279918189,0 | 333012218,9 | 313385462,7 | 285358578,9 | 337542796,6 | 0,631 |
| PEo(18:1_20:4)\|PEp(18:0/20:4)_M+H | 344957876,9 | 302238427,9 | 373607604,0 | 328759113,4 | 288705637,9 | 364379049,5 | 0,684 |
| PEo(18:1_20:4)_M+Na | 13887694,8 | 12766632,1 | 15190811,4 | 13520428,9 | 12414365,3 | 14245568,2 | 0,436 |
| PEo(18:1_22:6)_M+H | 946459351,3 | 808031650,2 | 1032184476,3 | 946426515,1 | 850205245,1 | 976322158,1 | 0,684 |
| PEo(18:1_22:6)_M+Na | 33637812,7 | 29517624,2 | 37318351,6 | 33289356,4 | 29466263,7 | 34899100,8 | 0,684 |
| PEo(18:2_22:4)\|PEp(18:1/22:4)_M+H | 421730728,2 | 368891748,2 | 461368863,1 | 440412596,5 | 392051423,6 | 463288011,1 | 0,579 |
| PEo(18:2_22:6)_M+Na | 7948595,7 | 7063829,7 | 8532402,4 | 7797283,2 | 6211144,8 | 8526920,4 | 0,436 |
| PEo(34:2)\|PEp(34:1)_M+H | 180064347,2 | 172419807,4 | 194812474,5 | 187589963,7 | 177843333,6 | 193377146,3 | 0,353 |
| PEo(36:4)\|PEp(36:3)_M+H | 16161186,2 | 12420625,8 | 17610309,9 | 15486814,6 | 13185088,7 | 16781033,9 | 0,796 |
| PEo(36:5)\|PEp(36:4)_M+Na | 5145919,1 | 4610311,2 | 5831811,4 | 4417784,1 | 4251665,9 | 5205707,9 | 0,190 |
| PEo(37:5)\|PEp(37:4)_M+H | 6196341,0 | 5394564,3 | 6797306,7 | 6168085,8 | 5372292,6 | 6553706,7 | 0,912 |
| PEo(38:4)\|PEp(38:3)_M+H | 17091750,1 | 13650491,3 | 19452425,5 | 16978194,5 | 14911886,9 | 18825986,2 | 0,971 |
| PEo(38:6)\|PEp(38:5)_M+H | 57374649,2 | 49691131,3 | 61863009,5 | 54689325,4 | 50739740,4 | 64010875,5 | 0,853 |
| PEo(40:6)\|PEp(40:5)_M+H | 34033074,3 | 30559294,1 | 38693013,5 | 35872428,0 | 31012881,0 | 40523034,8 | 0,684 |
| PEo(40:7)\|PEp(40:6)_M+H | 13512636,6 | 10008087,3 | 15206701,5 | 13034986,5 | 10552166,9 | 13951441,1 | 0,631 |
| Total PEo | 3300473471,0 | 2960397796,3 | 3592654384,8 | 3205934286,5 | 2966031345,0 | 3434442215,8 | 0,739 |
| PG(34:1)_M+NH4 | 68625868,6 | 65632326,4 | 75276178,6 | 66759746,8 | 64236152,5 | 70809290,5 | 0,529 |
| PG(34:1)_M+Na | 8596272,8 | 6665849,6 | 9832190,4 | 7893820,2 | 6764933,0 | 8591422,7 | 0,393 |
| T.Pg | 1701122997,5 | 1628446534,8 | 1847042737,5 | 1897460697,0 | 1851429264,3 | 1963724025,5 | 0,029 |
| SM(d18:1/17:0)_M+Na | 286156423,4 | 273057748,7 | 304890749,7 | 298227764,8 | 284920884,3 | 333083884,4 | 0,280 |
| SM(d18:1/18:0)_M+H | 446248035,7 | 379369043,8 | 497906914,1 | 454308114,8 | 396814810,4 | 535978769,2 | 0,796 |
| SM(d18:1/18:0)_M+Na | 9955535,4 | 8172542,3 | 10854406,3 | 9522697,7 | 8828651,6 | 11221326,0 | 0,971 |
| SM(d38:1)_M+H | 39041347,4 | 35304484,1 | 43060988,0 | 43694730,3 | 40741594,7 | 45850583,6 | 0,105 |
| SM(d42:2)_M+H | 20352593,8 | 16769932,7 | 22622059,5 | 19715190,6 | 17927392,9 | 21626708,0 | 0,853 |
| Total SM | 797123664,1 | 750400827,4 | 847391485,7 | 823001015,7 | 773995354,9 | 903328535,1 | 0,280 |
| TAG(10:0/14:1/18:0)_M+NH4 | 14608579,6 | 13994514,9 | 15528500,4 | 15391426,4 | 14438907,1 | 16503875,8 | 0,280 |
| TAG(10:0/15:0/18:0)_M+NH4 | 11650814,0 | 11278141,2 | 12503090,6 | 12697053,9 | 11706398,2 | 13645723,9 | 0,143 |
| TAG(10:0/16:1/18:0)_M+Na | 12905724,7 | 12596181,1 | 13527683,7 | 13997188,2 | 13006838,1 | 14720833,0 | 0,143 |
| TAG(10:0/16:1/18:0)_M+NH4 | 57468733,8 | 54589250,0 | 61977120,5 | 60915543,1 | 57342183,2 | 66858212,6 | 0,218 |
| TAG(10:0/18:2/18:0)_M+Na | 24943987,4 | 23747123,2 | 26331300,4 | 26325684,6 | 24691809,7 | 28072059,9 | 0,280 |
| TAG(12:0/14:0/18:0)_M+NH4 | 51574086,0 | 50355729,5 | 55211672,1 | 54365859,0 | 52033282,1 | 59467597,4 | 0,143 |
| TAG(12:0/14:0/18:0)_M+Na | 9706667,7 | 9569269,6 | 10516695,1 | 10390352,5 | 9521964,6 | 10742260,3 | 0,393 |
| TAG(12:0/14:0/18:2)_M+NH4 | 19331335,9 | 18494133,9 | 20589339,5 | 20639073,6 | 19305196,0 | 22181746,3 | 0,190 |
| TAG(12:0/15:0/18:0)_M+NH4 | 41685767,0 | 40192770,0 | 45200605,8 | 43715582,7 | 41070008,2 | 47313945,9 | 0,393 |
| TAG(12:0/15:0/18:0)_M+Na | 11484391,3 | 11412954,3 | 12215996,9 | 12022583,1 | 11249631,4 | 13152822,8 | 0,579 |
| TAG(12:0/16:0/20:1)_M+NH4 | 200224564,4 | 193780248,7 | 210211635,9 | 209054789,4 | 196400876,2 | 222125999,0 | 0,353 |
| TAG(12:0/16:0/20:1)_M+Na | 41112050,5 | 40117851,6 | 42525662,3 | 43492539,2 | 40612905,9 | 46280504,2 | 0,218 |
| TAG(12:0/16:1/17:0)_M+Na | 13110753,8 | 12449755,0 | 13280132,8 | 13535496,0 | 12496527,2 | 14079533,7 | 0,315 |
| TAG(14:0/16:1/15:0)_M+Na | 10023589,7 | 9574109,4 | 10675905,2 | 10719417,7 | 9794388,7 | 11540205,7 | 0,315 |
| TAG(12:0/16:1/17:0)_M+NH4 | 60021930,0 | 57707322,2 | 63616028,6 | 62718726,7 | 59375735,9 | 68555305,0 | 0,247 |
| TAG(12:0/16:1/18:0)_M+Na | 48399062,1 | 46113385,1 | 50658660,1 | 50893974,4 | 47459374,7 | 54884597,0 | 0,280 |
| TAG(12:0/18:2/12:0)_M+NH4 | 2317175,7 | 2201865,1 | 2567148,0 | 2525466,5 | 2260781,2 | 2606496,2 | 0,436 |
| TAG(12:0/20:0/22:1)_M+NH4 | 27421929,6 | 25391357,2 | 32361424,5 | 27823004,4 | 24508866,4 | 32402012,1 | 0,971 |
| TAG(12:0/20:0/22:1)_M+Na | 6753867,4 | 6235688,3 | 8367707,4 | 6717425,0 | 6006806,3 | 8083948,2 | 0,796 |
| TAG(14:0/14:0/14:0)_M+Na | 4960474,7 | 4763757,9 | 5117735,9 | 5202187,4 | 4647580,3 | 5638221,8 | 0,529 |
| TAG(14:0/14:0/14:0)_M+NH4 | 16176900,6 | 15628429,0 | 16824227,6 | 17077008,5 | 16026745,1 | 18866883,9 | 0,143 |
| TAG(14:0/14:0/18:0)_M+NH4 | 102212296,7 | 100043218,0 | 107660503,3 | 106957145,7 | 101361524,1 | 114690763,2 | 0,218 |
| TAG(14:0/14:0/18:2)_M+NH4 | 109725221,0 | 104171718,1 | 117545873,4 | 116319730,4 | 109302604,4 | 125162320,7 | 0,247 |
| TAG(14:0/14:0/24:0)_M+NH4 | 19383696,3 | 16490598,5 | 22766759,0 | 20826546,4 | 17754718,9 | 24525971,7 | 0,393 |
| TAG(14:0/15:0/18:1)_M+Na | 35723499,3 | 34813218,2 | 37759045,4 | 38190299,5 | 35404897,5 | 41053761,4 | 0,280 |
| TAG(14:0/15:0/18:1)_M+NH4 | 139613697,0 | 134552763,6 | 147638951,1 | 147980839,5 | 139385793,5 | 162009900,9 | 0,218 |
| TAG(14:0/15:0/18:2)_M+NH4 | 104319986,1 | 100026152,5 | 112098393,9 | 111262662,5 | 104471874,5 | 119573649,8 | 0,165 |
| TAG(14:0/15:0/20:0)_M+NH4 | 48408469,5 | 44929759,0 | 53978444,8 | 52749507,2 | 48270510,1 | 54469089,9 | 0,315 |
| TAG(14:0/15:0/20:0)_ | 12002518,3 | 11021677,3 | 13247671,5 | 12915109,8 | 11798614,2 | 13666940,6 | 0,315 |
| TAG(14:0/15:0/20:1)_M+Na | 27930312,9 | 27287030,9 | 29852676,5 | 28817256,3 | 27582075,4 | 30780750,2 | 0,481 |
| TAG(14:0/15:0/20:1)_M+NH4 | 103231660,5 | 101345886,6 | 112471803,6 | 108212997,4 | 102617413,6 | 114883087,3 | 0,436 |
| TAG(14:0/15:0/20:2)_M+Na | 33801597,4 | 32751462,1 | 35273570,1 | 35779485,8 | 33213185,4 | 38155672,1 | 0,393 |
| TAG(14:0/15:0/20:2)_M+NH4 | 119546230,3 | 113550235,0 | 125942943,3 | 126777556,4 | 118508417,1 | 136920568,7 | 0,165 |
| TAG(14:0/15:0/20:3)_M+NH4 | 47849071,0 | 45485567,1 | 50516344,8 | 50826163,4 | 47839342,4 | 55110248,1 | 0,165 |
| TAG(14:0/16:0/20:0)_M+NH4 | 48938394,5 | 42809905,5 | 52215277,9 | 52111492,5 | 45858406,9 | 54085892,1 | 0,218 |
| TAG(14:0/16:0/20:1)_M+Na | 64808977,0 | 63592755,3 | 66062348,9 | 64407129,9 | 59071748,8 | 69831412,2 | 0,796 |
| TAG(14:0/18:1/18:3)_M+NH4 | 9918898,9 | 9416283,1 | 10155708,2 | 10221596,4 | 9605226,7 | 10635985,4 | 0,353 |
| TAG(14:0/24:0/18:0)_M+NH4 | 6251408,4 | 6112237,7 | 6874031,9 | 6882173,7 | 6475625,5 | 7375297,1 | 0,089 |
| TAG(14:0/24:0/20:1)_M+NH4 | 7471518,3 | 7092161,7 | 8078454,6 | 7897198,7 | 7562603,5 | 8794948,8 | 0,218 |
| TAG(14:1/15:0/22:0)_M+NH4 | 37650111,7 | 35960638,6 | 39850763,2 | 37869889,1 | 35785713,1 | 40829676,5 | 0,912 |
| TAG(14:1/16:1/15:0)_M+NH4 | 19035049,6 | 18281045,0 | 20094137,1 | 20233267,4 | 18990864,9 | 21863451,0 | 0,143 |
| TAG(14:1/18:2/15:0)_M+NH4 | 12550399,8 | 12050797,7 | 13507146,8 | 13410402,7 | 12634397,9 | 14606655,8 | 0,089 |
| TAG(15:0/16:0/22:3)_M+NH4 | 24523837,2 | 23836834,1 | 25175872,0 | 24991752,4 | 23285621,9 | 27463491,7 | 0,481 |
| TAG(15:0/16:0/24:0)_M+NH4 | 7455469,1 | 7234138,2 | 7987928,4 | 7973785,0 | 7593700,4 | 8445848,8 | 0,123 |
| TAG(15:0/16:0/26:0)_M+NH4 | 4012537,8 | 3864700,9 | 4470519,2 | 4423753,3 | 4076994,5 | 4875681,7 | 0,105 |
| TAG(15:0/16:1/26:0)_M+NH4 | 8755265,3 | 8238088,1 | 9465463,7 | 9091309,2 | 8790413,4 | 10259817,3 | 0,218 |
| TAG(15:0/18:0/20:2)_M+NH4 | 25951310,7 | 25250669,9 | 27409652,9 | 26022950,8 | 24233802,3 | 28058207,8 | 0,912 |
| TAG(15:0/18:2/20:1)_M+NH4 | 23882173,2 | 23374610,3 | 24666010,3 | 24511084,6 | 22868412,7 | 26910842,7 | 0,436 |
| TAG(16:0/16:0/16:0)_M+NH4 | 101960169,5 | 100277055,2 | 108658380,3 | 104914281,4 | 98951817,7 | 111872542,9 | 0,796 |
| TAG(16:0/16:0/18:1)_M+Na | 64692283,0 | 63530468,9 | 65909728,7 | 64360806,3 | 59121974,1 | 69796998,3 | 0,796 |
| TAG(16:0/16:0/18:1)_M+NH4 | 302843826,4 | 286868745,1 | 313850394,4 | 302710437,2 | 251646143,2 | 325410859,1 | 0,912 |
| TAG(16:0/16:0/18:2)_M+NH4 | 274373999,6 | 261694043,9 | 282777452,0 | 271678143,7 | 259457247,4 | 289844693,1 | 1,000 |
| TAG(16:0/16:0/18:2)_M+Na | 73008676,3 | 69796373,1 | 74784043,6 | 71543228,2 | 68708490,0 | 77163249,2 | 0,912 |
| TAG(16:0/16:0/20:1)_M+NH4 | 123025025,3 | 110831708,4 | 136777585,4 | 127090776,2 | 95690985,2 | 135520583,3 | 0,971 |
| TAG(16:0/16:0/20:1)_M+Na | 9181699,7 | 8135066,2 | 11088221,5 | 9226350,8 | 6812164,1 | 10542228,8 | 0,853 |
| TAG(16:0/16:0/20:2)_M+NH4 | 1124507662,5 | 1039365456,4 | 1193931195,3 | 1129331311,5 | 866127148,9 | 1210797714,0 | 0,796 |
| TAG(16:0/16:0/20:2)_M+Na | 231288987,1 | 221936622,8 | 240531854,4 | 229267341,7 | 197676864,8 | 248598446,2 | 0,853 |
| TAG(16:0/16:0/20:3)_M+NH4 | 463045212,2 | 448912827,7 | 490328887,1 | 462938317,1 | 422317494,4 | 504901468,4 | 0,796 |
| TAG(16:0/16:0/20:3)_M+Na | 118988153,6 | 114998997,4 | 124386277,5 | 118571905,4 | 109574468,8 | 129859078,3 | 0,853 |
| TAG(16:0/16:1/18:2)_M+NH4 | 128833644,4 | 126089238,6 | 132658773,0 | 134360947,1 | 125219397,9 | 144295126,2 | 0,353 |
| TAG(16:0/16:1/18:2)_M+Na | 25858165,2 | 24902470,5 | 26757224,2 | 26875634,9 | 24973755,7 | 27775875,4 | 0,393 |
| TAG(16:0/18:1/22:1)_M+Na | 5948410,7 | 5351777,8 | 7705863,8 | 5956501,6 | 4646116,5 | 6959418,0 | 0,739 |
| TAG(16:0/18:1/22:1)_M+NH4 | 26458113,7 | 23533310,8 | 32716544,4 | 26696687,9 | 21011415,9 | 30594211,7 | 0,853 |
| TAG(16:0/18:1/24:1)_M+NH4 | 11563233,2 | 10814069,4 | 12542356,3 | 11386273,7 | 11060143,9 | 13229173,0 | 1,000 |
| TAG(16:1/15:0/20:1)_M+NH4 | 70196117,1 | 67680293,4 | 75238125,7 | 72664944,6 | 69260746,5 | 77801444,8 | 0,353 |
| TAG(16:1/15:0/20:1)_M+Na | 18090009,1 | 17524546,2 | 19068297,6 | 18677499,4 | 17927802,1 | 20135813,4 | 0,353 |
| TAG(16:1/16:0/16:1)_M+NH4 | 200264185,8 | 193565639,5 | 208007277,6 | 211802816,2 | 197186816,6 | 228885800,5 | 0,165 |
| TAG(16:1/16:1/16:1)_M+Na | 13799369,9 | 13156836,3 | 14676212,9 | 14490558,2 | 13697690,7 | 15462350,6 | 0,247 |
| TAG(16:1/16:1/20:2)_M+NH4 | 149376645,6 | 141467749,8 | 151207777,1 | 149131389,3 | 139031741,9 | 155335163,8 | 0,631 |
| TAG(16:1/16:1/20:2)_M+Na | 39044483,1 | 36550225,9 | 39592067,1 | 38485825,0 | 35692971,1 | 40577409,0 | 1,000 |
| TAG(17:0/17:0/17:0)_M+Na | 516959273,5 | 302467729,5 | 661506273,1 | 615764860,7 | 409150191,8 | 764978030,6 | 0,280 |
| TAG(17:0/18:1/18:3)_M+NH4 | 5316846,0 | 4970956,0 | 5543652,3 | 5571989,3 | 5246245,1 | 6013639,6 | 0,165 |
| TAG(18:1/18:0/18:1)_M+NH4 | 281796403,3 | 241110794,4 | 322102641,2 | 287768343,0 | 193996934,6 | 311897524,0 | 0,912 |
| TAG(18:1/18:0/18:1)_M+Na | 27801676,6 | 24557067,4 | 32876431,7 | 27199371,7 | 18665099,3 | 31116776,5 | 0,739 |
| TAG(18:1/18:1/18:1)_M+NH4 | 1837381777,5 | 1705214883,0 | 1943324347,5 | 1849891634,5 | 1432104010,5 | 1994576101,8 | 0,912 |
| TAG(18:1/18:1/18:1)_M+Na | 369564409,8 | 352354573,9 | 380835837,8 | 364990365,1 | 317362321,4 | 400672368,9 | 0,912 |
| TAG(18:1/18:1/18:2)_M+NH4 | 582618364,2 | 565975429,5 | 624910769,7 | 584649208,0 | 518345164,2 | 643701383,8 | 1,000 |
| TAG(18:1/18:1/18:2)_M+Na | 142902025,0 | 138667106,0 | 152120721,6 | 143832287,3 | 129557648,6 | 158476502,2 | 1,000 |
| TAG(18:1/18:1/18:3)_M+Na | 59027758,8 | 57279659,3 | 60161475,9 | 59287815,9 | 55134331,9 | 63763642,7 | 0,739 |
| TAG(18:1/18:1/18:3)_M+NH4 | 219519957,6 | 213347993,3 | 227054970,2 | 223570632,5 | 209497406,8 | 234249746,3 | 0,529 |
| TAG(18:1/20:1/18:1)_M+NH4 | 31829446,4 | 29197173,7 | 35741591,2 | 33008319,3 | 23681054,3 | 35088297,6 | 0,853 |
| TAG(18:1/20:1/18:1)_M+Na | 1813444,2 | 1484506,0 | 2282178,9 | 1729970,5 | 1115789,5 | 2078927,2 | 0,631 |
| TAG(18:1/20:1/18:1)\|TAG(18:2/20:1/18:0)_M+NH4 | 33274708,3 | 30687181,8 | 37611235,4 | 34461761,3 | 24956320,0 | 36671891,8 | 0,853 |
| TAG(18:1/20:1/18:2)_M+NH4 | 14506656,3 | 13712783,3 | 15823676,2 | 14465214,4 | 12004335,1 | 15853262,5 | 0,971 |
| TAG(18:2/16:1/18:2)_M+Na | 2726635,8 | 2544914,4 | 2903923,0 | 2781451,2 | 2534437,6 | 2882982,5 | 0,971 |
| TAG(18:2/18:1/15:0)_M+NH4 | 20812575,4 | 20257294,4 | 21672515,8 | 22170238,2 | 20697355,2 | 23702006,7 | 0,247 |
| TAG(18:2/18:1/15:0)_M+Na | 6804436,2 | 6538938,2 | 7172429,5 | 7350726,4 | 6516800,3 | 7733027,0 | 0,280 |
| TAG(24:0/18:2/17:0)_M+NH4 | 3787712,6 | 3643045,2 | 4128040,9 | 3894050,3 | 3661836,6 | 4304053,8 | 0,579 |
| Total TAG | 9549015300,5 | 9266307424,3 | 9921752887,5 | 9766337381,5 | 8667369589,5 | 10557754131,3 | 0,684 |

**Table S8.** Lipid variables by mouse genotype with median, interquartile and p-value in negative ionization mode in the cortex.

| **Lipid variables** | **WT** | | | **TG** | | | **p value (Mann-Whitney)** |
| --- | --- | --- | --- | --- | --- | --- | --- |
|  | **Median** | **IQ1** | **IQ3** | **Median** | **IQ1** | **IQ3** |  |
| Cer(d18:1/16:0)_M-H | 47598999,4 | 45778470,0 | 52526208,7 | 48247021,8 | 41476617,6 | 54302057,7 | 0,853 |
| Cer(d18:1/16:0)_M+CH3COO | 1172668557,0 | 1007807727,8 | 1459241629,3 | 1249743495,0 | 1118495907,8 | 1370809297,3 | 0,631 |
| Cer(d18:0_18:1)_M-H | 444431090,1 | 362691259,3 | 507158635,2 | 412548358,3 | 343246238,0 | 537311732,5 | 0,853 |
| Cer(d18:0_18:1)_M+CH3COO | 672693637,1 | 541239146,9 | 767529373,6 | 617103052,4 | 517014341,1 | 810485658,8 | 0,853 |
| Cer(d18:1/20:0)_M-H | 51574914,8 | 45773217,7 | 61233580,1 | 52627320,8 | 45607044,1 | 56043736,0 | 1,000 |
| Cer(d18:1/20:0)_M+CH3COO | 90113182,0 | 80747789,3 | 106239973,8 | 91615647,3 | 80645846,6 | 98327928,8 | 0,971 |
| Cer(d18:1/24:0)_M-H | 9132426,3 | 7715676,3 | 9930268,2 | 8195796,2 | 7405770,0 | 9301141,4 | 0,353 |
| Cer(d18:1/24:0)_M+CH3COO | 21778468,2 | 17619284,9 | 24584973,6 | 19919105,5 | 17649903,8 | 21298851,0 | 0,393 |
| Cer(d18:1/24:1)_M-H | 93298654,7 | 89404173,0 | 99786044,4 | 88802105,5 | 78113933,4 | 99184302,4 | 0,481 |
| Cer(d18:1/24:1)_M+CH3COO | 209362125,0 | 198631638,9 | 221479745,3 | 196453098,7 | 174114050,2 | 222058418,7 | 0,481 |
| Total Cer | 2748679618,0 | 2662617884,5 | 3293937967,8 | 2844985223,0 | 2640665596,5 | 3129365505,3 | 0,912 |
| CL(16:1_18:1_20:4_20:4)_M-H | 14992196,8 | 13984357,1 | 15844685,4 | 13388397,1 | 12865696,6 | 15942517,8 | 0,393 |
| CL(16:1_18:1_20:4_22:6)_M-H | 10887308,1 | 10296964,8 | 11484368,4 | 10066792,6 | 9498084,4 | 11570774,3 | 0,436 |
| CL(18:1/18:1/18:1/18:1)_M-H | 13362793,5 | 12304721,1 | 14212007,1 | 12465681,6 | 11564914,8 | 13224280,3 | 0,280 |
| CL(18:1_18:1_18:1_18:2)_M-H | 10418878,0 | 9411821,6 | 11339203,0 | 10004532,1 | 9275149,7 | 11339222,3 | 0,971 |
| CL(18:1_18:1_18:1_20:4)_M-H | 20214438,4 | 17838264,6 | 20921461,3 | 18208380,9 | 16903049,8 | 21075998,8 | 0,529 |
| CL(18:1_18:1_18:1_22:6)_M-H | 16542913,3 | 14905875,1 | 17373546,7 | 15228265,8 | 14006076,1 | 16335184,9 | 0,393 |
| CL(18:1_18:1_20:4_20:4)_M-H | 25634433,7 | 23651193,2 | 26645983,1 | 23786340,3 | 21968672,9 | 27270946,3 | 0,529 |
| CL(18:1_18:1_20:4_22:6)_M-H | 23974318,7 | 20970579,7 | 24841042,4 | 21762311,5 | 20227111,1 | 24029102,3 | 0,247 |
| CL(18:1_18:1_22:6_22:6)_M-H | 13652033,9 | 12320117,4 | 14179565,8 | 12920462,2 | 11781598,2 | 13385244,1 | 0,218 |
| CL(18:1_18:2_22:6_22:6)_M-H | 12843936,4 | 11913285,6 | 13596682,9 | 12296138,6 | 11382929,2 | 12986655,0 | 0,218 |
| CL(18:1_20:4_22:6_22:6)_M-H | 8648415,0 | 7936957,3 | 9120673,1 | 7592177,2 | 7172444,1 | 8967470,1 | 0,190 |
| CL(18:2/18:2/18:2/18:2)_M-H | 6265895,2 | 5763275,9 | 6634757,4 | 6136039,3 | 5562520,9 | 6850551,6 | 0,971 |
| Total CL | 176597271,8 | 161222652,0 | 185448045,5 | 162249901,8 | 153473983,0 | 180226645,5 | 0,353 |
| FA(16:0)_M-H | 2692579623,0 | 2347463341,5 | 2973270398,5 | 2706994438,0 | 2553360149,0 | 3001991039,8 | 0,529 |
| FA(17:0)_M-H | 215823190,5 | 205046950,3 | 239348648,9 | 229990032,0 | 220925356,5 | 256802544,5 | 0,190 |
| FA(18:0)_M-H | 3800779336,0 | 3459569989,5 | 4640007916,8 | 4147860003,5 | 3970599394,0 | 4795774647,8 | 0,247 |
| FA(18:1)_M-H | 4821549566,0 | 4191121024,8 | 5830567065,8 | 4995969752,0 | 4399851131,5 | 5358680266,8 | 0,912 |
| FA(18:2)_M-H | 243624034,5 | 226752032,9 | 279553918,7 | 235878693,5 | 210460745,1 | 288417697,5 | 0,971 |
| FA(20:0)_M-H | 67220951,2 | 54557604,9 | 75272093,9 | 77655295,2 | 72257639,7 | 91581069,9 | 0,063 |
| FA(20:1)_M-H | 493153919,9 | 343309408,2 | 606822984,0 | 503578312,5 | 412740956,2 | 553216729,8 | 0,796 |
| FA(20:4)_M-H | 9872971806,0 | 9074069872,5 | 11323242400,5 | 9572245046,0 | 8820067861,8 | 10234818421,0 | 0,393 |
| FA(22:0)_M-H | 47257650,8 | 40762857,3 | 53453888,5 | 54683626,9 | 48575830,1 | 61838490,9 | 0,089 |
| FA(22:1)_M-H | 57329003,6 | 41117480,7 | 69717816,3 | 60778152,2 | 46528706,7 | 65384113,8 | 0,579 |
| FA(24:0)_M-H | 62604574,9 | 55694338,6 | 72846938,4 | 72237169,0 | 63051740,0 | 91386846,2 | 0,143 |
| FA(24:1)_M-H | 35248784,4 | 24974816,9 | 40264363,0 | 30155035,4 | 27032348,8 | 39243806,7 | 1,000 |
| FA(26:0)_M-H | 40038563,5 | 36782734,2 | 47977042,1 | 47966120,7 | 38793306,7 | 57193105,2 | 0,143 |
| Total FA | 22359235061,0 | 20640353678,5 | 27078670292,8 | 23231464180,0 | 20795719574,0 | 25228113833,5 | 1,000 |
| LPC(16:0)_M-CH3 | 57552711,8 | 51475459,0 | 62032414,8 | 52256705,2 | 49964380,1 | 56270201,6 | 0,218 |
| LPC(16:0)_M+CH3COO | 230624816,7 | 202574112,6 | 246013835,2 | 207413458,9 | 200443257,5 | 224692791,8 | 0,218 |
| LPC(16:1)_M-CH3 | 29718208,2 | 25604141,0 | 32331446,8 | 28031132,8 | 25983343,4 | 30651823,8 | 0,631 |
| LPC(18:0)_M-CH3 | 15660562,7 | 14213017,6 | 16771934,8 | 15211309,5 | 14188340,7 | 16845461,3 | 0,684 |
| LPC(18:0)_M+CH3COO | 73191321,1 | 66249029,3 | 76741120,6 | 70592924,5 | 66822396,4 | 76815990,9 | 0,739 |
| LPC(18:1)_M-CH3 | 15027169,5 | 13647564,3 | 16754832,5 | 14414688,1 | 13325013,4 | 15424245,0 | 0,353 |
| LPC(18:1)_M+CH3COO | 69989932,4 | 63116761,0 | 77028779,2 | 68153548,0 | 62996701,3 | 71738952,9 | 0,481 |
| LPC(20:1)_M+CH3COO | 3626116,7 | 3225012,0 | 3848877,5 | 3466349,5 | 3293514,9 | 3696032,3 | 0,529 |
| LPC(20:4)_M+CH3COO | 40708121,1 | 37345303,9 | 45852162,6 | 37421100,1 | 32929354,3 | 39448481,9 | 0,063 |
| LPC(22:6)_M+CH3COO | 32758954,5 | 29733889,0 | 37044975,2 | 28973078,4 | 27084603,5 | 34149293,6 | 0,190 |
| Total LPC | 579103728,7 | 503877366,4 | 606231119,7 | 523448211,7 | 506185750,1 | 561377134,5 | 0,280 |
| LPE(18:0)_M-H | 61215520,2 | 51059897,1 | 64588423,1 | 64409326,9 | 52506388,0 | 70511263,9 | 0,579 |
| LPE(20:4)_M-H | 66408641,5 | 56534522,3 | 69123719,8 | 55637644,8 | 51124983,4 | 60695914,3 | 0,063 |
| LPE(22:6)_M-H | 184096921,2 | 157447961,5 | 204209291,7 | 163356037,4 | 149004869,1 | 182051893,6 | 0,165 |
| Total LPE | 313224140,0 | 268588059,5 | 332032075,1 | 278367178,3 | 261569515,1 | 304384809,6 | 0,218 |
| LPI(18:0)_M-H | 106888480,2 | 85464332,9 | 115158151,1 | 88372509,6 | 75101021,2 | 99755203,9 | 0,075 |
| LPI(20:4)_M-H | 53940270,5 | 44638499,9 | 61414108,5 | 46650613,6 | 41636525,1 | 52476940,7 | 0,247 |
| Total LPI | 163037179,6 | 127206377,7 | 174662829,5 | 132882247,0 | 119520313,2 | 149502819,5 | 0,165 |
| LPS(18:0)_M-H | 12674367,0 | 11679912,8 | 14212525,5 | 11891095,8 | 10834525,3 | 13371992,0 | 0,353 |
| LPS(22:6)_M-H | 117277266,8 | 95848246,1 | 125104599,7 | 104033087,0 | 93183062,7 | 115160455,2 | 0,165 |
| Total LPS | 130148832,2 | 107528158,9 | 138435428,3 | 115223297,5 | 104466618,1 | 127644329,4 | 0,165 |
| PC(16:0/16:0)_M-CH3 | 126295826,8 | 109182931,0 | 131491767,9 | 119252063,7 | 105906838,4 | 132103597,4 | 0,796 |
| PC(16:0/16:0)_M+CH3COO-CH3 | 46447931,5 | 37030750,3 | 52691297,9 | 47520374,8 | 45379654,7 | 52292825,1 | 0,684 |
| PC(16:0_20:4)_M-CH3 | 71291056,5 | 66880161,7 | 78263842,4 | 68111238,2 | 63937621,6 | 77156924,2 | 0,353 |
| PC(16:0_20:4)_M+CH3COO | 828927923,1 | 783732735,1 | 903178021,9 | 800548652,9 | 747682281,3 | 880388726,9 | 0,481 |
| PC(16:0/22:4)_M+CH3COO | 69013649,4 | 65143529,2 | 72790900,0 | 66631919,4 | 65814435,6 | 72665820,6 | 0,796 |
| PC(18:0/16:1)_M+CH3COO | 60138731,5 | 57624092,1 | 67095053,3 | 63236944,7 | 59327336,5 | 68523234,7 | 0,481 |
| PC(16:0_18:1)_M+CH3COO // M+CH3COO | 59824525,0 | 57419595,7 | 66880232,6 | 62993621,6 | 59155973,6 | 68145083,1 | 0,436 |
| PC(16:1/22:6)_M+CH3COO | 12768800,8 | 12367428,3 | 13794853,0 | 12360238,2 | 11142912,2 | 14181363,7 | 0,353 |
| PC(17:0/16:0)_M+CH3COO | 14006450,9 | 12826153,5 | 15051955,0 | 14025637,3 | 13490548,9 | 14652475,7 | 1,000 |
| PC(17:0/18:1)_M+CH3COO | 17945613,5 | 17203000,6 | 19071622,4 | 18312175,8 | 17472242,8 | 19727243,5 | 0,631 |
| PC(18:0/18:1)_M-CH3 | 80045829,8 | 76413373,4 | 83259258,9 | 82138332,8 | 80209346,8 | 86528433,0 | 0,393 |
| PC(18:0/18:1)_M+CH3COO | 947357476,1 | 896412710,5 | 988074764,8 | 966754196,4 | 953432492,2 | 1023391639,0 | 0,393 |
| PC(18:0/20:4)_M-CH3 | 58579194,8 | 57229572,2 | 61638951,0 | 56103043,8 | 52851505,5 | 64832548,4 | 0,436 |
| PC(18:0/20:4)_M+CH3COO | 687865551,9 | 674157093,5 | 729884945,4 | 666307412,5 | 622021424,8 | 733075779,7 | 0,353 |
| PC(18:0/22:4)_M+CH3COO | 34961172,1 | 33351864,9 | 38964896,7 | 35082608,5 | 33619220,7 | 37356398,2 | 1,000 |
| PC(18:0/22:5)_M+CH3COO | 31847647,4 | 30874518,6 | 34563735,0 | 31979909,4 | 29937503,3 | 37165751,4 | 1,000 |
| PC(18:0/22:6)_M+CH3COO | 234804218,1 | 214196443,2 | 251563337,8 | 231573972,8 | 199952144,2 | 259732061,8 | 0,853 |
| PC(18:1/18:1)_M-CH3 | 20332983,2 | 19976582,7 | 22144872,8 | 21469106,8 | 19148683,0 | 22911895,4 | 0,912 |
| PC(18:1/18:1)_M+CH3COO | 316686055,6 | 301283687,5 | 323257248,6 | 319405609,7 | 288999771,4 | 341277849,7 | 0,796 |
| PC(18:1/20:4)_M+CH3COO | 148656634,6 | 134078866,4 | 170590736,2 | 143602430,9 | 128552723,8 | 153787307,1 | 0,579 |
| PC(18:1_22:0)_M+CH3COO | 28887892,9 | 24718113,2 | 30315703,9 | 28370733,2 | 25415171,9 | 32468806,7 | 0,579 |
| PC(22:6/22:6)_M+CH3COO | 40195638,2 | 38020398,1 | 47061811,5 | 37227791,2 | 34783539,3 | 45371041,9 | 0,280 |
| PC(30:0)_M-CH3 | 2410552,2 | 2069437,9 | 2844488,9 | 2048099,0 | 1913277,7 | 2753263,0 | 0,436 |
| PC(30:0)_M+CH3COO | 27000104,0 | 23315149,0 | 31521579,1 | 23465000,1 | 22240063,6 | 31739326,0 | 0,579 |
| PC(32:0)_M+CH3COO | 1368219261,5 | 1184190429,0 | 1413171900,3 | 1273894813,5 | 1141647643,0 | 1425007379,8 | 0,796 |
| PC(32:1)_M+CH3COO | 135760617,1 | 128252239,5 | 139833792,3 | 132932572,6 | 129196132,8 | 155437559,4 | 0,853 |
| PC(33:1)_M+CH3COO | 16905326,7 | 15945184,8 | 18065261,4 | 17409212,1 | 16736447,8 | 18277306,7 | 0,353 |
| PC(34:1)_M-CH3 | 231319257,7 | 217079423,1 | 239163382,2 | 231860593,3 | 207577580,6 | 239967676,5 | 1,000 |
| PC(34:1)_M+CH3COO | 2553344412,0 | 2392018823,3 | 2655891892,5 | 2587198645,0 | 2285709812,3 | 2637390274,5 | 0,912 |
| PC(34:2)_M+CH3COO | 42796095,5 | 38444684,6 | 50018928,2 | 46368317,6 | 40927362,5 | 52715087,0 | 0,436 |
| PC(34:5)_M-CH3 | 10038039,2 | 9441432,3 | 10777649,9 | 9767108,1 | 8799779,5 | 11098821,0 | 0,739 |
| PC(36:2)_M+CH3COO | 46522384,8 | 43684515,9 | 47878541,3 | 48812297,4 | 45830401,1 | 51853150,8 | 0,190 |
| PC(36:2)_M-CH3 | 29212392,4 | 25402928,7 | 30434391,3 | 31971420,4 | 29423981,4 | 36332756,0 | 0,043 |
| PC(36:3)_M+CH3COO | 17436971,8 | 16845710,0 | 18863540,4 | 18315363,5 | 14616033,9 | 21866278,5 | 0,796 |
| PC(16:0_20:3)_M+CH3COO | 41583372,6 | 40018945,5 | 42604992,6 | 42661372,0 | 39169249,6 | 45482612,4 | 0,481 |
| PC(37:4)_M+CH3COO | 9291048,1 | 9018663,2 | 9523574,1 | 9017837,6 | 8569753,2 | 9683524,8 | 0,280 |
| PC(38:2)_M+CH3COO | 25677883,2 | 22263690,8 | 27071004,0 | 27456478,6 | 24996360,6 | 29558432,8 | 0,190 |
| PC(38:3)_M+CH3COO | 13743427,1 | 13095283,4 | 14240390,4 | 13676283,6 | 13174660,8 | 14621793,6 | 0,971 |
| PC(38:6)_M+CH3COO | 600278267,6 | 557266461,3 | 624341628,1 | 581514052,9 | 520618027,7 | 643460628,0 | 0,579 |
| PC(40:5)_M+CH3COO | 10682486,6 | 9299018,5 | 11906322,7 | 10800561,9 | 9991807,3 | 11814462,8 | 0,796 |
| PC(40:6)_M-CH3 | 16179638,3 | 14698115,2 | 17175150,6 | 15922886,0 | 13148813,9 | 17594600,7 | 0,684 |
| PC(40:7)_M+CH3COO | 243178095,8 | 228298628,6 | 255201292,8 | 220450734,5 | 214274306,8 | 243073286,9 | 0,105 |
| PC(42:10)_M+CH3COO | 54076785,6 | 50576766,8 | 55855708,2 | 48345084,6 | 46052904,7 | 53257546,5 | 0,035 |
| PC(42:7)_M+CH3COO | 6503779,0 | 5818272,1 | 6749552,8 | 5835695,2 | 5503226,2 | 7235602,6 | 0,481 |
| PC(42:8)_M+CH3COO | 4180716,6 | 3870775,8 | 4921845,9 | 4274767,9 | 3966173,3 | 4647484,9 | 0,912 |
| PC(44:10)_M+CH3COO | 2457634,5 | 2159640,8 | 2579874,6 | 2351960,7 | 2142654,4 | 2643188,2 | 0,853 |
| Total PC | 9497493815,5 | 9017458439,0 | 9543821234,5 | 9346661875,0 | 8668072508,8 | 9600472244,8 | 0,912 |
| PCo(34:2)\|PCp(34:1)_M+CH3COO | 8383600,9 | 8088116,8 | 8531315,6 | 8404247,5 | 8061217,9 | 9128319,6 | 0,436 |
| PCo(34:1)\|PCp(34:0)_M+CH3COO | 37322974,6 | 34120698,1 | 41023188,6 | 38809368,3 | 36215125,8 | 43728002,7 | 0,315 |
| Total Pco | 44884407,0 | 42109593,9 | 49526832,6 | 46612623,3 | 44114149,6 | 53199495,8 | 0,218 |
| PE(16:0/20:3)_M-H | 8396909,5 | 7878979,9 | 8929239,4 | 8805861,5 | 8247622,2 | 9445480,0 | 0,280 |
| PE(16:0/20:4)_M-H | 123183102,7 | 109729395,6 | 140732769,3 | 117601454,9 | 109096711,6 | 131844703,7 | 0,739 |
| PE(16:0/22:4)_M-H | 97703741,9 | 89754036,7 | 110395442,0 | 106661368,8 | 90199101,7 | 116888541,4 | 0,529 |
| PE(16:0/22:5)_M-H | 43007802,4 | 38357682,4 | 45571203,3 | 43740232,2 | 40835052,9 | 48035843,5 | 0,481 |
| PE(16:0/22:6)_M-H | 969558480,3 | 932519684,7 | 999816598,2 | 958543921,5 | 901735431,5 | 1005818429,0 | 0,796 |
| PE(18:0_18:1)_M-H | 665447795,4 | 618249109,9 | 710107821,3 | 702048496,8 | 613707260,6 | 749283533,6 | 0,436 |
| PE(18:0/18:2)_M-H | 16061764,3 | 15490394,7 | 17008896,2 | 17635208,9 | 15843031,4 | 18359998,4 | 0,043 |
| PE(18:0/20:4)_M-H | 1565932268,5 | 1525038021,0 | 1614169748,8 | 1580648425,5 | 1520722950,0 | 1639793286,8 | 0,684 |
| PE(18:0/22:6)_M-H | 2732719237,5 | 2525732939,3 | 3120518273,8 | 2821949847,0 | 2733928893,8 | 3034752463,0 | 0,529 |
| PE(18:1/18:1)_M-H | 250260772,1 | 243329058,7 | 264502648,0 | 257165962,7 | 244961477,6 | 282813570,9 | 0,529 |
| PE(18:1/20:4)_M-H | 387933559,1 | 360402371,4 | 404861058,9 | 391759385,2 | 376818497,9 | 409697848,4 | 0,684 |
| PE(18:1_18:2)_M-H | 17106255,9 | 15792110,7 | 18006520,5 | 18523777,5 | 16856006,4 | 19936333,1 | 0,105 |
| PE(34:2)_M-H | 35589954,9 | 29959462,0 | 40090604,7 | 31084183,7 | 18691903,2 | 45133422,9 | 0,529 |
| PE(38:2)_M-H | 26068900,1 | 23159608,2 | 27800084,4 | 28949047,5 | 26104178,2 | 32997795,1 | 0,063 |
| Total PE | 7082556881,5 | 6537212489,5 | 7347536618,3 | 7147819071,5 | 6893506106,3 | 7222140741,0 | 0,796 |
| PEo(36:4)\|PEp(36:3)_M-H | 32951529,9 | 27385491,2 | 35896401,0 | 31912714,3 | 28751062,0 | 35425961,6 | 1,000 |
| PEo(38:6)\|PEp(38:5)_M-H | 111863261,6 | 105748811,1 | 117252154,3 | 113775291,8 | 104595652,7 | 123242551,5 | 0,796 |
| PEo(32:2)\|PEp(32:1)_M-H | 5934225,5 | 5103355,6 | 6459350,6 | 5334453,3 | 4901295,8 | 6682867,3 | 0,631 |
| PEo(16:1/20:3)\|PEp(16:0/20:3)_M-H | 18679453,1 | 16688821,3 | 20608078,1 | 18885403,9 | 17696562,8 | 24812557,6 | 0,739 |
| PEo(16:1/20:4)\|PEp(16:0/20:4)_M-H | 336818379,7 | 278211269,4 | 387529500,4 | 301803174,5 | 271861058,6 | 342747983,9 | 0,353 |
| PEo(16:1/20:4)\|PEp(16:0/20:4)_M+NaCH3COO | 18299468,1 | 15651157,1 | 19161617,8 | 16761635,9 | 14659079,5 | 17875478,5 | 0,143 |
| PEo(16:1/22:5)PEp(16:0/22:5)_M-H | 645867928,6 | 604765746,4 | 669487171,1 | 652543025,4 | 606568380,9 | 705771626,7 | 0,853 |
| PEo(16:1/22:5)\|PEp(16:0/22:5)_M-H | 46900624,5 | 37677672,8 | 60578022,0 | 53011266,2 | 39451973,4 | 67116295,0 | 0,739 |
| PEo(16:1/22:6)\|PEp(16:0_22:6)_M+NaCH3COO | 24916833,1 | 21273101,5 | 26395698,0 | 22468704,2 | 21009136,9 | 24188995,1 | 0,105 |
| PEo(16:1_22:6)\|PEp(16:0_22:6)_M-H | 1044933382,0 | 968286600,9 | 1068980181,3 | 969972981,7 | 908757179,0 | 999034552,3 | 0,035 |
| PEo(16:1_22:6)\|PEp(16:0_22:6)_M+NaCH3COO | 24871928,4 | 21231855,5 | 26375481,9 | 22420170,3 | 20966515,6 | 24139785,1 | 0,105 |
| PEo(18:1/22:6)\|PEp(18:0/22:6)_M-H // M-H | 38446248,5 | 37353043,0 | 39207136,3 | 37953173,9 | 35834823,6 | 43058614,7 | 1,000 |
| PEo(18:1/22:6)\|PEp(18:0/22:6)_M+NaCH3COO | 39620676,0 | 37798332,0 | 41654408,6 | 39164052,8 | 33224191,0 | 43064300,0 | 0,971 |
| PEo(18:1_22:6)\|PEp(18:0_22:6)_M-H | 1572716494,0 | 1456221611,5 | 1665626148,5 | 1557336298,0 | 1496821966,8 | 1603949851,0 | 0,912 |
| PEo(18:1_22:6)\|PEp(18:0_22:6)_M+NaCH3COO | 39629157,8 | 37880973,5 | 41654408,6 | 39210057,6 | 33321931,5 | 43066926,7 | 0,971 |
| PEo(18:2/16:1)\|PEp(18:1/16:1)_M-H | 31395516,0 | 29875612,9 | 33208257,2 | 31452701,2 | 30395522,7 | 35712832,4 | 0,631 |
| PEo(18:2/18:1)\|PEp(18:1/18:1)_M-H | 657043776,6 | 620634986,3 | 676555004,1 | 677412304,5 | 622565244,7 | 735590065,9 | 0,481 |
| PEo(18:2/18:2)\|PEp(18:1_18:2)\|PEp(18:2/18:1)_M-H | 16899004,3 | 14464452,4 | 18357039,3 | 18065094,5 | 15310683,9 | 21995134,5 | 0,436 |
| PEo(18:2/20:4)\|PEp(18:1/20:4)_M+NaCH3COO | 13438263,0 | 12223684,7 | 13737316,3 | 13289776,5 | 12774287,1 | 13785690,9 | 0,912 |
| PEo(18:2/22:6)\|PEp(18:1/22:6)_M-H | 676347629,4 | 647461820,4 | 731200234,9 | 663777764,4 | 640324546,7 | 707962212,5 | 0,481 |
| PEo(18:2/22:6)\|PEp(18:1/22:6)_M+NaCH3COO | 20961501,7 | 19607322,6 | 22201377,1 | 20646931,1 | 19447458,5 | 20971066,6 | 0,353 |
| PEo(35:2)\|PEp(17:0/18:1)_M-H | 22369409,5 | 19417338,2 | 24586488,1 | 24064191,3 | 19617079,2 | 25808120,7 | 0,353 |
| PEo(37:3)\|PEp(37:2)_M-H | 4592233,7 | 3915840,6 | 4893084,9 | 4733208,7 | 3990951,7 | 5836537,1 | 0,353 |
| PEo(38:5)\|PEp(38:4)_M-H | 233847306,4 | 216495826,4 | 252861773,2 | 236009862,2 | 214081116,3 | 249820363,2 | 1,000 |
| PEo(38:5)\|PEp(38:4)_M+NaCH3COO | 3979665,8 | 3288692,6 | 4253284,1 | 3744866,1 | 3427933,6 | 4022517,3 | 0,529 |
| PEo(39:5)\|PEp(17:0/22:4)_M-H | 7491217,5 | 6860404,8 | 8396193,9 | 7810578,4 | 7446106,5 | 8123783,0 | 0,631 |
| PEp(17:0/22:6)_M-H | 57347164,3 | 47941411,0 | 64099165,8 | 54402729,8 | 48039085,6 | 67695668,3 | 1,000 |
| Total PEo/Pep | 5685698164,0 | 5396093144,0 | 6036831383,5 | 5564329228,0 | 5476557521,3 | 5816470540,5 | 0,796 |
| PG(18:1/18:1)_M-H | 15814152,7 | 14432352,6 | 18395729,5 | 16463744,8 | 13830835,8 | 18198893,3 | 0,739 |
| PG(22:6/22:6)_M-H | 214846610,2 | 187242393,4 | 302849194,9 | 190556012,8 | 141494579,1 | 250869587,3 | 0,280 |
| PG(34:1)_M-H | 128297510,7 | 116269769,7 | 171183558,9 | 131270426,8 | 113775357,7 | 174601957,1 | 0,796 |
| PG(36:4)_M-H | 72034387,9 | 69519086,8 | 80057100,4 | 65616657,5 | 56658440,9 | 71261164,4 | 0,029 |
| PG(38:4)_M-H | 54273498,9 | 52482700,5 | 63637511,2 | 51821897,2 | 47849925,3 | 60511206,1 | 0,105 |
| PG(38:5)_M-H | 26919454,3 | 23412845,8 | 32387980,6 | 24961835,7 | 22587544,4 | 29619664,4 | 0,436 |
| PG(38:6)_M-H | 13548555,6 | 12109721,1 | 15871175,7 | 12917756,7 | 11196605,4 | 13613120,7 | 0,353 |
| Total PG | 521924695,2 | 476893957,7 | 683620156,3 | 490169440,7 | 409138058,3 | 608700116,6 | 0,247 |
| PI(16:0/20:4)_M-H | 513686909,0 | 502388216,4 | 644237075,0 | 532885415,9 | 443205345,7 | 591215702,5 | 0,529 |
| PI(18:0/20:4)_M-H | 2175922550,5 | 2064827224,5 | 2467637340,8 | 2180601399,5 | 1991917326,3 | 2522612450,8 | 0,796 |
| PI(18:0/22:6)_M-H | 84440892,8 | 71931443,4 | 91654977,1 | 76144362,5 | 65710355,9 | 97744891,1 | 0,739 |
| PI(18:1/18:1)_M-H | 21082578,5 | 17821840,6 | 23398384,9 | 21004583,9 | 18551058,0 | 23879648,4 | 1,000 |
| PI(18:1/20:4)_M-H | 440049824,1 | 404702079,7 | 499972709,3 | 436951365,8 | 381727852,7 | 467579880,4 | 0,579 |
| Total PI | 3178988883,0 | 3123027907,5 | 3704240845,0 | 3242288223,5 | 2902039816,3 | 3690832610,0 | 0,529 |
| PS(34:1)_M-H | 73750766,7 | 70897445,6 | 80301460,7 | 73349376,9 | 69822879,0 | 75300287,2 | 0,436 |
| PS(38:6)_M-H | 72944963,1 | 62239629,6 | 93389571,3 | 53522509,6 | 51209495,8 | 60251823,0 | <0,001 |
| PS(39:6)_M-H | 5661630,4 | 5505643,8 | 6018692,4 | 4965522,6 | 4840290,6 | 5430359,7 | 0,011 |
| PS(18:0/18:1)_M-H | 646895561,0 | 589665149,2 | 694121603,9 | 662759683,8 | 628408954,2 | 716310420,8 | 0,315 |
| PS(38:3)_M-H | 22882501,4 | 20548127,8 | 24464076,4 | 24070934,1 | 22431952,3 | 26008494,1 | 0,190 |
| PS(18:0/20:4)_M+Na-2H | 13014212,9 | 12111367,0 | 14687188,9 | 14196912,6 | 8773038,0 | 14734977,8 | 0,912 |
| PS(18:0/20:4)_M-H | 408019680,4 | 367864655,7 | 428168726,4 | 405059988,8 | 372385213,4 | 418277517,1 | 0,912 |
| PS(18:0/22:4)_M-H | 636609712,3 | 603893956,0 | 678465734,5 | 650218817,9 | 616640702,8 | 819279858,0 | 0,315 |
| PS(18:0/22:5)_M-H | 123723228,2 | 114755233,5 | 128174244,4 | 131701950,8 | 112108436,2 | 142595887,3 | 0,436 |
| PS(18:0/22:6)_M-H | 4166111012,0 | 4113862221,5 | 4252395453,0 | 3949331882,0 | 3793807623,8 | 4153238277,8 | 0,015 |
| PS(18:0/22:6)_M+Na-2H | 54768800,0 | 48459366,3 | 60085977,4 | 52532759,5 | 40897409,0 | 60253652,8 | 0,684 |
| PS(18:1/18:1)_M-H | 300452726,9 | 290056225,5 | 319685555,7 | 299500360,3 | 286079751,4 | 355905879,7 | 0,739 |
| PS(18:1/20:4)_M-H | 66735941,4 | 61925410,9 | 72518189,1 | 63146362,4 | 58948315,3 | 68812854,4 | 0,393 |
| PS(18:1/22:6)_M-H | 15060325,0 | 14282426,8 | 15324865,6 | 13920173,3 | 13203962,3 | 15526767,5 | 0,218 |
| PS(18:1/22:6))_M-H | 226574139,5 | 206662304,1 | 237651894,5 | 194879920,5 | 187750139,0 | 208100676,5 | 0,009 |
| PS(18:1_20:1)_M-H | 5275736,4 | 3585318,9 | 6179583,1 | 5096523,1 | 4363219,3 | 7085362,1 | 0,853 |
| PS(22:4/22:6)_M-H | 261561083,4 | 240476813,4 | 296625555,6 | 257266516,4 | 229492958,9 | 260299932,6 | 0,393 |
| PS(22:6/22:6)_M-H | 153363384,0 | 143186584,3 | 194644791,0 | 150222563,9 | 134809988,5 | 174143598,2 | 0,481 |
| PS(22:6/22:6)_M+Na-2H | 5470803,5 | 4848379,5 | 6705107,4 | 5007193,7 | 4803113,6 | 6095331,5 | 0,529 |
| PS(36:1)_M-H | 647917291,0 | 591072160,3 | 697707567,2 | 662078313,2 | 629330578,9 | 720422310,8 | 0,315 |
| PS(36:4)_M-H | 15230842,3 | 14488577,1 | 15956180,4 | 14172902,9 | 12539422,2 | 15307213,3 | 0,089 |
| PS(40:2)_M-H | 10162878,3 | 8362732,3 | 11329287,7 | 10523177,8 | 9592264,2 | 11152231,2 | 0,796 |
| PS(40:7)_M+Na-2H | 3935425,4 | 3682877,9 | 4349550,8 | 3573559,6 | 3348878,4 | 3704212,0 | 0,019 |
| PS(42:10)_M-H | 46788192,6 | 45002276,6 | 58493589,2 | 45029772,8 | 42296055,2 | 53045580,0 | 0,218 |
| PS(42:5)_M-H | 13327842,9 | 12750892,2 | 14287198,6 | 12985909,1 | 10903586,5 | 15328101,5 | 0,853 |
| PS(42:7)_M-H | 4385718,7 | 3486851,3 | 5311682,7 | 4177078,8 | 2439761,6 | 5607623,0 | 0,684 |
| PS(44:10)_M+Na-2H | 8408029,8 | 7972669,7 | 9790009,9 | 8250440,5 | 7740983,9 | 8951635,8 | 0,436 |
| PS(44:11)_M-H | 13887416,9 | 11585239,6 | 16114995,6 | 13527546,3 | 11825612,6 | 15341369,0 | 0,912 |
| PS(44:7)_M+Na-2H | 7684330,6 | 7098639,7 | 8654402,6 | 6566684,8 | 6240775,9 | 7281511,5 | 0,015 |
| Total PS | 8094171344,0 | 7853813282,3 | 8297115885,5 | 7885071492,0 | 7623400446,3 | 8134529762,5 | 0,165 |
