# Supplementary material for: Brain areas lipidomics in female transgenic mouse model of Alzheimer's disease
Source: Sci Rep. 2024 Jan 9;14:870. doi: 10.1038/s41598-024-51463-3 (PMC10776612; doi:10.1038/s41598-024-51463-3)
Supplement: Supplementary file 4 — Supplementary Tables. [file 41598_2024_51463_MOESM4_ESM.docx]

**Table S9.** Lipid variables selection using the LASSO regression, for positive ionization mode and each brain area.

| **Brain areas** | **Lipid family** | **Lipid variables selected** |
| --- | --- | --- |
| All areas (n=25) | GL | MAG(22:2) [M+NH4]^+^ |
|  | GL | DAG(18:0/18:0) [M+NH4] ^+^ |
|  | GL | DAG(18:0/18:0) [M+Na] ^+^ |
|  | GL | TAG(14:0/16:0/20:0) [M+NH4] ^+^ |
|  | GL | TAG(14:0/24:0/18:0) [M+NH4] ^+^ |
|  | GL | TAG(16:0/16:0/18:2) [M+Na] ^+^ |
|  | GL | TAG(16:0/16:0/20:1) [M+NH4] ^+^ |
|  | GL | TAG(16:0/16:0/20:1) [M+Na] ^+^ |
|  | GL | TAG(18:1/18:1/18:3) [M+Na] ^+^ |
|  | GP | PC(14:0_16:0) [M+H] ^+^ |
|  | GP | PC(16:0/16:0) [M+Na] ^+^ |
|  | GP | PC(18:1/16:1) [M+Na] ^+^ |
|  | GP | PC(18:1/16:1) [M+H] ^+^ |
|  | GP | PC(31:0) [M+H] ^+^ |
|  | GP | PC(36:4) [M+H] ^+^ |
|  | GP | PC(40:7) [M+Na] ^+^ |
|  | GP | PE(22:6/16:0) [M+Na] ^+^ |
|  | GP | LPC(18:0) [M+H] ^+^ |
|  | GP | PCp(34:6) [M+H] ^+^ |
|  | GP | PEo(16:1_22:6) [M+Na] ^+^ |
|  | GP | PEo(18:2_22:4) [M+H] ^+^ |
|  | SP | Cer(d18:1/18:0) [M+H] ^+^ |
|  | SP | Cer(d18:1/24:1) [M+H-H2O] ^+^ |
|  | SP | SM(d18:1/17:0) [M+Na] ^+^ |
|  | SP | SM(d38:1) [M+H] ^+^ |
| Cerebellum (n=11) | GL | MAG(20:1) [M+NH4] ^+^ |
|  | GL | DAG(16:0/18:2) [M+H-H2O] ^+^ |
|  | GL | DAG(18:0/18:0) [M+H-H2O] ^+^ |
|  | GL | DAG(18:0/18:0) [M+Na] ^+^ |
|  | GL | DAG(18:0/20:4) [M+NH4] ^+^ |
|  | GL | TAG(12:0/15:0/18:0) [M+NH4] ^+^ |
|  | GL | TAG(17:0/17:0/17:0) [M+Na] ^+^ |
|  | GP | LPC(18:0) [M+H] ^+^ |
|  | GP | PEo(36:4) [M+H] ^+^ |
|  | GP | PEo(38:4) [M+H] ^+^ |
|  | SP | Cer(d18:1/24:1) [M+H] ^+^ |
| Amygdala (n=4) | GL | MAG(20:2) [M+NH4] ^+^ |
|  | GL | DAG(18:0/22:4) [M+Na] ^+^ |
|  | GP | PEo(18:2/22:6) [M+Na] ^+^ |
|  | SP | Cer(d18:1/17:0) [M+Na] ^+^ |
| Hippocampus (n=3) | GP | PC(20:1/22:6) [M+H] ^+^ |
|  | GP | PEo(18:1/20:4) [M+Na] ^+^ |
|  | GP | PG(34:1) [M+Na] ^+^ |
| Cortex (n=10) | GL | DAG(16:0/20:4) [M+Na] ^+^ |
|  | GL | DAG(18:0/18:0) [M+H-H2O] ^+^ |
|  | GL | TAG(18:2/18:1/15:0) [M+Na] ^+^ |
|  | GP | PC(18:1/16:1) [M+Na] ^+^ |
|  | GP | PC(40:7) [M+H] ^+^ |
|  | GP | PC(40:7) [M+Na] ^+^ |
|  | GP | PE(22:6/18:0) [M+Na] ^+^ |
|  | GP | LPC(16:0) [M+H] ^+^ |
|  | GP | PEo(16:1_22:6) [M+Na] ^+^ |
|  | GP | PEo(36:5) [M+Na] ^+^ |

GL: glycerolipid; GP: glycerophospholipid; SP: sphingolipid.

**Table S10.** Lipid variables selection LASSO regression, for negative ionization mode and each brain area.

| **Brain areas** | **Lipid family** | **Lipid variables selected** |
| --- | --- | --- |
| All areas  (n=23) | FA | FA(20:0) [M-H]^-^ |
|  | FA | FA(24:0) [M-H] ^-^ |
|  | GP | PC(18:0/22:4) [M+CH3COO] ^-^ |
|  | GP | PC(30:0) [M-CH3] ^-^ |
|  | GP | PC(32:0) [M+CH3COO] ^-^ |
|  | GP | PC(34:5) [M-CH3] ^-^ |
|  | GP | PC(36:2) [M+CH3COO] ^-^ |
|  | GP | PC(36:3] [M+CH3COO] ^-^ |
|  | GP | PC(16:0_20:3] [M+CH3COO] ^-^ |
|  | GP | PC(40:5) [M+CH3COO] ^-^ |
|  | GP | PE(18:1_18:2) [M-H] ^-^ |
|  | GP | PE(38:2) [M-H] ^-^ |
|  | GP | PG(22:6/22:6) [M-H] ^-^ |
|  | GP | PS(18:0/20:3] [M-H] ^-^ |
|  | GP | PS(18:0/20:4) [M+Na-2H] ^-^ |
|  | GP | PS(18:0/22:4) [M-H] ^-^ |
|  | GP | LPE(18:0) [M-H] ^-^ |
|  | GP | PEo(16:1/20:4) [M-H] ^-^ |
|  | GP | PEo(18:1/22:6) [M-H] ^-^ |
|  | GP | PEo(18:2/20:4) [M+NaCH3COO] ^-^ |
|  | GP | CL(18:1/18:1/18:1/18:1) [M-H] ^-^ |
|  | GP | CL(18:1_20:4_22:6_22:6) [M-H] ^-^ |
|  | SP | Cer(d18:1/18:0) [M+CH3COO] ^-^ |
| Cerebellum (n=11) | FA | FA(17:0) [M-H] ^-^ |
|  | GP | PC(18:0/20:4) [M+CH3COO] ^-^ |
|  | GP | Total PC |
|  | GP | PE(38:2) [M-H] ^-^ |
|  | GP | PS(17:0/22:6) [M-H] ^-^ |
|  | GP | PS(18:0/22:4) [M-H] ^-^ |
|  | GP | PS(18:0/22:5) [M-H] ^-^ |
|  | GP | LPI(18:0) [M-H] ^-^ |
|  | GP | LPS(18:0) [M-H] ^-^ |
|  | GP | PEp(16:0/22:5) [M-H] ^-^ |
|  | SP | Cer(d18:1/20:0) [M+CH3COO] ^-^ |
| Amygdala  (n=10) | FA | FA(18:0) [M-H] ^-^ |
|  | GP | PC(34:1) [M+CH3COO] ^-^ |
|  | GP | PC(38:6) [M+CH3COO] ^-^ |
|  | GP | PC(40:5) [M+CH3COO] ^-^ |
|  | GP | PE(16:0/20:3] [M-H] ^-^ |
|  | GP | PS(22:6/22:6) [M-H] ^-^ |
|  | GP | Total PS |
|  | GP | PEo(16:1/16:1) [M-H] ^-^ |
|  | GP | PEp(16:0/22:5) [M-H] ^-^ |
|  | SP | Cer(d18:1/16:0) [M+CH3COO] ^-^ |
| Hippocampus (n=5) | FA | FA(18:2) [M-H] ^-^ |
|  | GP | PS(42:5) [M-H] ^-^ |
|  | GP | PEo(16:0/20:4) [M-H] ^-^ |
|  | GP | CL(18:1_18:1_22:6_22:6) [M-H] ^-^ |
|  | GP | CL(18:1_20:4_22:6_22:6) [M-H] ^-^ |
| Cortex  (n=2) | GP | PS(16:0/22:6) [M-H] ^-^ |
|  | GP | PS(18:0/22:6) [M-H] ^-^ |

FA: fatty acid; GP: glycerophospholipid; SP: sphingolipid.
